# Mechanism of the cooperative Si–H bond activation at Ru–S bonds†
†Electronic supplementary information (ESI) available: Experimental procedures and computational details, characterization, crystallographic and quantum-chemical calculation data as well as NMR spectra. CCDC 1055362–1055364. For ESI and crystallographic data in CIF or other electronic format see DOI: 10.1039/c5sc01035g


**DOI:** 10.1039/c5sc01035g

**Published:** 2015-05-18

**Authors:** Timo Stahl, Peter Hrobárik, C. David F. Königs, Yasuhiro Ohki, Kazuyuki Tatsumi, Sebastian Kemper, Martin Kaupp, Hendrik F. T. Klare, Martin Oestreich

**Affiliations:** a Institut für Chemie , Technische Universität Berlin , Straße des 17. Juni 115 , 10623 Berlin , Germany . Email: peter.hrobarik@tu-berlin.de ; Email: hendrik.klare@tu-berlin.de ; Email: martin.oestreich@tu-berlin.de; b Department of Chemistry , Graduate School of Science and Research Center for Materials Science , Nagoya University , Furo-cho, Chikusa-ku , Nagoya 464-8602 , Japan

## Abstract

The nature of the hydrosilane activation mediated by ruthenium(ii) thiolate complexes of type [(R_3_P)Ru(SDmp)]^+^[BAr^F^_4_]^–^ is elucidated by an in-depth experimental and theoretical study. The combination of various ruthenium(ii) thiolate complexes and tertiary hydrosilanes under variation of the phosphine ligand and the substitution pattern at the silicon atom is investigated, providing detailed insight into the activation mode. The mechanism of action involves reversible heterolytic splitting of the Si–H bond across the polar Ru–S bond without changing the oxidation state of the metal, generating a ruthenium(ii) hydride and sulfur-stabilized silicon cations, *i.e.* metallasilylsulfonium ions. These stable yet highly reactive adducts, which serve as potent silicon electrophiles in various catalytic transformations, are fully characterized by systematic multinuclear NMR spectroscopy. The structural assignment is further verified by successful isolation and crystallographic characterization of these key intermediates. Quantum-chemical analyses of diverse bonding scenarios are in excellent agreement with the experimental findings. Moreover, the calculations reveal that formation of the hydrosilane adducts proceeds *via* barrierless electrophilic activation of the hydrosilane by sterically controlled η^1^ (end-on) or η^2^ (side-on) coordination of the Si–H bond to the Lewis acidic metal center, followed by heterolytic cleavage of the Si–H bond through a concerted four-membered transition state. The Ru–S bond remains virtually intact during the Si–H bond activation event and also preserves appreciable bonding character in the hydrosilane adducts. The overall Si–H bond activation process is exergonic with Δ*G*0r ranging from –20 to –40 kJ mol^–1^, proceeding instantly already at low temperatures.

## Abstract 2

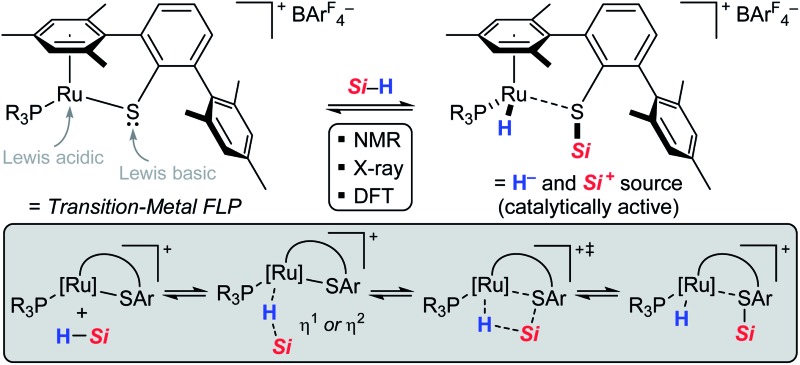
The heterolytic splitting of hydrosilanes by ruthenium(ii) thiolates is illuminated by a combined spectroscopic, crystallographic, and computational analysis.

## Introduction

**Scheme 1:**
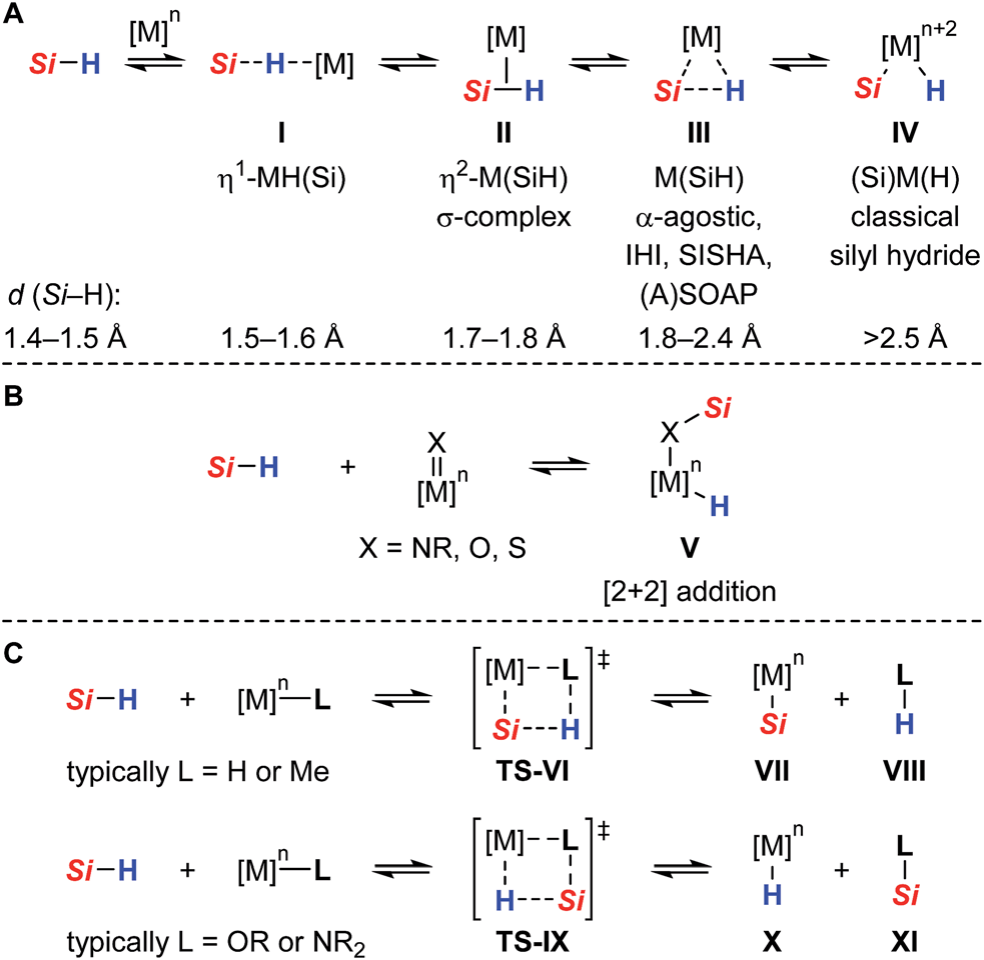
Diverse bonding scenarios in the Si–H bond activation by transition metal complexes [IHI = interligand hypervalent interactions, SISHA = secondary interactions between a silicon and a hydrogen atom, (A)SOAP = (a)symmetric oxidation addition products, and *Si* = R_3_Si = triorganosilyl].

Neutral tetracoordinate hydrosilanes do generally not undergo spontaneous reactions with organic substrates and require the activation of the Si–H bond. Among the diverse modes that are known for hydrosilane activation,^1^ protocols using transition metal complexes clearly prevail. Numerous transition metal hydrosilane complexes were structurally characterized and identified as key intermediates in various catalytic processes.^2^ The different bonding motifs in these structures reveal a continuum along the Si–H bond activation pathway, ranging from several nonclassical (three-center-two-electron) interactions to full homolytic cleavage of the Si–H bond (**I** → **IV**, Scheme 1A).^3^ Since oxidative addition typically leads to an increase of both the formal oxidation state and coordination number of the metal (*cf.***IV**), this mechanism is usually invoked with low-valent, electron-rich transition metal complexes, particularly those of groups 9 and 10. Alternatively, the Si–H bond is heterolytically split without change in the oxidation state of the metal to formally generate a hydride (H^–^) and a silylium ion (R_3_Si^+^).^4^ This event is favored with electrophilic (cationic) metal centers and requires assistance of a Lewis base. By coordination to the Lewis acidic metal center, through either binding modes **I**, **II**, and **III**, the silicon atom is rendered sufficiently electrophilic to react with a wide variety of nucleophiles. In the absence of any externally added Lewis base, heterolytic cleavage of the Si–H bond is also facilitated by cooperative metal–ligand interactions where the ancillary ligand serves as an internal Lewis-basic site and is directly involved in the Si–H bond activation process.^5^ In this case, a mechanism following a [2 + 2]-type cycloaddition (Scheme 1B) or a σ-bond metathesis (Scheme 1C) is generally postulated.^6^ In the former scenario, well-defined addition of the Si–H bond across a metal–ligand multiple bond results in the formation of a metal hydride complex in which the silyl group is incorporated (*cf.***V**). In the latter, the product distribution strongly depends on the nature of the metal–ligand combination. While σ-bond metathesis *via* concerted transition state **TS-VI** (Scheme 1C, upper) leads to a metal silyl complex **VII** with release of side product **VIII** (typically dihydrogen or methane), reaction *via***TS-IX** (Scheme 1C, lower) produces a metal hydride **X** with concomitant dissociation of silylated ligand **XI** (typically a silyl ether or silyl amine).

Compared to the classical oxidative addition pathway commonly proposed, heterolytic Si–H bond activation with transition metals is less well-established and was initially postulated by Luo and Crabtree in the iridium(iii)-catalyzed alcoholysis of hydrosilanes.^7^ Since this report, several transition metal mediated heterolyses of Si–H bonds have been documented.^8^ In most cases, however, a highly reactive silicon electrophile was generated through coordination to a cationic metal center and subsequently trapped in a less controlled manner by intermolecular reaction with adventitious water, the solvent (*e.g.* CH_2_Cl_2_), or counteranion (*e.g.* OTf^–^, SbF_6_^–^ or BF_4_^–^). A few examples of intramolecular cooperative Si–H bond activation were shown where the Si–H bond is preferentially split across a polar M=X double bond (*cf.*Scheme 1B with X = NR, O, S), using early or middle transition metals in high oxidation states where oxidative addition is not possible or unlikely [*e.g.* Ti(iv), Ta(v), Re(v)].8f–o Although this activation mode allowed for the isolation and crystallographic characterization of unique hydrosilane adducts, as specified by complex **V**, its synthetic use and application to catalytic processes is still out of the ordinary and mainly restricted to hydrosilylation reactions.^8^

**Scheme 2:**
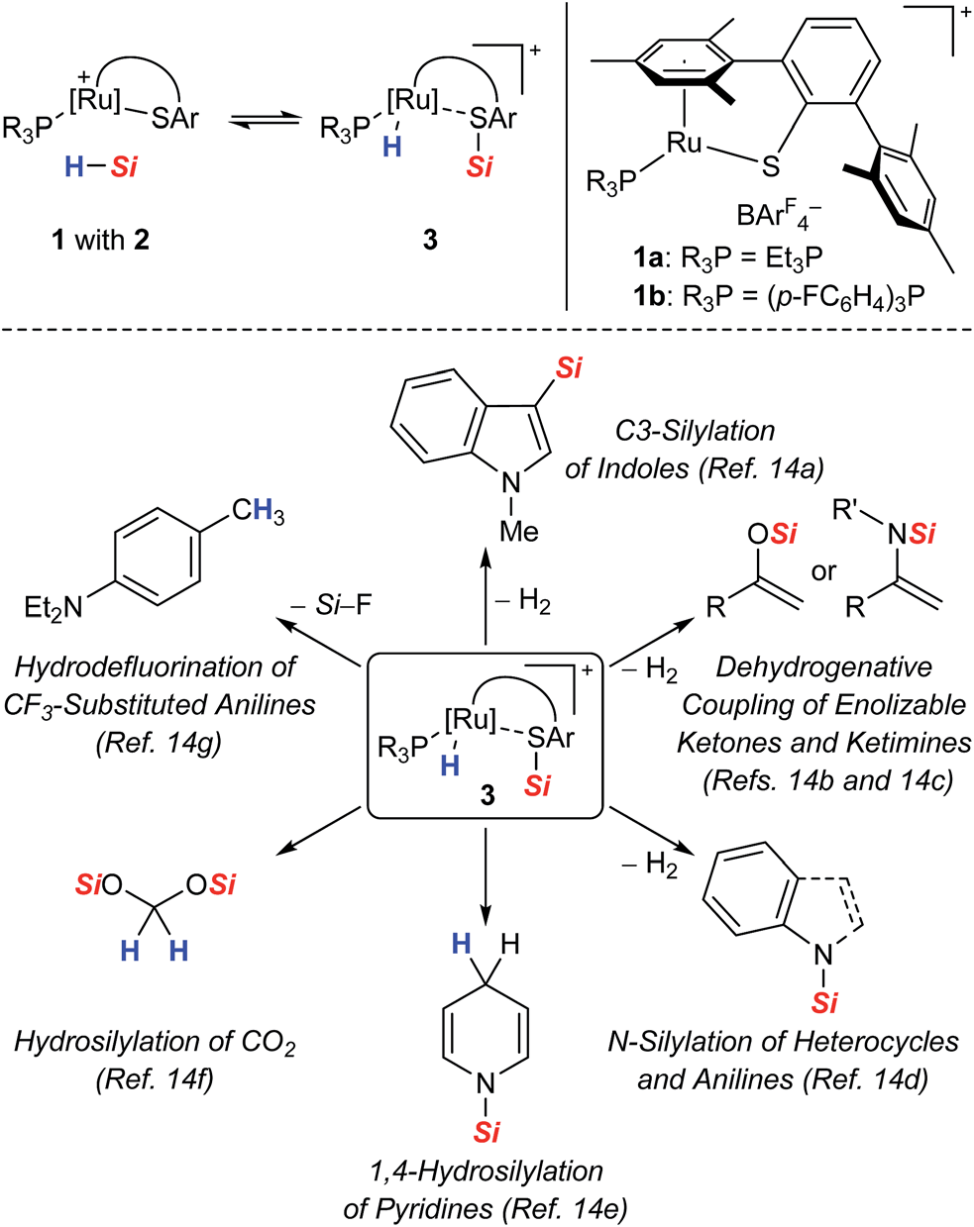
Proposed cooperative Si–H bond heterolysis by ruthenium(ii) thiolate complexes **1** (top) and reported catalytic applications (bottom).

As part of our ongoing research endeavors of exploring new approaches to the generation of silicon electrophiles, we introduced the cationic ruthenium(ii) thiolate complexes **1** (with Ar^F^ = 3,5-bis(trifluoromethyl)phenyl, Scheme 2, top right).^9^ The tethered bulky 2,6-dimesitylphenyl thiolate (SDmp) ligand stabilizes the coordinatively unsaturated metal center in **1** and also prevents formation of binuclear sulfur-bridged complexes. The polar Ru–S bond of these (formally) 16-electron complexes combines Lewis acidity at the metal center and Lewis basicity at the adjacent sulfur atom. We reasoned that this motif that could be considered as a transition metal frustrated Lewis pair (FLP)^10^ mediates the heterolytic cleavage of Si–H bonds, generating a metal hydride and a sulfur-stabilized silicon cation (Scheme 2, top left).^11^ This assumption was corroborated by a related study of Stradiotto and co-workers, reporting the addition of the Si–H bond of Ph_2_SiH_2_ and PhSiH_3_ across the M–S bond of cationic [Cp*M(κ^2^-3-PiPr_2_-2-S-indene)]^+^[B(C_6_F_5_)_4_]^–^ complexes (with M = Rh^III^ and Ir^III^).^12^ Compared to *harder* nitrogen or oxygen donors, the interaction with the *soft* sulfur atom was expected to give a more reactive silicon electrophile.11b,^13^ In addition, weak stabilization ought to favor reversible coordination, thereby facilitating R_3_Si^+^-transfer and securing turnover in catalytic processes. The rationally designed complexes **1** indeed proved to serve as potent catalysts for the facile activation not only of Si–H^14^ but also H–H^9^ and B–H^15^ bonds. Since our initial report on Si–H bond activation,14*a* we have disclosed a number of catalytic transformations, including dehydrogenative silylations,14a–d chemoselective hydrosilylations,14e,f as well as hydrodefluorination reactions14*g* (Scheme 2, bottom). It is interesting that Stradiotto and co-workers had been able to apply their rhodium(iii) thiolate complex in ketone hydrosilylation (not shown)^12^ where we later observed dehydrogenative silyl enol ether formation with our system.14*b* All catalyses proceeded already at room temperature, neither requiring a hydrogen acceptor nor an added base for the catalytic cycle to close.

Encouraged by the broad spectrum of catalytic reactions that were accomplished by using a single catalyst, we pursued an in-depth analysis of the Si–H bond activation event by ruthenium(ii) thiolate complexes **1**. Since the activation mode naturally impacts the course and outcome of a reaction, its mechanistic understanding is of vital importance and provides useful insights for the future design and development of more efficient catalysts.

**Chart 1:**
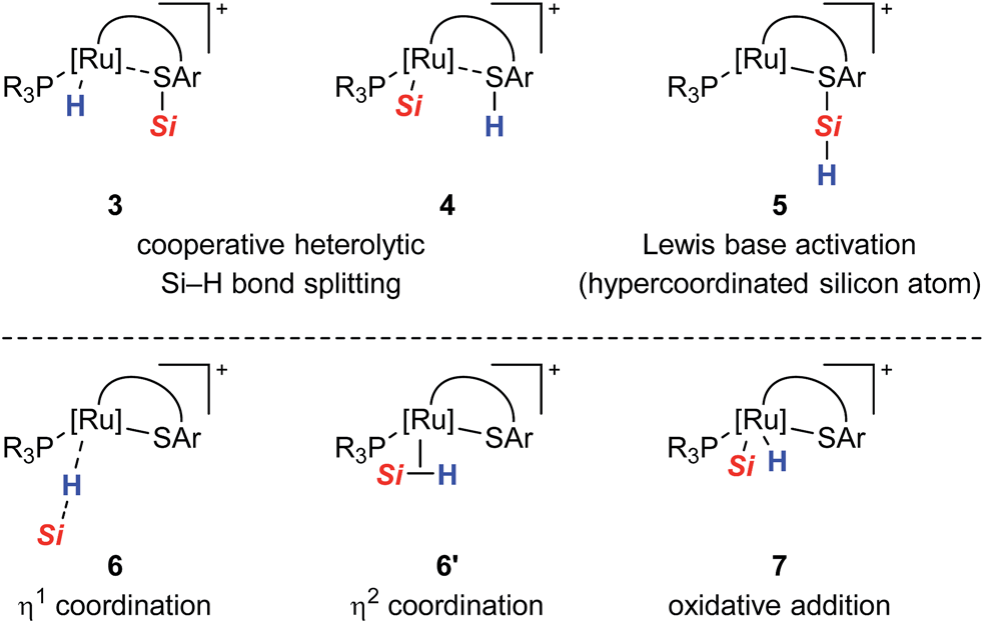
Possible modes for the Si–H bond activation by ruthenium(ii) thiolate complexes **1** with (top) and without (bottom) participation of the sulfur atom.

All proposed mechanistic assumptions have so far relied on the heterolytic splitting of the Si–H bond at the polar Ru–S bond in **1** (*cf.*Scheme 2, top left) and have been primarily based on the interpretation of experimental observations.^12^,^14^ In view of the fact that the ruthenium(ii) metal center is coordinatively unsaturated and in low oxidation state several possibilities of Si–H bond activation exist (Chart 1). Aside from the proposed cooperative activation mode (*cf.***3** in Chart 1), addition of the Si–H bond across the Ru–S bond with reversed regioselectivity might occur, leading to metal silyl complex **4** (*cf.*Scheme 1C). Since tetracoordinate silicon readily expands its coordination sphere, activation of the hydrosilane through coordination to the Lewis basic sulfur atom as in **5** needs to be considered. Moreover, pathways without participation of the sulfur atom are another option. These could proceed *via* electrophilic activation of the Si–H bond either by η^1^ (end-on) or η^2^ (side-on) coordination (*cf.***6** and **6′**, respectively), followed by heterolytic cleavage of the Si–H bond by an externally added nucleophile. Likewise, full homolytic cleavage of the hydrosilane *via* classical oxidative addition affording metal silyl hydride **7** cannot be ignored. Therefore, several mechanistic questions have remained, including the role of the thiolate ligand.^12^

We report here the results of our mechanistic study on hydrosilane activation promoted by ruthenium(ii) thiolate complexes **1**. By using various NMR techniques, we were able to detect the key intermediate of the Si–H bond activation step and fully assign its structure. The mechanism was further elucidated with the aid of a deuterium-labeled and a silicon-stereogenic hydrosilane as a stereochemical probe. Supported by quantum-chemical calculations, the experimental findings provide compelling evidence for a cooperative Si–H bond activation pathway that is in accordance with our originally formulated mechanistic model (*cf.*Scheme 2, top left)^14^ and Stradiotto's work.^12^ Finally, we succeeded in the isolation and crystallographic characterization of the sensitive silylthioruthenium hydride intermediate **3**.

## Results and discussion

From our previous studies, we already knew that Si–H bond activation with cationic ruthenium(ii) thiolate complexes **1** is remarkable facile, proceeding instantly at room temperature.^14^ The tethered coordination mode of the SDmp ligand proved to be crucial: while the lability of a monodentate thiolate ligand in related rhodium(iii) and iridium(iii) complexes^16^ resulted in decomposition and formation of various metal hydride species in the presence of hydrosilanes, the two-point binding mode in **1** imparts increased stability to the Ru–S bond. In addition, this motif leads to structural rigidity and improved steric accessibility of the Ru–S bond, as seen in the molecular structure of these complexes.^9^

### Elucidation of the Si–H bond activation by NMR spectroscopy

**Scheme 3:**
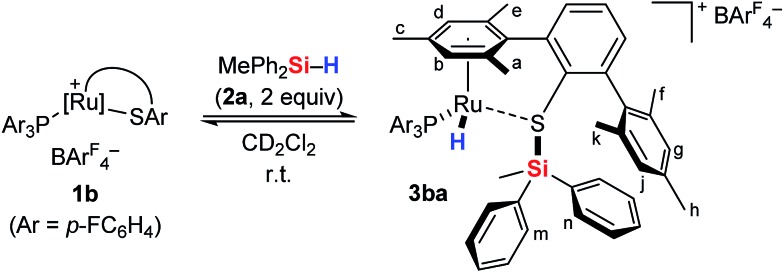
Cooperative Si–H bond heterolysis with ruthenium(ii) thiolate complex **1b**.

To gain deeper insight into the Si–H bond activation, we performed a detailed NMR study using one- and two-dimensional multinuclear NMR measurements. In an initial investigation, monitoring the reaction of ruthenium(ii) thiolate complex **1a** (R_3_P = Et_3_P) with hydrosilanes under typical catalysis conditions (room temperature, excess hydrosilane) emerged as difficult since dynamic processes led to significant line-broadening in the NMR spectra.14*a* However, we identified the combination of complex **1b** where the ruthenium atom is coordinated by a *para*-fluorinated aryl phosphine and MePh_2_SiH (**2a**) to be particularly suitable for NMR studies. Treatment of **1b** with two equivalents of MePh_2_SiH (**2a**) at ambient temperature resulted in an immediate color change from green to yellow, “visualizing” successful Si–H bond activation. Clearly resolved spectra, obtained in both C_6_D_6_ and CD_2_Cl_2_ (with slightly better resolution in CD_2_Cl_2_, see the ESI† for details) were consistent with formation of hydrosilane adduct **3ba** (**1b** → **3ba**, Scheme 3).

**Fig. 1:**
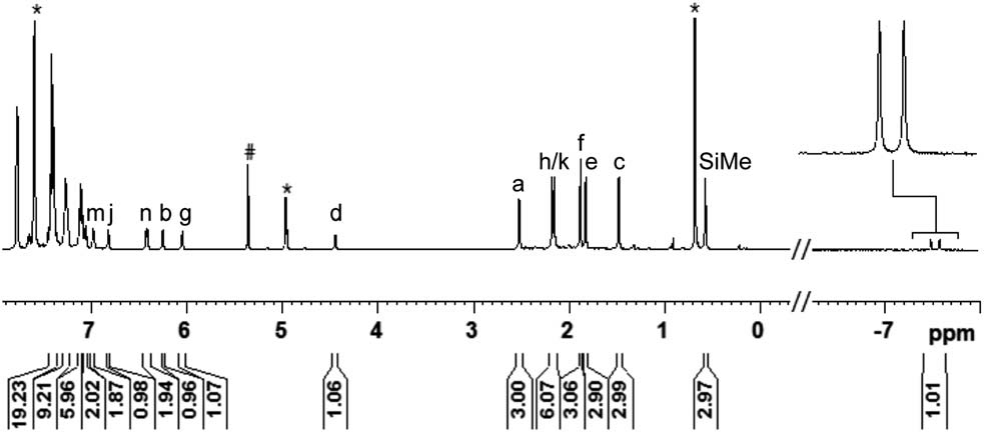
^1^H NMR spectrum (500 MHz, CD_2_Cl_2_, 300 K) of adduct **3ba**, formed by reaction of complex **1b** and hydrosilane **2a** [# = CDHCl_2_, * = excess MePh_2_SiH (**2a**)].

The reaction proceeded cleanly, and resonance signals of unsaturated ruthenium(ii) thiolate complex **1b** were no longer detectable. Instead, a doublet at –7.5 ppm in the ^1^H NMR spectrum (Fig. 1) provided unambiguous evidence for a phosphine-ligated ruthenium hydride (ruling out intermediates **4** and **5**). The absence of any ^1^*J*_H,Si_ satellites, typically observed for η^1^ and η^2^ hydrosilane complexes, further supported complete Si–H bond cleavage (making **6** and **6′** unlikely). The coupling constant of 47 Hz is in the typical range for ^2^*J*_H,P_ couplings and was also identified in the ^31^P NMR spectrum at 48.5 ppm whereas a singlet is observed at 30.0 ppm for complex **1b**. While the latter is *C*_s_ symmetric, the additional hydride ligand creates a chiral center at the ruthenium atom in **3ba**. This is also reflected in the ^1^H NMR spectrum that shows six rather than four singlets for the methyl groups and four rather than two signals for the *meta*-CH groups of the SDmp ligand (Fig. 1).

Using a ^1^H, ^29^Si HMQC experiment, a ^29^Si NMR resonance signal was detected at 20.1 ppm, significant downfield shifted (Δ*δ* = 37.6 ppm) relative to free MePh_2_SiH (**2a**, *δ* = –17.5 ppm). This is an indication for the generation of an electrophilic silicon species and is in accordance with postulated intermediate **3ba**.^17^ For comparison, the corresponding neutral silyl thioether MePh_2_SiSDmp (for its preparation and characterization, see the ESI†) that features an S–Si linkage without an adjacent metal center is characterized by a ^29^Si NMR chemical shift of 2.9 ppm [Δ*δ* = 20.4 ppm relative to MePh_2_SiH (**2a**)]. The substantial deshielding of the silicon nucleus in hydrosilane adduct **3ba** reveals a strong influence of the metal center on its Lewis acidity that is expected to be higher compared to MePh_2_SiSDmp. The latter is in fact not a potent silyl transfer agent.

All NMR signal assignments were also confirmed by our state-of-the-art relativistic calculations of NMR chemical shifts at the four-component matrix Dirac–Kohn–Sham (mDKS) level^18^ (*cf.*Table 1 and S2 in the ESI† for detailed data). Spin–orbit (SO) effects were found to have a sizeable shielding contribution to both ^1^H (up to –3.1 ppm) and ^31^P (up to –35 ppm) NMR chemical shifts, owing to the large involvement of both ruthenium 4d-orbitals and ligand s-orbitals in metal–ligand binding.^18^,^19^

**Table 1:** Experimental and calculated ^1^H, ^29^Si, and ^31^P NMR chemical shifts of hydrosilane adducts **3**^*a*^

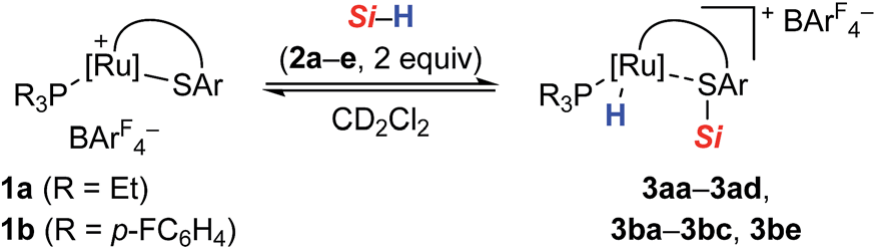										
Entry	Reaction	^1^H NMR			^29^Si NMR^*c*^			^31^P NMR		
		*δ*(Si–H) [ppm]	*δ*(Ru–H)^*d*^ [ppm]	Δ*δ*(^1^H)^*e*^ [ppm]	*δ*(Si–H) [ppm]	*δ*(S–Si) [ppm]	Δ*δ*(^29^Si)^*f*^ [ppm]	*δ*(**1**) [ppm]	*δ*(**3**) [ppm]	Δ*δ*(^31^P)^*g*^ [ppm]
1^*h*^	**1a** + MePh_2_SiH (**2a**) → **3aa**	4.8	–8.2 (–7.8)^*b*^	–13.0	–17.5	18.2 (14.8)^*b*^	35.7	23.0 (23.0)^*b*^	39.8 (37.2)^*b*^	16.8
2^*h*^	**1a** + Me_2_PhSiH (**2b**) → **3ab**	4.4	–8.3 (–7.9)^*b*^	–12.7	–17.0	28.4 (33.5)^*b*^	45.4	23.0	40.4 (36.7)^*b*^	17.4
3^*i*^	**1a** + Et_3_SiH (**2c**) → **3ac**	3.7	–8.0	–11.7	0.4	41.0	40.6	23.0	40.1	17.1
4^*i*^	**1a** + EtMe_2_SiH (**2d**) → **3ad**	3.7	–8.1 (–7.8)^*b*^	–11.8	–10.7	39.0 (44.1)^*b*^	49.7	23.0	40.2 (38.2)^*b*^	17.2
5^*i*^	**1b** + MePh_2_SiH (**2a**) → **3ba**	4.8	–7.5 (–7.6)^*b*^	–12.3	–17.5	20.1 (28.3)^*b*^	37.6	30.0 (31.6)^*b*^	48.5 (39.7)^*b*^	18.5
6^*i*^	**1b** + Me_2_PhSiH (**2b**) → **3bb**	4.4	–7.7 (–7.5)^*b*^	–12.1	–17.0	29.8 (33.3)^*b*^	46.8	30.0	48.7 (41.3)^*b*^	18.7
7^*i*^	**1b** + Et_3_SiH (**2c**) → **3bc**	3.7	–7.6	–11.3	0.4	41.6	41.2	30.0	48.8	18.8
8^*i*^	**1b** + iPrMePhSiH (**2e**) → **3be**	4.3	–7.7	–12.0	–6.4	32.1	38.5	30.0	47.2	17.2

^*a*^All reactions were performed in an NMR tube using ruthenium(ii) thiolate complex **1a** or **1b** (1.0 equiv., 20 mM) and the corresponding hydrosilane **2** (2.0 equiv.).

^*b*^In parentheses, NMR chemical shifts calculated at the four-component mDKS level using the PBE functional and the GIAO method in conjunction with Dyall's VDZ basis set on Ru and fully uncontracted IGLO-II basis sets on the ligand atoms (*cf.* computational details and Table S2 in the ESI†).

^*c*^ ^1^H, ^29^Si HMQC NMR spectroscopy optimized for *J* = 8 Hz.

^*d*^The resonance appears as a doublet with a coupling constant of 47–50 Hz.

^*e*^Δ*δ*(^1^H) = *δ*(Ru–H) – *δ*(Si–H).

^*f*^Δ*δ*(^29^Si) = *δ*(S–Si) – *δ*(Si–H).

^*g*^Δ*δ*(^31^P) = *δ*(**3**) – *δ*(**1**).

^*h*^In CD_2_Cl_2_ at 250 K.

^*i*^In CD_2_Cl_2_ at 300 K.

**Fig. 2:**
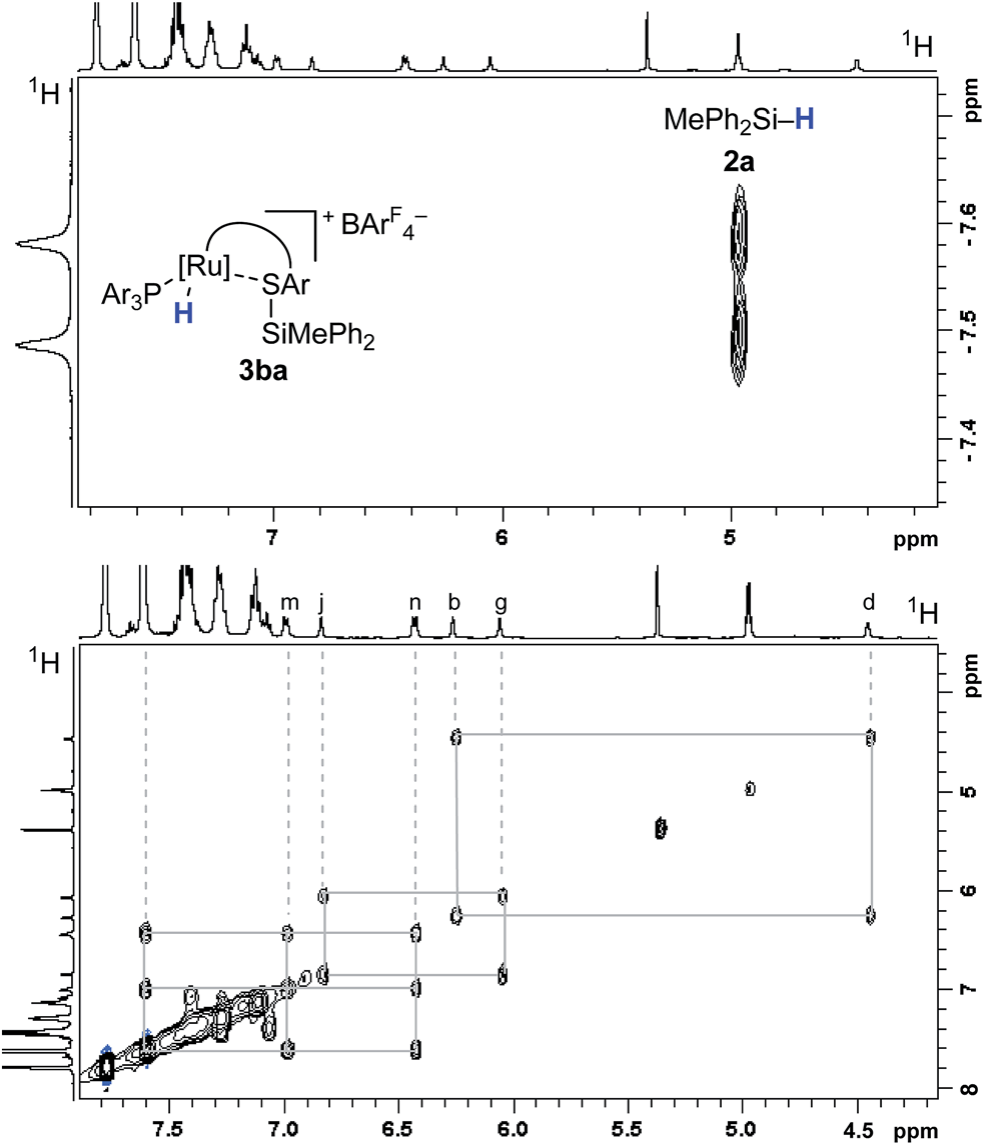
Selected segments of the ^1^H, ^1^H EXSY NMR spectrum (500/500 MHz, CD_2_Cl_2_, 300 K, *T*_m_ = 200 ms) of adduct **3ba**, formed by reaction of complex **1b** and hydrosilane **2a**.

**Scheme 4:**
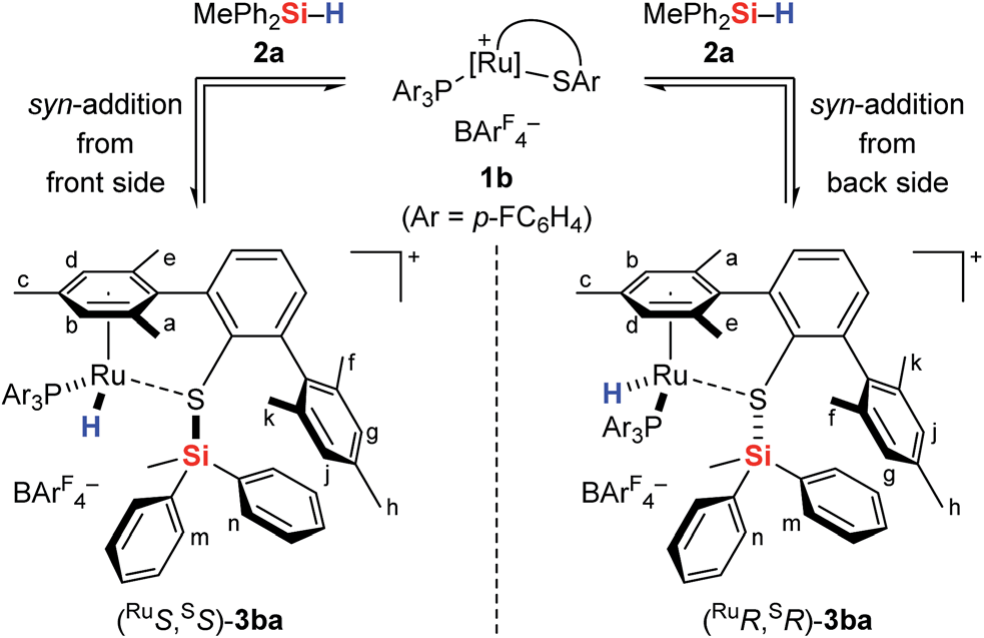
Reversible *syn*-addition of the Si–H bond across the Ru–S bond.

The analysis of the NMR spectroscopic data, including further 2D NMR measurements (see the ESI† for details), clearly supports the structural assignment of adduct **3ba**. Evidence for the intact Ru–S bond is provided by a ^3^*J*_C,H_ coupling in the ^1^H, ^13^C HMBC NMR spectrum between the quaternary sulfur-substituted carbon atom of the SDmp ligand and the ruthenium hydride. However, unambiguous information about the existence of an S–Si linkage and the reversibility of the Si–H bond activation step was still elusive. A ^1^H, ^1^H EXSY NMR then shed light on this problem: cross peaks revealed chemical exchange between the hydrides of Ru–H and MePh_2_Si–H on the NMR time scale (Fig. 2, upper); further exchange effects were found in the alkyl and aryl range (Fig. 2, lower). The two phenyl groups at the silicon atom are chemically inequivalent, showing chemical exchange between each other and the phenyl group of the free hydrosilane ([m,n,Ph_2_Si]). Surprisingly, chemical exchange is also observed for the “half-sites” of the SDmp ligand. These findings can be rationalized by cooperative Si–H bond activation. Heterolytic splitting of the Si–H bond across the polar Ru–S bond not only generates a stereogenic metal center but also stereogenicity at the sulfur atom. By reversible *syn*-addition from either side (front or back), the resulting enantiomers of **3ba** are in equilibrium, and this is exactly what is observed as chemical exchange between the diastereotopic protons of [a,e], [b,d], [f,k], and [g,j] (Scheme 4). The *syn*-selectivity of the activation step was secured by the absence of any detectable diastereomers of **3ba**.

### The role of the phosphine ligand and the substitution pattern at the silicon atom

Having elucidated a cooperative hydrosilane activation mode, we next examined the reaction of hydrosilanes **2a–e** with complexes **1a** and **1b**. In all cases, successful Si–H bond heterolysis was monitored by NMR spectroscopy. The characteristic ^1^H, ^29^Si, and ^31^P NMR chemical shifts of the various hydrosilane adducts **3** as well as the corresponding free hydrosilanes **2** and thiolate complexes **1** are collected in Table 1. While the resonance signals for mixtures of ruthenium complex **1a**, decorated with a Et_3_P ligand, and hydrosilanes **2a** and **2b** were too broad to be observed at 300 K, better resolved NMR resonances are obtained at 250 K (entries 1 and 2). The ^1^H NMR spectra of adducts **3** each feature two signature resonances, one corresponding to the Si–H group of excess hydrosilane **2** (3.7–4.8 ppm) and one upfield-shifted doublet for the ruthenium hydride either at around –8.2 (for **3aa–3ad**, entries 1–4) or –7.6 ppm (for **3ba–3bc** and **3be**, entries 5–8). The ^29^Si NMR resonances of adducts **3** (18.2–41.6 ppm) are shifted to higher frequencies relative to **2** (–17.5 to 0.6 ppm), indicating decreased electron density at the silicon atom.^17^ Although this is an oversimplified argument (for instance, a rather poor correlation, if any, between ^29^Si NMR chemical shifts and atomic charges on the silicon atom in ferrocene-stabilized silylium ions was found in our previous study^20^), the more electrophilic nature of the silicon atom in hydrosilane adducts **3** as compared to the starting hydrosilanes **2** is also evident from NPA charge analysis (*cf.* Table S4 in the ESI†). As expected, the ^29^Si NMR chemical shifts are sensitive towards the substituents at the silicon atom, and the highest downfield shifts of **3** are observed for trialkylsilanes **2c** and **2d** (entries 3, 4, and 7). The ^31^P NMR chemical shifts are static again, and a resonance at 40 ppm is characteristic for **3aa–3ad** (entries 1–4), whereas a chemical shift at 48 ppm is indicative for **3ba–3bc** and **3be** (entries 5–8). The overall trends in the NMR spectroscopic data reveal that the phosphine ligand mainly influences the electronic nature of the ruthenium hydride while the Lewis acidity of the silicon electrophile is largely controlled by the substitution pattern at the silicon atom.

### Mechanistic control experiments with deuterium-labeled and silicon-stereogenic hydrosilanes

**Scheme 5:**
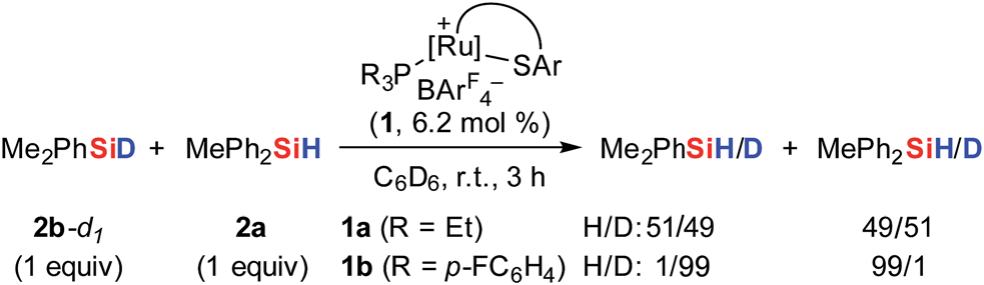
^1^H/^2^H scrambling experiments: the crucial role of the phosphine ligand.

To further probe the Si–H bond activation step, control experiments employing deuterium-labeled MePh_2_SiD (**2a**-d_1_) were performed. As expected, reaction of ruthenium(ii) thiolate complex **1b** with two equivalents of deuterosilane **2a**-d_1_ in CD_2_Cl_2_ at room temperature resulted in complete incorporation of deuterium into adduct **3ba**-d_1_ (not shown). In ^1^H/^2^H scrambling experiments, deuterium-labeled Me_2_PhSiD (**2b**-d_1_) was treated with non-deuterated MePh_2_SiH (**2a**) in the presence of catalytic amounts of either complex **1a** or **1b** at room temperature (Scheme 5). Whereas ^1^H/^2^H exchange was fast and complete with ruthenium(ii) thiolate complex **1a**, no scrambling was observed with **1b** even after 3 h.

**Scheme 6:**
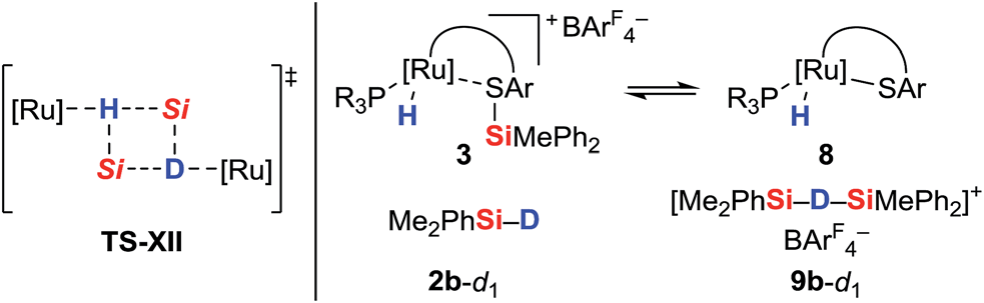
Possible pathways for ^1^H/^2^H exchange.

The pronounced differences in reactivity of both complexes led us to investigate the role of the phosphine ligand (Et_3_P in **1a***versus* (*p*-FC_6_H_4_)_3_P in **1b**) in more detail. Since electron-rich Et_3_P is a stronger σ-donor than the electron-deficient *para*-fluorinated aryl phosphine, the ruthenium hydride in hydrosilane adduct **3a** is expected to be a better hydride donor than in **3b**. On the other hand, **3b** is likely to be a better R_3_Si^+^-transfer reagent compared to **3a**. Both of these properties will contribute to the ^1^H/^2^H exchange, depending on whether this process proceeds through a σ-bond metathesis^6^ solely involving the metal hydride (**TS-XII**, Scheme 6, left)^21^ or through formation of hydronium ions **9** ([Si–H–Si]^+^)^22^ by R_3_Si^+^-transfer to a free hydrosilane (Scheme 6, right).

**Scheme 7:**
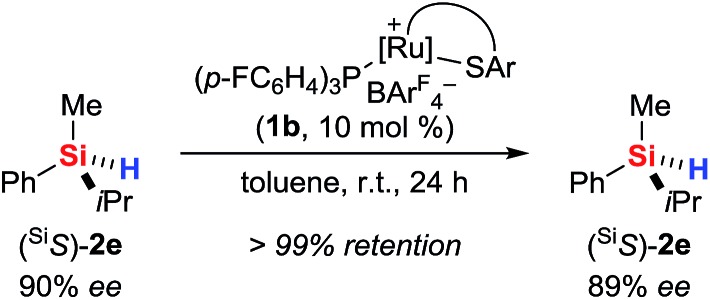
Control experiment with a silicon-stereogenic hydrosilane: no support for the intermediacy of hydronium ions.

The use of a silicon-stereogenic hydrosilane as a stereochemical probe finally allowed to distinguish between these two mechanisms: enantioenriched hydrosilane (^Si^*S*)-**2e** was reisolated with complete retention of configuration at the silicon atom after treatment with complex **1b** (Scheme 7). On the basis of this result, the generation of hydronium ions is highly unlikely, as this pathway would result in racemization. The better hydride donor strength of **3a** thus is likely to account for the ^1^H/^2^H exchange.

### Isolation and crystallographic characterization of hydrosilane adducts **3ab** and **3ad**

**Fig. 3:**
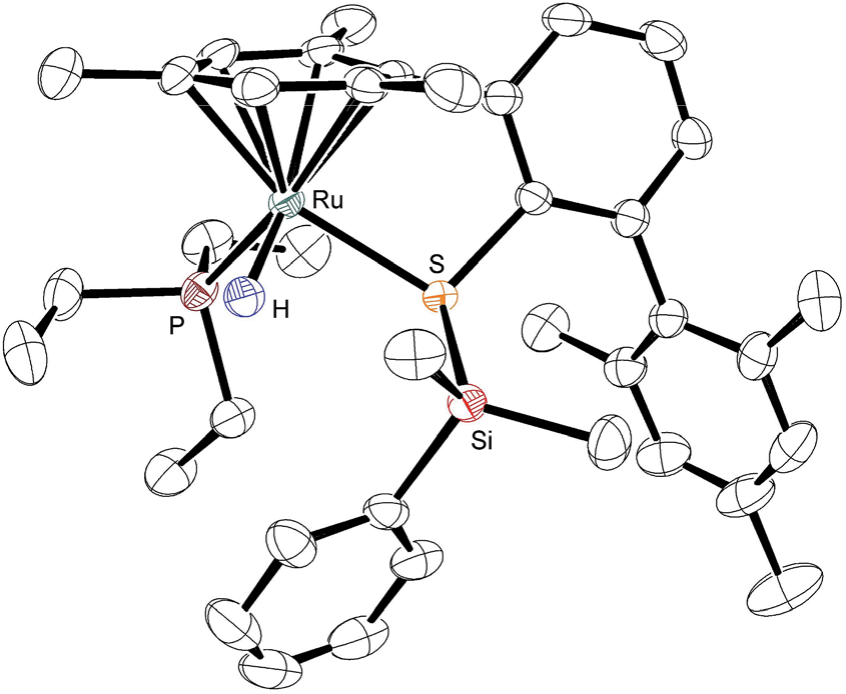
ORTEP diagram of the molecular structure of **3ab**. Thermal ellipsoids are shown at the 50% possibility level. Hydrogen atoms, except for the ruthenium hydride, and the counteranion are omitted for clarity. Selected interatomic distances (Å): Si···H, 3.17(4); S–Si, 2.2445(11); Ru–H, 1.58(4); Ru–S, 2.3882(10). For comparison, the DFT-optimized distances (Å) are as follows: Si···H, 3.188; S–Si, 2.255; Ru–H, 1.60; Ru–S, 2.394.

To provide unambiguous evidence for the generation of silylthioruthenium hydride intermediates, we pursued the crystallization of hydrosilane adducts **3**. Single crystals suitable for X-ray diffraction were obtained for **3ab** as well as **3ad** at –30 °C from a solution of complex **1a** in neat hydrosilane, either Me_2_PhSiH (**2b**) or EtMe_2_SiH (**2d**). Compared to the Ru–S bond in coordinatively unsaturated complex **1a** (2.21 Å), the molecular structure of **3ab** shows a slightly elongated (about 8%) yet intact Ru–S bond (2.39 Å, Fig. 3).^9^ The Si–H bond is completely broken and an interatomic Si···H distance of 3.17 Å indicates no interaction of the silicon with the hydrogen atom. While the hydride is bound to the ruthenium center with a bond length of 1.58 Å, the silicon atom is connected to the sulfur atom with a distance of 2.24 Å. This S–Si distance is slightly elongated (about 6–7%) compared to structurally related neutral ruthenium(ii) silylthiolate complexes (2.11 Å (ref. 23*a*) and 2.09 Å (ref. 23*b*)). The average C–Si–C angle of 110.5° reflects a tetrahedral (silylated sulfonium ion) rather than a trigonal planar (sulfur-stabilized silicon cation) coordination around the silicon atom. All selected bond lengths and angles of the EtMe_2_SiH adduct **3ad** are comparable to those of **3ab** and are in excellent agreement with the DFT optimized structures (see the ESI† for detailed data).

### Isolation and characterization of [R_3_POSiR′_3_]^+^[BAr^F^_4_]^–^ (**10**)

**Fig. 4:**
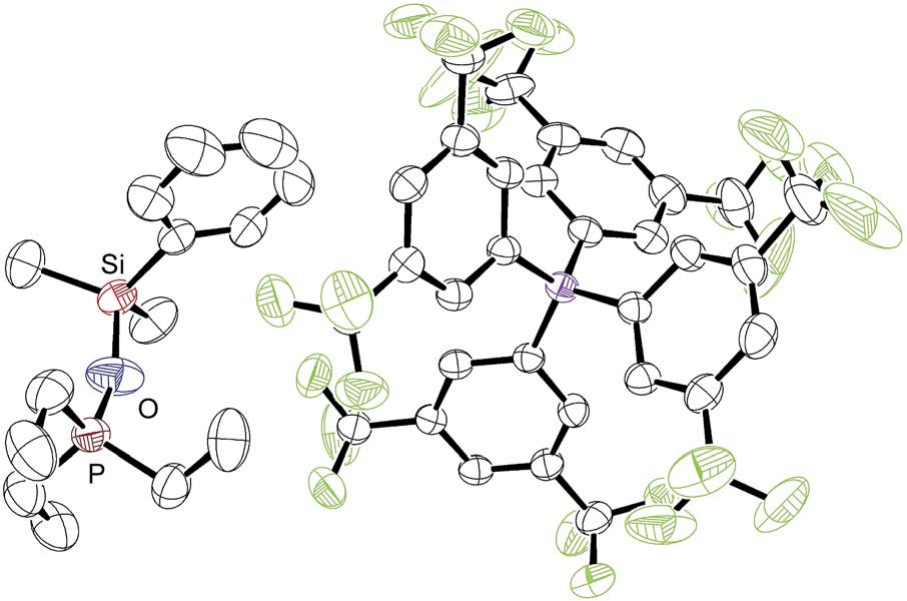
ORTEP diagram of the molecular structure of [Et_3_POSiMe_2_Ph]^+^[BAr^F^_4_]^–^ (**10ab**). Thermal ellipsoids are shown at the 50% possibility level. Hydrogen atoms are omitted for clarity. Selected bond lengths (Å): Si–O, 1.674(4); P–O, 1.529(4).

During our NMR spectroscopy studies, we noticed the presence of minor amounts of another species next to hydrosilane adduct **3** in the majority of the measurements. This observation became apparent by a second high-frequency-shifted signal in both the ^29^Si NMR and ^31^P NMR spectra. For instance, when ruthenium(ii) thiolate complex **1a** was treated with MePh_2_SiH (**2a**) to generate **3aa**, an additional minor peak was observed at 8.8 ppm in the ^29^Si NMR spectrum and at 92.9 ppm in the ^31^P NMR spectrum (later assigned to **10aa**; for a tabulated summary of all combinations of **1** and **2** to form **10**, see Table S1 in the ESI†). While the structure determination solely on the basis of NMR spectroscopy failed, the calculated mass for [(*p*-FC_6_H_4_)_3_POSiMePh_2_]^+^ (**10ba**^+^) was found by ESI mass spectrometry. We were then able to crystallize the related silyloxyphosphonium salt [Et_3_POSiMe_2_Ph]^+^[BAr^F^_4_]^–^ (**10ab**), providing conclusive evidence for our structural assignment (Fig. 4). Single crystals of **10ab** suitable for X-ray diffraction were obtained from a solution of ruthenium(ii) thiolate complex **1a** and excess hydrosilane **1b** in toluene layered by *n*-hexane at –30 °C.

**Scheme 8:**
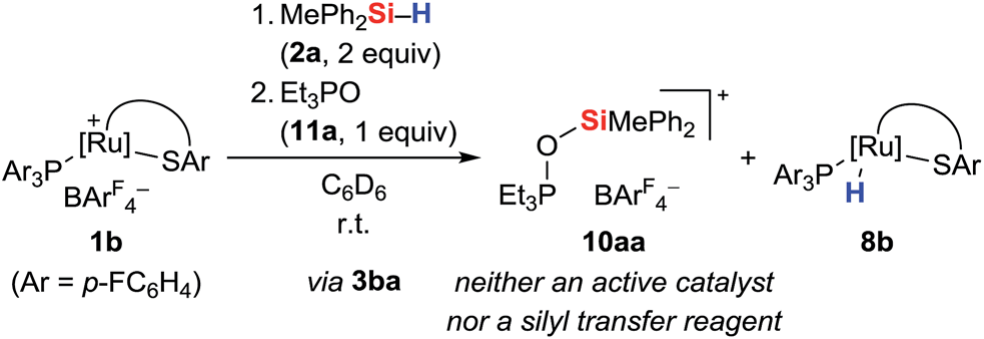
Independent preparation of [Et_3_POSiMePh_2_]^+^[BAr^F^_4_]^–^ (**10aa**).

To further probe the structure and reactivity of the silyloxyphosphonium salts, we independently prepared [Et_3_POSiMePh_2_]^+^[BAr^F^_4_]^–^ (**10aa**) by treatment of *in situ* generated silylthioruthenium hydride intermediate **3ba** with stoichiometric amounts of Et_3_PO (**11a**) in C_6_D_6_ (Scheme 8). Beside the expected chemical shift for ruthenium hydride complex **8b** at 53.5 ppm, a resonance signal at 91.3 ppm was seen in the ^31^P NMR spectrum, matching with the observed side product found in the above-described preparation of hydrosilane adduct **3aa** (*cf.* Table S1in the ESI,† entry 1).

To test the ability of the silyloxyphosphonium salts to act as silyl transfer reagents, acetophenone was added to the independently prepared mixture of **10aa** and **8b**. No reaction was observed even at prolonged reaction times, thereby ruling out **10aa** as an active species in our catalyses.14*b* The source of oxygen, however, remains unclear but traces of dioxygen cannot be fully excluded given the high reactivity of intermediate **3**. Water is unlikely since this would result in immediate silanol or disiloxane formation, and neither various equivalents of the hydrosilane nor a change of the solvent had an effect on the proportion of **10**.

### DFT calculations

**Fig. 5:**
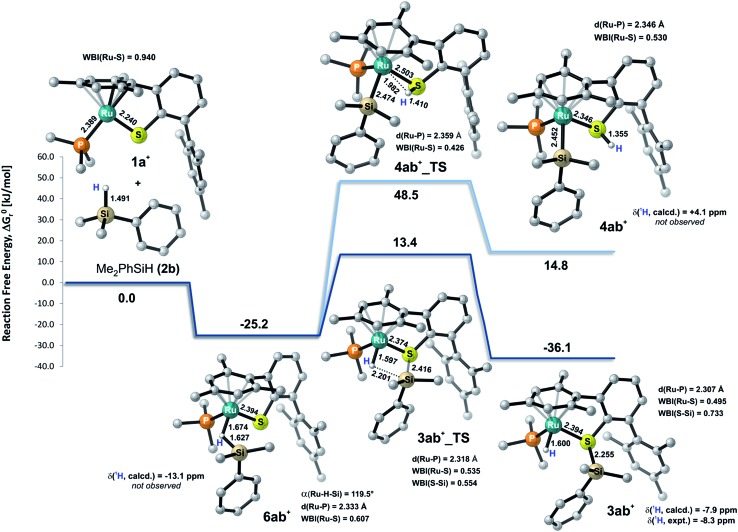
Calculated relative free energies Δ*G*0r together with the structure of relevant intermediates and transition states for the Si–H bond activation of Me_2_PhSiH (**2b**) by ruthenium(ii) thiolate complex **1a**^+^ (results obtained at the B3LYP-D3(BJ)/ECP/6-31+G** level of theory using an SMD solvation model). All C–H hydrogen atoms are omitted for clarity. Bond lengths are in Å. Computed and experimental ^1^H NMR shifts of intermediates are reported in ppm (with respect to TMS). Wiberg bond indices (WBI) of selected bonds are also indicated.

To gain deeper insight into the nature of the Si–H bond activation step and to identify possible intermediates, quantum-chemical calculations at the B3LYP-D3(BJ)/ECP/6-31+G(d,p) level including an atom-pairwise correction of Grimme's D3 model with Becke–Johnson (BJ) damping for dispersion forces and using an SMD solvation model to account for bulk solvent effects (benzene as a solvent) were performed (*cf.* computational details in the ESI†). Combination of various triaryl- and trialkylphosphine ligands and hydrosilanes [MePh_2_SiH (**2a**), Me_2_PhSiH (**2b**), EtMe_2_SiH (**2d**), *t*BuMe_2_SiH] were examined computationally. The free-energy profile together with the optimized structures of relevant intermediates and transition states for the reaction of ruthenium(ii) thiolate complex **1a**^+^ with Me_2_PhSiH (**2b**) is shown in Fig. 5 (selected structural parameters and thermodynamic data for the entire series of complexes studied in this work are collected in Table S3 in the ESI†).

**Fig. 6:**
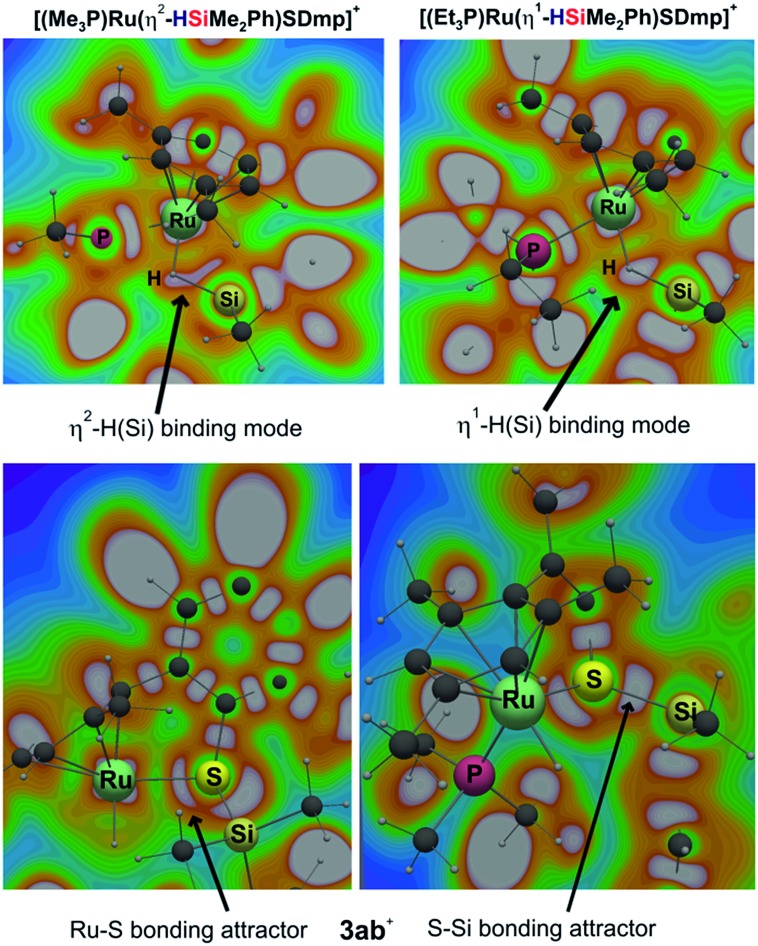
Cut-plane plots from ELI-D analyses of bonding in hydrosilane complexes **6** of [(R_3_P)Ru(SDmp)]^+^ (R_3_P = Me_3_P, upper left and R_3_P = Et_3_P, upper right) with Me_2_PhSiH (**2b**), and in silylthioruthenium hydride intermediate **3ab**^+^ (lower). The gray-white regions represent ELI-D maxima (bonding attractors).

Cooperative heterolysis of the Si–H bond commences with coordination of the hydrosilane to the unsaturated 16-electron [(R_3_P)Ru(SDmp)]^+^ complex **1**^+^, filling the ruthenium(ii) vacant coordination site by forming a stable η^1^- or η^2^-hydrosilane complex (*cf.***6** or **6′** in Chart 1). The coordination of the hydrosilane to ruthenium(ii) thiolate complex **1**^+^ was revealed as barrierless on the electronic energy surface, albeit the counteranion might affect the reaction barrier when ion-pair dissociation/formation takes place. The reaction is in most cases exergonic, with exception of the hydrosilane decorated with a bulky *tert*-butyl group, where the Δ*G*0r is slightly positive (*cf.* Table S3 in the ESI†). The bonding situation in the hydrosilane adducts **6**^+^ depends on the nature of the phosphine ligand as well as on the hydrosilane (*cf.*Fig. 6). For instance, the sterically least demanding complex bearing a Me_3_P ligand prefers side-on η^2^ coordination of Me_2_PhSiH (**2b**) with a somewhat more elongated Si–H bond (1.794 Å) and a more acute *α*(Ru···H···Si) angle (99°) as compared to the η^1^-hydrosilane complex of **1a**^+^ (with Et_3_P instead of Me_3_P) and Me_2_PhSiH (**2b**), featuring a Si–H bond distance of 1.627 Å and *α*(Ru···H···Si) angle of 120°. While the end-on η^1^-hydrosilane coordination appears to be driven mainly by steric hindrance (changing the electron-donating/-withdrawing substituents in the Ar_3_P ligand does not have any significant influence on the structural preference), the preference for a η^2^-H(Si) binding mode may be recovered by introducing a second phenyl group at the silicon atom (*cf.* Si–H bond distances in Table S3†). In spite of some structural and NMR spectroscopic differences between the η^1^- and η^2^-hydrosilane complexes (*cf.* Tables S2 and S3 in the ESI† for computed properties), the Δ*G*0r of their formation are very similar (*cf.* Table S3 in the ESI†), indicating a rather weak stabilization energy due to Ru–Si interaction in the side-on adduct. These observations are fully consistent with some of the previous findings of Brookhart and co-workers on iridium(iii) hydrosilane complexes.^24^

In analogy to our previous study on iridium pincer complexes,^25^ we attempted to detect any intermediate experimentally by using NMR spectroscopy at low temperatures (note that the hydrosilane complexes **6**^+^ and **6′**^+^ are predicted to display a characteristic ^1^H hydride resonance shifted to lower frequencies by *ca.* –3 to –5 ppm as compared to those of the observed adducts **3**; *cf.*Fig. 5 and Table S2 in the ESI†). Those attempts were, however, not successful, most likely due to the high reactivity of these species (*vide infra*). Irrespective of the hydrosilane binding mode (η^1^ or η^2^), the Ru–S bond length in these complexes is much longer (2.39–2.40 Å) than in the starting complex **1**^+^ (∼2.24 Å; *cf.* Table S3 in the ESI†). Nevertheless, various bonding analyses including Wiberg bond indices (WBI, *cf.* Table S4 in the ESI†) and the electron localizability indicator (ELI-D; *cf.*Fig. 6, lower left) show the remaining partial Ru–S bonding character.

While a classical oxidative addition pathway (*cf.***7** in Chart 1) could not be located by the calculations, two different pathways for the heterolytic Si–H bond cleavage based on hydrosilane complexes **6**^+^ or **6′**^+^ have been investigated. These include either migration of the silyl group or the hydrogen atom to the sulfur atom of the SDmp ligand. The hydrogen transfer has been found to be energetically disfavored by more than 30 kJ mol^–1^ in both the transition state energy and in the free energy of the products when compared to the transfer of a silylium ion. This coincides with the experimental observations that a ruthenium silyl complex (*cf.***4** in Chart 1) could not be detected by NMR spectroscopy (for predicted NMR shifts of these species, see Fig. 5 and Table S2 in the ESI†). The silyl transfer proceeds *via* a concerted four-membered transition state **3_TS** with a remarkably weakened (broken) Si···H bond (∼2.15–2.25 Å) and a nearly completed S–Si bond (∼2.41–2.46 Å, Wiberg bond index ∼0.55; *cf.* Tables S3 and S4 in the ESI†). The transition states are computed to lie at *ca.* 10–75 kJ mol^–1^ relative to the reactants. In general, ruthenium(ii) thiolate complexes that are decorated with a trialkylphosphine instead of a triarylphosphine ligand are found to have a lower reaction barrier by about 14–20 kJ mol^–1^. The highest barrier (56–75 kJ mol^–1^) is computed for reactions with *t*BuMe_2_SiH, which is by >20 kJ mol^–1^ higher compared to reactions using hydrosilanes such as Me_2_PhSiH (**2b**) and EtMe_2_SiH (**2d**). The overall Si–H bond activation process resulting in hydrosilane adducts **3**^+^ is exergonic with Δ*G*0r ranging from *ca.* –20 to –40 kJ mol^–1^. Nevertheless, the higher reaction barrier in the case of *t*BuMe_2_SiH would explain why hydrosilanes decorated with bulky groups do not react with ruthenium(ii) thiolate complexes **1** although the overall free reaction energy is negative. Notably, the catalytically active cation in **1** may also form ion pairs with the sterically demanding BAr^F^_4_^–^ counterion (particularly in organic solvents with low polarity), and that could additionally hamper the interaction of **1** with bulky substrates.

**Fig. 7:**
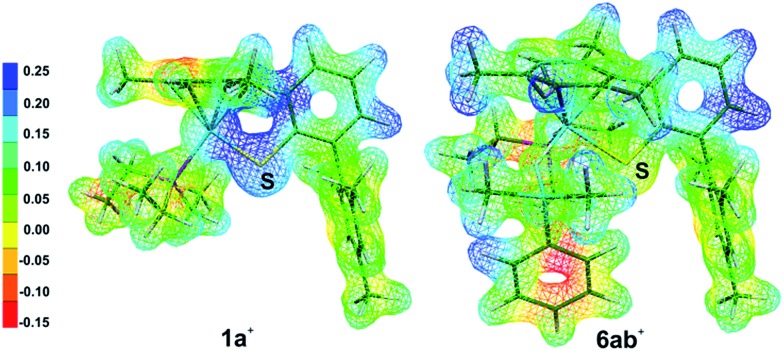
Molecular electrostatic potentials (MEP) of [(Et_3_P)Ru(SDmp)]^+^ (**1a**^+^) and [(Et_3_P)Ru(SDmp)·Me_2_PhSiH]^+^ (**6ab**^+^). Isovalue surfaces are displayed at an electron density of 0.04 a.u. (B3LYP/ECP/6-31+G** results). Red and blue regions correspond to the extreme negative and positive potentials, respectively.

During the formation of complexes **6**^+^ or **6′**^+^, the Ru–S bond remains virtually intact and also preserves some bonding character in the hydrosilane adducts **3**^+^. Attempts to locate a possible intermediate corresponding to the activation of the hydrosilane through coordination to the Lewis basic sulfur atom failed (*cf.***5** in Chart 1). This may be ascribed to a partial positive charge located on the sulfur atom in complex **1**^+^, reducing its electron-donating ability. Upon binding of the hydrosilane, the positive charge at the sulfur atom diminishes, which enhances its Lewis basic properties (*cf.*Fig. 7 for MEP and Table S4 in the ESI†).

## Conclusions

In the light of the reactivity of ruthenium(ii) thiolate complexes of type [(R_3_P)Ru(SDmp)]^+^[BAr^F^_4_]^–^ (**1**) in the activation of E–H bonds, *e.g.* H–H,^9^ Si–H,^14^ and B–H^15^ bonds, detailed mechanistic insight into the hydrosilane activation mode have been disclosed. A combined experimental, spectroscopic, crystallographic, and theoretical investigation provided conclusive evidence for a heterolytic Si–H bond activation pathway involving metal–ligand cooperativity.^5^ The principal results are summarized as follows:

(1) The quantum-chemical analyses reveal that the hydrosilane is initially activated by coordination of the Si–H bond to the Lewis acidic metal center^26^ rather than Lewis base activation through the Lewis basic thiolate ligand, as often proposed for metal–alkoxide and –oxo complexes (*cf.***6** or **6′***vs.***5** in Chart 1). In accordance with previous studies,^24^ the exact coordination mode, either η^1^ (end-on) or η^2^ (side-on), is driven by the steric demand of the phosphine ligand as well as the substituents at the silicon atom. While the η^1^-complexes can be considered as a more potent source of electrophilic silicon, the energy differences are very small, indicating a rather weak stabilization energy due to ruthenium to σ*(Si–H) backbonding. Both coordination modes are barrierless on the electronic energy surface and could not be detected spectroscopically.

(2) After electrophilic activation, the Si–H bond is heterolytically split at the polar Ru–S bond *via* a concerted four-membered transition state (*cf.***3ab^+^_TS** in Fig. 5). The σ-bond metathesis results in formation of a cationic silylthioruthenium hydride intermediate (*cf.***3** in Chart 1), combining a ruthenium(ii) hydride and a sulfur-stabilized silicon cation, *i.e.* metallasilylsulfonium ion, in one molecule.^12^

(3) Addition of the Si–H bond across the Ru–S bond with reversed regioselectivity, affording a ruthenium silyl complex with a ligated thiol (*cf.***4***vs.***3** in Chart 1), is energetically disfavored by more than 30 kJ mol^–1^.

(4) The Ru–S bond remains virtually intact during the Si–H bond activation event, even preserving bonding character in the hydrosilane adducts (Wiberg bond indices around 0.5).

(5) The overall Si–H bond activation process is reversible and exergonic with Δ*G*0r ranging from –20 to –40 kJ mol^–1^, proceeding instantly already at low temperatures.

(6) The spectroscopic and computational characterization of the stable yet reactive cationic silylthioruthenium hydride intermediates is reported. Unambiguous proof for the structural assignment was provided by the successful isolation and crystallographic characterization of these catalytically active key intermediates. The computational data are in full accordance with the experimental findings.

(7) The regioselective and stereospecific *syn*-addition of the Si–H bond was further verified with the aid of deuterium labeling and a silicon-stereogenic hydrosilane as a stereochemical probe.

(8) The analysis of the NMR spectra indicated the presence of a side product, which could be identified crystallographically as silyloxyphosphonium salt [R_3_POSiR′_3_]^+^[BAr^F^_4_]^–^. Even though its formation remains unclear, these species have been shown to be catalytically inactive.

Overall, the mechanistic details of the Si–H bond activation at polar Ru–S bonds have been clarified. Compared to nature, where a polar Ni–S bond potentially serves as a reactive site for heterolytic dihydrogen splitting in [NiFe] hydrogenases,^27^ the present study of the closely related hydrosilane activation represents, next to Stradiotto's seminal report,^12^ a rare example of a fully-understood system of heterolytic bond splitting mediated by a transition metal thiolate complex and might provide a solid foundation for the understanding of the basic mechanistic principles of both processes.

## Supplementary Material

Supplementary information

Crystal structure data
