# Supplementary material for: Mechanism of the cooperative Si–H bond activation at Ru–S bonds
Source: Chem Sci. 2015 May 18;6(7):4324–34. doi: 10.1039/c5sc01035g (PMC5707498; doi:10.1039/c5sc01035g)
Supplement: Supplementary file 1 [file SC-006-C5SC01035G-s001.pdf]

Electronic Supplementary Material (ESI) for Chemical Science.
This journal is © The Royal Society of Chemistry 2015

Mechanism of the cooperative Si–H bond activation at Ru–S bonds

Timo Stahl,[a] Peter Hrobárik,*[a] C. David F. Königs,[ab] Yasuhiro Ohki,[b] Kazuyuki Tatsumi,[b] Sebastian Kemper,[a] Martin Kaupp,[a] Hendrik F. T. Klare*[a] and Martin Oestreich*[a]

[a] Institut für Chemie, Technische Universität Berlin,
Straße des 17. Juni 115, 10623 Berlin, Germany
peter.hrobarik@tu-berlin.de
hendrik.klare@tu-berlin.de
martin.oestreich@tu-berlin.de

[b] Department of Chemistry, Graduate School of Science and Research Center for Materials Science,
Nagoya University, Furo-cho, Chikusa-ku, Nagoya 464-8602, Japan

Electronic Supplementary Information

Table of Contents

**1**	**General Information**	**S3**
1.1	Experimental Details	S3
1.2	Computational Details	S4
**2**	**Experimental Details and NMR Spectroscopic Data**	**S6**
2.1	Si–H Bond Activation of Hydrosilanes **2a**–**e** with Ruthenium Thiolate Complexes $[(R_3P)Ru(SDmp)]^+[BAr^F_4]^-$ (**1a**: $R_3P = Et_3P$; **1b**: $R_3P = (p\text{-}FC_6H_4)_3P$)	S6
2.2	Characterization of Side Products $[R_3POSiR'_3]^+[BAr^F_4]^-$ (**8**)	S16
2.3	Independent Preparation of $[Et_3POSiMePh_2]^+[BAr^F_4]^-$ (**10aa**) by Addition of Et_3PO (**11a**) to In-Situ Generated Adduct **3ba**	S16
2.4	Control Experiment: Probing the Reactivity of $[Et_3POSiMePh_2]^+[BAr^F_4]^-$ (**10aa**)	S17
2.5	Preparation of Silyl Thioether $Ph_2MeSiSDmp$	S18
**3**	**Mechanistic Control Experiment with a Silicon-Stereogenic Hydrosilane**	**S19**
3.1	Racemization Experiment of Enantioenriched Hydrosilane (^{Si}S)-**2e** with Ruthenium Thiolate Complex **1b**	S19
**4**	**Mechanistic Control Experiments with a Deuterium-Labeled Hydrosilane**	**S19**
4.1	Si–H Bond Activation of Deuterium-Labeled Dimethylphenylsilane (**2b**-d_1) with Ruthenium(II) Thiolate Complex **1b**	S19

4.2	^{2}H-Scrambling Experiment of Deuterium-Labeled Dimethylphenylsilane (**2b**-*d*$_1$) and Non-Deuterated Methyldiphenylsilane (**2a**) with Ruthenium(II) Thiolate Complex **1a**	S20
4.3	^{2}H-Scrambling Experiment of Deuterium-Labeled Dimethylphenylsilane (**2b**-*d*$_1$) and Non-Deuterated Methyldiphenylsilane (**2a**) with Ruthenium(II) Thiolate Complex **1b**	S20
**5**	**DFT Calculations**	**S21**
**6**	**NMR Spectra**	**S29**
6.1	NMR Spectra of Hydrosilane Adducts **3aa**–**3ad**, **3ba**–**3bc**, and **3be**	S29
6.2	NMR Spectra of $[Et_3POSiMePh_2]^+[BAr^F{}_4]^-$ (**10aa**)	S85
**7**	**Crystallographic Data**	**S89**
7.1	Molecular Structure of **3ab**	S90
7.2	Molecular Structure of **3ad**	S91
7.3	Molecular Structure of **10ab**	S92
**8**	**Optimized Cartesian Coordinates of Selected Ruthenium(II)–Thiolate Complexes**	**S93**
**9**	**References**	**S124**

1 General Information

1.1 Experimental Details

All reactions were performed in flame-dried glassware using an *MBraun* glove box (O_2 < 0.5 ppm, H_2O < 0.5 ppm) or conventional Schlenk techniques under a static pressure of argon or nitrogen. Liquids and solutions were transferred with syringes. Solvents (THF, toluene, *n*-hexane, and CH_2Cl_2) were purified and dried following standard procedures. C_6D_6 (purchased from *Eurisotop*, dried over CaH_2, distilled, and degassed prior to use), CD_2Cl_2 (purchased from *Eurisotop*, dried over $CaCO_3$, distilled, and degassed prior to use), and toluene-d_8 (purchased from *Eurisotop* in sealed glass ampoules and used as received) were stored under an inert atmosphere. Hydrosilanes $MePh_2SiH$ (**2a**), Me_2PhSiH (**2b**), Et_3SiH (**2c**), and $EtMe_2SiH$ (**2d**) were obtained from commercial sources, distilled, and degassed prior to use. Ruthenium(II) thiolate complexes $[(Et_3P)Ru(SDmp)]^+[BAr^F{}_4]^-$ (**1a**)[S1] and $[\{(p\text{-}FC_6H_4)_3P\}Ru(SDmp)]^+[BAr^F{}_4]^-$ (**2b**)[S2], as well as an enantioenriched sample of silicon-stereogenic hydrosilane *i*PrMePhSiH (**2e**)[S3] were prepared according to reported procedures [SDmp = 2,6-bis(2,4,6-trimethylphenyl)phenyl-thiolate, Ar^F = 3,5-bis(trifluoromethyl)phenyl]. ^1H, ^{11}B, $^{19}F\{^1H\}$, and $^{31}P\{^1H\}$ spectra as well as 2D NMR data sets ($^1H,^1H$ COSY, $^1H,^1H$ EXSY, $^1H,^{13}C$ HSQC, $^1H,^{13}C$ HMBC, $^1H,^{19}F$ HMQC, $^1H,^{29}Si$ HMQC, $^1H,^{31}P$ HMQC) were recorded in C_6D_6 or CD_2Cl_2 on *Bruker* AV500 instruments. Chemical shifts are reported in parts per million (ppm) and are referenced to the residual solvent resonance in the ^1H NMR spectra as the internal standard (C_6D_5H: δ = 7.16 ppm for ^1H NMR; $CDHCl_2$: δ = 5.32 ppm for ^1H NMR). All other nuclei (^{11}B, ^{13}C, ^{19}F, ^{29}Si, and ^{31}P) are referenced in compliance with the unified scale for NMR chemical shifts as recommended by the IUPAC.[S4] Data are reported as follows: chemical shift, multiplicity (s = singlet, d = doublet, t = triplet, q = quartet, m = multiplet, m_c = centrosymmetric mulitplet), coupling constants (Hz) and integration. In some NMR experiments, the resonance signals of the desired compound overlap with chemical shifts derived from other molecules (e.g., solvent or hydrosilane). In these cases, the expected integration is reported and marked with a star (*). The assignment of the resonance signals was verified by 2D NMR spectroscopy. Infrared (IR) spectra were recorded on an *Agilent Technologies* Cary 630 FT-IR spectrophotometer equipped with an ATR unit and are reported in wavenumbers (cm^{-1}). Enantiomeric excesses were determined by analytical high pressure liquid chromatography (HPLC) analysis on an *Agilent Technologies* 1290 Infinity instrument with a chiral stationary phase using a *Daicel Chiralcel* OJ-RH column ($MeCN/H_2O$ mixtures as solvent). High-resolution mass spectrometry (HRMS) and elemental analysis were performed by the Analytical Facility of the Institut für Chemie, Technische Universität Berlin.

1.2 Computational Details

All structures (without adding counteranions) were fully optimized at the B3LYP level of theory,[S5] including an atom-pairwise correction for dispersion forces via Grimme's D3 model[S6] with Becke-Johnson (BJ) damping[S7] in the Gaussian 09 program package.[S8] A quasirelativistic energy-consistent small-core pseudopotential (effective-core potential, ECP)[S9] in conjunction with a (8s7p6d)/[6s5p3d] GTO valence basis set was used for the ruthenium atom, whereas ligand atoms (C, H, O, F, Si, P) have been treated with an all-electron 6-31+G(d,p) basis set. To simulate solvent effects and to obtain the relative solvation free energies, the SMD solvation model (a reaction field calculation using the integral equation formalism for the polarizable continuum model, IEF-PCM, with radii and non-electrostatic terms from Truhlar and co-workers)[S10] was used as implemented in Gaussian 09. Benzene (ε_r = 2.27) was considered as the solvent. Harmonic vibrational frequency calculations at the same level of theory were performed to verify all stationary points as minima (no imaginary frequency) or transition states (one imaginary frequency), as well as to provide free energies at 298.15 K. The final Gibbs free energies (G^0) reported here are based on SCF energies with Gibbs free energy corrections (at 298.15 K), solvation corrections, and corrections for dispersion effects using Grimme's D3(BJ) method.

Natural population analysis (NPA) atomic charges[S11] and Wiberg bond indices (WBI)[S12] were evaluated using the built-in NBO subroutines of Gaussian.[S13] The Kohn–Sham wave functions were also analyzed by means of the electron localizability indicator (ELI-D)[S14] in the DGrid program[S15], and the results were visualized by using the ParaView program.[S16]

Fully relativistic density functional (DFT) calculations of NMR nuclear shieldings have been carried out at the matrix Dirac–Kohn–Sham (mDKS) level with the ReSpect-MAG code, including a new four-component module.[S17] This method combines the concept of gauge including atomic orbitals (GIAOs) with restricted magnetically balanced (RMB) orbitals for the small component. Details of the mDKS-RMB-GIAO method are given in refs. [S18] and [S19]. The four-component mDKS calculations have been done at the generalized-gradient-approximation level (GGA) with the Perdew–Burke–Ernzerhof (PBE) exchange-correlation functional,[S20] which was evaluated numerically on an adaptive molecular grid (program default). Dyall's all-electron valence double-ζ (VDZ) basis set of 21s14p10d2f quality was used for the metal center.[S21] For ligand atoms we have employed fully uncontracted Huzinaga–Kutzelnigg-type IGLO-II basis sets.[S22] In comparison to our previous study,[S19] no fitting (resolution-of-identity) of the total electron density and of the components of the spin density was applied. All relativistic calculations were done

with a finite-size nucleus model employing a Gaussian charge distribution. The computed ^{1}H and ^{31}P nuclear shieldings were converted to chemical shifts (δ, in ppm) relative to the shielding of tetramethylsilane (TMS) and 85% aq. H_3PO_4, respectively, using $[H_2Ru(CO)_4]$ with δ(^{1}H)= –7.9 ppm[S23] and $[(Et_3P)Ru(SDmp)]^+$ (**1a**$^+$) with δ(^{31}P)= +23.0 ppm[S24] as secondary standards. The computed ^{29}Si NMR shieldings were converted to chemical shifts relative to the shielding of TMS, obtained at the same computational level.

2 Experimental Details and NMR Spectroscopic Data

2.1 Si–H Bond Activation of Hydrosilanes 2a–e with Ruthenium Thiolate Complexes $[(R_3P)Ru(SDmp)]^+[BAr^F_4]^-$ (1a: R_3P = Et_3P; 1b: R_3P = $(p\text{-}FC_6H_4)_3P$)

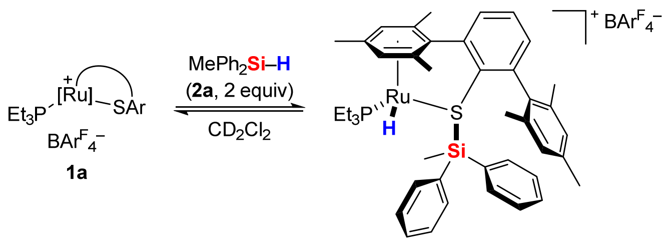

3aa
$C_{75}H_{66}BF_{24}PSSiRu$
M = 1626.3 g/mol

In an NMR tube, $MePh_2SiH$ (**2a**, 4.8 mg, 24 μmol, 2.0 equiv) was added to a solution of $[(Et_3P)Ru(SDmp)]^+[BAr^F_4]^-$ (**1a**, 17 mg, 12 μmol, 1.0 equiv) in CD_2Cl_2 (0.6 mL). The sample was shaken vigorously and directly subjected to NMR spectroscopic analysis indicating the formation of adduct **3aa** along with excess hydrosilane **2a** and small amounts of side product $[Et_3POSiMePh_2]^+[BAr^F_4]^-$ (**10aa**).

Selected NMR spectroscopic data for ***3aa**:*
^{1}H NMR (500 MHz, CD_2Cl_2, 250 K): δ = –8.24 (d, *J* = 47.5 Hz, 1H), 0.64 (s, 3H), 0.70 (dt, *J* = 15.7 Hz, *J* = 7.5 Hz, 9H*), 1.09 (qd, *J* = 7.3 Hz, *J* = 7.3 Hz, 3H), 1.45 (qd, *J* = 7.3 Hz, *J* = 7.3 Hz, 3H), 1.84 (s, 3H), 1.87 (s, 3H), 1.92 (s, 3H), 2.04 (s, 3H), 2.09 (s, 3H), 2.46 (s, 3H), 5.58 (d, *J* = 2.5 Hz, 1H), 5.96 (s, 1H), 6.00 (s, 1H), 6.80 (s, 1H), 7.05 (d, *J* = 7.0 Hz, 2H), 7.15 (d, *J* = 7.0 Hz, 2H), 7.19 (d, *J* = 8.0 Hz, 1H), 7.21 (dd, *J* = 7.0 Hz, *J* = 7.0 Hz, 2H), 7.29 (dd, *J* = 7.3 Hz, *J* = 7.3 Hz, 2H), 7.60 (s, 4H*), 7.77 (s, 8H*) ppm. **^{11}B NMR** (161 MHz, CD_2Cl_2, 250 K): δ = –6.7 ppm. **^{19}F{^{1}H} NMR** (471 MHz, CD_2Cl_2, 250 K): δ = –62.6 ppm. **^{31}P{^{1}H} NMR** (203 MHz, CD_2Cl_2, 250 K): δ = 39.8 ppm. **^{1}H,^{13}C HSQC NMR** (500/126 MHz, CD_2Cl_2, 250 K): δ = 0.64/–2.8, 0.70/6.9, 1.09/18.9, 1.45/18.9, 1.84/19.2, 1.87/18.7, 1.92/21.1, 2.04/21.1, 2.09/20.7, 2.46/19.5, 5.58/97.8, 5.96/129.0, 6.00/88.0, 6.80/128.5, 7.05/132.5, 7.15/137.1, 7.19/133.5, 7.21/128.3, 7.29/127.8, 7.60/117.5, 7.77/134.7 ppm. **^{1}H,^{13}C HMBC** (500/126 MHz, CD_2Cl_2, 250 K): δ = 1.84/85.2, 1.84/88.0, 1.84/97.8, 1.87/97.8, 1.87/101.6, 1.87/111.5, 1.92/129.0, 1.92/134.5, 1.92/135.4, 2.04/128.5, 2.04/134.5, 2.04/134.8, 2.09/128.5, 2.09/129.0, 2.09/137.8, 2.46/88.0, 2.46/93.6, 2.46/111.5, 5.58/18.7, 5.58/88.0, 5.58/111.5, 5.96/21.1, 5.96/128.5, 5.96/134.5, 6.00/19.1, 6.00/97.8, 6.00/111.5, 6.80/21.1, 6.80/129.0, 6.80/134.5 ppm. **^{1}H,^{29}Si HMQC NMR** (500/99 MHz, CD_2Cl_2, 250 K, optimized for *J* = 8 Hz): δ = 0.64/18.2, 7.05/18.2, 7.15/18.2 ppm. **^{1}H,^{31}P HMQC NMR** (500/203 MHz, CD_2Cl_2, 250 K, optimized for *J* = 7 Hz): δ = –8.24/39.8, 0.70/39.8, 1.09/39.8, 1.45/39.8, 2.46/39.8, 5.58/39.8 ppm.

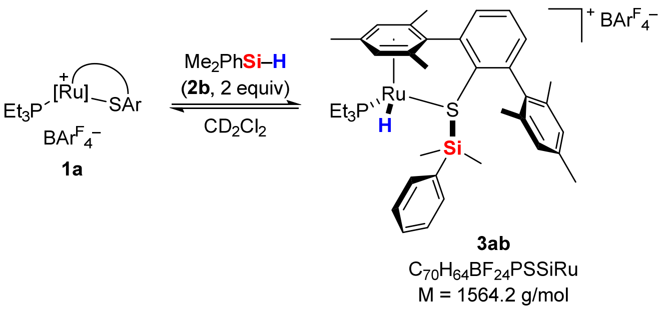

In an NMR tube, Me_2PhSiH (**2b**, 3.3 mg, 24 µmol, 2.0 equiv) was added to a solution of $[(Et_3P)Ru(SDmp)]^+[BAr^F{}_4]^-$ (**1a**, 17 mg, 12 µmol, 1.0 equiv) in CD_2Cl_2 (0.6 mL). The sample was shaken vigorously and directly subjected to NMR spectroscopic analysis indicating the formation of adduct **3ab** along with excess hydrosilane **2b** and small amounts of side product $[Et_3POSiMe_2Ph]^+[BAr^F{}_4]^-$ (**10ab**). Single crystals of **3ab** suitable for X-ray diffraction were obtained by treatment of ruthenium thiolate complex **1a** with a large excess of hydrosilane **2b**, followed by slow crystallization at –30 °C.

Selected NMR spectroscopic data for ***3ab****:*

^{1}H NMR (500 MHz, CD_2Cl_2, 250 K): δ = –8.26 (d, *J* = 48.8 Hz, 1H), 0.07 (s, 3H), 0.60 (s, 3H), 0.79 (dt, *J* = 16.1 Hz, *J* = 7.8 Hz, 9H), 1.20 (qd, *J* = 7.5 Hz, *J* = 7.5 Hz, 3H), 1.43 (qd, *J* = 7.5 Hz, *J* = 7.5 Hz, 3H), 1.87 (s, 3H), 1.94 (s, 3H), 2.07 (s, 3H), 2.18 (d, *J* = 1.3 Hz, 3H), 2.21 (s, 3H), 2.32 (s, 3H), 5.42 (d, *J* = 3.5 Hz, 1H), 6.13 (s, 1H), 6.85 (s, 1H), 7.00 (s, 1H), 7.18 (d, *J* = 7.2 Hz, 2H), 7.28–7.34 (m, 2H*), 7.37–7.45 (m, 2H*), 7.57–7.59 (m, 2H*), 7.61 (s, 4H*), 7.77 (s, 8H*) ppm. **^{11}B NMR** (161 MHz, CD_2Cl_2, 250 K): δ = –6.7 ppm. **^{19}F{^{1}H} NMR** (471 MHz, CD_2Cl_2, 250 K): δ = –62.6 ppm. **^{31}P{^{1}H} NMR** (203 MHz, CD_2Cl_2, 250 K): δ = 40.4 ppm. **^{1}H,^{13}C HSQC NMR** (500/126 MHz, CD_2Cl_2, 250 K): δ = 0.07/–0.7, 0.60/3.2, 0.79/7.1, 1.20/19.1, 1.43/19.1, 1.87/19.0, 1.94/18.6, 2.07/21.0, 2.18/19.5, 2.21/21.6, 2.32/20.7, 5.42/94.2, 6.13/91.5, 6.85/129.5, 7.00/128.9, 7.18/134.4, {7.28–7.34}/127.8, {7.28–7.34}/133.4, {7.37–7.45}/129.2, {7.37–7.45}/130.6, {7.57–7.59}/128.5, {7.57–7.59}/129.2, 7.61/117.2, 7.77/134.6 ppm. **^{1}H,^{13}C HMBC NMR** (500/126 MHz, CD_2Cl_2, 250 K): δ = 1.87/84.5, 1.87/91.4, 1.87/94.3, 1.94/94.3, 1.94/100.5, 1.94/113.0, 2.07/128.9, 2.07/134.9, 2.07/135.9, 2.18/91.4, 2.18/94.0, 2.18/113.0, 2.21/129.5, 2.21/134.9, 2.21/136.1, 2.32/128.9, 2.32/129.5, 2.32/138.2, 5.42/18.6, 5.42/91.5, 5.42/113.0, 6.13/19.5, 6.13/94.2, 6.13/113.0, 6.85/20.7, 6.85/21.6, 6.85/128.9, 6.85/134.9, 7.00/21.0, 7.00/129.5, 7.00/134.9, 7.18/130.6, {7.28–7.34}/141.5, {7.37–7.45}/134.2, {7.57–7.59}/113.0, {7.57–7.59}/139.9, {7.57–7.59}/141.5, {7.57–7.59}/144.5 ppm. **^{1}H,^{29}Si HMQC NMR** (500/99 MHz, CD_2Cl_2, 250 K, optimized for *J* = 8 Hz): δ = 0.07/28.4, 0.60/28.4, 7.18/28.4 ppm. **^{1}H,^{31}P HMQC NMR** (500/203 MHz, CD_2Cl_2, 250 K, optimized for *J* = 7 Hz): δ = –8.26/40.4, 0.79/40.4,1.20/40.4,1.43/40.4, 2.18/40.4, 5.42/40.4 ppm. **X-ray**: for X-ray data, see section 7.1.

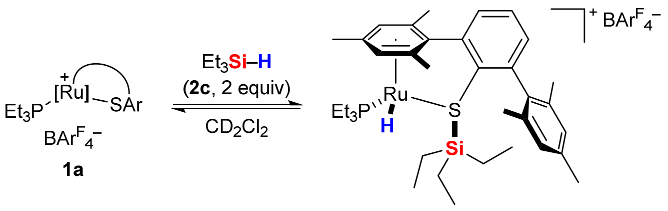

3ac
$C_{68}H_{68}BF_{24}PSSiRu$
M = 1544.3 g/mol

In an NMR tube, Et_3SiH (**2c**, 2.8 mg, 24 µmol, 2.0 equiv) was added to a solution of $[(Et_3P)Ru(SDmp)]^+[BAr^F{}_4]^-$ (**1a**, 17 mg, 12 µmol, 1.0 equiv) in CD_2Cl_2 (0.6 mL). The sample was shaken vigorously and directly subjected to NMR spectroscopic analysis indicating the formation of adduct **3ac** along with excess hydrosilane **2c** and small amounts of side product $[Et_3POSiEt_3]^+[BAr^F{}_4]^-$ (**10ac**).

*Selected NMR spectroscopic data for **3ac**:*

^{1}H NMR (500 MHz, CD_2Cl_2, 300 K): δ = –7.99 (d, *J* = 49.9 Hz, 1H), 0.31 (m_c, 3H), 0.53 (m_c, 3H), 0.83 (t, *J* = 7.3 Hz, 9H), 1.07 (dt, *J* = 15.7 Hz, *J* = 7.6 Hz, 9H*), 1.67 (m_c, 6H), 1.98 (s, 3H), 2.00 (s, 3H), 2.09 (s, 3H), 2.13 (s, 3H), 2.27 (s, 3H), 2.37 (s, 3H), 5.47 (s, 1H), 6.25 (s, 1H), 7.02 (s, 1H), 7.08 (s, 1H), 7.34 (dd, *J* = 7.2 Hz, *J* = 1.7 Hz, 1H), 7.56 (dd, *J* = 7.7 Hz, *J* = 1.7 Hz, 1H), 7.59 (dd, *J* = 7.7 Hz, *J* = 7.2 Hz, 1H), 7.61 (s, 4H), 7.78 (s, 8H*) ppm. **^{11}B NMR** (161 MHz, CD_2Cl_2, 300 K): δ = –6.6 ppm. **^{19}F{^{1}H} NMR** (471 MHz, CD_2Cl_2, 300 K): δ = –62.9 ppm. **^{31}P{^{1}H} NMR** (203 MHz, CD_2Cl_2, 300 K): δ = 40.1 ppm. **^{1}H,^{13}C HSQC NMR** (500/126 MHz, CD_2Cl_2, 300 K): δ = 0.31/7.22, 0.53/7.22, 0.83/7.22, 1.07/7.15, 1.67/19.9, 1.98/19.0, 2.00/18.6, 2.09/18.8, 2.13/21.0, 2.27/20.8, 2.37/20.5, 5.47/93.6, 6.25/92.3, 7.02/129.3, 7.08/129.5, 7.34/ 133.2, 7.56/128.4, 7.59/134.8, 7.61/117.4, 7.78/134.7 ppm. **^{1}H,^{13}C HMBC NMR** (500/126 MHz, CD_2Cl_2, 300 K): δ = –7.99/100.7, 1.98/84.4, 1.98/92.3, 1.98/93.6, 2.00/93.6, 2.00/100.7, 2.00/113.7, 2.09/92.3, 2.09/93.6, 2.09/113.7, 2.13/129.3, 2.13/135.0, 2.13/136.1, 2.27/129.5, 2.27/135.0, 2.27/135.7, 2.37/129.3, 2.37/129.5, 2.37/138.6, 5.47/18.6, 5.47/19.0, 5.47/92.3, 5.47/113.7, 6.25/18.8, 6.25/19.0, 6.35/93.6, 6.35/113.7, 7.02/20.5, 7.02/21.0, 7.02/129.5, 7.02/135.0, 7.07/20.5, 7.07/20.8, 7.07/129.3, 7.07/135.0, 7.34/128.4, 7.34/135.0, 7.34/142.3, 7.56/133.2, 7.56/142.3, 7.59/139.8, 7.59/144.1, 7.78/117.4, 7.78/161.5 ppm. **^{1}H,^{29}Si HMQC NMR** (500/99 MHz, CD_2Cl_2, 300 K, optimized for *J* = 8 Hz): δ = 0.31/41.0, 0.53/41.0, 0.83/41.0 ppm. **^{1}H,^{31}P HMQC NMR** (500/203 MHz, CD_2Cl_2, 300 K, optimized for *J* = 7 Hz): δ = –7.99/40.1, 1.07/40.1, 1.67/40.1, 2.09/40.1, 5.47/40.1 ppm.

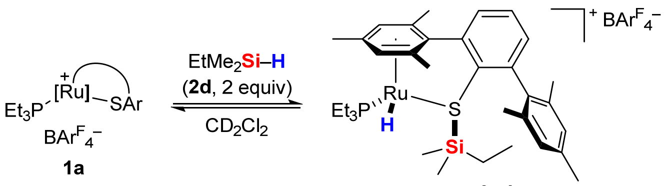

3ad
$C_{66}H_{64}BF_{24}PSSiRu$
M = 1516.2 g/mol

In an NMR tube, $EtMe_2SiH$ (**2d**, 2.1 mg, 24 µmol, 2.0 equiv) was added to a solution of $[(Et_3P)Ru(SDmp)]^+[BAr^F_4]^-$ (**1a**, 17 mg, 12 µmol, 1.0 equiv) in CD_2Cl_2 (0.6 mL). The sample was shaken vigorously and directly subjected to NMR spectroscopic analysis indicating the formation of adduct **3ad** along with excess hydrosilane **2d** and small amounts of side product $[Et_3POSiEtMe_2]^+[BAr^F_4]^-$ (**10ad**). Single crystals of **3ad** suitable for X-ray diffraction were obtained by treatment of ruthenium thiolate complex **1a** with a large excess of hydrosilane **2d**, followed by slow crystallization at –30 °C.

*Selected NMR spectroscopic data for **3ad**:*
^{1}H NMR (500 MHz, CD_2Cl_2, 300 K): δ = –8.08 (d, *J* = 49.9 Hz, 1H), 0.00 (s, 3H), 0.15–0.21 (m, 4H), 0.22–0.31 (m, 1H), 0.73 (dd, *J* = 7.8 Hz, *J* = 7.8 Hz, 3H), 1.07 (dt, *J* = 15.4 Hz, *J* = 7.7 Hz, 9H*), 1.65 (m_c, 6H), 1.97 (s, 3H), 2.00 (s, 3H), 2.09–2.13 (m, 6H), 2.27 (s, 3H), 2.36 (s, 3H), 5.52 (d, *J* = 3.3 Hz, 1H), 6.24 (s, 1H), 7.01 (s, 1H), 7.07 (s, 1H), 7.34 (dd, *J* = 7.3 Hz, *J* = 1.8 Hz, 1H), 7.57 (dd, *J* = 7.6 Hz, *J* = 1.7 Hz, 1H), 7.58–7.63 (s, 5H*), 7.78 (s, 8H*) ppm. **^{11}B NMR** (161 MHz, CD_2Cl_2, 300 K): δ = –6.6 ppm. **^{19}F{^{1}H} NMR** (471 MHz, CD_2Cl_2, 300 K): δ = –62.9 ppm. **^{31}P{^{1}H} NMR** (203 MHz, CD_2Cl_2, 300 K): δ = 40.2 ppm. **^{1}H,^{13}C HSQC NMR** (500/126 MHz, CD_2Cl_2, 300 K): δ = 0.00/0.19, {0.15–0.21}/1.6, {0.15–0.21}/10.5, {0.22–0.31}/10.5, 0.73/6.7, 1.07/7.2, 1.65/19.9, 1.97/18.9, 2.00/18.5, {2.09–2.13}/18.8, {2.09–2.13}/20.9, 2.27/21.2, 2.36/20.4, 5.52/94.0, 6.24/91.9, 7.01/129.1, 7.07/129.4, 7.34/133.3, 7.57/128.4, {7.58–7.63}/117.3, {7.58–7.63}/129.0, 7.78/134.7 ppm. **^{1}H,^{13}C HMBC NMR** (500/126 MHz, CD_2Cl_2, 300 K): δ = 1.97/84.9, 1.97/91.9, 1.97/94.0, 2.00/94.0, 2.00/100.4, 2.00/114.2, {2.09–2.13}/91.9, {2.09–2.13}/93.4, {2.09–2.13}/114.2, {2.09–2.13}/129.1, {2.09–2.13}/134.7, {2.09–2.13}/136.4, 2.27/129.4, 2.27/134.7, 2.27/135.8, 2.36/129.1, 2.36/129.4, 2.36/138.5, 5.52/18.5, 5.52/18.9, 5.52/91.9, 5.52/114.2, 6.24/18.8, 6.24/18.9, 6.24/94.0, 6.24/114.2, 7.01/20.4, 7.01/20.9, 7.01/129.4, 7.01/134.7, 7.07/20.4, 7.07/21.2, 7.07/129.1, 7.07/134.7, 7.34/128.4, 7.34/134.7, 7.34/142.3, 7.57/114.2, 7.57/133.3, 7.57/142.3, {7.58–7.63}/134.7, {7.58–7.63}/139.7, {7.58–7.63}/144.3, 7.78/117.3, 7.78/162 ppm. **^{1}H,^{29}Si HMQC NMR** (500/99 MHz, CD_2Cl_2, 300 K, optimized for *J* = 8 Hz): δ = 0.00/39.0, {0.15–0.21}/39.0, 0.73/39.0 ppm. **^{1}H,^{31}P HMQC NMR** (500/203 MHz, CD_2Cl_2, 300 K, optimized for *J* = 7 Hz): δ = –8.08/40.2, 1.07/40.2, 1.65/40.2, {2.09–2.13}/40.2, 5.52/40.2 ppm. **X-ray**: For X-ray data, see section 7.2.

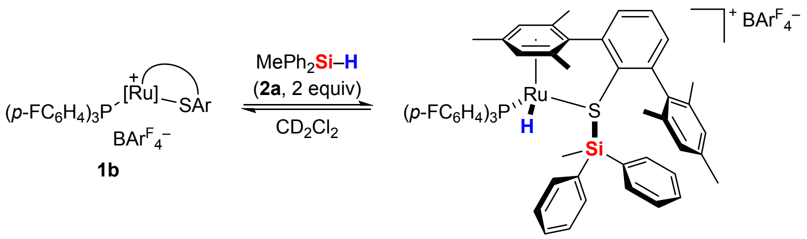

3ba
$C_{87}H_{63}BF_{27}PSSiRu$
M = 1824.4 g/mol

In an NMR tube, $MePh_2SiH$ (**2a**, 4.8 mg, 24 μmol, 2.0 equiv) was added to a solution of $[\{(p\text{-}FC_6H_4)_3P\}Ru(SDmp)]^+[BAr^F{}_4]^-$ (**1b**, 20 mg, 12 μmol, 1.0 equiv) in CD_2Cl_2 (0.6 mL). The sample was shaken vigorously and directly subjected to NMR spectroscopic analysis indicating the formation of adduct **3ba** along with excess hydrosilane **2a** and small amounts of side product $[(p\text{-}FC_6H_4)_3POSiMePh_2]^+[BAr^F{}_4]^-$ (**10ba**).

*Selected NMR spectroscopic data for **3ba** in CD_2Cl_2:*

^{1}**H NMR** (500 MHz, CD_2Cl_2, 300 K): δ = –7.54 (d, *J* = 47.3 Hz, 1H), 0.58 (s, 3H), 1.47 (s, 3H), 1.82 (s, 3H), 1.88 (s, 3H), 2.16 (s, 3H), 2.18 (s, 3H), 2.52 (s, 3H), 4.45 (s, 1H), 6.06 (s, 1H), 6.25 (s, 1H), 6.43 (d, *J* = 7.3 Hz, 2H), 6.83 (s, 1H), 6.99 (d, *J* = 7.3 Hz, 2H), 7.07 (dd, *J* = 7.4 Hz, *J* = 7.3 Hz, 2H), 7.11 (dd, *J* = 8.2 Hz, *J* = 8.2 Hz, 6H), 7.23–7.32 (m, 9H), 7.36–7.47 (m, 2H*), 7.58–7.63 (m, 5H*), 7.66 (dd, *J* = 7.4 Hz, *J* = 7.3 Hz, 1H), 7.78 (s, 8H*) ppm. ^{11}**B NMR** (161 MHz, CD_2Cl_2, 300 K): δ = –6.6 ppm. ^{19}**F{^{1}H} NMR** (471 MHz, CD_2Cl_2, 300 K): δ = –108.6, –62.9 ppm. ^{31}**P{^{1}H} NMR** (203 MHz, CD_2Cl_2, 300 K): δ = 48.5 ppm. ^{1}**H,**^{13}**C HSQC NMR** (500/126 MHz, CD_2Cl_2, 300 K): δ = 0.58/–1.7, 1.47/18.2, 1.82/17.5, 1.88/20.8, 2.16/21.3, 2.18/20.6, 2.52/19.4, 4.45/104.9, 6.06/129.4, 6.25/89.4, 6.43/133.3, 6.83/128.3, 6.99/136.9, 7.07/127.7, 7.11/115.7, {7.23–7.32}/127.4, {7.23–7.32}/133.7, {7.23–7.32}/135.6, {7.36–7.47}/129.3, {7.36–7.47}/131.4, {7.58–7.63}/117.4, {7.58–7.63}/128.3, 7.66/130.0, 7.78/134.7 ppm. ^{1}**H,**^{13}**C HMBC NMR** (500/126 MHz, CD_2Cl_2, 300 K): δ = –7.54/104.9, 0.58/129.5, 0.58/134.1, 1.47/88.2, 1.47/89.4, 1.47/104.9, 1.82/104.7, 1.82/111.7, 1.88/129.4, 1.88/134.2, 1.88/135.3, 2.16/128.3, 2.16/134.2, 2.16/134.8, 2.18/128.3, 2.18/129.4, 2.18/138.2, 2.52/89.4, 2.52/95.0, 2.52/111.7, 4.45/17.5, 4.45/18.2, 6.06/20.6, 6.06/20.8, 6.06/128.3, 6.06/134.2, 6.25/18.2, 6.25/19.4, 6.25/104.9, 6.25/111.7, 6.43/129.4, 6.83/20.6, 6.83/21.3, 6.83/129.4, 6.83/134.2, 6.99/131.4, 7.07/129.5, {7.23–7.32}/128.3, {7.23–7.32}/143.2, {7.23–7.32}/140.8, {7.23–7.32}/163.8, {7.58–7.63}/133.8, {7.58–7.63}/134.7, {7.58–7.63}/140.8, 7.66/139.9, 7.66/145.2, 7.78/117.5 ppm. ^{1}**H,**^{19}**F HMQC NMR** (500/471 MHz, CD_2Cl_2, 300 K, optimized for *J* = 30 Hz): δ = 7.07/–108.6, {7.23–7.32}/–108.6, {7.58–7.63}/–62.9, 7.78/–62.9 ppm. ^{1}**H,**^{29}**Si HMQC NMR** (500/99 MHz, CD_2Cl_2, 300 K, optimized for *J* = 7 Hz): δ = 0.58/20.1, 6.43/20.1, 6.99/20.1 ppm. ^{1}**H,**^{31}**P HMQC NMR** (500/

203 MHz, CD_2Cl_2, 300 K, optimized for *J* = 7 Hz): δ = –7.54/48.5, 1.82/48.5, 2.52/48.5, 4.45/48.5, {7.23–7.32}/48.5 ppm.

This experiment was also carried out in C_6D_6 (0.6 mL) as solvent, leading to the same result. All remaining NMR experiments, however, were performed in CD_2Cl_2 due to a better signal resolution in the ^{1}H NMR spectrum (see NMR spectra in section 6).

*Selected NMR spectroscopic data for **3ba** in C_6D_6:*
^{1}H NMR (500 MHz, C_6D_6, 300 K): δ = –7.89 (d, *J* = 47.5 Hz, 1H), 0.20 (s, 3H), 0.89 (s, 3H), 1.33 (s, 3H), 1.59 (s, 3H), 1.93 (s, 6H), 2.00 (s, 3H), 3.80 (s, 1H), 5.44 (s, 1H), 5.84 (s, 1H), 6.13 (d, *J* = 7.3 Hz, 2H), 6.49 (ddd, *J* = 10.7 Hz, *J* = 8.3 Hz, *J* = 2.8 Hz, 2H*), 6.65 (dd, *J* = 8.0 Hz, *J* = 8.0 Hz, 6H*), 6.69–7.07 (m, 13H*) 7.67 (s, 4H*), 8.39 (s, 8H*) ppm. **^{11}B NMR** (161 MHz, C_6D_6, 300 K): δ = –5.9 ppm. **^{19}F{^{1}H} NMR** (471 MHz, C_6D_6, 300 K): δ = –107.5, –62.0 ppm. **^{31}P{^{1}H} NMR** (203 MHz, C_6D_6, 300 K): δ = 48.6 ppm.

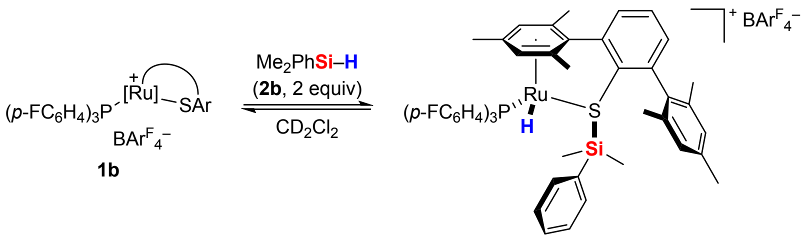

3bb
$C_{82}H_{61}BF_{27}PSSiRu$
M = 1762.3 g/mol

In an NMR tube, Me_2PhSiH (**2b**, 3.3 mg, 24 μmol, 2.0 equiv) was added to a solution of $[\{(p\text{-}FC_6H_4)_3P\}Ru(SDmp)]^+[BAr^F{}_4]^-$ (**1b**, 20 mg, 12 μmol, 1.0 equiv) in CD_2Cl_2 (0.6 mL). The sample was shaken vigorously and directly subjected to NMR spectroscopic analysis indicating the formation of adduct **3bb** along with excess hydrosilane **2b** and small amounts of side product $[(p\text{-}FC_6H_4)_3POSiMe_2Ph]^+[BAr^F{}_4]^-$ (**10bb**).

*Selected NMR spectroscopic data for **3bb**:*
^{1}**H NMR** (500 MHz, CD_2Cl_2, 300 K): δ = –7.67 (d, *J* = 48.5 Hz, 1H), 0.01 (s, 3H), 0.55 (s, 3H), 1.48 (s, 3H), 1.79 (s, 3H), 2.00 (d, 3H), 2.17 (s, 3H), 2.33 (s, 3H), 2.36 (s, 3H), 4.64 (s, 1H), 6.32 (s, 1H), 6.69 (d, *J* = 6.5 Hz, 2H), 6.76 (s, 1H), 6.89 (s, 1H), 7.08 (dd, *J* = 8.5 Hz, *J* = 8.5 Hz, 6H), 7.17 (dd, *J* = 6.7 Hz, *J* = 6.7 Hz, 2H), 7.31–7.38 (m, 8H), 7.57 (d, *J* = 8.0 Hz, 1H), 7.61 (s, 4H*), 7.66 (dd, *J* = 7.6 Hz, *J* = 7.6 Hz, 1H), 7.78 (s, 8H*) ppm. ^{11}**B NMR** (161 MHz, CD_2Cl_2, 300 K): δ = –6.6 ppm. ^{19}**F{**^{1}**H} NMR** (471 MHz, CD_2Cl_2, 300 K): δ = –108.7, –62.8 ppm. ^{31}**P{**^{1}**H} NMR** (203 MHz, CD_2Cl_2, 300 K): δ = 48.7 ppm. ^{1}**H,**^{13}**C HSQC NMR** (500/126 MHz, CD_2Cl_2, 300 K): δ = 0.01/–0.19, 0.55/3.81, 1.48/18.3, 1.79/17.7, 2.00/21.1, 2.17/21.0, 2.33/20.5, 2.36/19.3, 4.64/101.8, 6.32/91.2, 6.69/133.7, 6.76/129.7, 6.89/128.7, 7.08/115.7, 7.17/127.7, {7.31–7.38}/133.5, {7.31–7.38}/135.4, 7.42/129.1, 7.57/128.4, 7.61/117.3, 7.66/129.9, 7.78/134.7 ppm. ^{1}**H,**^{13}**C HMBC NMR** (500/126 MHz, CD_2Cl_2, 300 K): δ = 0.01/133.6, 0.55/133.6, 1.48/88.1, 1.48/91.2, 1.48/101.8, 1.79/101.8, 1.79/104.0, 1.79/113.5, 2.00/128.7, 2.00/134.2, 2.00/135.8, 2.17/129.8, 2.17/134.2, 2.17/135.7, 2.33/128.7, 2.33/129.7, 2.33/138.5, 2.36/91.2, 2.36/94.4, 2.36/113.5, 4.64/17.7, 4.64/18.3, 4.64/91.2, 4.64/113.5, 6.32/18.3, 6.32/19.3, 6.32/101.8, 6.32/113.5, 6.76/20.5, 6.76/128.7, 6.76/134.2, 6.89/20.5, 6.89/21.1, 6.89/129.7, 6.89/134.2, 7.07/129.7, 7.17/127.1, 7.17/133.7, 7.17/135.3, {7.31–7.38}/128.4, {7.31–7.38}/141.7, {7.31–7.38}/164.0, 7.57/113.5, 7.57/141.7, 7.57/133.5, 7.67/139.7, 7.67/144.5 ppm. ^{1}**H,**^{29}**Si HMQC NMR** (500/99 MHz, CD_2Cl_2, 300 K, optimized for *J* = 8 Hz): δ = 0.01/29.8, 0.55/29.8, 6.69/29.8 ppm. ^{1}**H,**^{31}**P HMQC NMR** (500/203 MHz, CD_2Cl_2, 300 K, optimized for *J* = 7 Hz): δ = –7.67/48.7, 1.48/48.7, 1.79/48.7, 2.36/48.7, 4.64/48.7, 7.08/48.7, {7.31–7.38}/48.7 ppm.

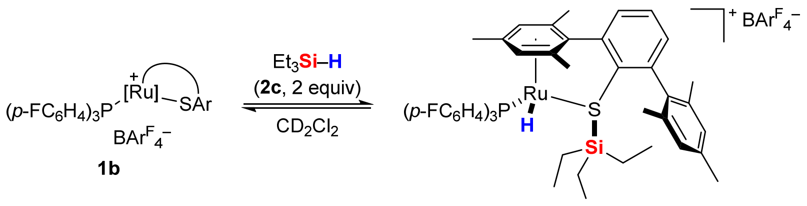

3bc
$C_{80}H_{65}BF_{27}PSSiRu$
M = 1742.4 g/mol

In an NMR tube, Et_3SiH (**2c**, 2.8 mg, 24 µmol, 2.0 equiv) was added to a solution of [{(*p*-$FC_6H_4)_3P$}Ru(SDmp)]$^+$[BAr$^F{}_4$]$^-$ (**1b**, 20 mg, 12 µmol, 1.0 equiv) in CD_2Cl_2 (0.6 mL). The sample was shaken vigorously and directly subjected to NMR spectroscopic analysis indicating the formation of adduct **3bc** along with unreacted hydrosilane **2c** and small amounts of side product [(*p*-$FC_6H_4)_3POSiEt_3$]$^+$[BAr$^F{}_4$]$^-$ (**10bc**).

*Selected NMR spectroscopic data for **3bc**:*

^{1}H NMR (500 MHz, CD_2Cl_2, 300 K): δ = –7.61 (d, *J* = 49.9 Hz, 1H), 0.22 (m_c, 6H), 0.62–0.70 (m, 9H*), 1.45 (s, 3H), 1.83 (s, 3H), 2.05 (s, 3H), 2.17 (d, *J* = 2.4 Hz, 3H), 2.26 (s, 3H), 2.34 (s, 3H), 4.74 (d, *J* = 3.5 Hz, 1H), 6.38 (s, 1H), 6.89 (s, 1H), 7.04 (s, 1H), 7.19 (ddd, *J* = 8.5 Hz, *J* = 8.5 Hz, *J* = 1.3 Hz, 6H), 7.37 (dd, *J* = 7.6 Hz, *J* = 1.0 Hz, 1H), 7.51 (m_c, 6H), 7.54 (d, *J* = 7.3 Hz, 1H), 7.61 (s, 4H), 7.66 (dd, *J* = 7.8 Hz, *J* = 7.8 Hz, 1H), 7.77 (s, 8H) ppm. **^{11}B NMR** (161 MHz, CD_2Cl_2, 300 K): δ = –6.6 ppm. **^{19}F{^{1}H} NMR** (471 MHz, CD_2Cl_2, 300 K): δ = –108.7, –62.8 ppm. **^{31}P{^{1}H} NMR** (203 MHz, CD_2Cl_2, 300 K): δ = 48.4 ppm. **^{1}H,^{13}C HSQC NMR** (500/126 MHz, CD_2Cl_2, 300 K): δ = 0.22/7.5, {0.62–0.70}/2.4, 1.45/18.1, 1.83/18.2, 2.05/21.3, 2.17/18.9, 2.26/20.8, 2.34/20.4, 4.74/99.4, 6.38/92.0, 6.89/128.9, 7.04/129.5, 7.19/115.9, 7.37/133.3, 7.51/135.5, 7.54/128.4, 7.61/117.3, 7.66/129.9, 7.77/134.7 ppm. **^{1}H,^{13}C HMBC NMR** (500/126 MHz, CD_2Cl_2, 300 K): δ = 1.45/88.3, 1.45/92.0, 1.45/99.4, 1.83/99.4, 1.83/103.6, 1.83/113.9, 2.05/128.9, 2.05/134.5, 2.05/136.0, 2.17/92.0, 2.17/94.1, 2.17/113.9, 2.26/129.4, 2.26/134.5, 2.26/135.6, 2.34/128.9, 2.34/129.5, 2.34/138.7, 4.75/18.1, 4.75/18.2, 4.75/92.0, 4.75/113.9, 6.38/18.1, 6.38/18.9, 6.38/99.4, 6.38/113.9, 6.89/20.4, 6.89/21.3, 6.89/129.5, 6.89/134.5, 7.04/20.4, 7.04/20.8, 7.04/128.9, 7.04/134.5, 7.19/129.7, 7.19/164.1, 7.37/128.4, 7.37/142.1, 7.51/164.1, 7.54/133.3, 7.54/142.1, 7.61/134.7, 7.66/139.5, 7.66/144.1, 7.77/117.3 ppm. **^{1}H,^{29}Si HMQC NMR** (500/99 MHz, CD_2Cl_2, 300 K, optimized for *J* = 8 Hz): δ = 0.22/41.6, {0.62–0.70}/41.6 ppm. **^{1}H,^{31}P HMQC NMR** (500/203 MHz, CD_2Cl_2, 300 K, optimized for *J* = 7 Hz): δ = –7.61/48.8, 1.83/48.8, 2.17/48.8, 4.74/48.8, 7.19/48.8, 7.51/48.8 ppm.

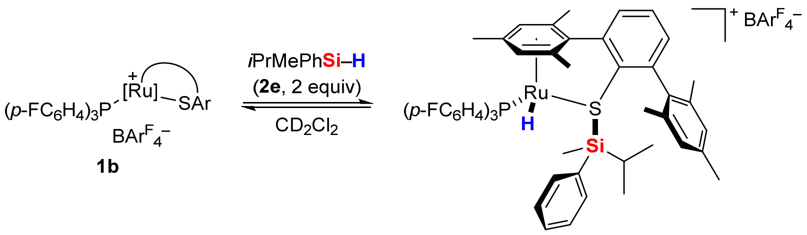

3be
$C_{84}H_{65}BF_{27}PSSiRu$
M = 1790.4 g/mol

In an NMR tube, *i*PrMePhSiH (**2e**, 3.9 mg, 24 µmol, 2.0 equiv) was added to a solution of [{(*p*-$FC_6H_4)_3$P}Ru(SDmp)]$^+$[BAr$^F{}_4$]$^-$ (**1b**, 20 mg, 12 µmol, 1.0 equiv) in CD_2Cl_2 (0.6 mL). The sample was shaken vigorously and directly subjected to NMR spectroscopic analysis indicating the formation of adduct **3be** (d.r. = 2.3:1) along with unreacted hydrosilane **2e** and small amounts of side product [(*p*-$FC_6H_4)_3$POSi*i*PrMePh]$^+$[BAr$^F{}_4$]$^-$ (**10be**).

Selected NMR spectroscopic data for the major diastereomer of ***3be****:*

^{1}H NMR (500 MHz, CD_2Cl_2, 300 K): δ = –7.74 (d, *J* = 48.5 Hz, 1H), 0.16 (s, 3H), 0.28 (d, *J* = 7.4 Hz, 3H*), 0.71 (d, *J* = 7.4 Hz, 3H), 1.46 (s, 3H), 1.83 (s, 3H), 1.94 (d, *J* = 2.5 Hz, 3H), 2.08 (s, 3H*), 2.18–2.21 (m, 6H), 4.65 (d, *J* = 3.2 Hz, 1H), 6.27 (s, 1H), 6.35 (s, 1H), 6.54 (d, *J* = 7.4 Hz, 2H), 6.83 (s, 1H), 7.03–7.11 (m, 1H*), 7.23 (ddd, *J* = 8.5 Hz, *J* = 8.5 Hz, *J* = 1.2 Hz, 6H*), 7.31–7.35 (m, 2H*), 7.53 (d, *J* = 7.8 Hz, 1H*), 7.56–7.60 (m, 6H*), 7.61 (s, 4H*), 7.62–7.64 (m, 1H*), 7.78 (s, 8H*) ppm. **^{11}B NMR** (161 MHz, CD_2Cl_2, 300 K): δ = –6.6 ppm. **^{19}F{^{1}H} NMR** (471 MHz, CD_2Cl_2, 300 K): δ = –108.4, –62.8 ppm. **^{31}P{^{1}H} NMR** (203 MHz, CD_2Cl_2, 300 K): δ = 47.2 ppm. **^{1}H,^{13}C HSQC NMR** (500/126 MHz, CD_2Cl_2, 300 K): δ = 0.16/–4.8, 0.28/17.5, 0.71/17.0, 1.46/ 18.1, 1.83/17.7, 1.94/18.1, 2.08/21.1, {2.18–2.21}/20.5, {2.18–2.21}/21.7, 4.65/103.6, 6.27/90.1, 6.35/129.5, 6.54/133.1, 6.83/128.4, {7.03–7.11}/129.7, 7.23/116.0, {7.31–7.35}/129.9, {7.31–7.35}/133.7, 7.53/128.3, {7.56–7.60}/135.6, 7.61/117.1, {7.62–7.64}/129.7, 7.78/134.7 ppm. **^{1}H,^{13}C HMBC NMR** (500/126 MHz, CD_2Cl_2, 300 K): δ = –7.74/104.5, 1.46/88.6, 1.46/90.1, 1.46/103.6, 1.83/103.6, 1.83/104.5, 1.83/112.3, 1.94/90.1, 1.94/95.5, 1.94/112.3, 2.08/129.5, 2.08/134.4, 2.08/135.4, {2.18–2.21}/128.4, {2.18–2.21}/129.5, {2.18–2.21}/134.3, {2.18–2.21}/ 134.8, {2.18–2.21}/138.3, 4.65/17.7, 4.65/18.1, 4.65/90.1, 4.65/112.3, 6.27/18.1, 6.27/103.6, 6.27/112.3, 6.35/21.1, 6.35/21.7, 6.35/128.4, 6.35/134.3, 6.54/129.9, 6.83/20.5, 6.83/21.7, 6.83/129.5, 6.83/134.3, 7.23/129.5, {7.31–7.35}/128.3, {7.31–7.35}/133.1, {7.31–7.35}/134.3, {7.31–7.35}/141.0, 7.53/133.7, 7.53/141.0, 7.53/112.3, {7.56–7.60}/164.3, 7.61/134.7, {7.62–7.64}/140.0, {7.62–7.64}/144.9, 7.78/117.1 ppm. **^{1}H,^{29}Si HMQC** (500/99 MHz, CD_2Cl_2, 300 K, optimized for *J* = 8 Hz): δ = 0.16/32.1, 0.28/32.1, 0.71/32.1, 6.54/32.1 ppm. **^{1}H,^{31}P HMQC** (500/203 MHz, CD_2Cl_2, 300 K, optimized for *J* = 7 Hz): δ = –7.74/47.2, 1.94/47.2, 4.65/47.2, {7.56–7.60}/47.2 ppm.

Selected NMR spectroscopic data for the minor diastereomer of ***3be***:

^{1}H NMR (500 MHz, CD_2Cl_2, 300 K): δ = –7.58 (d, *J* = 48.5 Hz, 1H), 0.28 (d, *J* = 7.4 Hz, 3H*), 0.41 (d, *J* = 6.9 Hz, 3H), 0.49 (s, 3H), 1.41 (s, 3H), 1.77 (s, 3H), 1.90 (s, 3H*), 2.26 (s, 3H), 2.28 (s, 3H), 2.35 (s, 3H), 4.49 (d, *J* = 3.1 Hz, 1H), 6.16 (s, 1H), 6.85 (s, 2H), 6.91 (s, 1H*), 6.97 (s, 1H), 7.61 (s, 4H*), 7.78 (s, 8H*) ppm. **^{11}B NMR** (161 MHz, CD_2Cl_2, 300 K): δ = –6.6 ppm. **^{19}F{^{1}H} NMR** (471 MHz, CD_2Cl_2, 300 K): δ = –108.8, –62.8 ppm. **^{31}P{^{1}H} NMR** (203 MHz, CD_2Cl_2, 300 K): δ = 47.6 ppm. **^{1}H,^{13}C HSQC NMR** (500/126 MHz, CD_2Cl_2, 300 K): δ = 0.28/–3.8, 0.42/–8.3, 0.49/–5.5, 1.41/18.2, 1.77/17.7, 1.90/20.0, 2.26/19.5, 2.28/20.9, 2.35/20.5, 4.49/102.8, 6.18/90.8, 6.85/135.0, 6.91/127.8, 6.97/129.0, 7.61/117.1, 7.78/134.7 ppm. **^{1}H,^{13}C HMBC** (500/126 MHz, CD_2Cl_2, 300 K): δ = 1.41/87.6, 1.41/90.8, 1.41/102.8, 1.77/102.8, 1.77/104.1, 1.77/112.9, 1.90/127.8, 1.90/134.6, 1.90/135.3, 2.26/90.8, 2.26/94.8, 2.26/112.9, 2.28/129.9, 2.28/134.6, 2.28/135.7, 2.35/127.8, 2.35/129.9, 2.35/139.1, 4.49/18.2, 4.49/90.8, 4.49/112.9, 6.16/19.5, 6.16/102.8, 6.16/112.9, 6.91/20.5, 6.91/129.9, 6.91/134.6, 6.97/127.8, 6.97/134.6 ppm. **^{1}H,^{29}Si HMQC** (500/99 MHz, CD_2Cl_2, 300 K, optimized for *J* = 8 Hz): δ = 0.28/33.4, 0.42/33.4, 0.49/33.4, 6.85/33.4 ppm. **^{1}H,^{31}P HMQC** (500/203 MHz, CD_2Cl_2, 300 K, optimized for *J* = 7 Hz): δ = –7.58/47.6, 2.26/47.6, 4.49/47.6 ppm.

2.2 Characterization of Side Products $[R_3POSiR'_3]^+[BAr^F_4]^-$ (10)

Table S1. ^{29}Si and ^{31}P NMR chemical shifts of $[R_3POSiR'_3]^+[BAr^F_4]^-$ (**10**).[a]

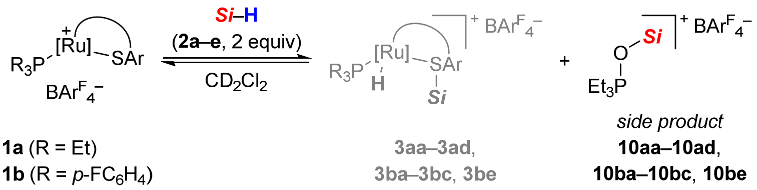

entry	reaction	side product	$\delta(^{29}Si)$ [ppm][b]	$\delta(^{31}P)$ [ppm]
1	**1a** + $MePh_2SiH$ (**2a**) → **3aa**	$[Et_3POSiMePh_2]^+[BAr^F_4]^-$ (**10aa**)	8.8[c]	92.9[c,d]
2	**1a** + Me_2PhSiH (**2b**) → **3ab**	$[Et_3POSiMe_2Ph]^+[BAr^F_4]^-$ (**10ab**)	21.9[c]	91.2[c]
3	**1a** + Et_3SiH (**2c**) → **3ac**	$[Et_3POSiEt_3]^+[BAr^F_4]^-$ (**10ac**)	36.7[e]	89.0[e]
4	**1a** + $EtMe_2SiH$ (**2d**) → **3ad**	$[Et_3POSiEtMe_2]^+[BAr^F_4]^-$ (**10ad**)	35.4[e]	89.9[e]
5	**1b** + $MePh_2SiH$ (**2a**) → **3ba**	$[(p\text{-}FC_6H_4)_3POSiMePh_2]^+[BAr^F_4]^-$ (**10ba**)[f]	11.5[e]	52.8[e]
6	**1b** + Me_2PhSiH (**2b**) → **3bb**	$[(p\text{-}FC_6H_4)_3POSiMe_2Ph]^+[BAr^F_4]^-$ (**10bb**)	25.1[e]	51.6[e]
7	**1b** + Et_3SiH (**2c**) → **3bc**	$[(p\text{-}FC_6H_4)_3POSiEt_3]^+[BAr^F_4]^-$ (**10bc**)	–[g]	51.3[e]
8	**1b** + *i*PrMePhSiH (**2e**) → **3be**	$[(p\text{-}FC_6H_4)_3POSi\textit{i}PrMePh]^+[BAr^F_4]^-$ (**10be**)	24.6[e]	52.0[e]

[a]All reactions were performed in an NMR tube in CD_2Cl_2 (20 mM) using ruthenium thiolate complex **1a** or **1b** (1.0 equiv) and the corresponding hydrosilane **1a**–**1e** (2.0 equiv). The reaction mixture was directly subjected to NMR spectroscopic analysis. [b]$^1H,^{29}Si$ HMQC NMR spectroscopy optimized for J = 8 Hz. [c]In CD_2Cl_2 at 250 K. [d]$\delta(^{31}P)$ = 91.3 ppm in C_6D_6 at 300 K. [e]In CD_2Cl_2 at 300 K. [f]HRMS (ESI) for $C_{31}H_{25}F_3OPSi$ $[M–BAr^F_4]^+$: calcd m/z 529.1359, found 529.1366. [g]No resonance signal detected.

Single crystals of $[Et_3POSiMe_2Ph]^+[BAr^F_4]^-$ (**10ab)** suitable for X-ray diffraction were obtained from a solution of ruthenium(II) thiolate complex **1a** and excess hydrosilane **2b** in toluene layered by *n*-hexane at –30 °C. For the crystallographic data, see section 7.3.

2.3 Independent Preparation of $[Et_3POSiMePh_2]^+[BAr^F_4]^-$ (10aa) by Addition of Et_3PO (11a) to In-Situ Generated Adduct 3ba

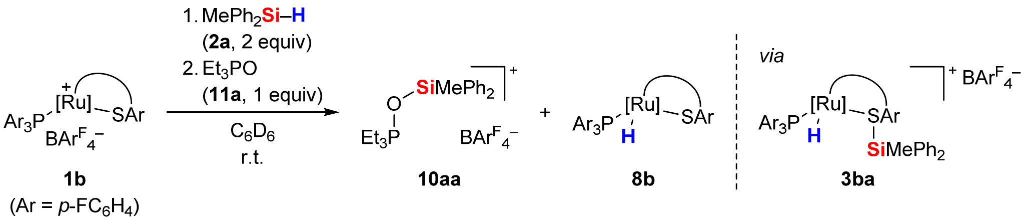

In an NMR tube, $MePh_2SiH$ (**2a**, 2.4 mg, 12 µmol, 2.0 equiv) was added to a solution of $[\{(p\text{-}FC_6H_4)_3P\}Ru(SDmp)]^+[BAr^F_4]^-$ (**1b**, 10 mg, 6.2 µmol, 1.0 equiv) in C_6D_6 (0.6 mL). The sample

was shaken vigorously, followed by addition of Et_3PO (0.83 mg, 6.2 µmol, 1.0 equiv). The sample was again shaken vigorously and directly subjected to NMR spectroscopic analysis indicating the formation of **10aa** along with ruthenium hydride complex **8b**.

Selected NMR spectroscopic data for ***10aa****:*

^{1}H NMR (500 MHz, C_6D_6, 300 K): δ = 0.04–0.21 (m, 9H), 0.23 (s, 3H), 0.63–0.77 (m, 6H), 7.08–7.12 (m, 4H*), 7.66 (s, 4H*), 8.32 (s, 8H*) ppm. **^{31}P{^{1}H} NMR** (203 MHz, C_6D_6, 300 K): δ = 91.3 ppm. **^{1}H,^{29}Si HMQC** (500/99 MHz, C_6D_6, 300 K, optimized for *J* = 8 Hz): δ = 0.23/7.9, {7.08–7.12}/7.9 ppm. The NMR spectroscopic data are in accordance with those obtained for the side product in the reaction of $[(Et_3P)Ru(SDmp)]^+[BAr^F_4]^-$ (**1a**) and $MePh_2SiH$ (**2a**) (cf. Table S1, entry 1).

Selected NMR spectroscopic data for ***8b****:*

^{1}H NMR (500 MHz, C_6D_6, 300 K): δ = –8.42 (d, *J* = 53.4 Hz, 1H), 1.03 (s, 3H), 1.66 (s, 3H), 2.03 (s, 3H), 2.14 (s, 3H), 2.16 (s, 3H), 2.27 (s, 3H), 4.27 (d, *J* = 2.9 Hz, 1H), 5.36 (s, 1H), 6.61 (dd, *J* = 8.5 Hz, *J* = 8.5 Hz, 6H), 6.67 (s, 1H), 6.87 (s, 1H), 7.29–7.37 (m, 4H) ppm. **^{19}F{^{1}H} NMR** (471 MHz, C_6D_6, 300 K): δ = –109.9 ppm. **^{31}P{^{1}H} NMR** (203 MHz, C_6D_6, 300 K): δ = 53.4 ppm.

2.4 Control Experiment: Probing the Reactivity of $[Et_3POSiMePh_2]^+[BAr^F_4]^-$ (10aa)

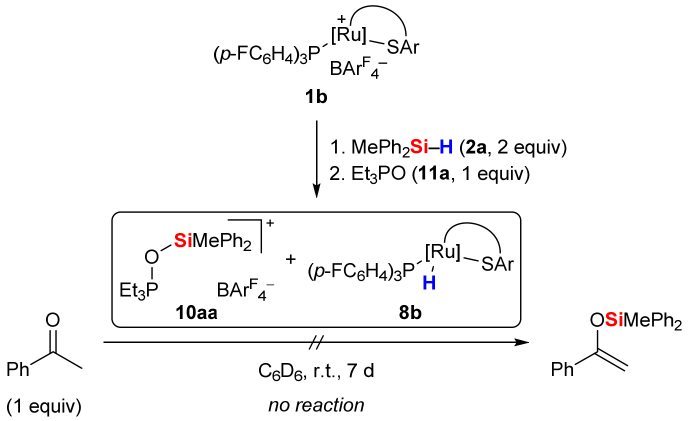

In an NMR tube, $MePh_2SiH$ (**2a**, 2.4 mg, 12 µmol, 2.0 equiv) was added to a solution of $[\{(p\text{-}FC_6H_4)_3P\}Ru(SDmp)]^+[BAr^F_4]^-$ (**1b**, 10 mg, 6.2 µmol, 1.0 equiv) in C_6D_6 (0.6 mL). The sample was shaken vigorously for 5 min, followed by addition of Et_3PO (**11a**, 0.83 mg, 6.2 µmol, 1.0 equiv). After shaking for additional 5 min, acetophenone (0.74 mg, 6.2 µmol, 1.0 equiv) was added, and the sample was maintained at room temperature for 7 d. No reaction was observed as indicated by NMR spectroscopic analysis.

2.5 Preparation of Silyl Thioether $Ph_2MeSiSDmp$

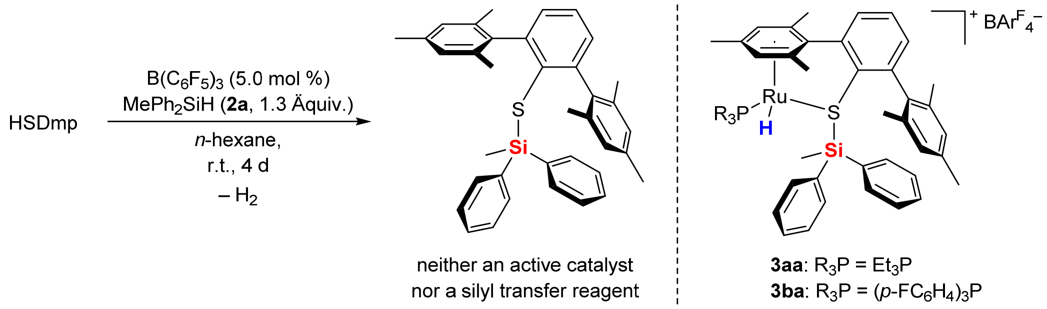

According to a reported procedure by Rosenberg and co-workers,[S25] $B(C_6F_5)_3$ (3.7 mg, 7.2 µmol, 5.0 mol %) was added to a solution of 2,6-bis(2,4,6-trimethylphenyl)phenylthiol (50 mg, 0.14 mmol, 1.0 equiv) und $MePh_2SiH$ (**2a**, 37 mg, 0.19 mmol, 1.3 equiv) in *n*-hexane (1 mL). The resulting mixture was stirred at room temperature for 4 d, followed by filtration and removal of the solvent under reduced pressure. The residue was dried under vacuum (0.5 mbar) at 80 °C, affording the title compound (64 mg, 82%) as a white solid. $^{19}F\{^1H\}$ NMR spectroscopic analysis indicated no contamination of the product by $B(C_6F_5)_3$.

Analytical data for $Ph_2MeSiSDmp$:

M.p. = 175 °C (*n*-hexane). **IR** (ATR): $\tilde{\nu}$ = 3043, 2993, 2912, 2851, 1610, 1562, 1446, 1421, 1372, 1245, 1103, 1030, 848, 801, 727, 695 cm^{-1}. **^{1}H NMR** (500 MHz, C_6D_6, 300 K): δ = 0.50 (s, 3H), 2.14 (s, 12H), 2.24 (s, 6H), 6.74 (s, 4H), 6.97–7.03 (m, 6H), 7.04–7.11 (m, 7H) ppm. **$^{13}C\{^1H\}$ NMR** (126 MHz, C_6D_6, 300 K): δ = –1.6, 20.9, 21.0, 127.3, 128.0, 128.3, 129.0, 129.9, 131.4, 134.8, 135.5, 135.9, 136.0, 139.0, 147.7 ppm. **^{29}Si DEPT NMR** (99 MHz, C_6D_6, 300 K, optimized for J = 8 Hz): δ = 2.9 ppm. **HRMS** (APCI) for $C_{37}H_{39}SSi$ $[M+H]^+$: calcd m/z 543.2536, found 543.2531.

In contrast to cationic silylthioruthenium hydride intermediates **3aa** and **3ba**, neutral silyl thioether $Ph_2MeSiSDmp$ proved to be not a potent silyl transfer agent.

3 Mechanistic Control Experiment with a Silicon-Stereogenic Hydrosilane

3.1 Racemization Experiment of Enantioenriched Hydrosilane (^{Si}S)-2e with Ruthenium Thiolate Complex 1b

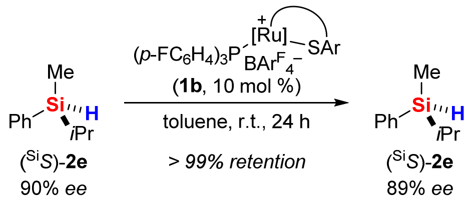

In a 2-mL vial, enantioenriched *i*PrMePhSiH ((^{Si}S)-**2e**, 3.6 mg, 22 μmol, 1.0 equiv, 90% *ee*) was added to a solution of [{(p-$FC_6H_4)_3P$}Ru(SDmp)]$^+$[$BAr^F{}_4$]$^-$ (**1b**, 3.6 mg, 2.2 μmol, 10 mol %) in toluene (0.4 mL), and the resulting reaction mixture was stirred at room temperature for 24 h. The reaction mixture was filtrated through a short pad of silica gel (cyclohexane/*tert* butyl methyl ether 10:1) and concentrated under reduced pressure. Hydrosilane (^{Si}S)-**2e** (89% *ee*) was reisolated as a colorless oil with >99% retention of configuration at the silicon atom. The enantiomeric excess was determined by HPLC analysis on a chiral stationary phase (Daicel OJ-RH, column temperature 20 °C, solvent MeCN:H_2O = 60:40, flow rate 0.40 mL/min, λ = 230 nm): t_R = 29.2 min for (^{Si}R)-**2e** (minor enantiomer), t_R = 31.8 min for (^{Si}S)-**2e** (major enantiomer).

4 Mechanistic Control Experiments with a Deuterium-Labeled Hydrosilane

4.1 Si–H Bond Activation of Deuterium-Labeled Dimethylphenylsilane (2b-*d*$_1$) with Ruthenium(II) Thiolate Complex 1b

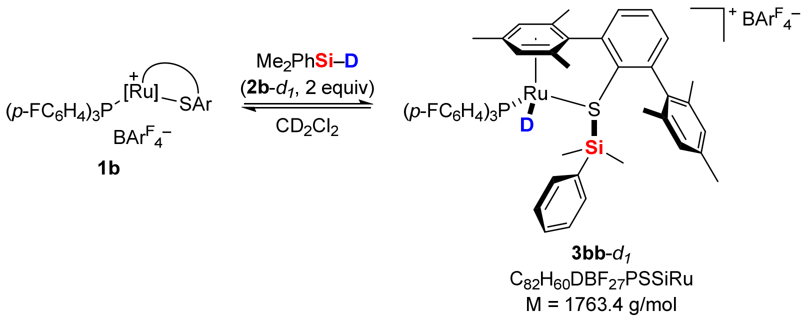

In an NMR tube, deuterium-labeled Me_2PhSiD (**2b**-*d*$_1$, 3.3 mg, 24 μmol, 2.0 equiv) was added to a solution of [{(p-$FC_6H_4)_3P$}Ru(SDmp)]$^+$[$BAr^F{}_4$]$^-$ (**1b**, 20 mg, 12 μmol, 1.0 equiv) in CD_2Cl_2 (0.6 mL). The sample was shaken vigorously and directly subjected to NMR spectroscopic analysis indicating the formation of adduct **3bb**-*d*$_1$ along with unreacted hydrosilane **2b**-*d*$_1$.

*NMR spectroscopic data for **3bb**-d$_1$:*

^{1}H NMR (500 MHz, CD_2Cl_2, 300 K): δ = 0.01 (s, 3H), 0.55 (s, 3H), 1.48 (s, 3H), 1.79 (s, 3H), 2.00 (s, 3H), 2.17 (s, 3H), 2.33 (s, 3H), 2.33 (s, 3H), 4.64 (s, 1H), 6.32 (s, 1H), 6.69 (d, *J* = 6.5 Hz, 2H), 6.76 (s, 1H), 6.89 (s, 1H), 7.08 (dd, *J* = 8.5 Hz, *J* = 8.5 Hz, 6H), 7.17 (dd, *J* = 6.7 Hz, *J* = 6.7 Hz, 2H), 7.31−7.38 (m, 8H), 7.57 (d, *J* = 8.0 Hz, 1H), 7.61 (s, 4H*), 7.66 (dd, *J* = 7.6 Hz, *J* = 7.6 Hz, 1H), 7.78 (s, 8H*) ppm. **^{11}B NMR** (161 MHz, CD_2Cl_2, 300 K): δ = −6.6 ppm. **^{19}F{^{1}H} NMR** (471 MHz, CD_2Cl_2, 300 K): δ = −108.7, −62.9 ppm. **^{31}P{^{1}H} NMR** (203 MHz, CD_2Cl_2, 300 K): δ = 48.7 ppm. **^{1}H,^{29}Si HMQC NMR** (500/99 MHz, CD_2Cl_2, 300 K, optimized for *J* = 8 Hz): δ = 0.01/29.8 ppm.

4.2 ^{2}H-Scrambling Experiment of Deuterium-Labeled Dimethylphenylsilane (2b-*d*$_1$) and Non-Deuterated Methyldiphenylsilane (2a) with Ruthenium(II) Thiolate Complex 1a

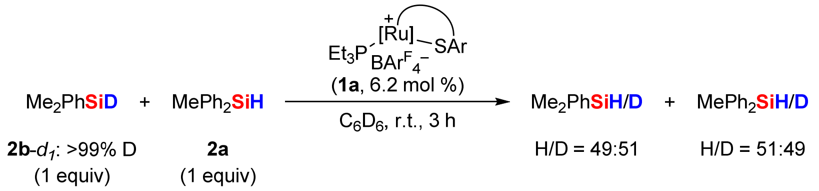

In a 2-mL vial, deuterium-labeled Me_2PhSiD (**2b-*d*$_1$**, 2.8 mg, 20 μmol, 1.0 equiv, >99% D) and non-deuterated $MePh_2SiH$ (**2a**, 4.0 mg, 20 μmol, 1.0 equiv) were added to a solution of $[(Et_3P)Ru(SDmp)]^+[BAr^F{}_4]^-$ (**1a**, 1.7 mg, 1.2 μmol, 6.2 mol %) in C_6D_6 (0.6 mL). The resulting reaction mixture was stirred at room temperature for 3 h. NMR and GLC-MS analysis indicated a H/D ratio of 49:51 for Me_2PhSiH/D corresponding to complete scrambling of isotope labels at the silicon atom. Consistently, for $MePh_2SiH/D$ an isotopic distribution of H/D = 51:49 was observed.

4.3 ^{2}H-Scrambling Experiment of Deuterium-Labeled Dimethylphenylsilane (2b-*d*$_1$) and Non-Deuterated Methyldiphenylsilane (2a) with Ruthenium(II) Thiolate Complex 1b

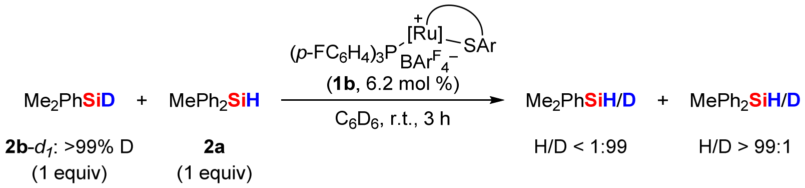

In a 2-mL vial, deuterium-labeled Me_2PhSiD (**2b-*d*$_1$**, 2.8 mg, 20 μmol, 1.0 equiv, >99% D) and non-deuterated $MePh_2SiH$ (**2a**, 4.0 mg, 20 μmol, 1.0 equiv) were added to a solution of $[\{(p\text{-}FC_6H_4)_3P\}Ru(SDmp)]^+[BAr^F{}_4]^-$ (**1b**, 2.0 mg, 1.2 μmol, 6.2 mol %) in C_6D_6 (0.6 mL). The resulting reaction mixture was stirred at room temperature for 3 h. NMR and GLC-MS analysis indicated a H/D ratio of <1:99 for Me_2PhSiH/D corresponding to no scrambling of isotope labels at the silicon atom. Consistently, for $MePh_2SiH/D$ an isotopic distribution of H/D > 99:1 was observed.

5 DFT Calculations

Table S2. Computed NMR chemical shifts (in ppm; ^{1}H and ^{29}Si with respect to TMS, ^{31}P with respect to 85% aq. H_3PO_4) in selected ruthenium(II) thiolate complexes.[a,b]

Compound	R_3P	*Si*	^{31}P NMR			^{1}H NMR			^{29}Si NMR		
			δ_{SR}	δ_{SO}	δ_{4c}	δ_{SR}	δ_{SO}	δ_{4c}	δ_{SR}	δ_{SO}	δ_{4c}
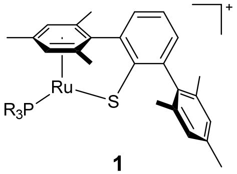 **1**	Me_3P		–4.2	–17.7	–21.9						
	Et_3P		38.4	–15.4	23.0						
	$Ar^F{}_3P$		45.1	–13.5	31.6						
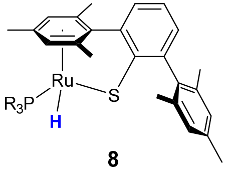 **8**	Me_3P		30.0	–33.0	–3.0	–6.3	–2.7	–9.0			
	Et_3P		61.7	–28.7	33.0	–6.3	–2.8	–9.0			
	$Ar^F{}_3P$		70.9	–24.5	46.4	–6.4	–3.1	–9.5			
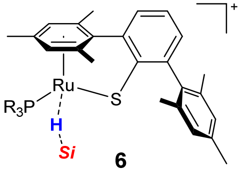 **6**	Me_3P	Me_2PhSi	40.5	–34.8	5.7	–8.9	–2.2	–11.1	17.9	–9.3	8.6
	Et_3P	Me_2PhSi	63.7	–31.5	32.3	–11.3	–1.7	–13.1	11.0	–7.6	3.3
	Et_3P	$MePh_2Si$	66.0	–31.9	34.1	–9.5	–2.0	–11.6	5.1	–9.0	–4.0
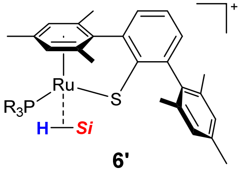 **6'**	Et_3P	$EtMe_2Si$	63.5	–30.7	32.8	–13.1	–1.7	–14.8	29.0	–6.6	22.4
	$Ar^F{}_3P$	Me_2PhSi	58.2	–35.5	22.8	–12.7	–1.5	–14.2	17.2	–5.4	11.7

Compound	R_3P	*Si*	^{31}P NMR			^{1}H NMR			^{29}Si NMR		
			δ_{SR}	δ_{SO}	δ_{4c}	δ_{SR}	δ_{SO}	δ_{4c}	δ_{SR}	δ_{SO}	δ_{4c}
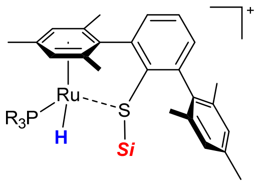 **3**	Me_3P	Me_2PhSi	33.2	–32.0	1.2	–5.3	–2.9	–8.2	37.1	–1.5	35.7
	Et_3P	Me_2PhSi	64.4	–27.7	36.7	–5.0	–2.9	–7.9	34.9	–1.4	33.5
	Et_3P	$MePh_2Si$	64.7	–27.4	37.2	–5.0	–2.8	–7.8	16.4	–1.6	14.8
	Et_3P	$EtMe_2Si$	64.7	–26.6	38.2	–5.0	–2.8	–7.8	45.9	–1.8	44.1
	$Ar^F{}_3P$	Me_2PhSi	66.5	–25.2	41.3	–4.4	–3.1	–7.5	35.0	–1.6	33.3
	$Ar^F{}_3P$	$MePh_2Si$	65.3	–25.7	39.7	–4.6	–3.0	–7.6	29.3	–1.0	28.3
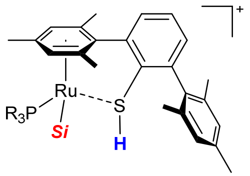 **4**	Et_3P	Me_2PhSi	50.4	–30.1	20.3	4.7	–0.6	4.1	38.2	–17.8	20.3
	$Ar^F{}_3P$	Me_2PhSi	57.3	–30.1	27.2	5.0	–0.7	4.3	40.5	–17.2	23.4

[a]Chemical shifts δ_{4c} calculated at the four-component mDKS level using the PBE functional in conjunction with Dyall's VDZ basis set on Ru and fully uncontracted IGLO-II basis sets on the ligand atoms (cf. Computational Details). Scalar relativistic shifts δ_{SR} were obtained at the same level of theory by scaling spin-orbit integrals to zero. The spin-orbit (SO) contribution to the chemical shift, δ_{SO}, is evaluated as a difference between δ_{4c} and δ_{SR}. [b]$Ar^F{}_3P$ = (p-$FC_6H_4)_3P$.

Table S3. Optimized structural parameters in selected ruthenium(II) thiolate complexes and Gibbs free energies of formation, ΔG_r^0, relative to the sum of the free energies of $[(R_3P)RuSDmp]^+$ ($\mathbf{1}^+$) and the corresponding silane **2**.[a,b]

Compound	R_3P	***Si***	d(Ru···S) [Å]	d(Ru–P) [Å]	d(Ru···H) [Å]	d(Si···H) [Å]	d(Ru···Si) [Å]	d(S···Si) [Å]	α(Ru···H···Si) [°]	ΔG_r^0 [kJ/mol]
	Me_3P		2.239	2.389						
	Et_3P		2.240	2.389						
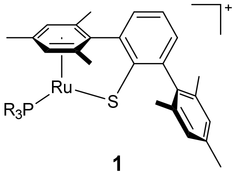 **1**	Ar_3P		2.242	2.383						
	Ph_3P		2.243	2.387						
	$Ar^O{}_3P$		2.242	2.386						
	Me_3P	Me_2PhSi	2.397	2.324	1.618	1.794	2.589	3.259	98.6	–23.0
	Et_3P	Me_2PhSi	2.394	2.333	1.674	1.627	2.851	3.458	119.5	–25.2
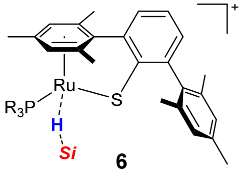 **6**	Et_3P	$MePh_2Si$	2.400	2.338	1.630	1.747	2.641	3.328	102.8	–17.9
	Et_3P	$EtMe_2Si$	2.398	2.339	1.711	1.594	2.966	3.619	127.6	–2.7
	Et_3P	*t*$BuMe_2Si$	2.399	2.340	1.769	1.593	3.078	3.742	132.5	13.2
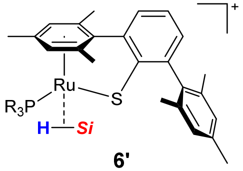 **6'**	$Ar^F{}_3P$	Me_2PhSi	2.391	2.335	1.719	1.570	3.111	3.753	142.1	–15.3
	Ph_3P	Me_2PhSi	2.392	2.338	1.718	1.568	3.105	3.749	141.7	–14.8
	$Ar^O{}_3P$	Me_2PhSi	2.389	2.342	1.723	1.567	3.110	3.743	141.8	–17.2
	$Ar^F{}_3P$	*t*$BuMe_2Si$	2.396	2.337	1.721	1.610	2.994	3.416	127.9	2.8

Compound	R_3P	***Si***	d(Ru···S) [Å]	d(Ru–P) [Å]	d(Ru···H) [Å]	d(Si···H) [Å]	d(Ru···Si) [Å]	d(S···Si) [Å]	α(Ru···H···Si) [°]	ΔG_r^0 [kJ/mol]
	Me_3P	Me_2PhSi	2.373	2.306	1.601	2.249	2.874	2.416	95.1	14.0
	Et_3P	Me_2PhSi	2.374	2.318	1.597	2.201	2.872	2.416	97.0	13.4
	Et_3P	$MePh_2Si$	2.371	2.320	1.603	2.166	2.896	2.428	99.3	6.4
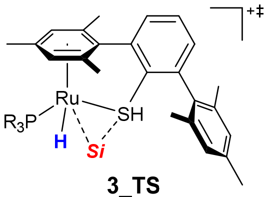	Et_3P	$EtMe_2Si$	2.385	2.316	1.599	2.219	2.895	2.423	97.3	34.0
	Et_3P	*t*BuMe$_2$Si	2.370	2.323	1.607	2.175	2.876	2.481	97.9	56.2
**3_TS**	$Ar^F{}_3P$	Me_2PhSi	2.376	2.322	1.585	2.171	2.914	2.446	100.6	27.9
	Ph_3P	Me_2PhSi	2.374	2.326	1.587	2.168	2.906	2.430	100.3	33.6
	$Ar^O{}_3P$	Me_2PhSi	2.373	2.331	1.584	2.156	2.921	2.434	101.6	24.1
	$Ar^F{}_3P$	*t*BuMe$_2$Si	2.368	2.333	1.605	2.095	2.891	2.545	102.0	74.8
	Me_3P	Me_2PhSi	2.380	2.300	1.605	3.280	3.763	2.253	94.5	–37.3
	Et_3P	Me_2PhSi	2.394	2.307	1.600	3.188	3.729	2.255	96.7	–36.1
	Et_3P	$MePh_2Si$	2.391	2.300	1.593	3.242	3.764	2.261	96.2	–43.6
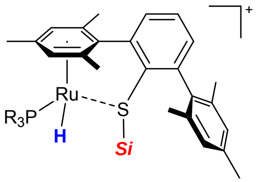	Et_3P	$EtMe_2Si$	2.403	2.306	1.592	3.486	3.919	2.255	93.5	–33.0
	Et_3P	*t*BuMe$_2$Si	2.408	2.303	1.597	3.441	3.844	2.271	92.0	–17.3
**3**	$Ar^F{}_3P$	Me_2PhSi	2.389	2.313	1.582	3.257	3.809	2.266	97.8	–35.6
	Ph_3P	Me_2PhSi	2.396	2.314	1.582	3.255	3.801	2.263	97.6	–26.4
	$Ar^O{}_3P$	Me_2PhSi	2.389	2.319	1.584	3.269	3.788	2.261	96.4	–34.4
	$Ar^F{}_3P$	*t*BuMe$_2$Si	2.389	2.320	1.585	3.356	3.836	2.282	95.1	–4.7

Compound	R_3P	***Si***	d(Ru···S) [Å]	d(Ru–P) [Å]	d(Ru···H) [Å]	d(Si···H) [Å]	d(Ru···Si) [Å]	d(S···Si) [Å]	α(Ru···H···Si) [°]	ΔG_r^0 [kJ/mol]
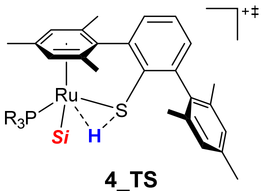 **4_TS**	Et_3P	Me_2PhSi	2.503	2.359	1.982	2.440	2.474	3.594	67.1	48.5
	Et_3P	$MePh_2Si$	2.490	2.340	2.048	2.511	2.474	3.625	64.8	43.9
	$Ar^F{}_3P$	Me_2PhSi	2.498	2.343	1.961	2.487	2.474	3.615	66.4	65.1
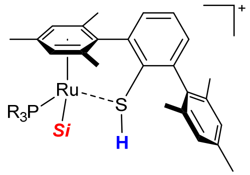 **4**	Et_3P	Me_2PhSi	2.346	2.346	3.052	3.110	2.452	3.341	46.9	14.8
	Et_3P	$MePh_2Si$	2.333	2.348	3.049	3.148	2.445	3.349	46.4	8.9
	$Ar^F{}_3P$	Me_2PhSi	2.330	2.328	3.050	3.197	2.455	3.342	46.2	22.1

[a]Results obtained at the B3LYP-D3(BJ)/ECP/6-31+G** level of theory using an SMD solvation model (cf. Computational Details and Figure 7 for a reaction profile of **1a**$^+$ with Me_2PhSiH). [b]$Ar^F{}_3P$ = (p-$FC_6H_4)_3P$; $Ar^O{}_3P$ = (p-$MeOC_6H_4)_3P$.

Table S4. NPA atomic charges and Wiberg bond indices (WBI) in selected structures and transition states.[a,b]

Compound	R_3P	*Si*	NPA atomic charges				Wiberg bond indices			
			q(Ru)	q(S)	q(Si)	q(H)	Ru···S	Ru···Si	S···Si	Si···H
	Me_3P		0.055	0.099			0.957			
	Et_3P		0.065	0.096			0.940			
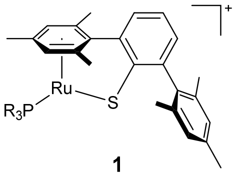 **1**	$Ar^F{}_3P$		0.070	0.123			0.944			
	Ph_3P		0.078	0.120			0.938			
	$Ar^O{}_3P$		0.073	0.122			0.933			
	Me_3P	Me_2PhSi	–0.317	–0.003	1.608	0.028	0.594	0.308	0.096	0.338
	Et_3P	Me_2PhSi	–0.192	–0.027	1.675	–0.098	0.607	0.165	0.046	0.469
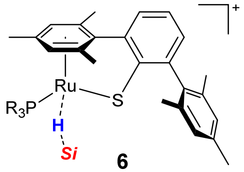 **6**	Et_3P	$MePh_2Si$	–0.281	–0.002	1.652	–0.001	0.596	0.273	0.085	0.373
	Et_3P	$EtMe_2Si$	–0.141	–0.037	1.683	–0.147	0.615	0.131	0.024	0.510
	Et_3P	*t*$BuMe_2Si$	–0.087	–0.037	1.741	–0.182	0.623	0.107	0.017	0.551
	$Ar^F{}_3P$	Me_2PhSi	–0.085	–0.043	1.705	–0.181	0.615	0.101	0.015	0.570
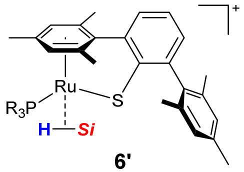 **6'**	Ph_3P	Me_2PhSi	–0.083	–0.042	1.704	–0.180	0.615	0.102	0.015	0.525
	$Ar^O{}_3P$	Me_2PhSi	–0.082	–0.041	1.701	–0.181	0.615	0.102	0.015	0.530
	$Ar^F{}_3P$	*t*$BuMe_2Si$	–0.120	–0.021	1.746	–0.153	0.616	0.122	0.045	0.509

Compound	R_3P	***Si***	NPA atomic charges				Wiberg bond indices			
			q(Ru)	q(S)	q(Si)	q(H)	Ru···S	Ru···Si	S···Si	Si···H
	Me_3P	Me_2PhSi	–0.316	0.087	1.638	0.031	0.533	0.166	0.552	0.122
	Et_3P	Me_2PhSi	–0.308	0.091	1.635	0.034	0.535	0.165	0.554	0.129
	Et_3P	$MePh_2Si$	–0.291	0.087	1.658	0.015	0.537	0.158	0.547	0.146
	Et_3P	$EtMe_2Si$	–0.301	0.085	1.636	0.031	0.531	0.160	0.559	0.128
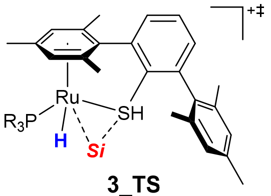 **3_TS**	Et_3P	*t*$BuMe_2Si$	–0.293	0.087	1.689	0.030	0.548	0.173	0.511	0.157
	$Ar^F{}_3P$	Me_2PhSi	–0.291	0.092	1.642	0.051	0.538	0.151	0.536	0.132
	Ph_3P	Me_2PhSi	–0.286	0.093	1.637	0.052	0.538	0.152	0.545	0.133
	$Ar^O{}_3P$	Me_2PhSi	–0.287	0.092	1.641	0.049	0.536	0.149	0.543	0.136
	$Ar^F{}_3P$	*t*$BuMe_2Si$	–0.262	0.086	1.692	0.037	0.562	0.169	0.468	0.178
	Me_3P	Me_2PhSi	–0.261	0.055	1.675	–0.014	0.502	0.020	0.727	0.004
	Et_3P	Me_2PhSi	–0.254	0.055	1.672	–0.003	0.495	0.021	0.733	0.005
	Et_3P	$MePh_2Si$	–0.258	0.052	1.696	–0.002	0.492	0.022	0.727	0.005
	Et_3P	$EtMe_2Si$	–0.252	0.035	1.668	0.007	0.485	0.016	0.721	0.002
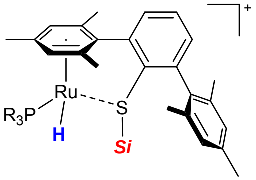 **3**	Et_3P	*t*$BuMe_2Si$	–0.243	0.027	1.743	0.000	0.483	0.017	0.723	0.003
	$Ar^F{}_3P$	Me_2PhSi	–0.247	0.065	1.673	0.026	0.504	0.017	0.716	0.002
	Ph_3P	Me_2PhSi	–0.244	0.062	1.672	0.028	0.500	0.017	0.725	0.002
	$Ar^O{}_3P$	Me_2PhSi	–0.247	0.065	1.668	0.024	0.499	0.018	0.729	0.002
	$Ar^F{}_3P$	*t*$BuMe_2Si$	–0.239	0.058	1.747	0.025	0.502	0.017	0.711	0.003

Compound	R_3P	*Si*	NPA atomic charges				Wiberg bond indices			
			q(Ru)	q(S)	q(Si)	q(H)	Ru···S	Ru···Si	S···Si	Si···H
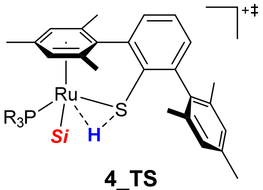 **4_TS**	Et_3P	Me_2PhSi	–0.328	0.205	1.437	0.271	0.426	0.585	0.022	0.043
	Et_3P	$MePh_2Si$	–0.327	0.213	1.453	0.272	0.441	0.581	0.024	0.038
	$Ar^F{}_3P$	Me_2PhSi	–0.333	0.195	1.453	0.197	0.429	0.577	0.022	0.040
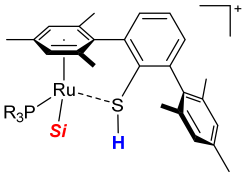 **4**	Et_3P	Me_2PhSi	–0.366	0.312	1.446	0.269	0.530	0.603	0.065	0.003
	Et_3P	$MePh_2Si$	–0.362	0.317	1.471	0.272	0.540	0.599	0.067	0.003
	$Ar^F{}_3P$	Me_2PhSi	–0.371	0.299	1.458	0.184	0.540	0.591	0.067	0.003
$MePh_2SiH$					1.469	–0.197				0.915
Me_2PhSiH					1.487	–0.206				0.911
$EtMe_2SiH$					1.466	–0.212				0.909
$Me_2tBuSiH$					1.513	–0.211				0.907

[a]Results obtained at the B3LYP-D3(BJ)/ECP/6-31+G** level of theory using an SMD solvation model (cf. Computational Details). [b]$Ar^F{}_3P$ = (*p*-$FC_6H_4)_3P$; $Ar^O{}_3P$ = (*p*-$MeOC_6H_4$)P.

6 NMR Spectra

6.1 NMR Spectra of Hydrosilane Adducts 3aa–3ad, 3ba–3bc, and 3be

[(Et$_3$P)Ru(SDmp)·MePh$_2$SiH]$^+$[BAr$^F{}_4$]$^-$ (**3aa**)

^{1}H NMR (500 MHz, CD$_2$Cl$_2$, 250 K): * = MePh$_2$SiH

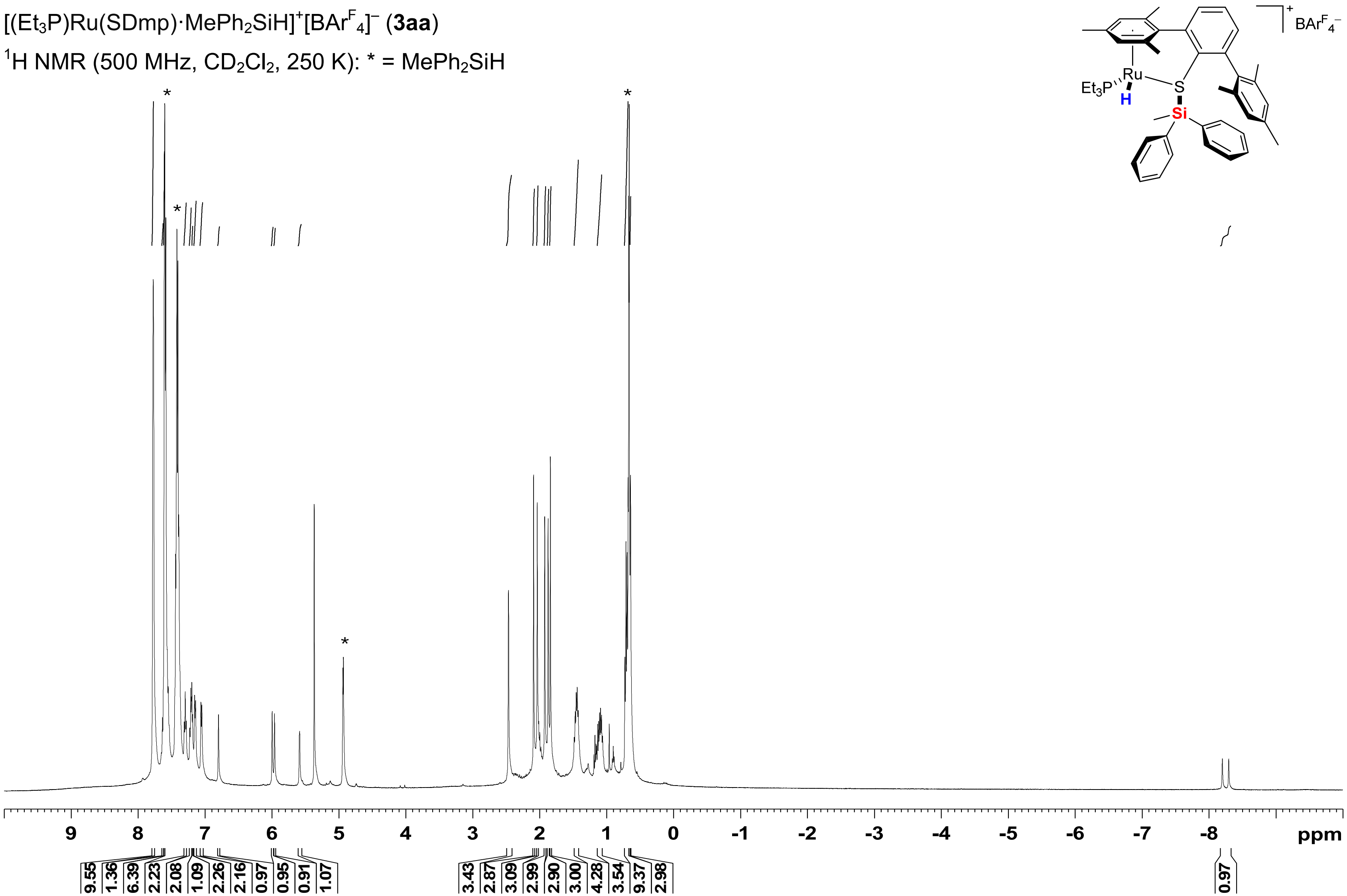

^{11}B NMR (161 MHz, CD_2Cl_2, 250 K):

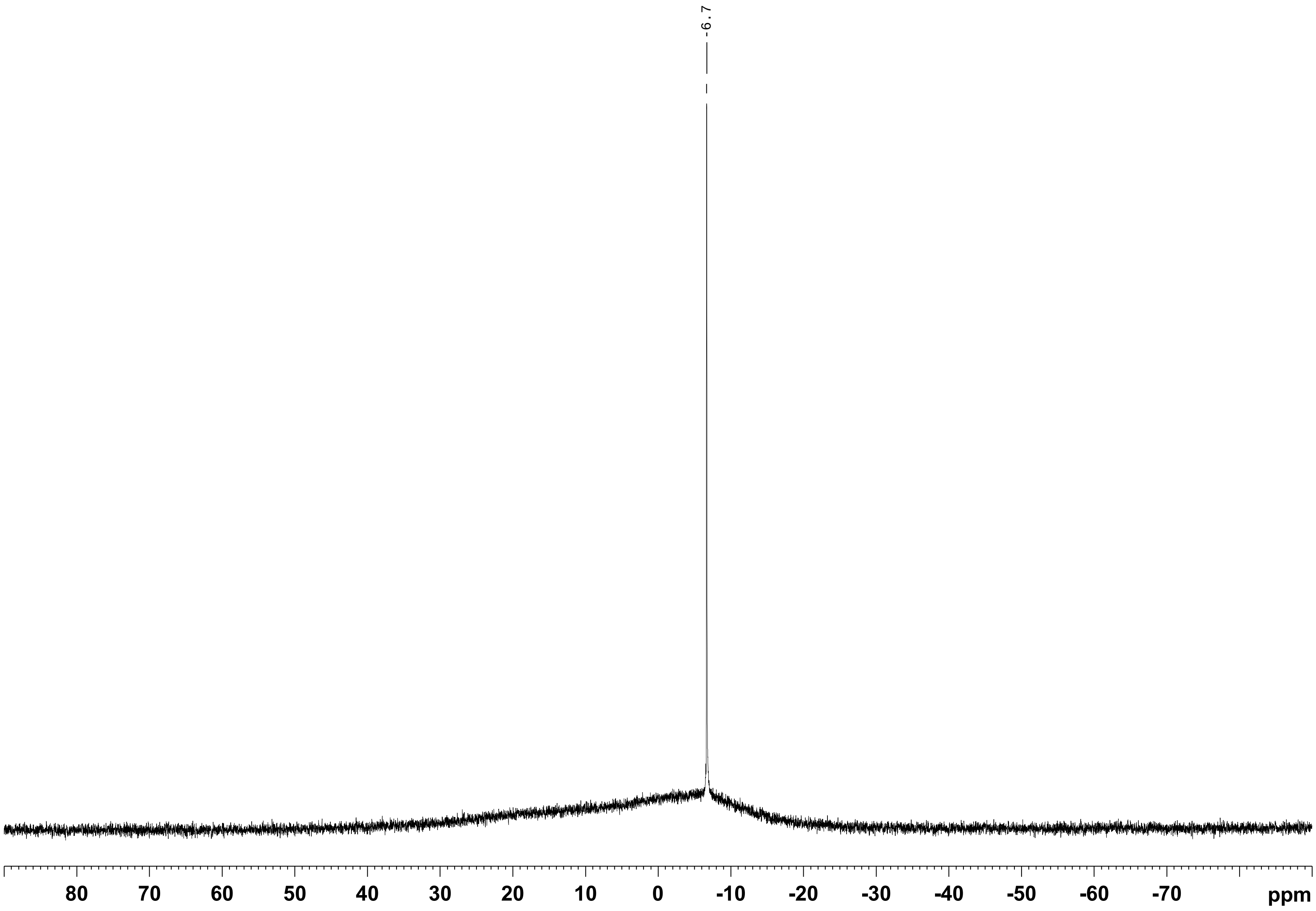

$^{19}F\{^1H\}$ NMR (471 MHz, CD_2Cl_2, 250 K):

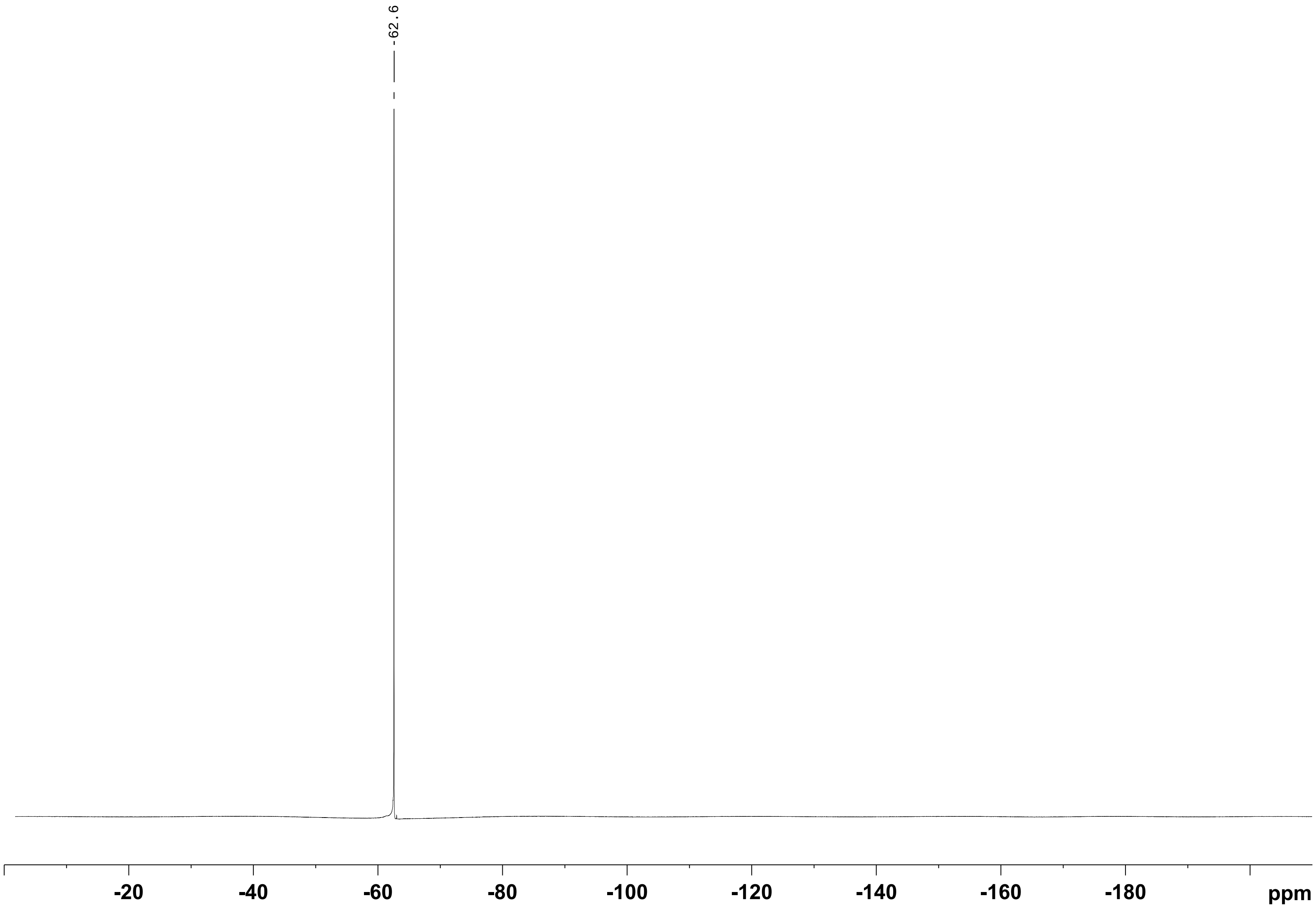

$^{31}P\{^1H\}$ NMR (203 MHz, CD_2Cl_2, 250 K): # = $[Et_3POSiMePh_2]^+[BAr^F{}_4]^-$

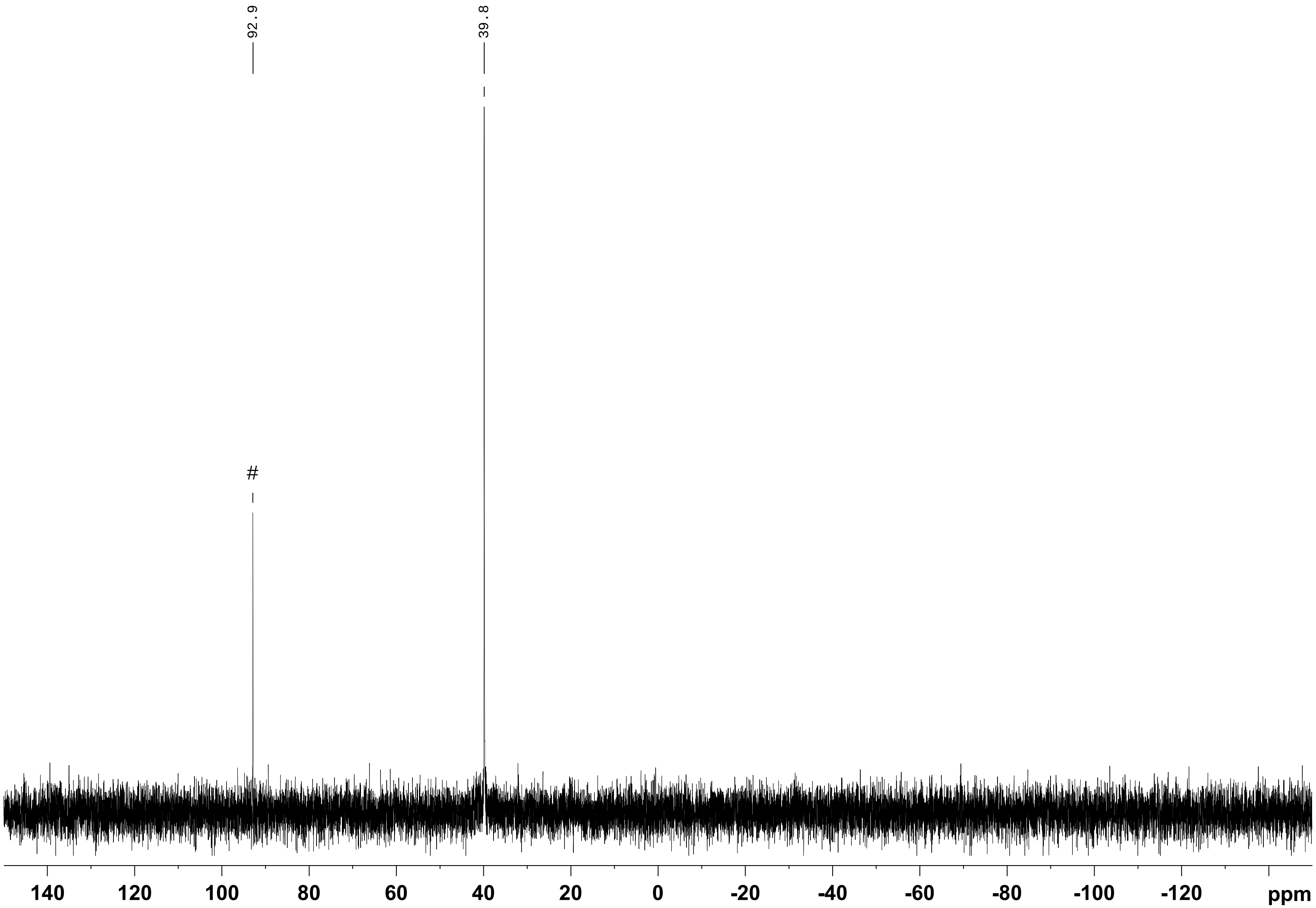

$^{1}H,^{29}Si$ HMQC NMR (500/99 MHz, CD_2Cl_2, 250 K, optimized for J = 8 Hz): * = $MePh_2SiH$, # = $[Et_3POSiMePh_2]^+[BAr^F{}_4]^-$

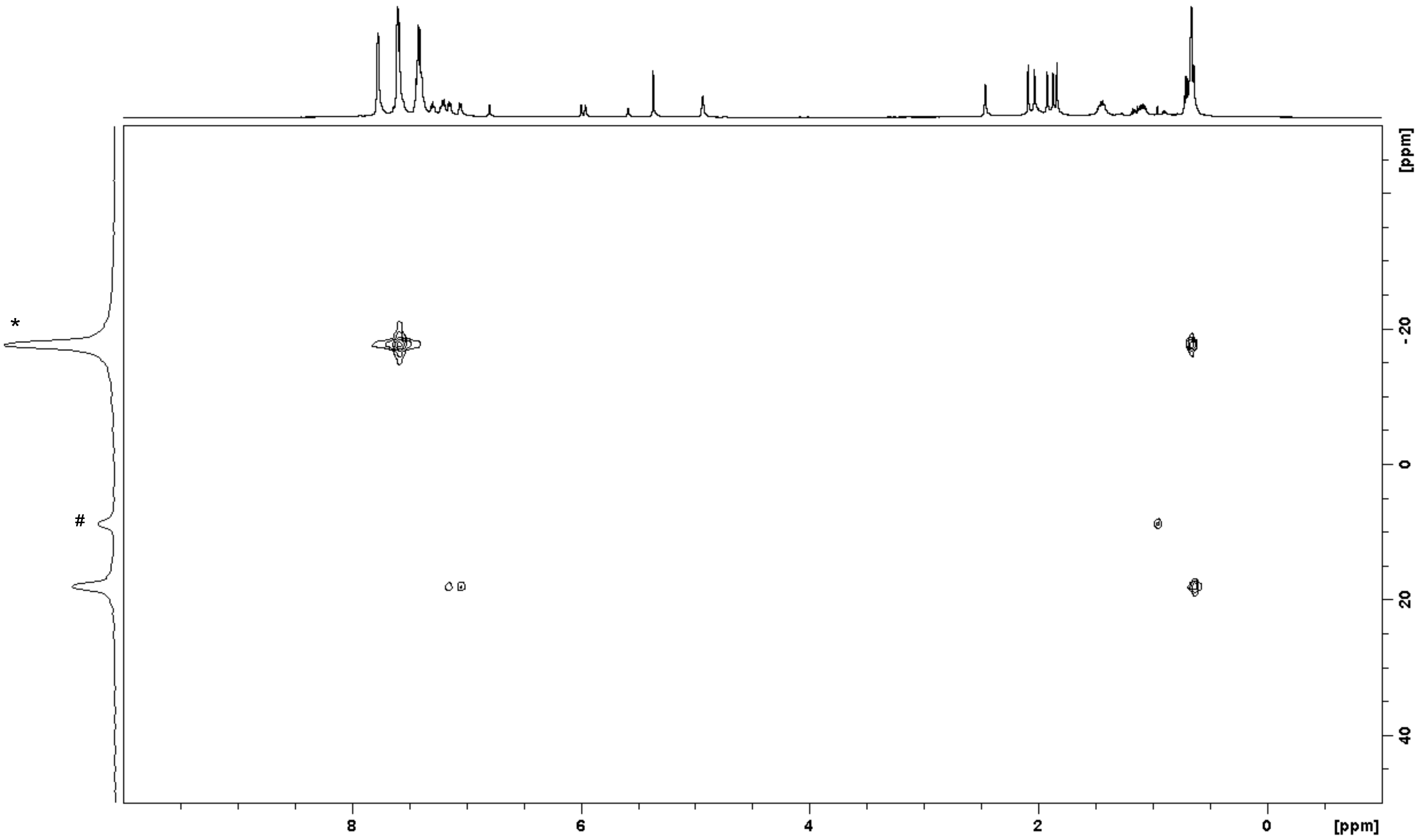

$^{1}H,^{31}P$ HMQC NMR (500/203 MHz, CD_2Cl_2, 300 K, optimized for J = 7 Hz): # = $[Et_3POSiMePh_2]^+[BAr^F{}_4]^-$

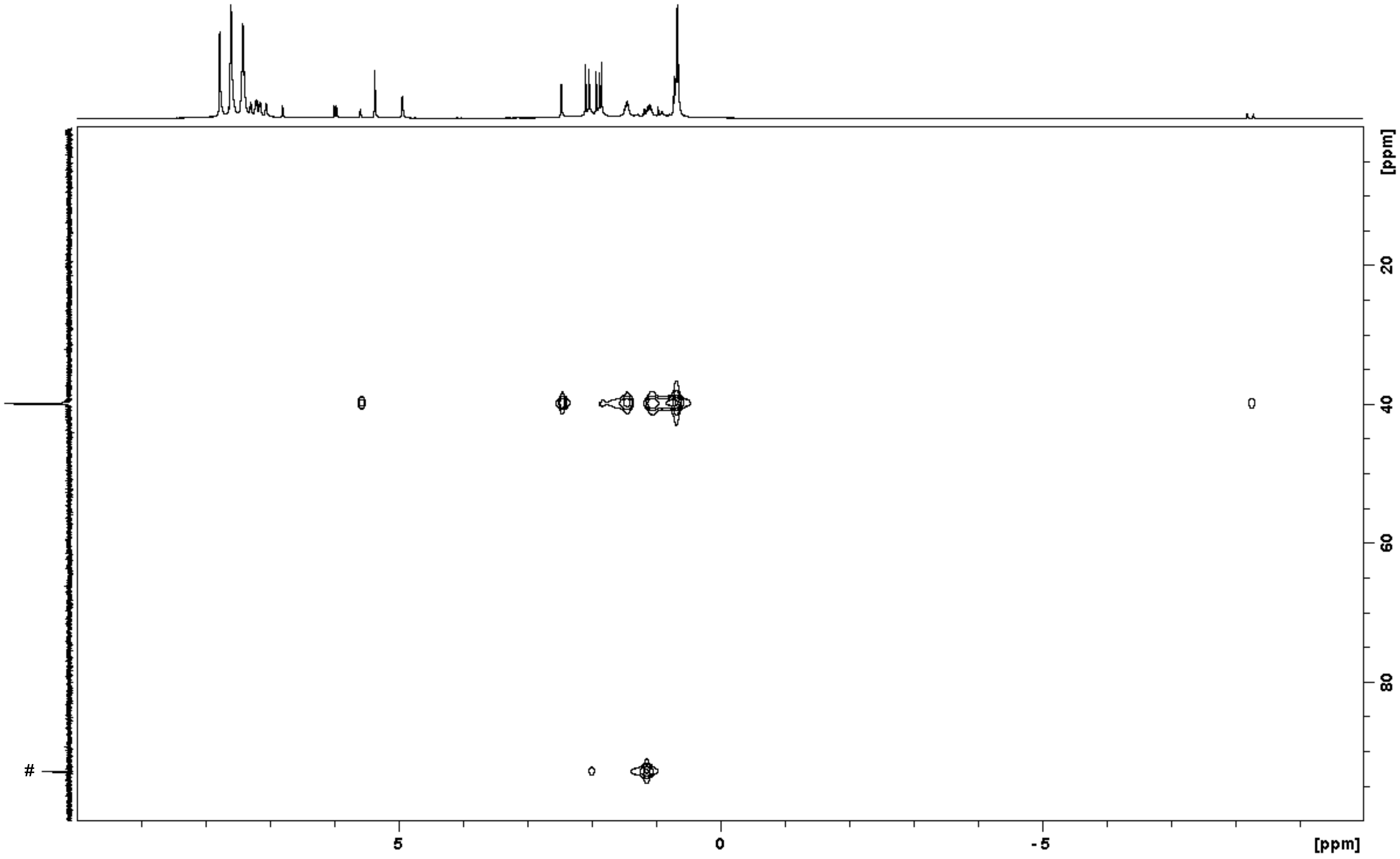

[(Et$_3$P)Ru(SDmp)·Me$_2$PhSiH]$^+$[BAr^F$_4$]$^-$ (**3ab**)

^{1}H NMR (500 MHz, CD$_2$Cl$_2$, 250 K): * = Me$_2$PhSiH

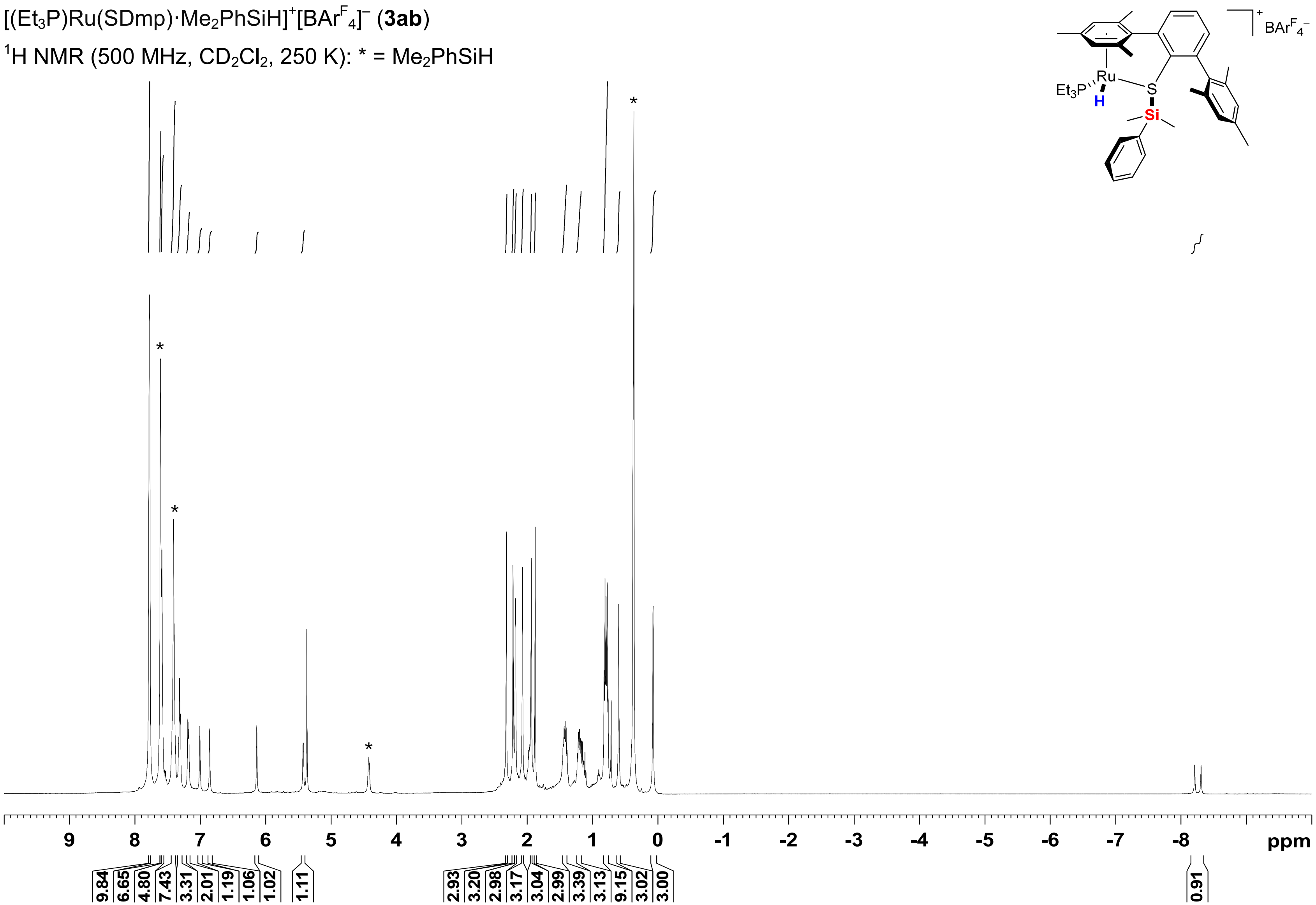

^{11}B NMR (161 MHz, CD_2Cl_2, 250 K):

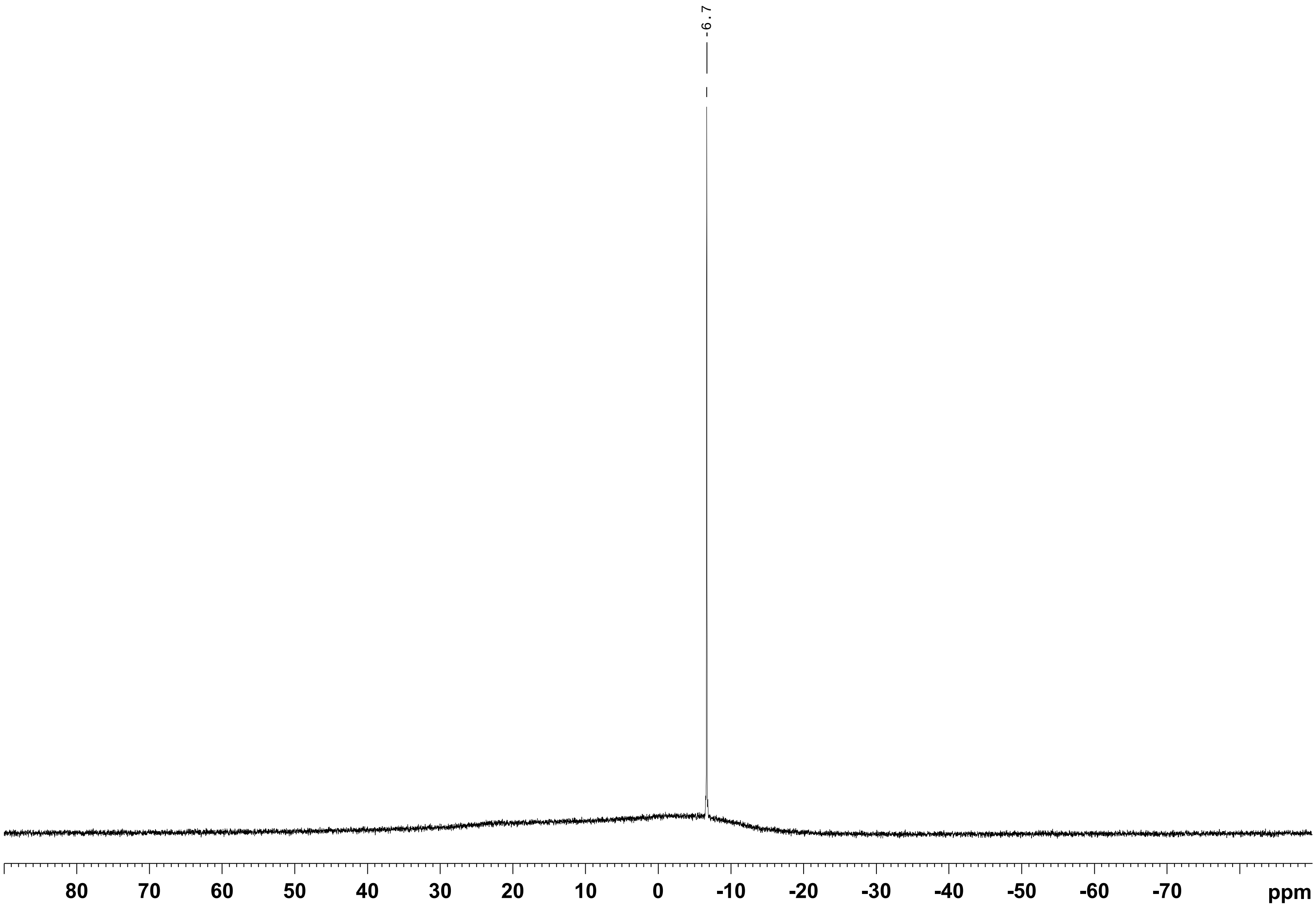

$^{19}F\{^{1}H\}$ NMR (471 MHz, CD_2Cl_2, 250 K):

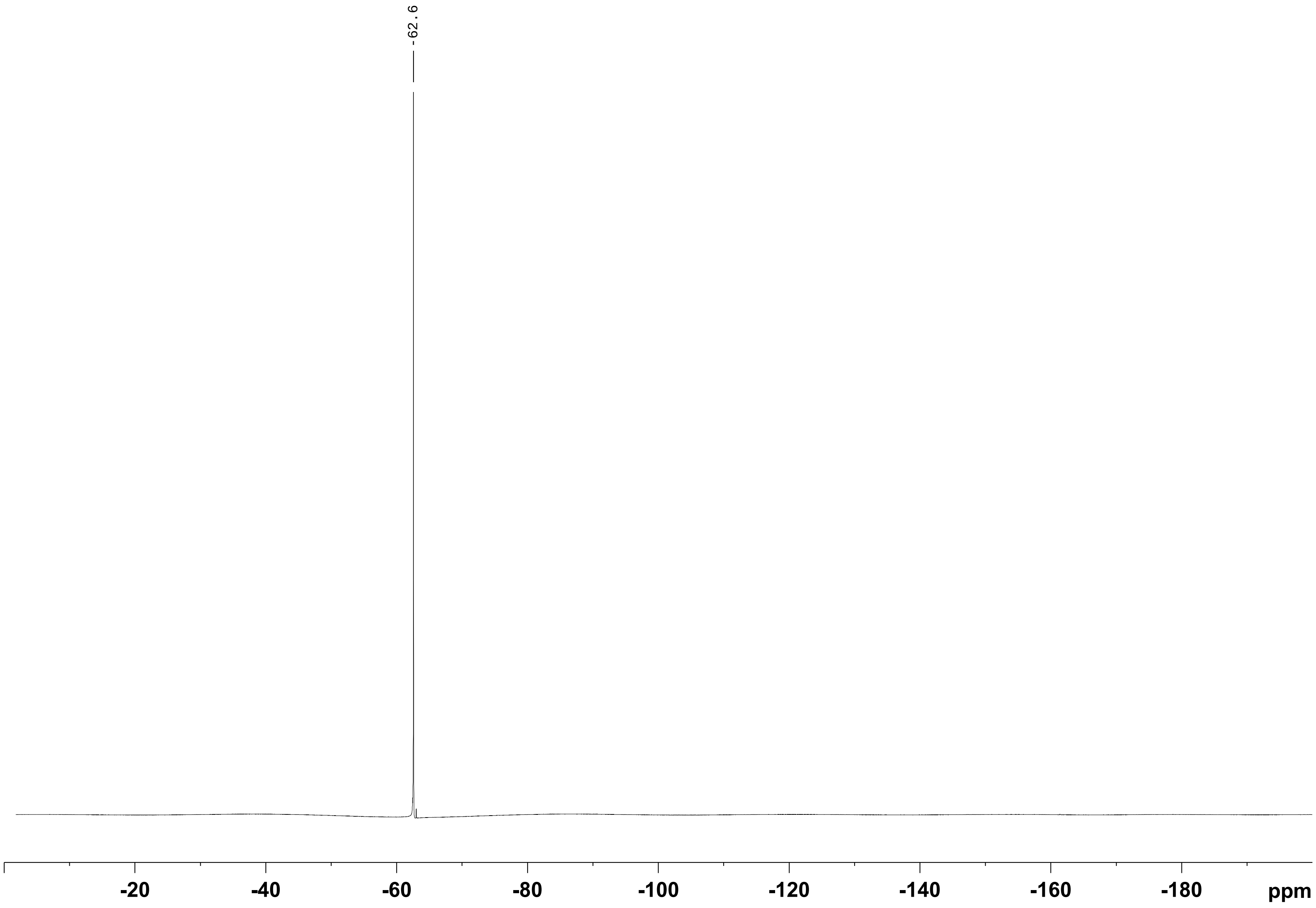

$^{31}P\{^{1}H\}$ NMR (203 MHz, CD_2Cl_2, 250 K): # = $[Et_3POSiMe_2Ph]^{+}[BAr^{F}_{4}]^{-}$

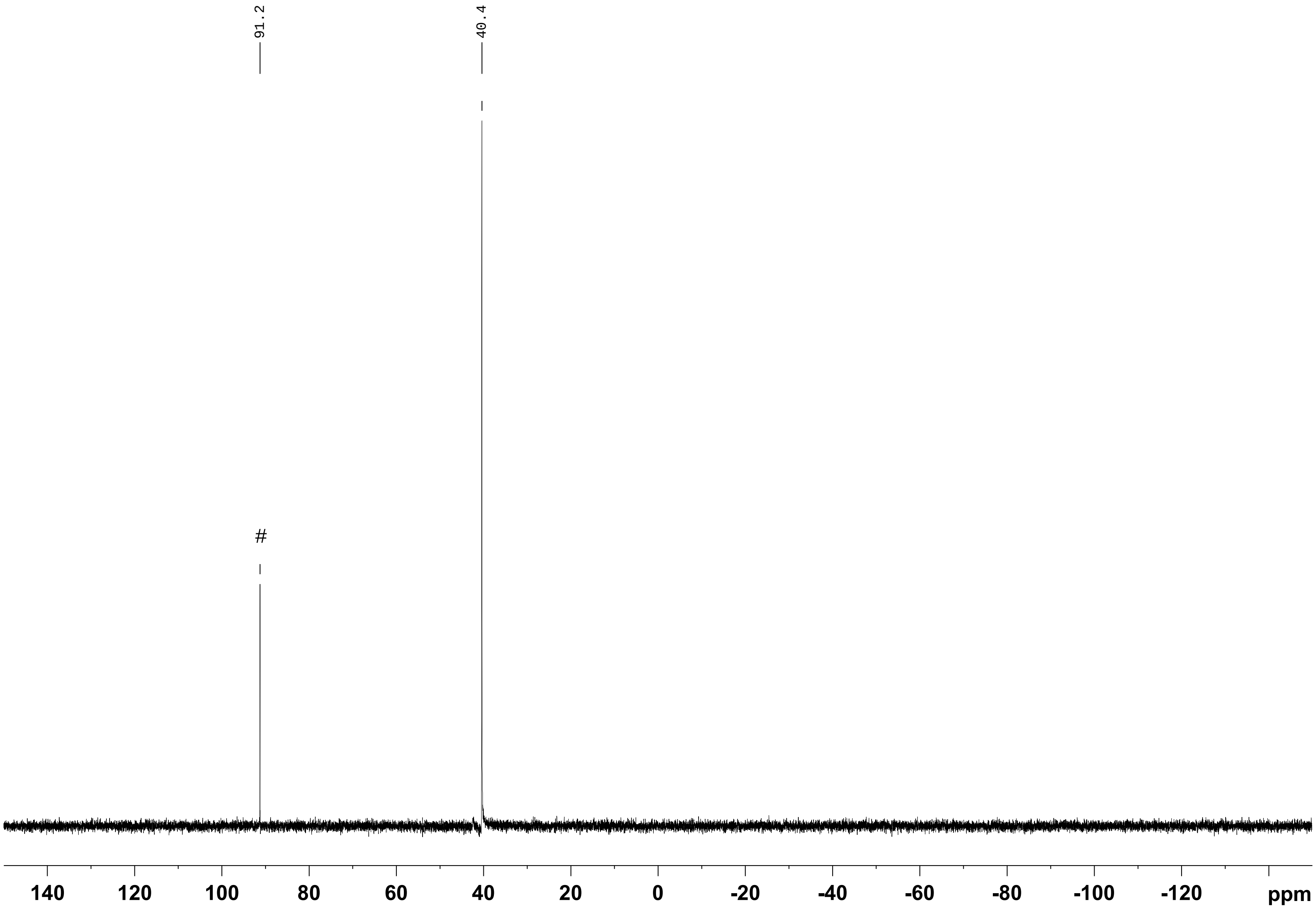

$^{1}H,^{29}Si$ HMQC NMR (500/99 MHz, CD_2Cl_2, 250 K, optimized for J = 8 Hz): * = Me_2PhSiH, # = $[Et_3POSiMe_2Ph]^+[BAr^F{}_4]^-$

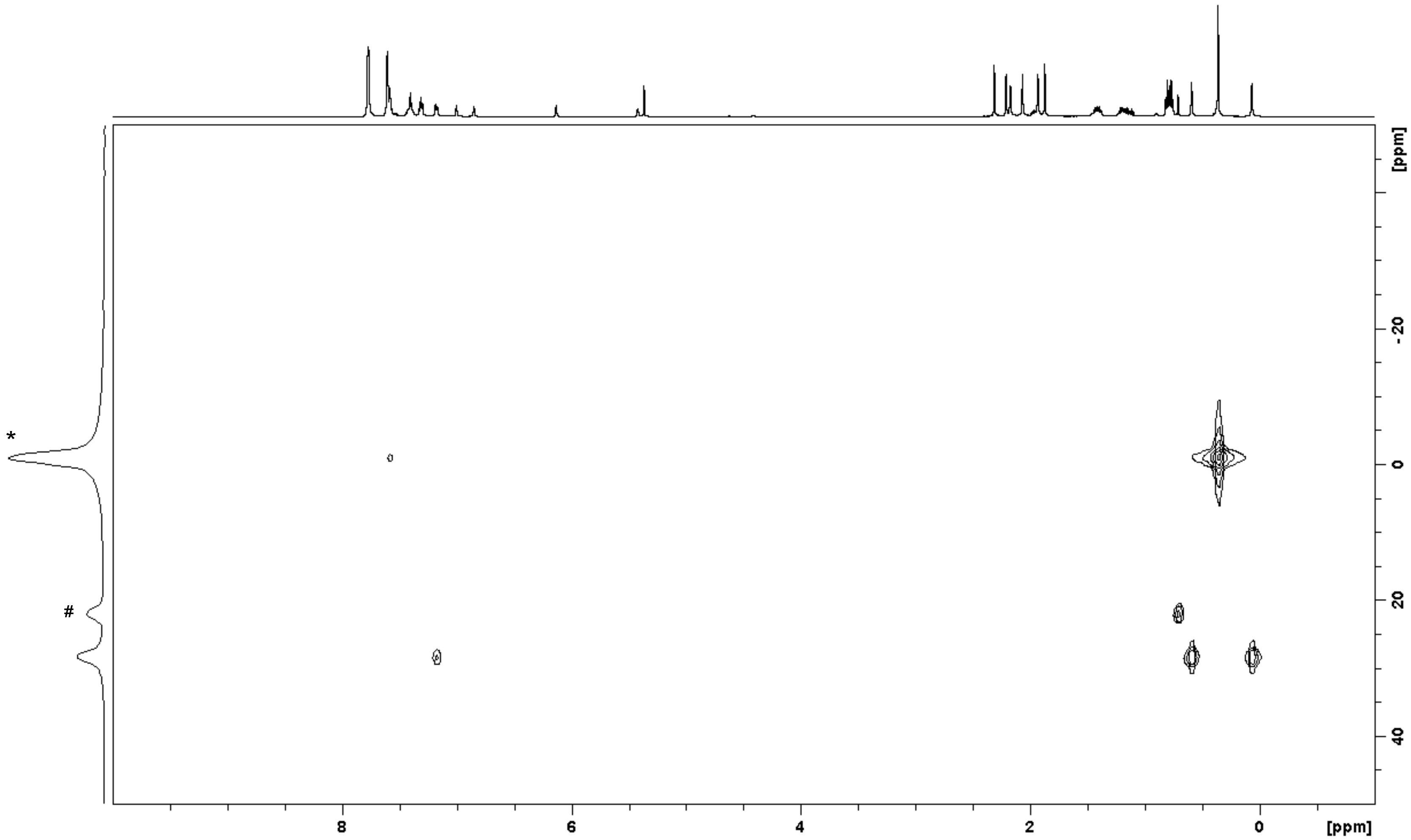

$^{1}H,^{31}P$ HMQC NMR (500/203 MHz, CD_2Cl_2, 250 K, optimized for J = 7 Hz): # = $[Et_3POSiMe_2Ph]^{+}[BAr^{F}{}_4]^{-}$

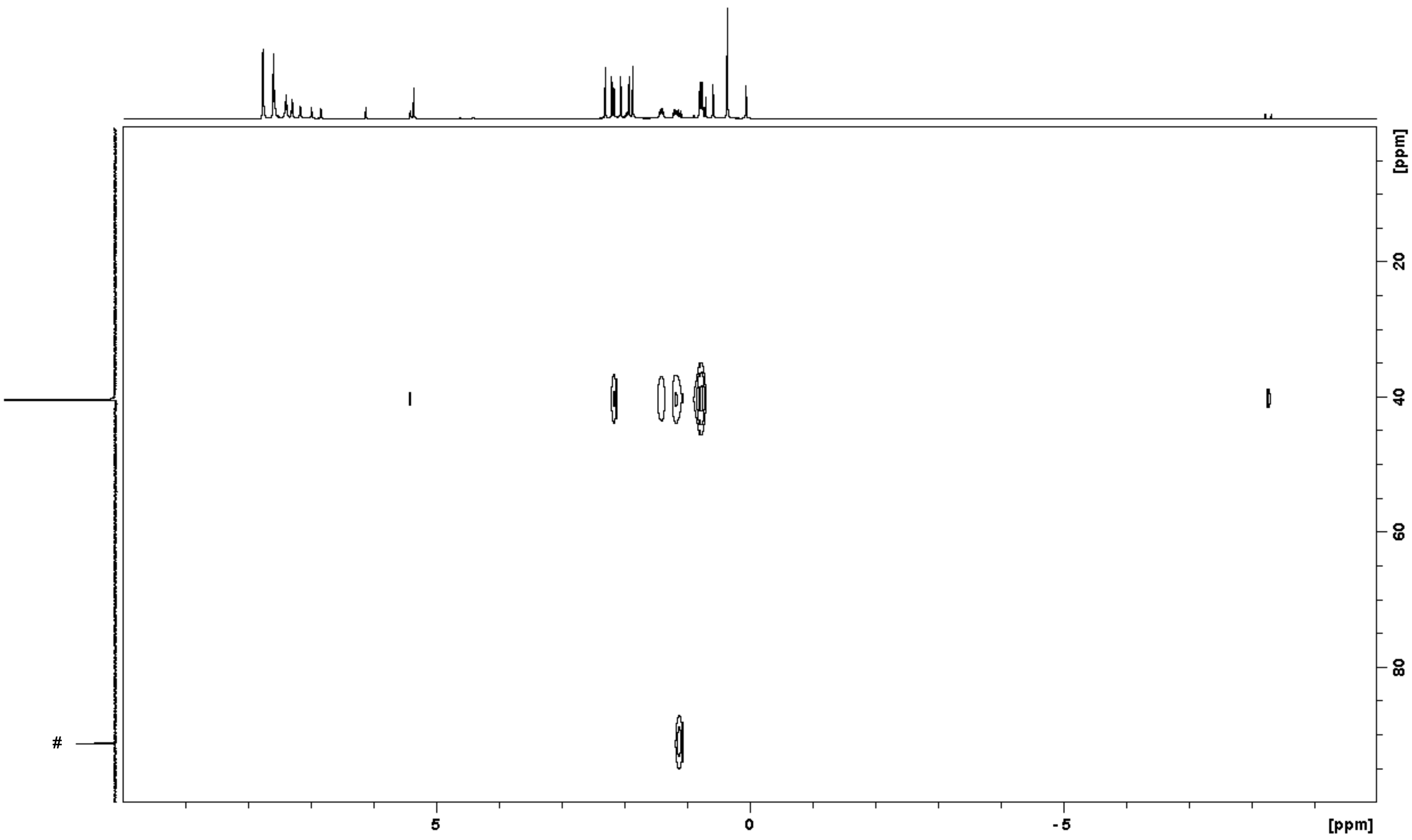

[(Et$_3$P)Ru(SDmp)·Et$_3$SiH]$^+$[BAr$^F{}_4$]$^-$ (**3ac**)

^{1}H NMR (500 MHz, CD_2Cl_2, 300 K): * = Et$_3$SiH

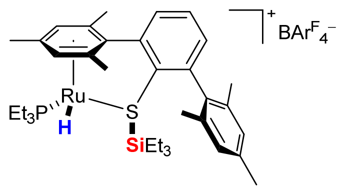

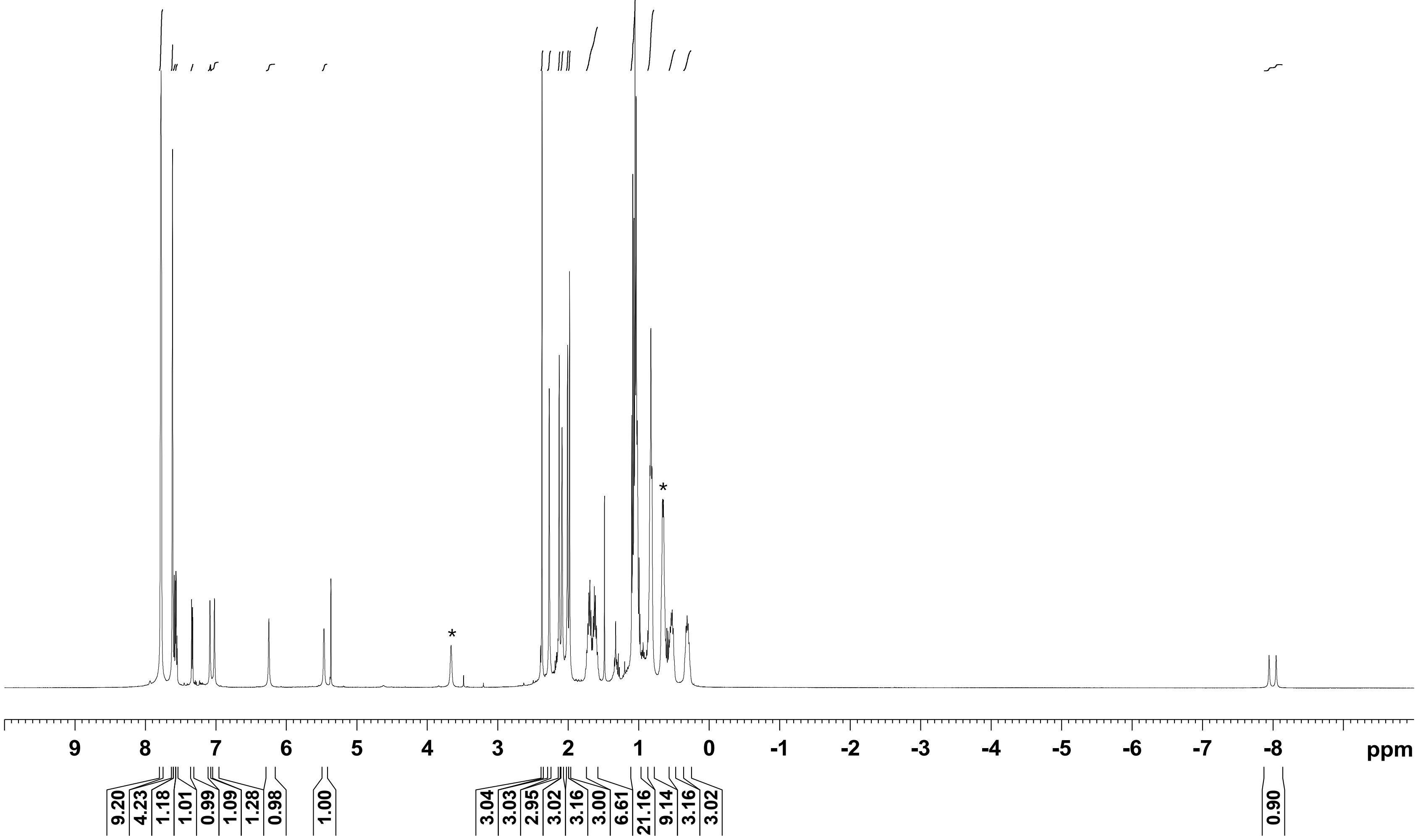

^{11}B NMR (161 MHz, CD_2Cl_2, 300 K):

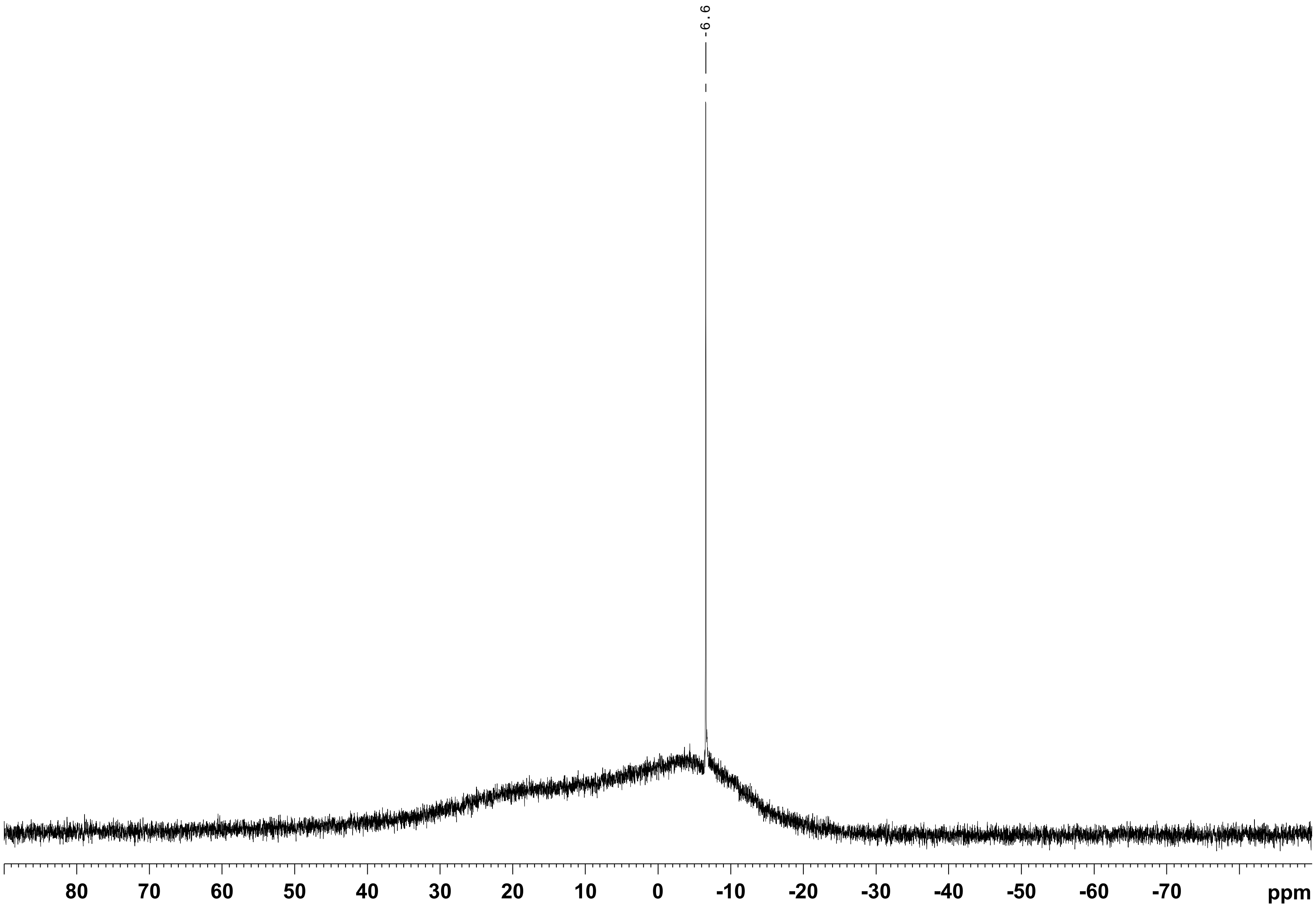

$^{19}F\{^1H\}$ NMR (471 MHz, CD_2Cl_2, 300 K):

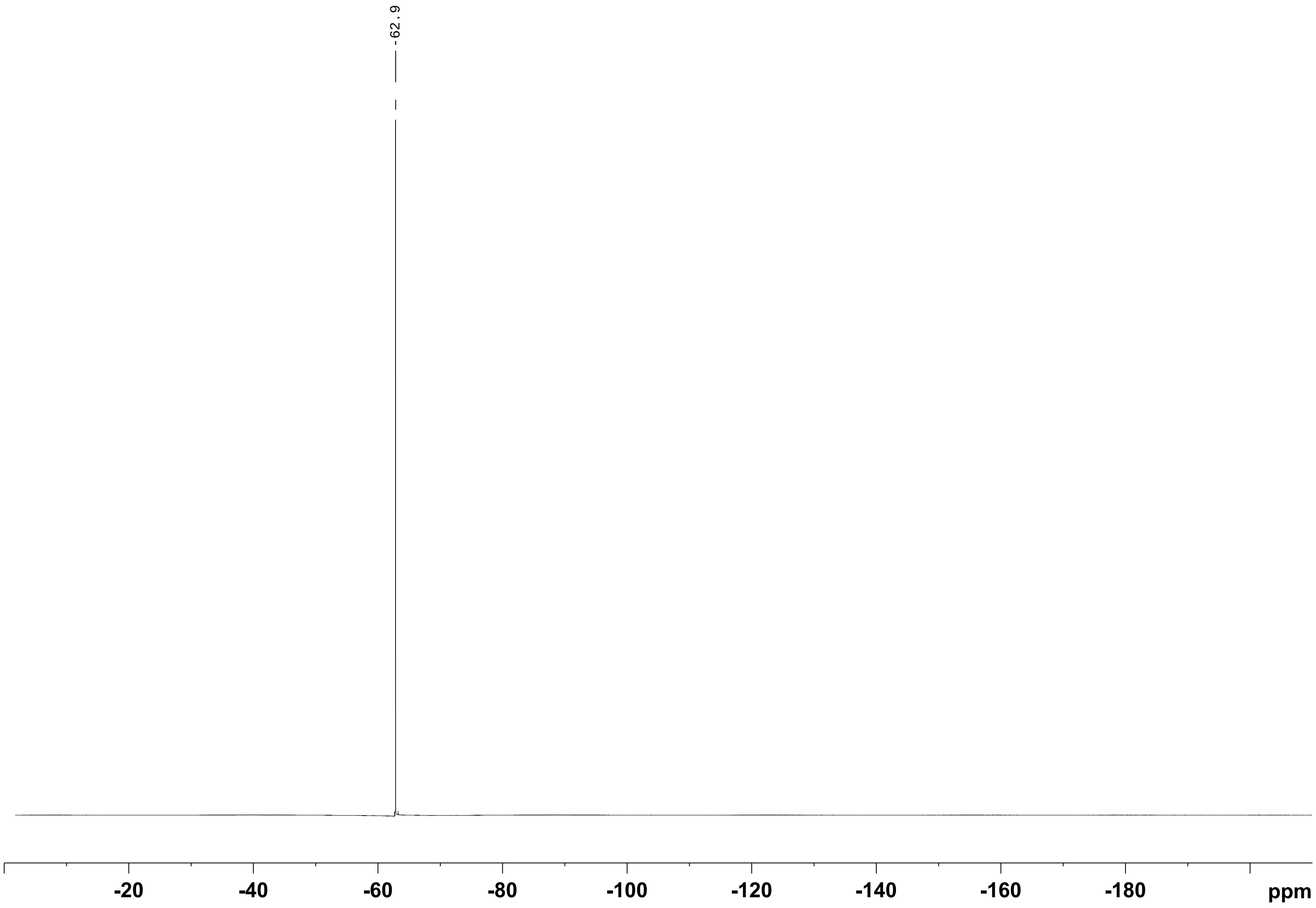

$^{31}P\{^1H\}$ NMR (203 MHz, CD_2Cl_2, 300 K): # = $[Et_3POSiEt_3]^+[BAr^F{}_4]^-$

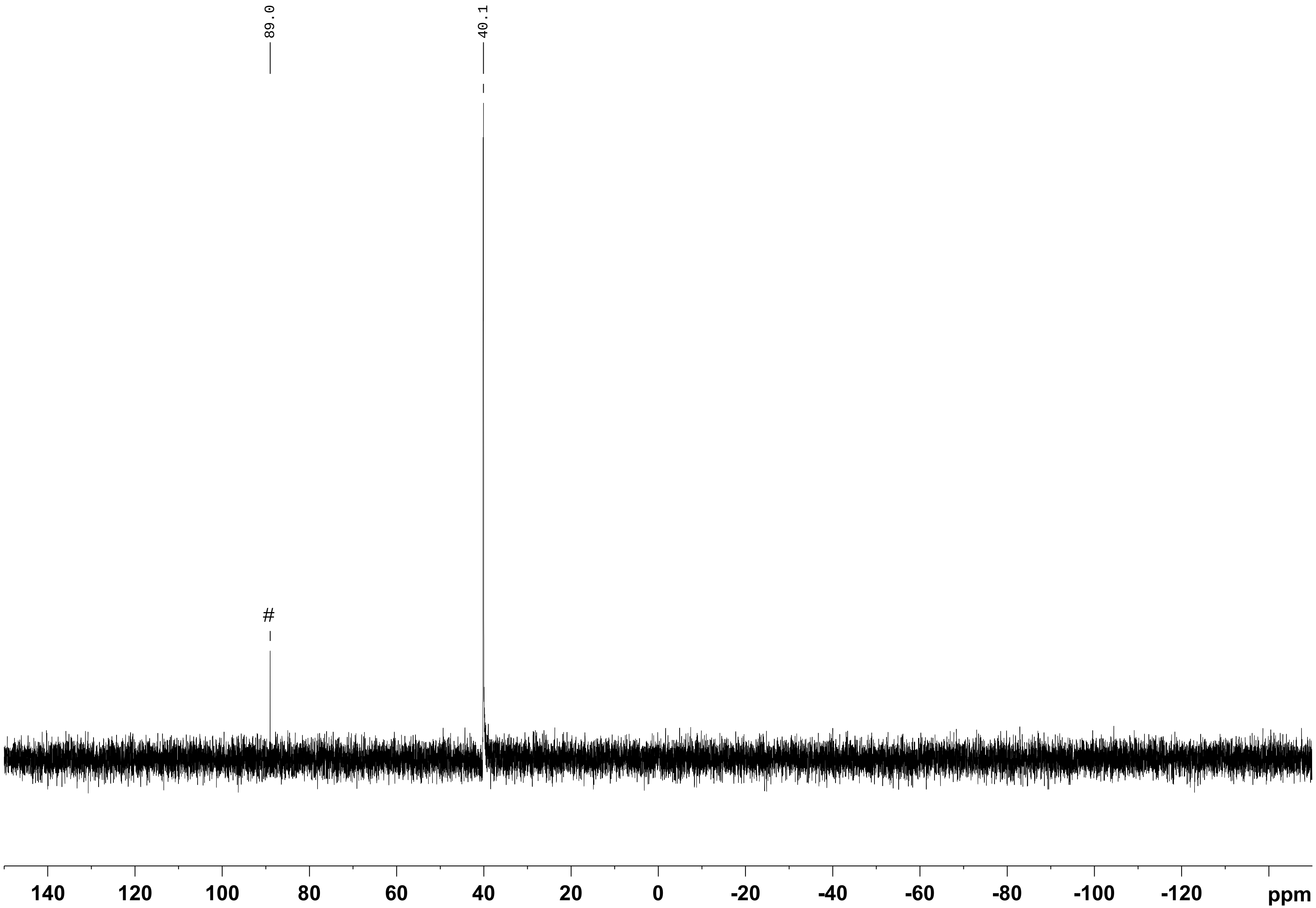

$^{1}H,^{29}Si$ HMQC NMR (500/99 MHz, CD_2Cl_2, 300 K, optimized for J = 8 Hz): * = Et_3SiH, # = $[Et_3POSiEt_3]^+[BAr^F{}_4]^-$

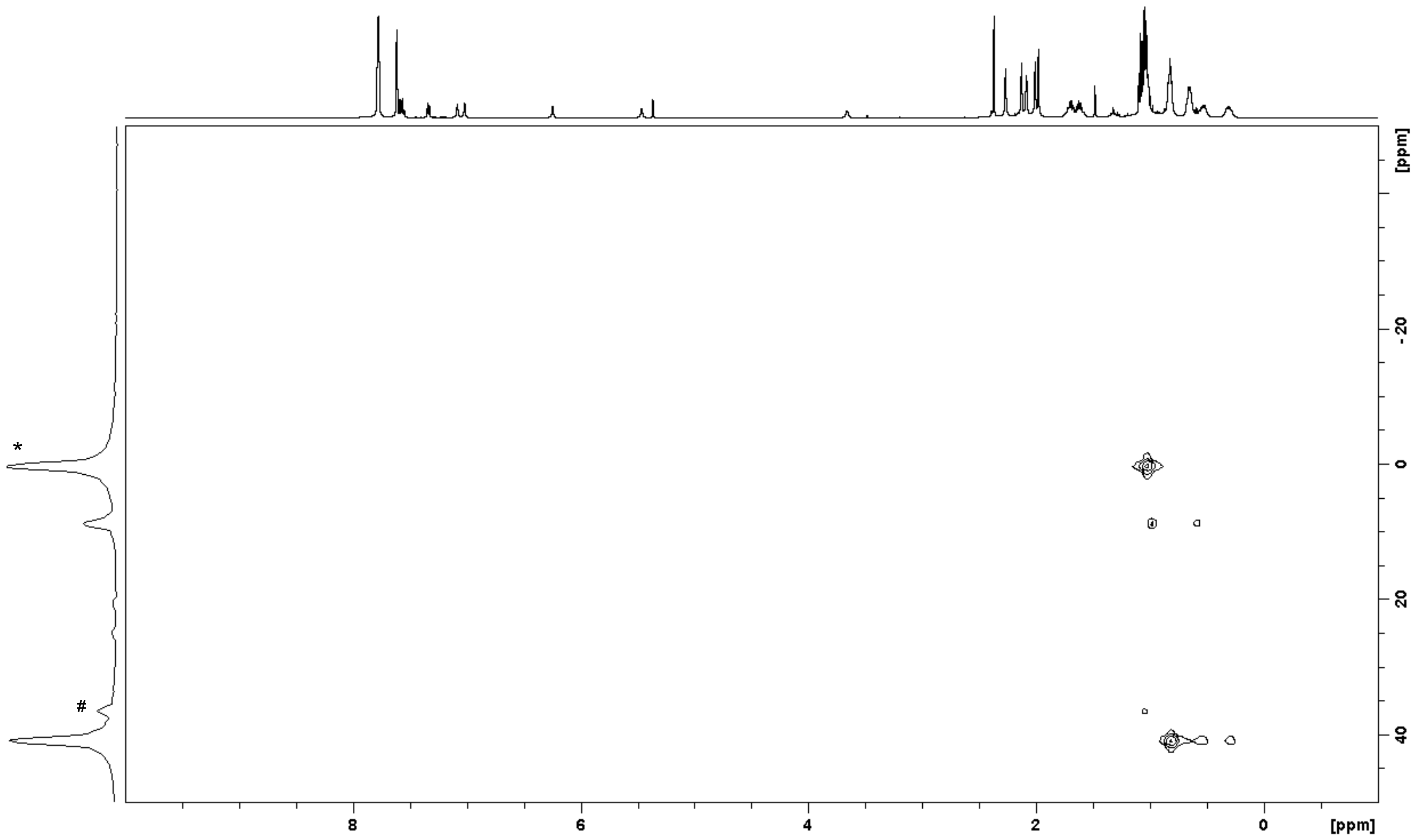

$^{1}H,^{31}P$ HMQC NMR (500/203 MHz, CD_2Cl_2, 300 K, optimized for J = 7 Hz): # = $[Et_3POSiEt_3]^+[BAr^F{}_4]^-$

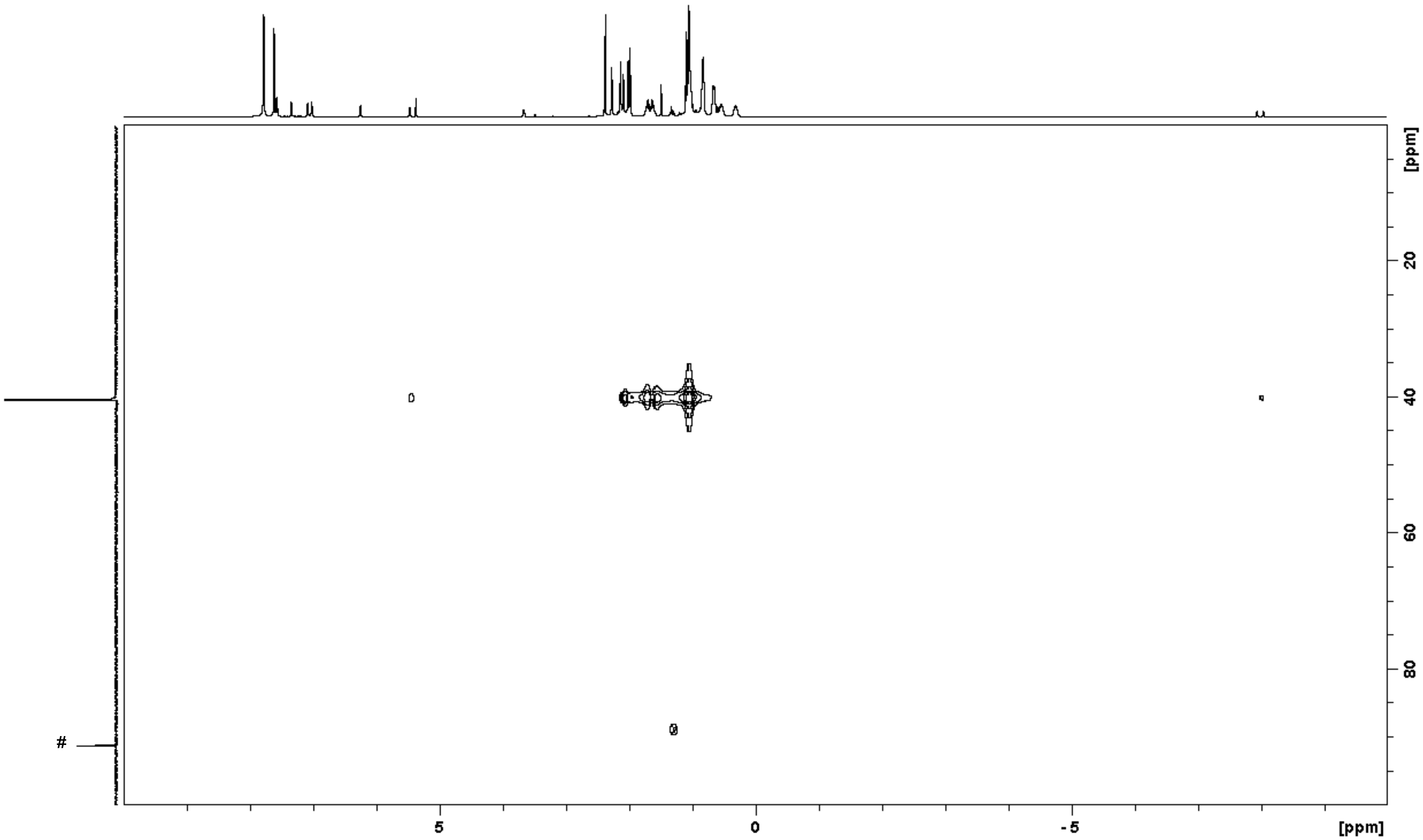

[(Et$_3$P)Ru(SDmp)·EtMe$_2$SiH]$^+$[BAr$^F_4$]$^-$ (**3ad**)

^{1}H NMR (500 MHz, CD_2Cl_2, 300 K): * = EtMe$_2$SiH

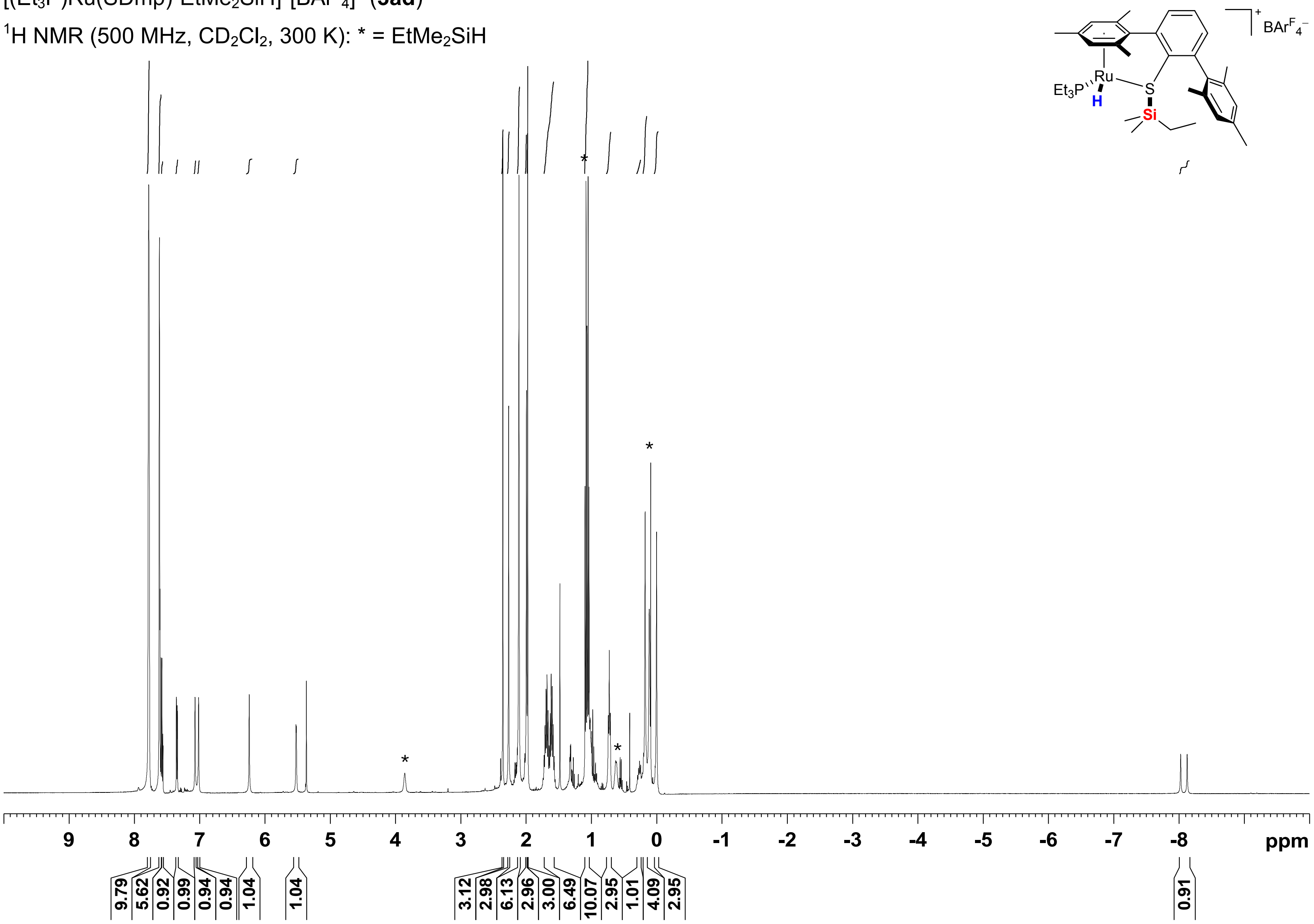

^{11}B NMR (161 MHz, CD_2Cl_2, 300 K):

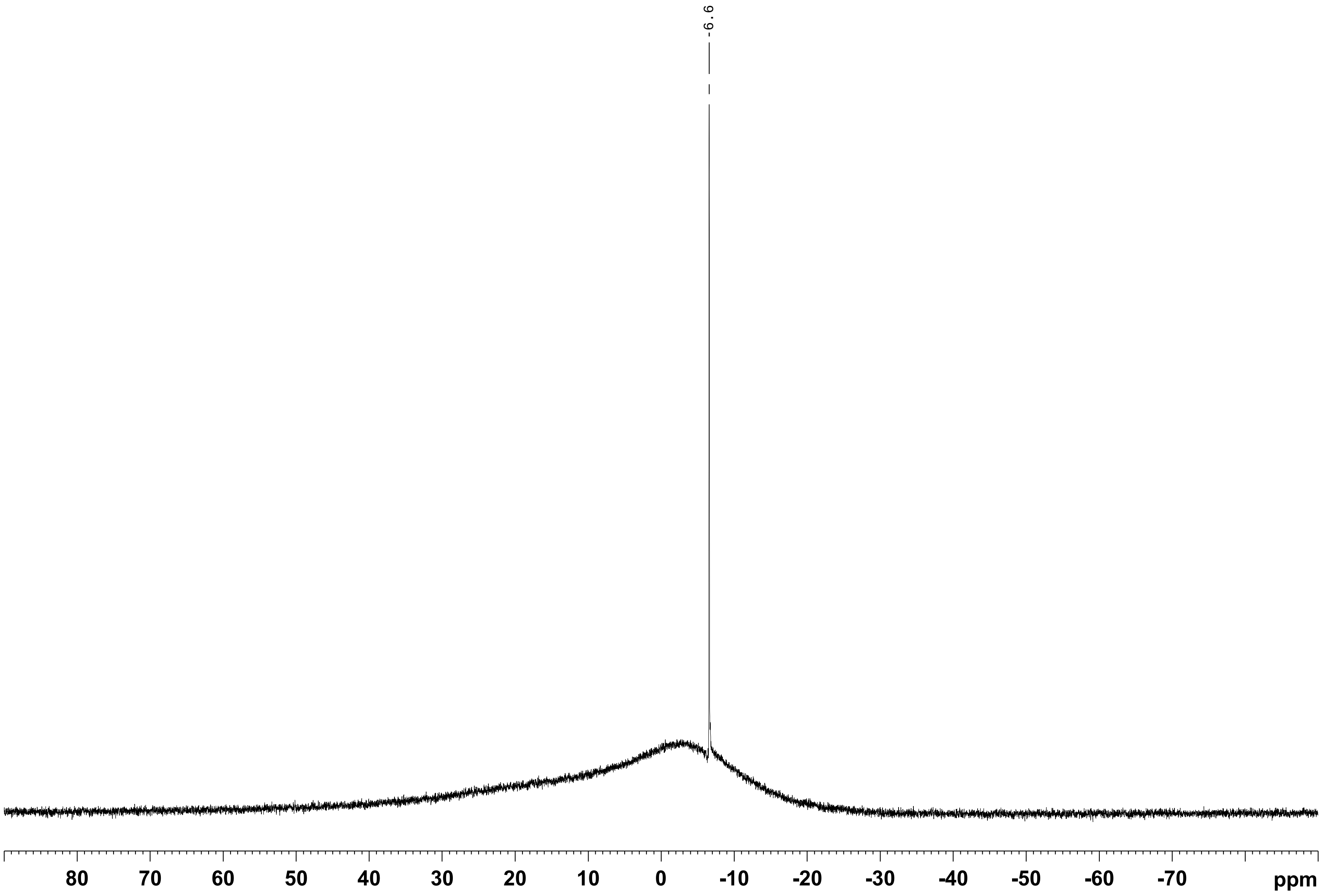

$^{19}F\{^{1}H\}$ NMR (471 MHz, CD_2Cl_2, 300 K):

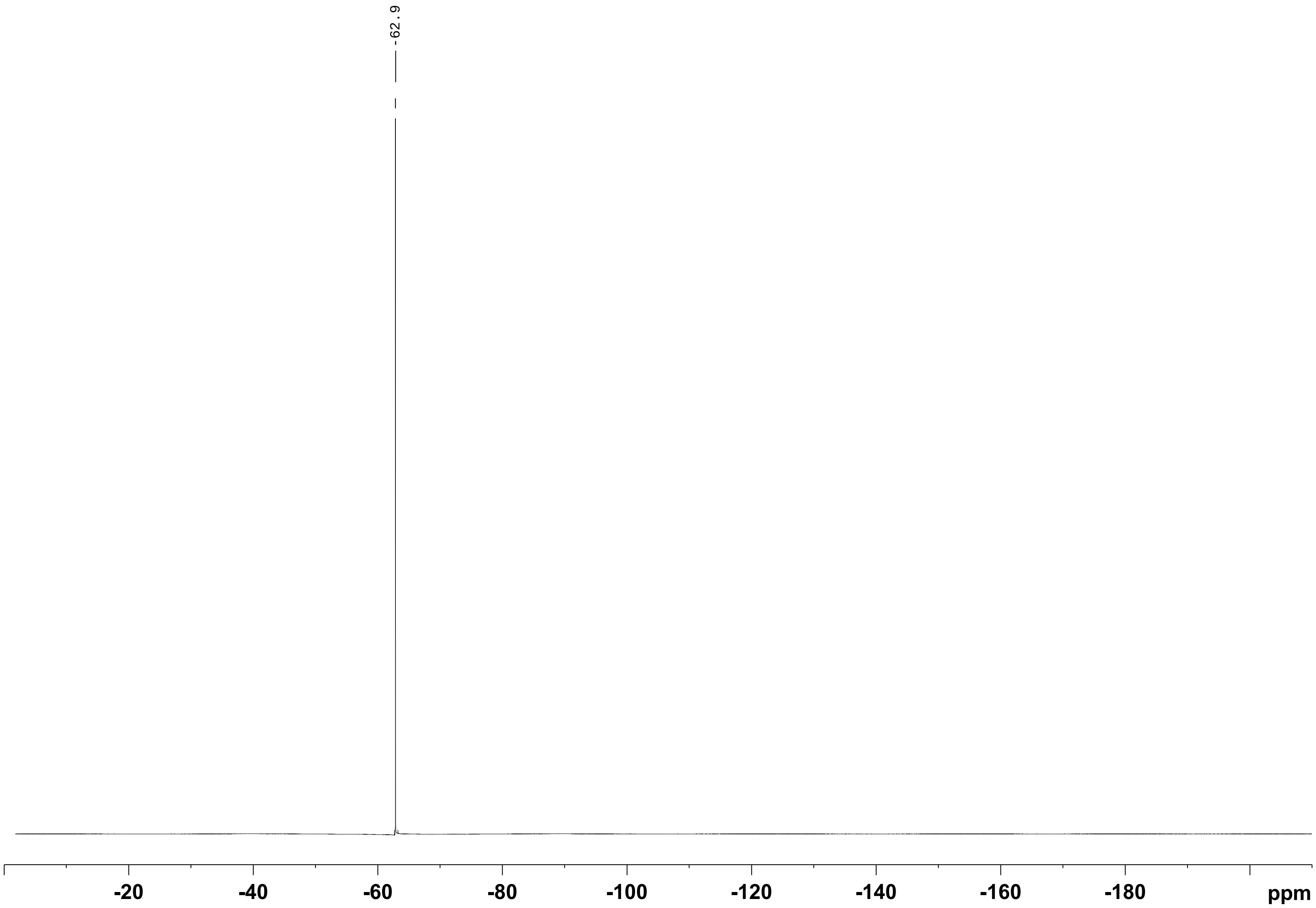

$^{31}P\{^{1}H\}$ NMR (203 MHz, CD_2Cl_2, 300 K): # = $[Et_3POSiEtMe_2]^{+}[BAr^{F}{}_4]^{-}$

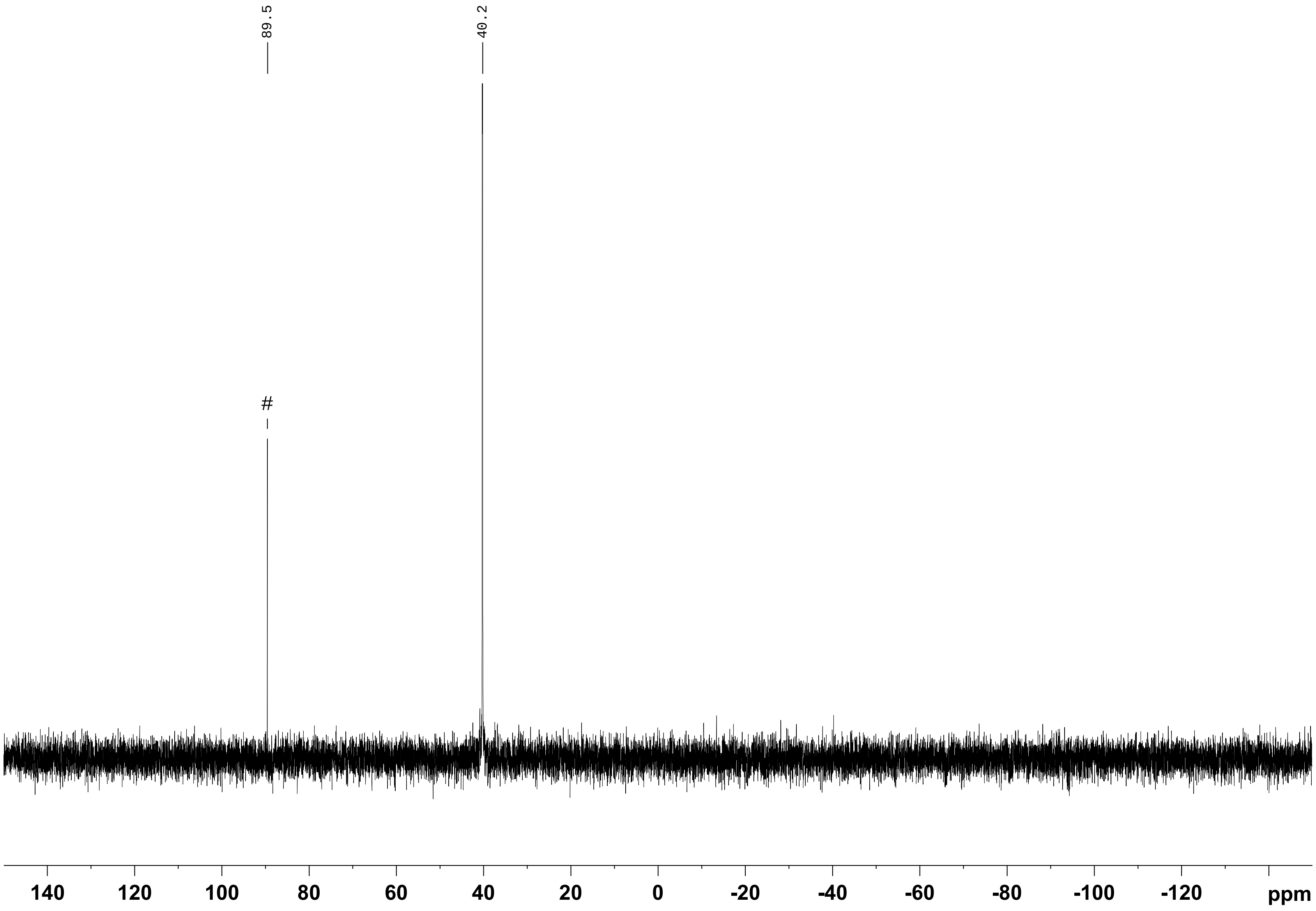

^{1}H,^{29}Si HMQC NMR (500/99 MHz, CD_2Cl_2, 300 K, optimized for *J* = 8 Hz): * = $EtMe_2SiH$, # = $[Et_3POSiEtMe_2]^+[BAr^F{}_4]^-$

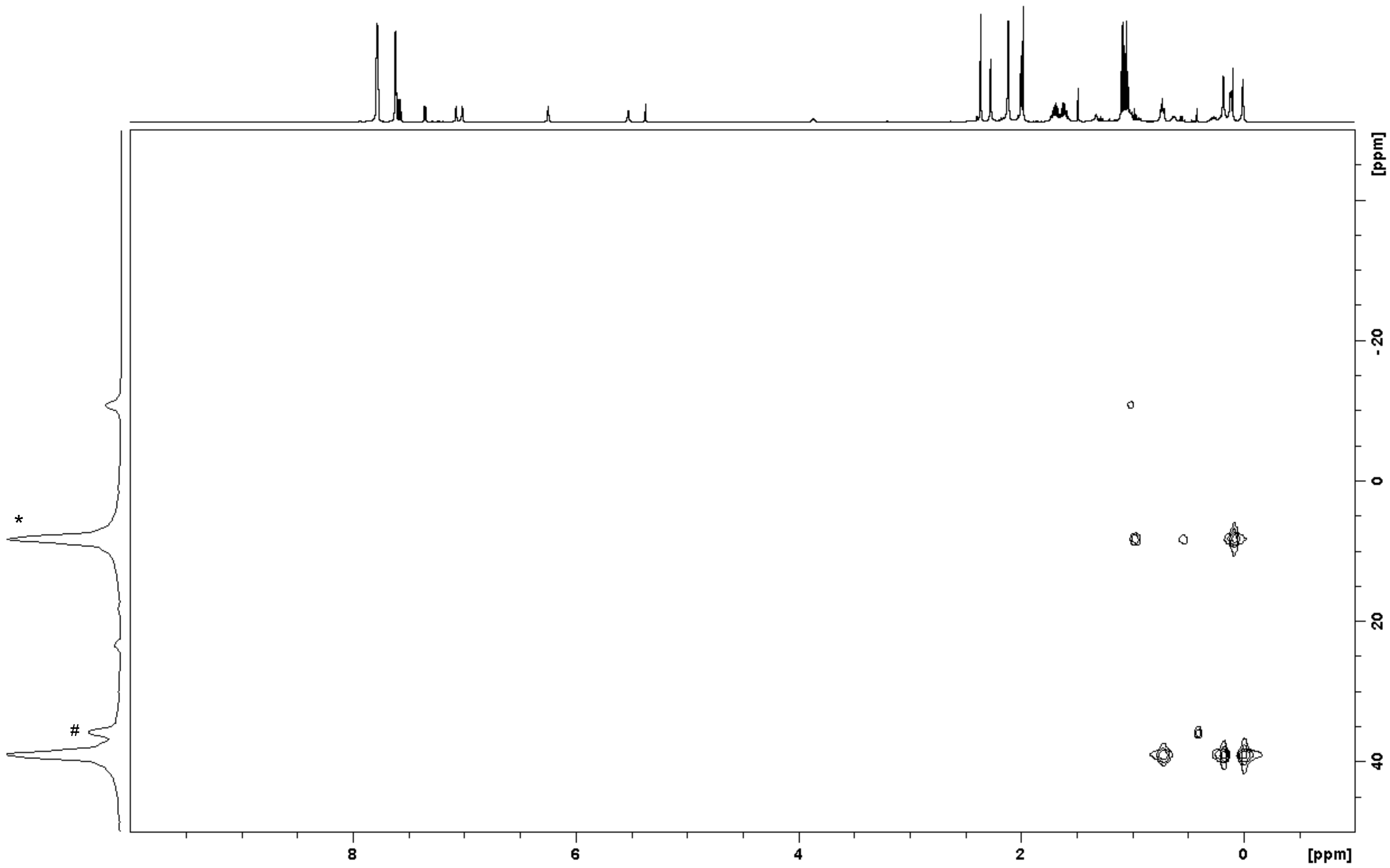

$^{1}H,^{31}P$ HMQC NMR (500/203 MHz, CD_2Cl_2, 300 K, optimized for J = 7 Hz): # = $[Et_3POSiEtMe_2]^{+}[BAr^{F}{}_{4}]^{-}$

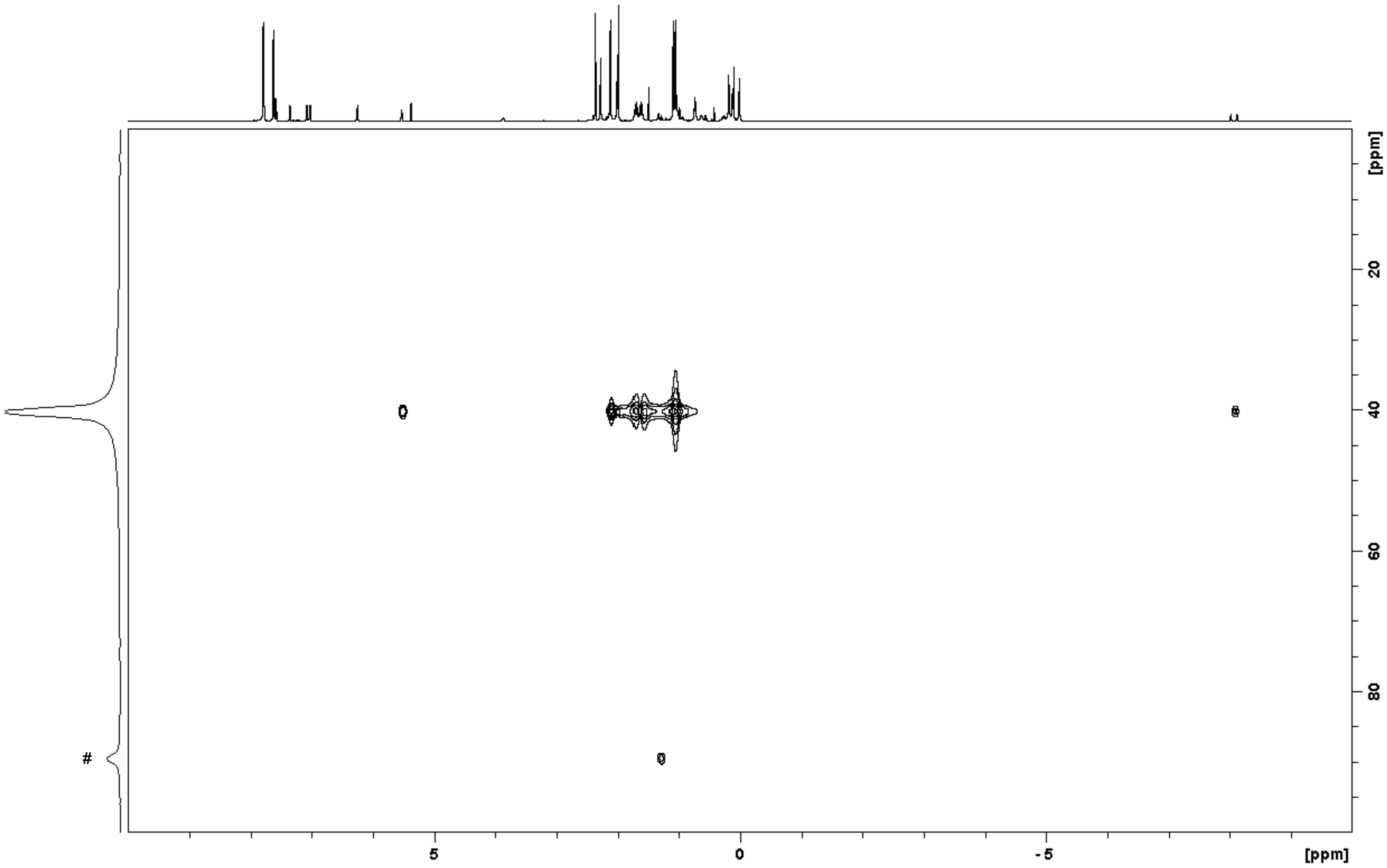

[{(*p*-$FC_6H_4)_3P$}Ru(SDmp)·$MePh_2SiH$i]$^+$[BAr^F_4]$^-$ (**3ba**)

^{1}H NMR (500 MHz, CD_2Cl_2, 300 K): * = $MePh_2SiH$

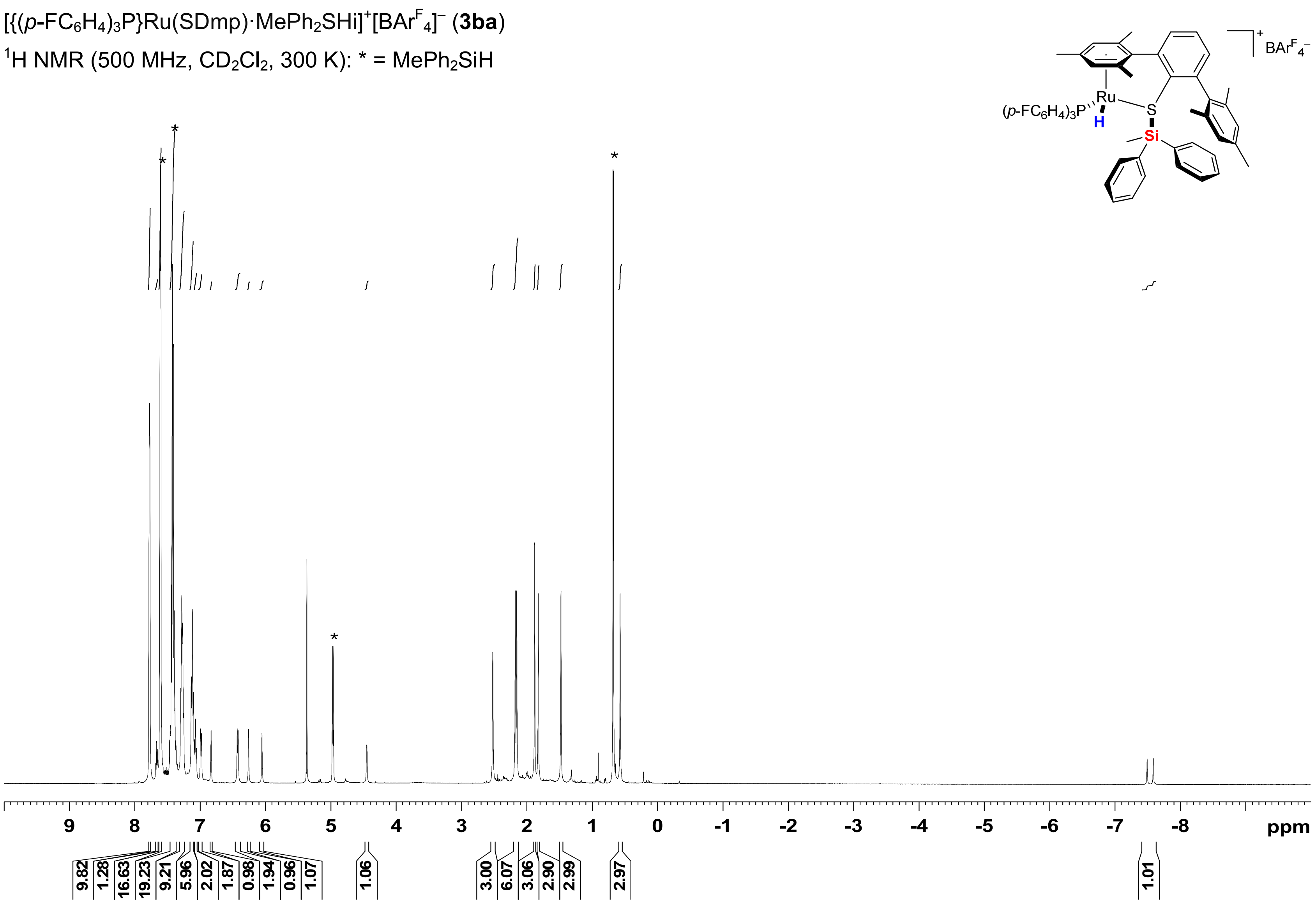

Solvent comparison

^{1}H NMR (500 MHz, 300 K):

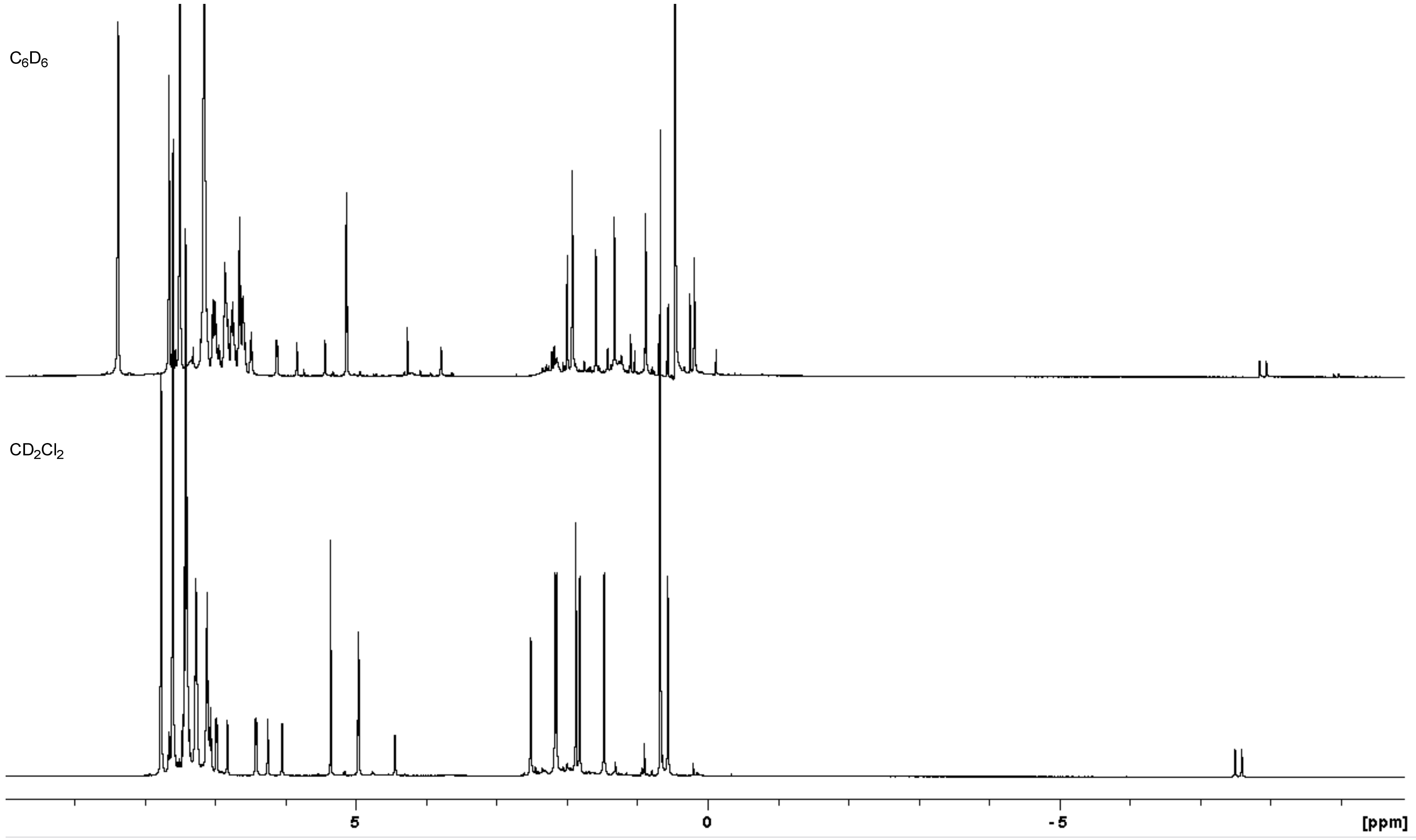

^{11}B NMR (161 MHz, CD_2Cl_2, 300 K):

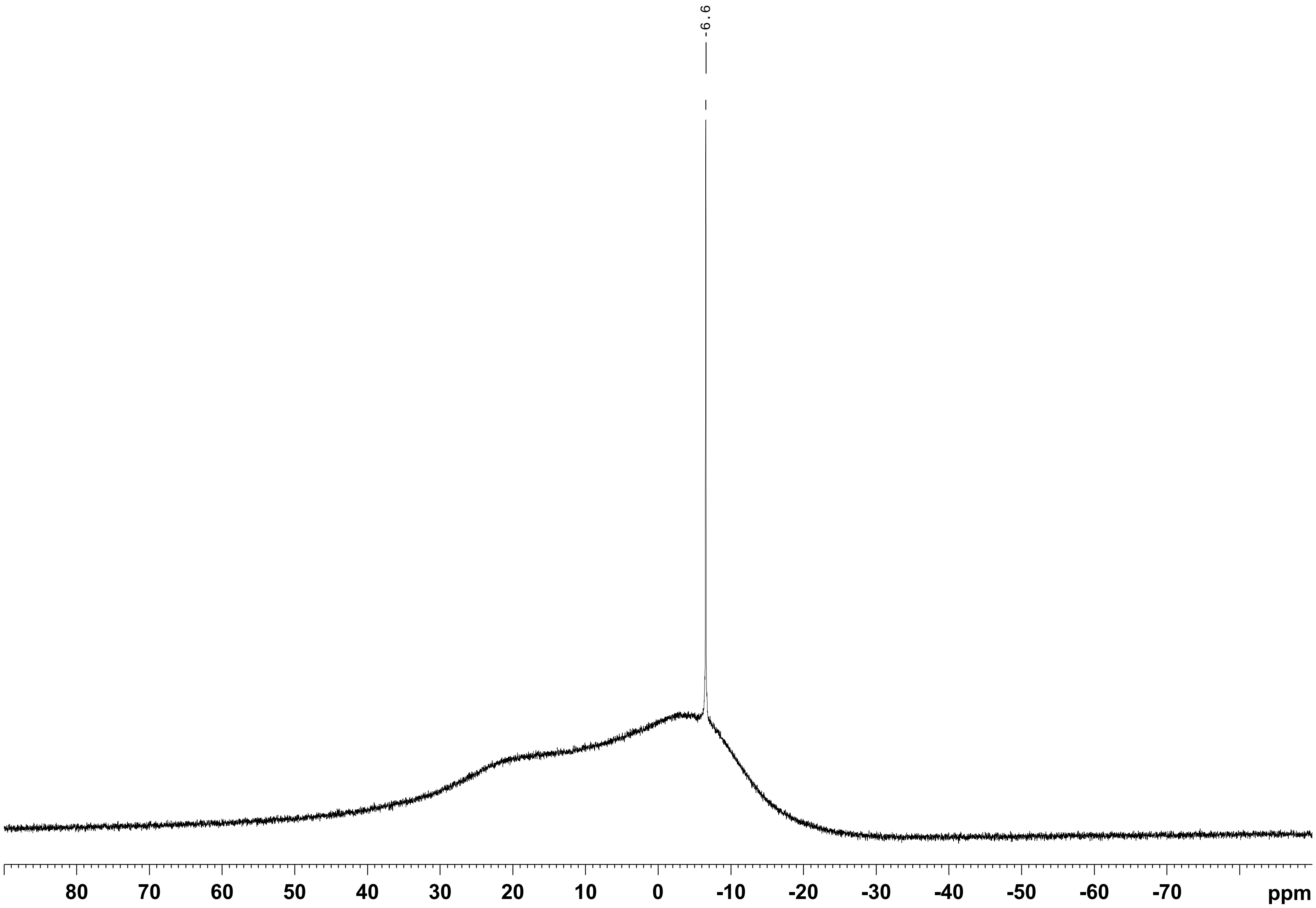

$^{19}F\{^{1}H\}$ NMR (471 MHz, CD_2Cl_2, 300 K):

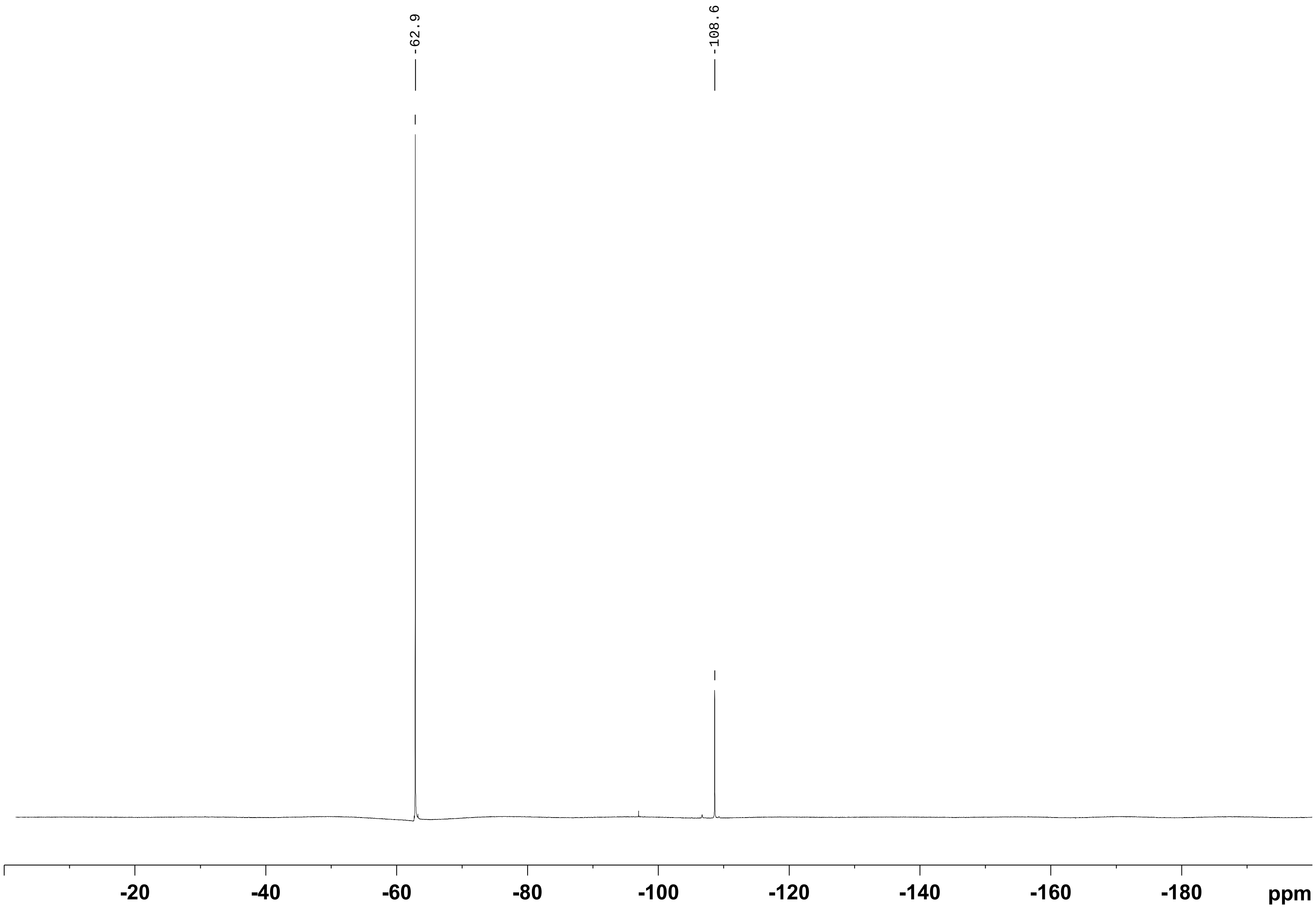

Solvent comparison

$^{19}F\{^{1}H\}$ NMR (471 MHz, 300 K):

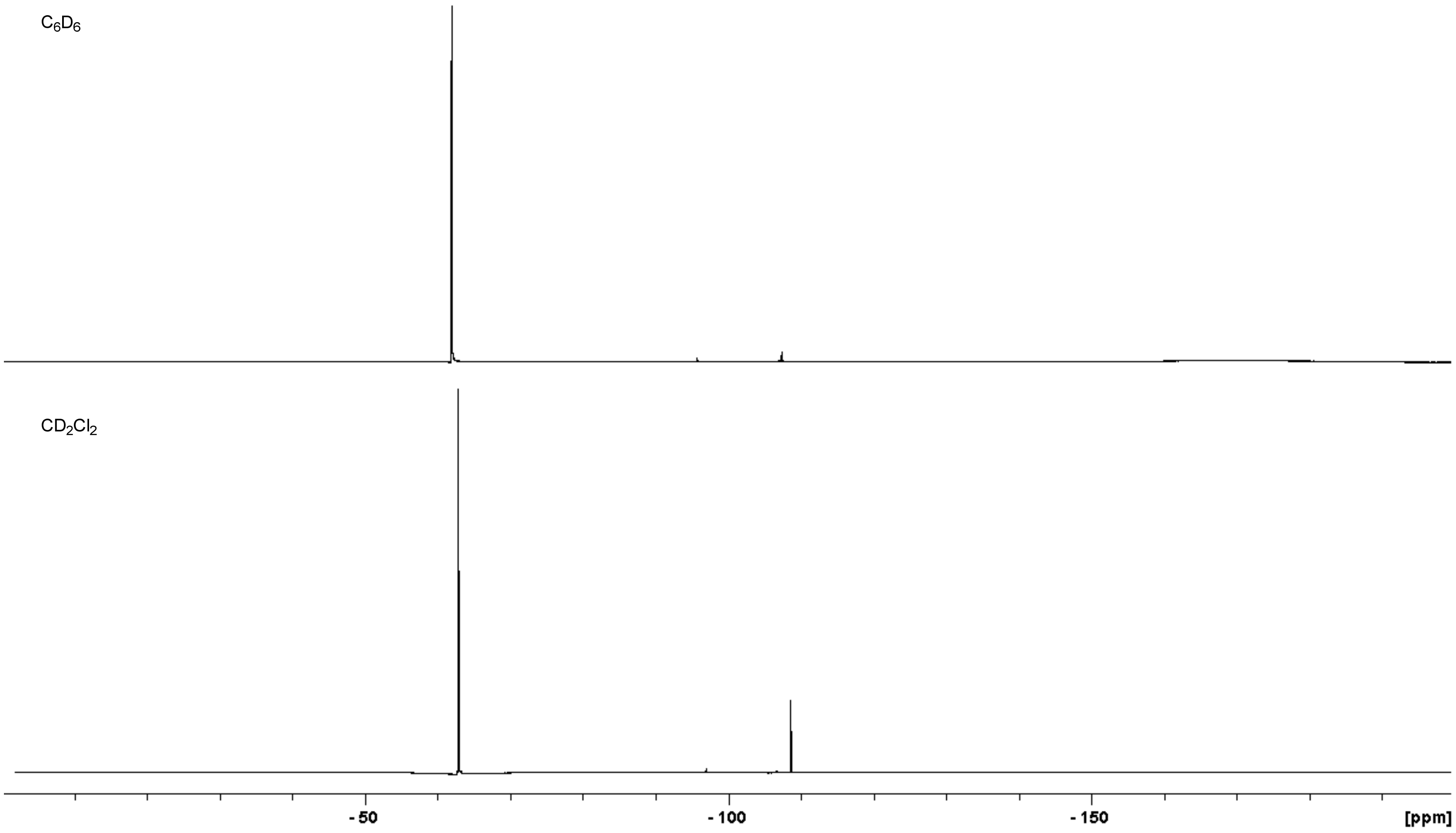

$^{31}P\{^{1}H\}$ NMR (203 MHz, CD_2Cl_2, 300 K): # = $[(p\text{-}FC_6H_4)_3POSiMePh_2]^+[BAr^F{}_4]^-$

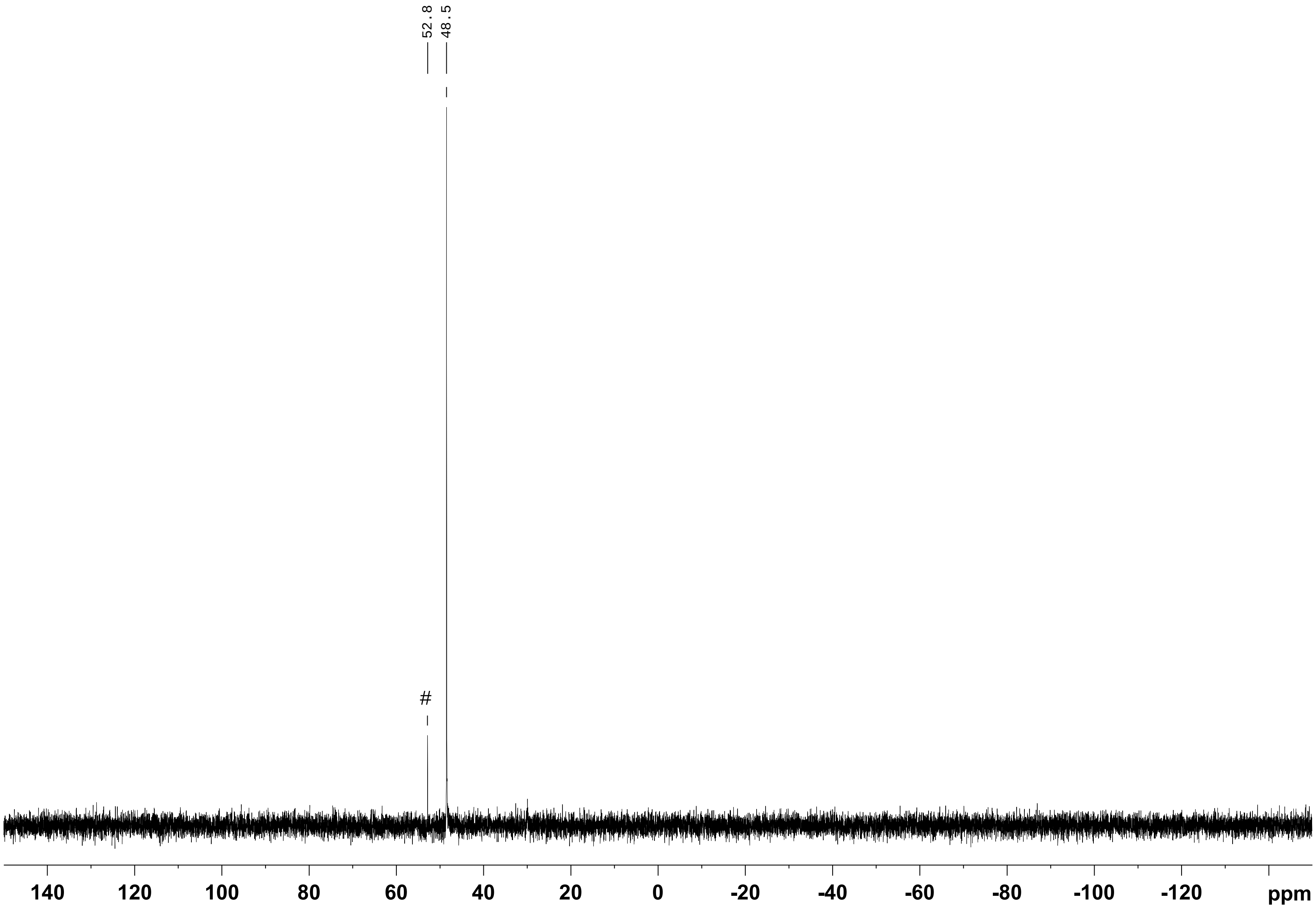

Solvent Comparison

$^{31}P\{^{1}H\}$ NMR (203 MHz, 300 K):

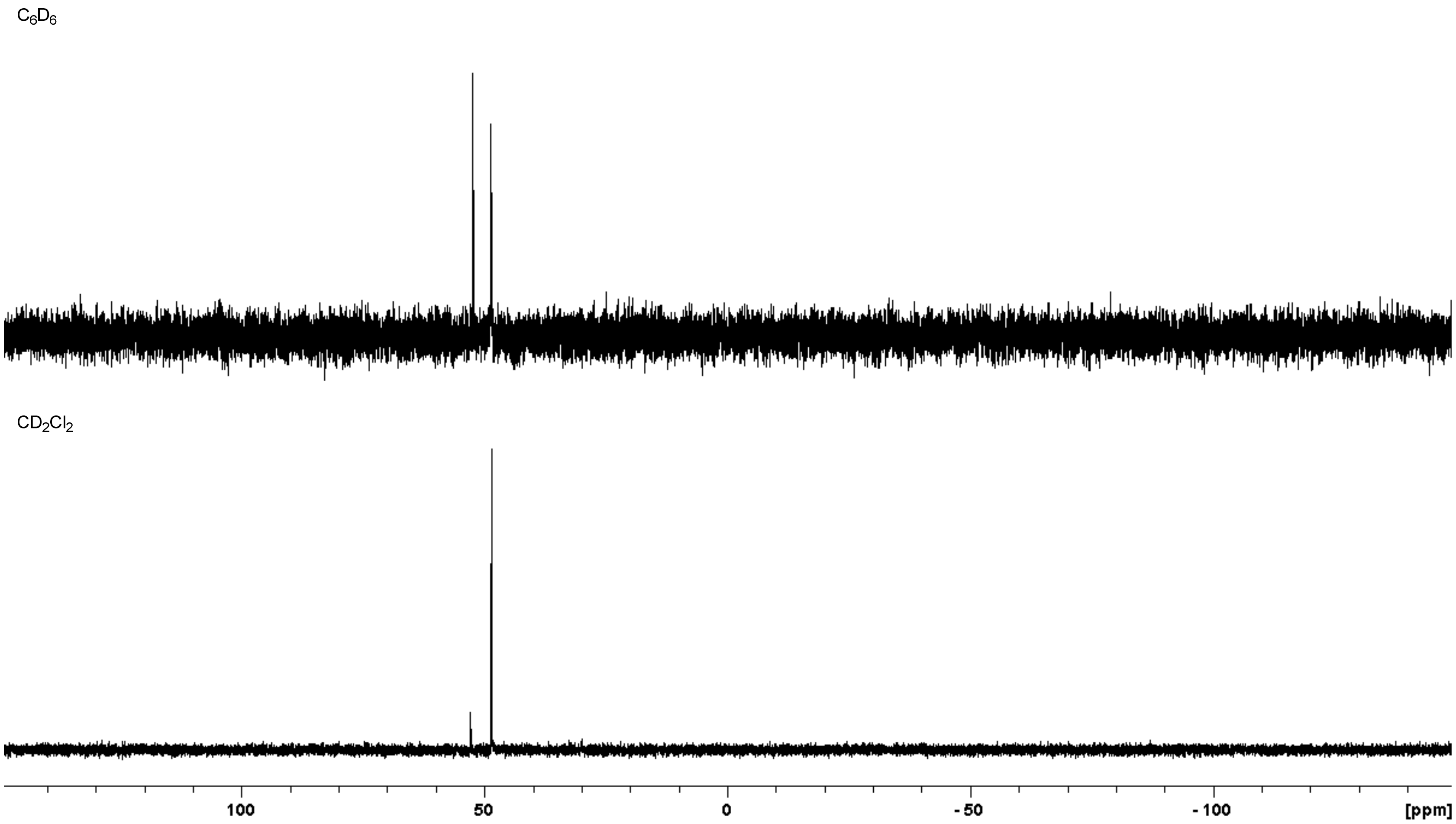

$^{1}H,^{1}H$ EXSY NMR (500/500 MHz, CD_2Cl_2, 300 K, T_m = 200 ms):

blue = normal phase, black = negative phase

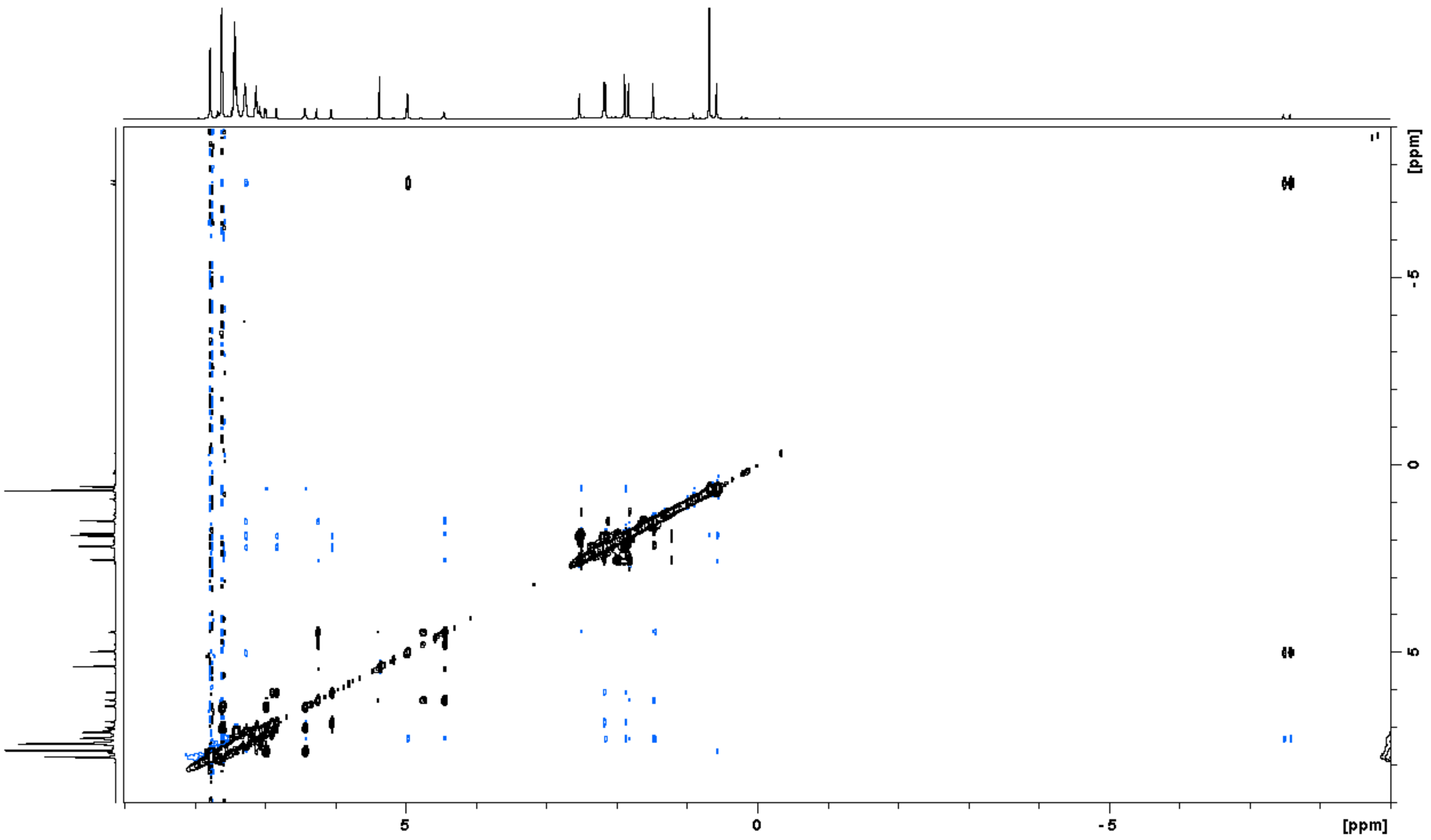

$^{1}H,^{13}C$ HSQC NMR (500/126 MHz, CD_2Cl_2, 300 K):

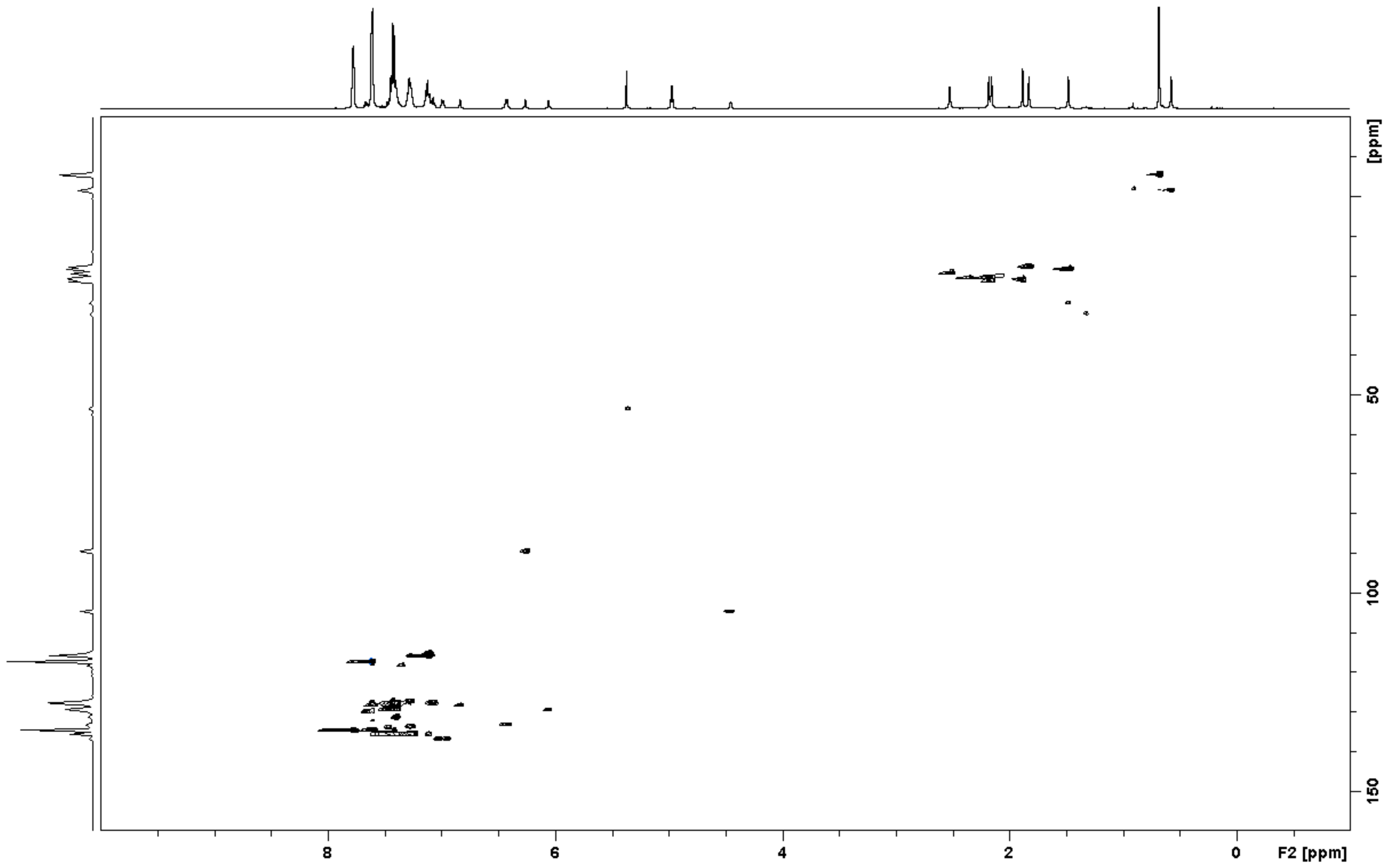

$^{1}H,^{13}C$ HMBC NMR (500/126 MHz, CD_2Cl_2, 300 K):

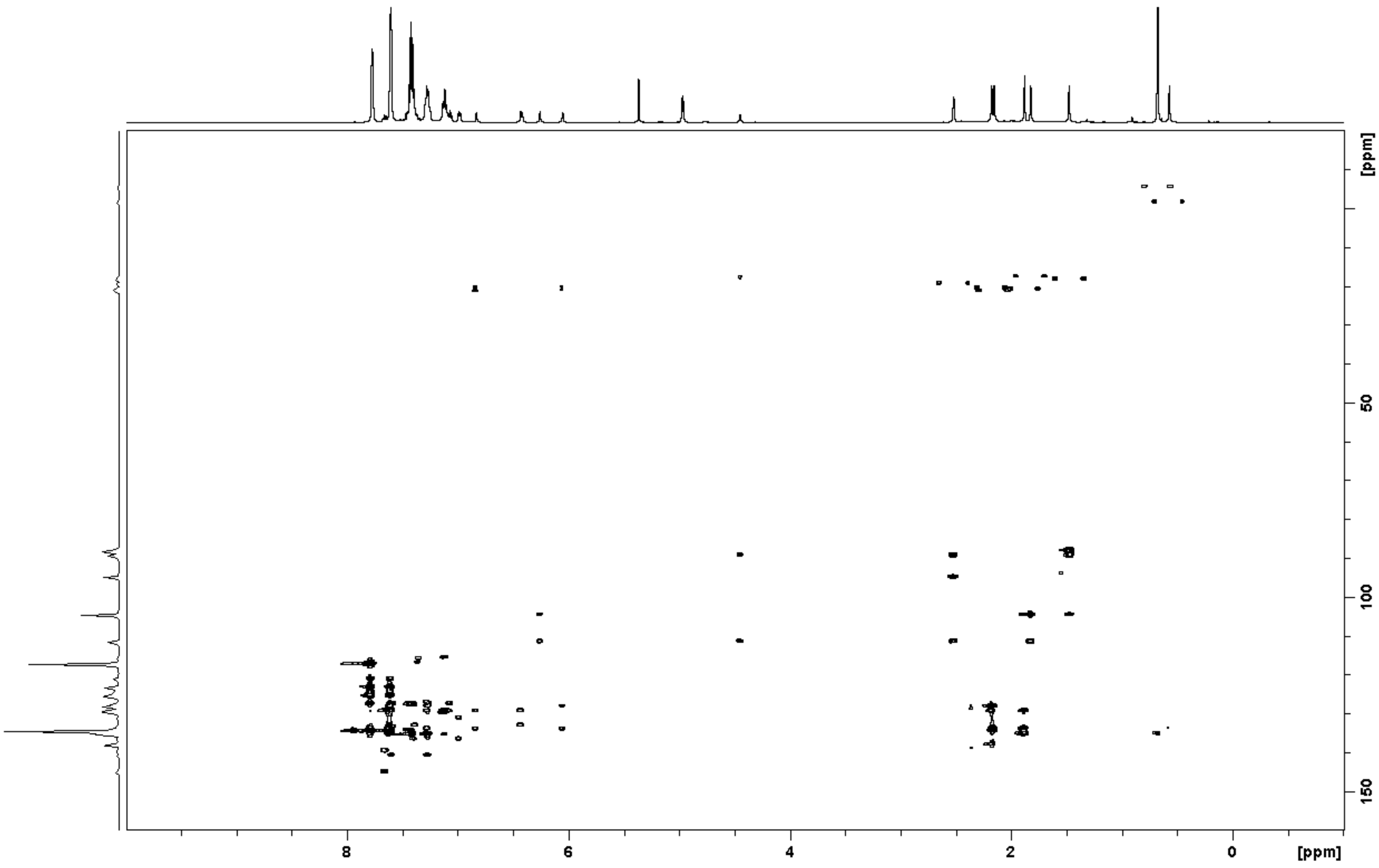

$^{1}H,^{19}F$ HMQC NMR (500/471 MHz, CD_2Cl_2, 300 K, optimized for J = 30 Hz):

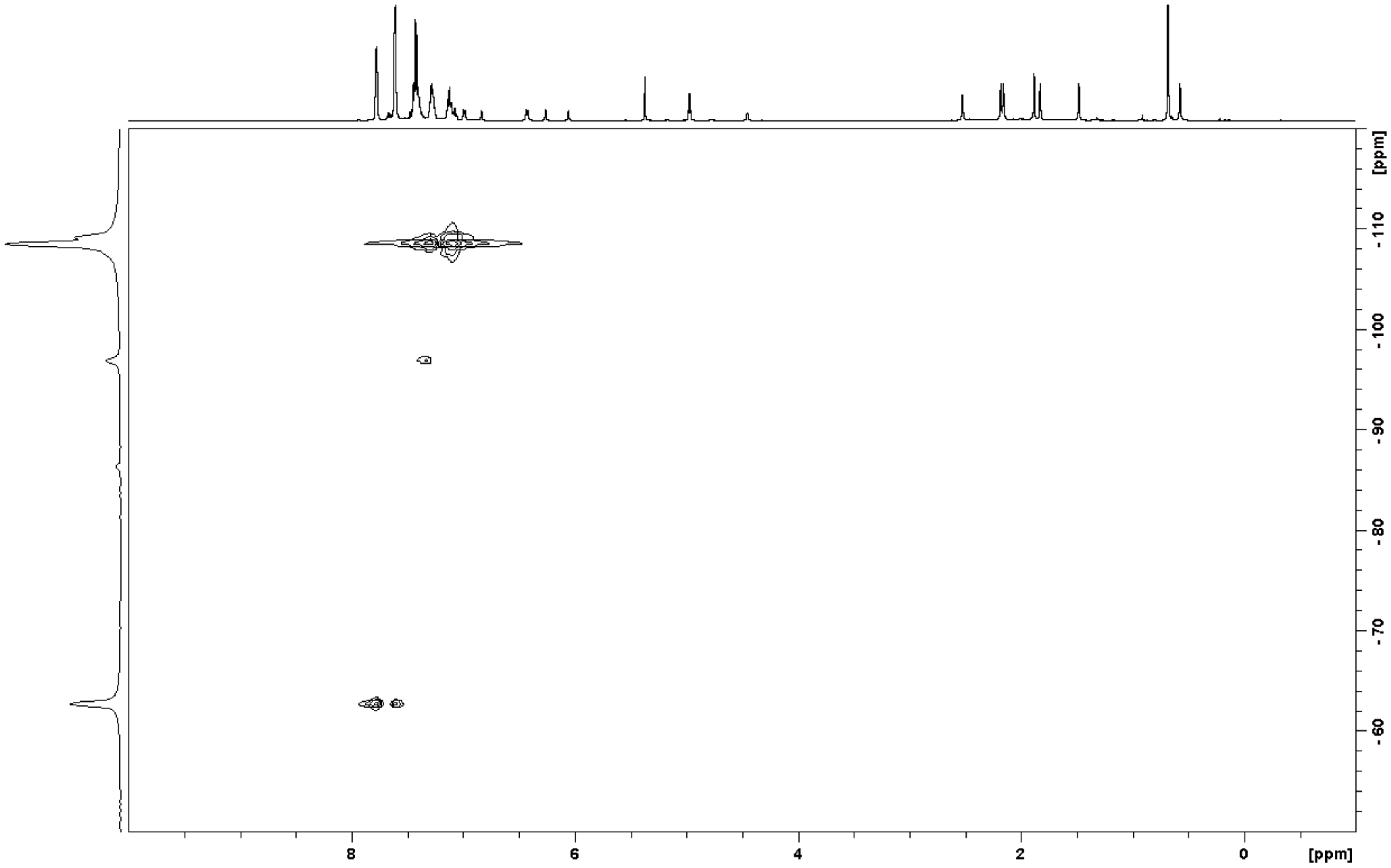

$^{1}H,^{29}Si$ HMQC NMR (500/99 MHz, CD_2Cl_2, 300 K, optimized for *J* = 8 Hz): * = $MePh_2SiH$, # = $[(p\text{-}FC_6H_4)_3POSiMePh_2]^+[BAr^F{}_4]^-$

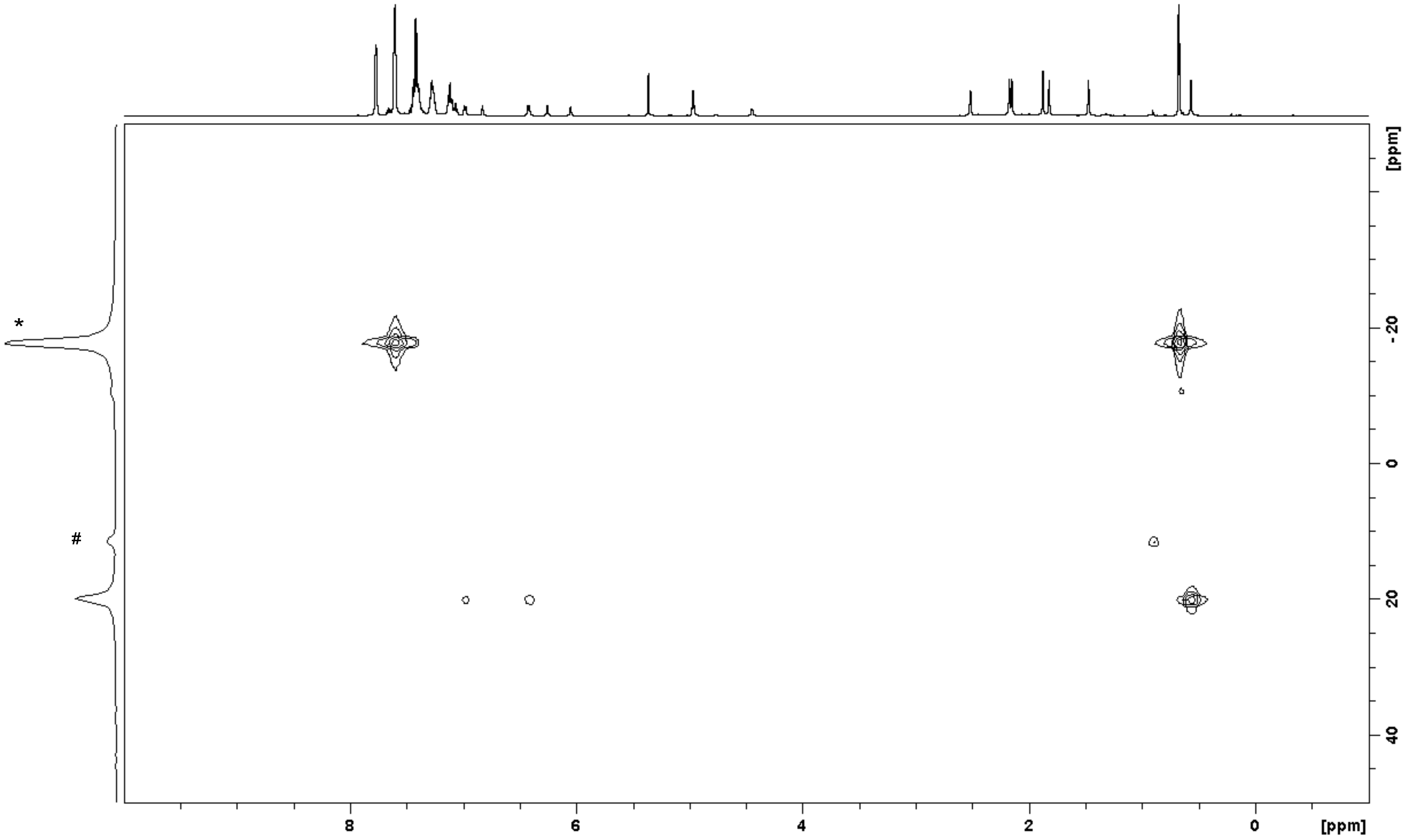

$^{1}H,^{31}P$ HMQC NMR (500/203 MHz, CD_2Cl_2, 300 K, optimized for J = 7 Hz): # = $[(p\text{-}FC_6H_4)_3POSiMePh_2]^+[BAr^F{}_4]^-$

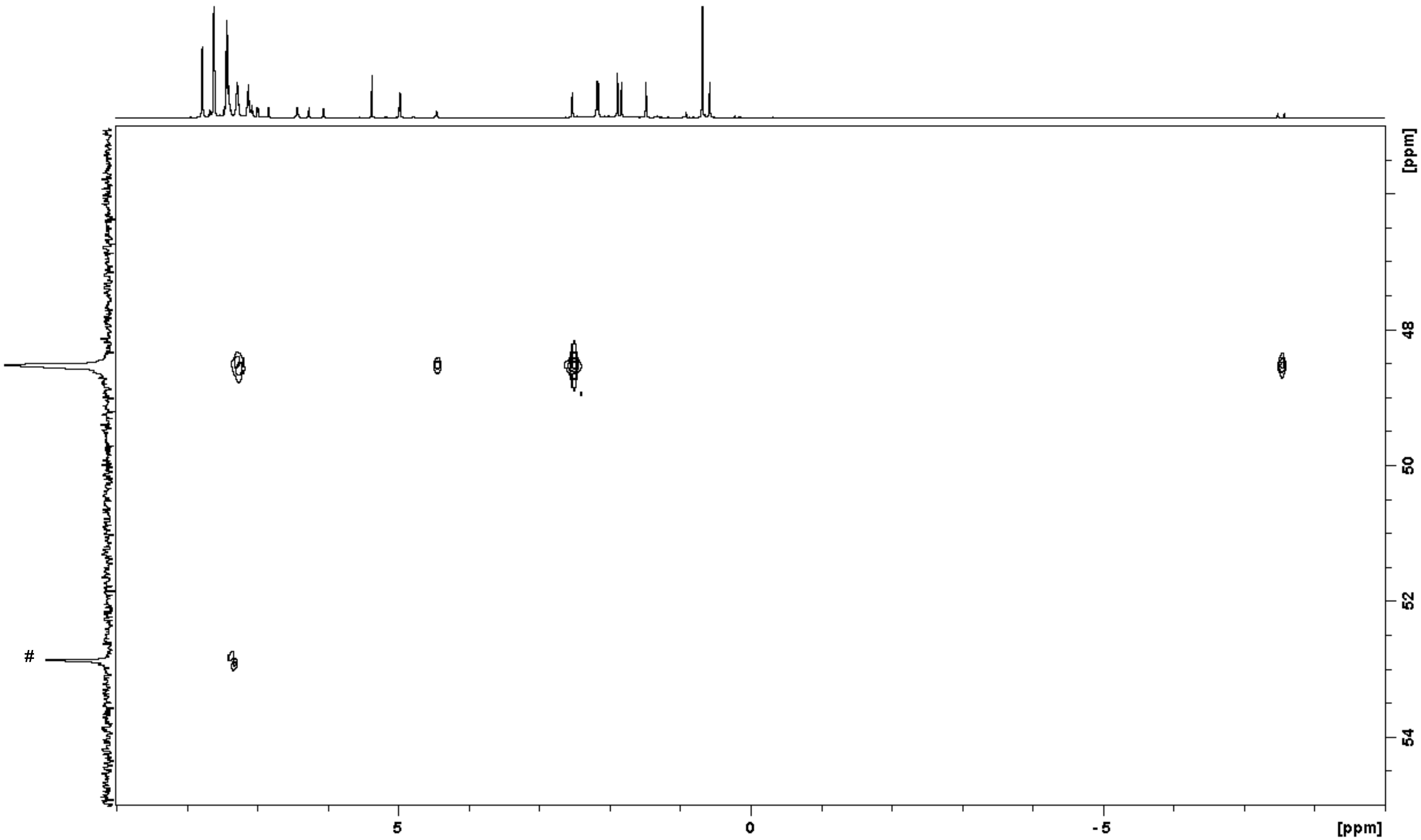

^{1}H DOSY NMR (500 MHz, CD_2Cl_2, 300 K):

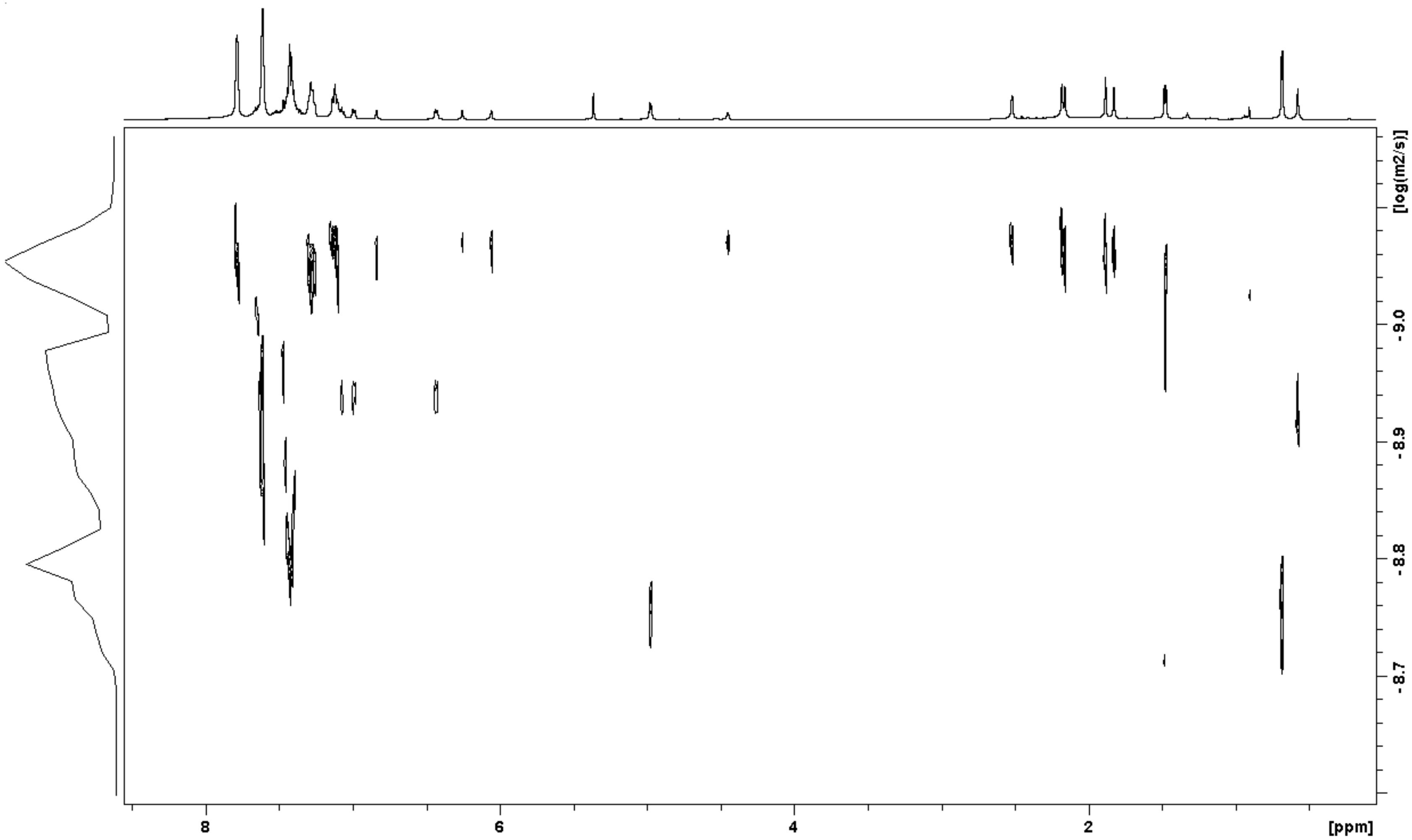

[{(*p*-$FC_6H_4)_3P$}Ru(SDmp)·$Me_2PhSiH]^+[BAr^F_4]^-$ (**3bb**)

^1H NMR (500 MHz, CD_2Cl_2, 300 K): * = Me_2PhSiH

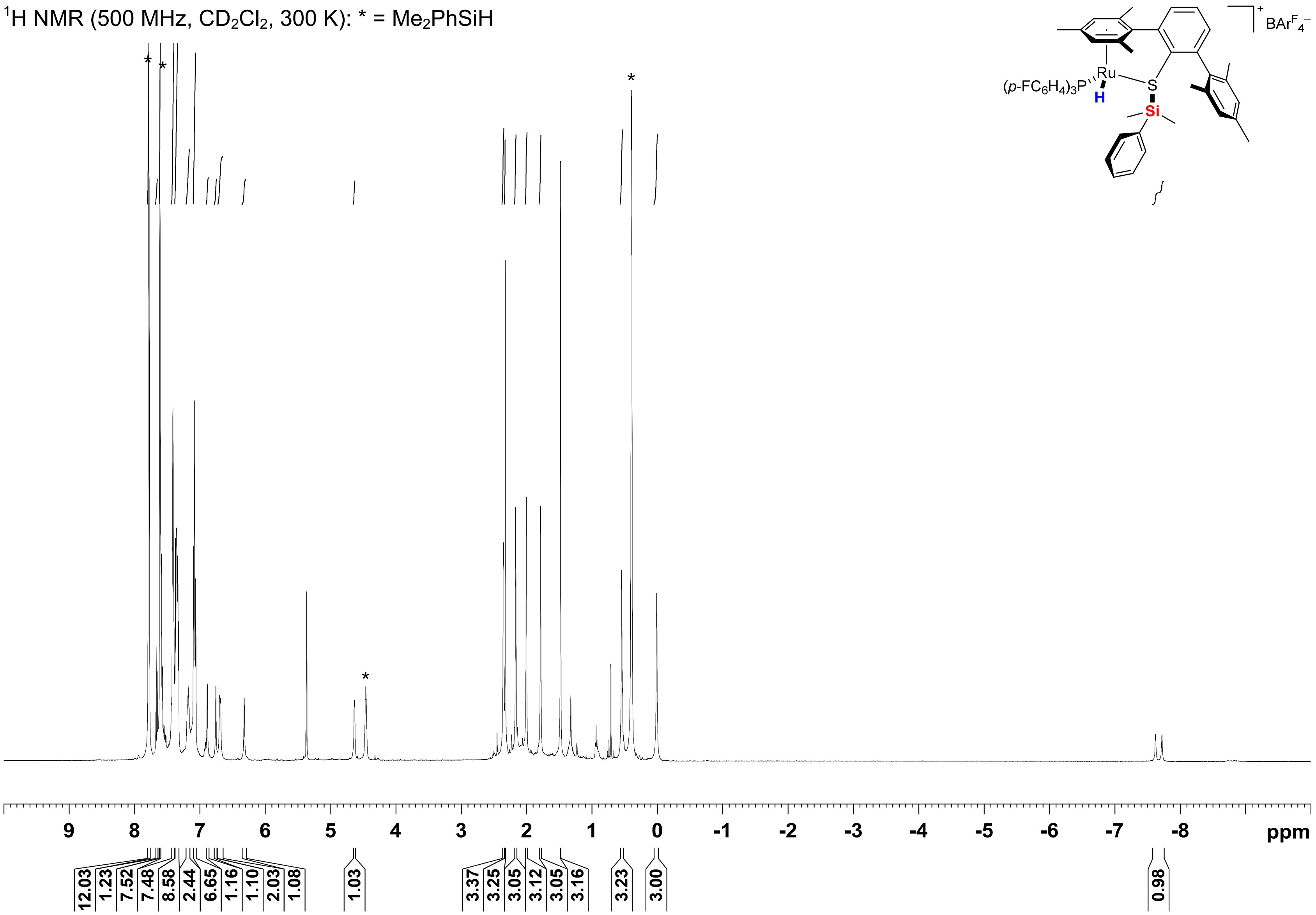

^{11}B NMR (161 MHz, CD_2Cl_2, 300 K):

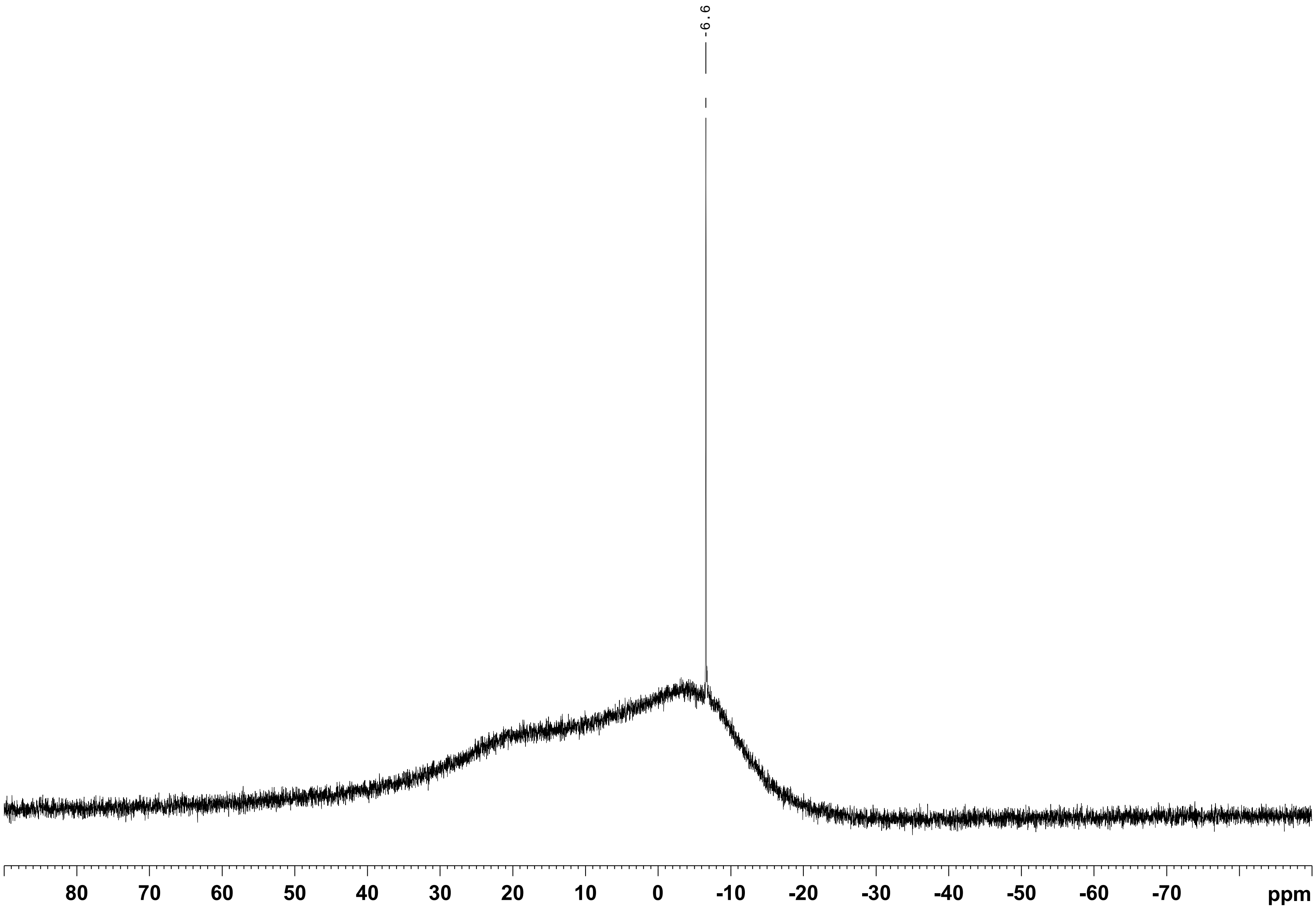

$^{19}F\{^1H\}$ NMR (471 MHz, CD_2Cl_2, 300 K):

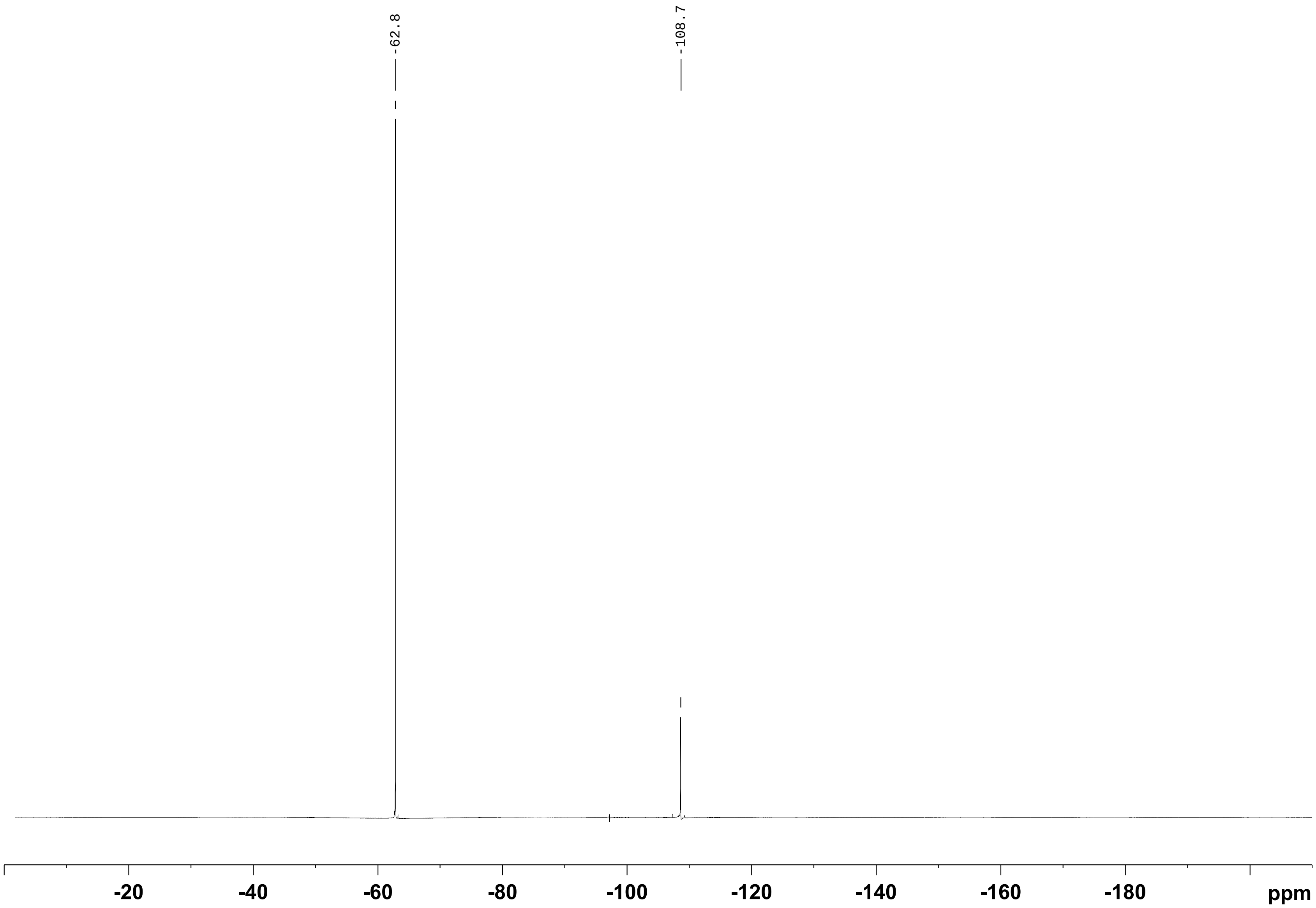

$^{31}P\{^{1}H\}$ NMR (203 MHz, CD_2Cl_2, 300 K): # = $[(p\text{-}FC_6H_4)_3POSiMe_2Ph]^+[BAr^F{}_4]^-$

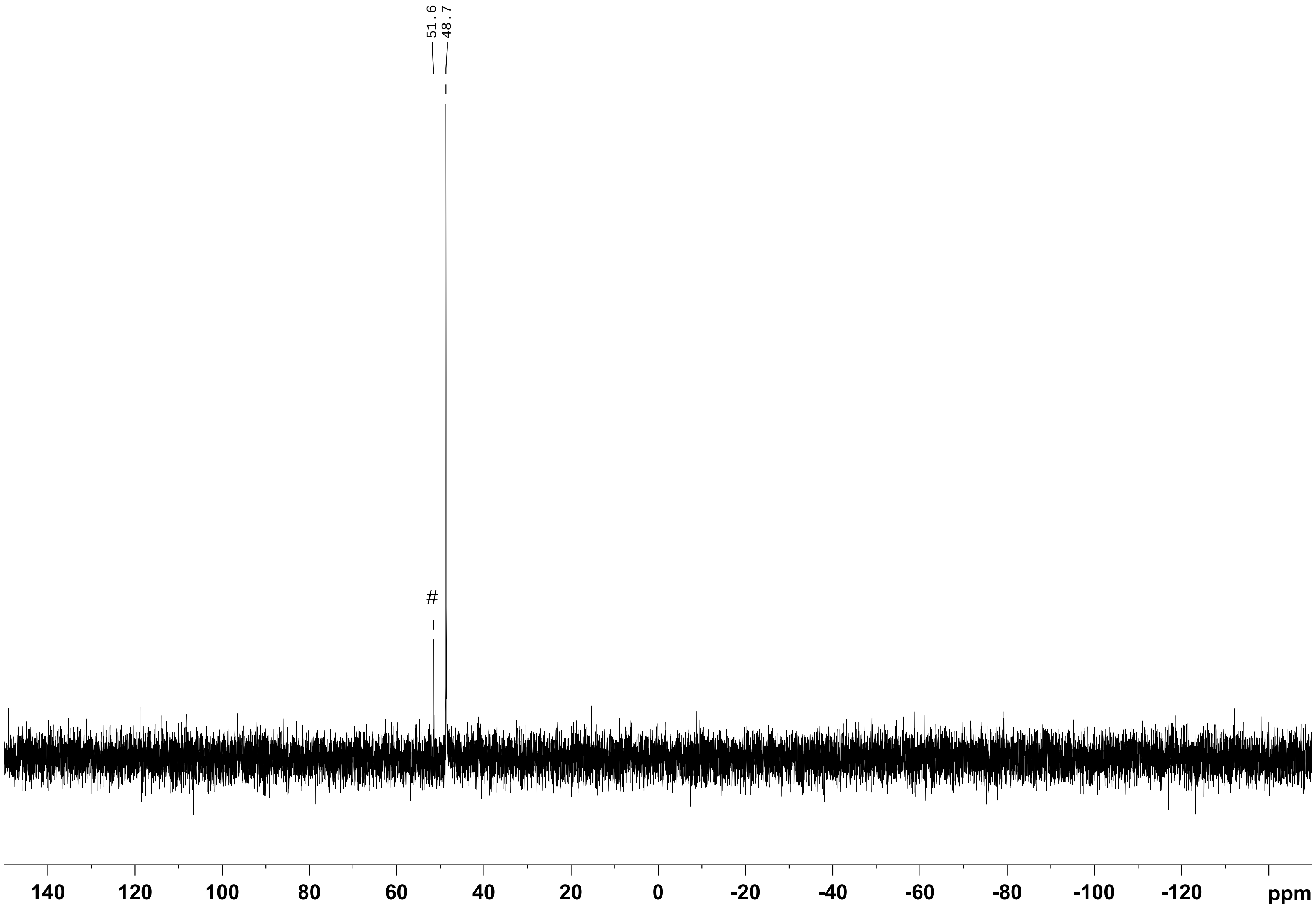

$^{1}H,^{29}Si$ HMQC NMR (500/99 MHz, CD_2Cl_2, 300 K, optimized for J = 8 Hz): * = Me_2PhSiH, # = $[(p\text{-}FC_6H_4)_3POSiMe_2Ph]^+[BAr^F{}_4]^-$

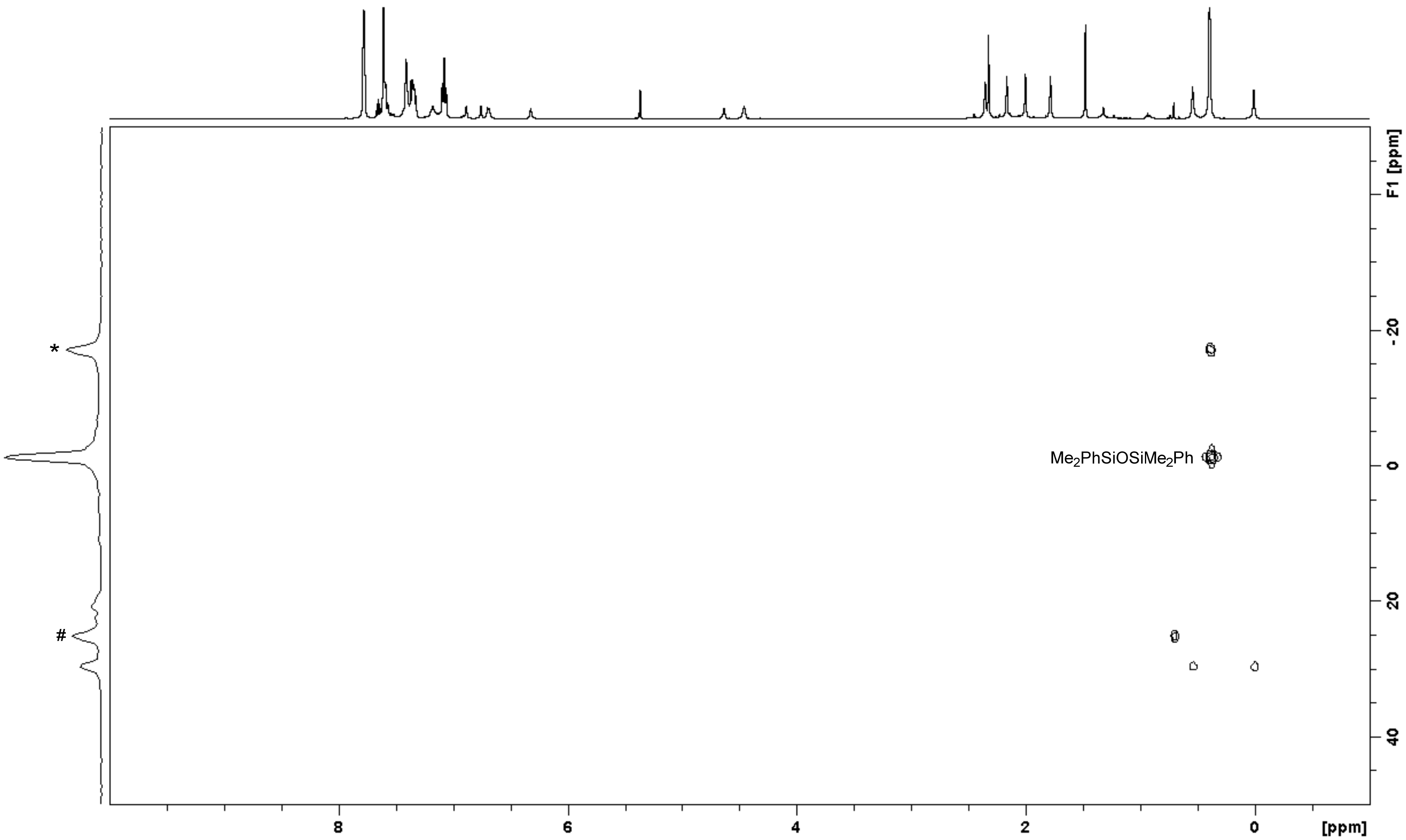

$^{1}H,^{31}P$ HMQC NMR (500/203 MHz, CD_2Cl_2, 300 K, optimized for J = 7 Hz): # = $[(p\text{-}FC_6H_4)_3POSiMe_2Ph]^+[BAr^F{}_4]^-$

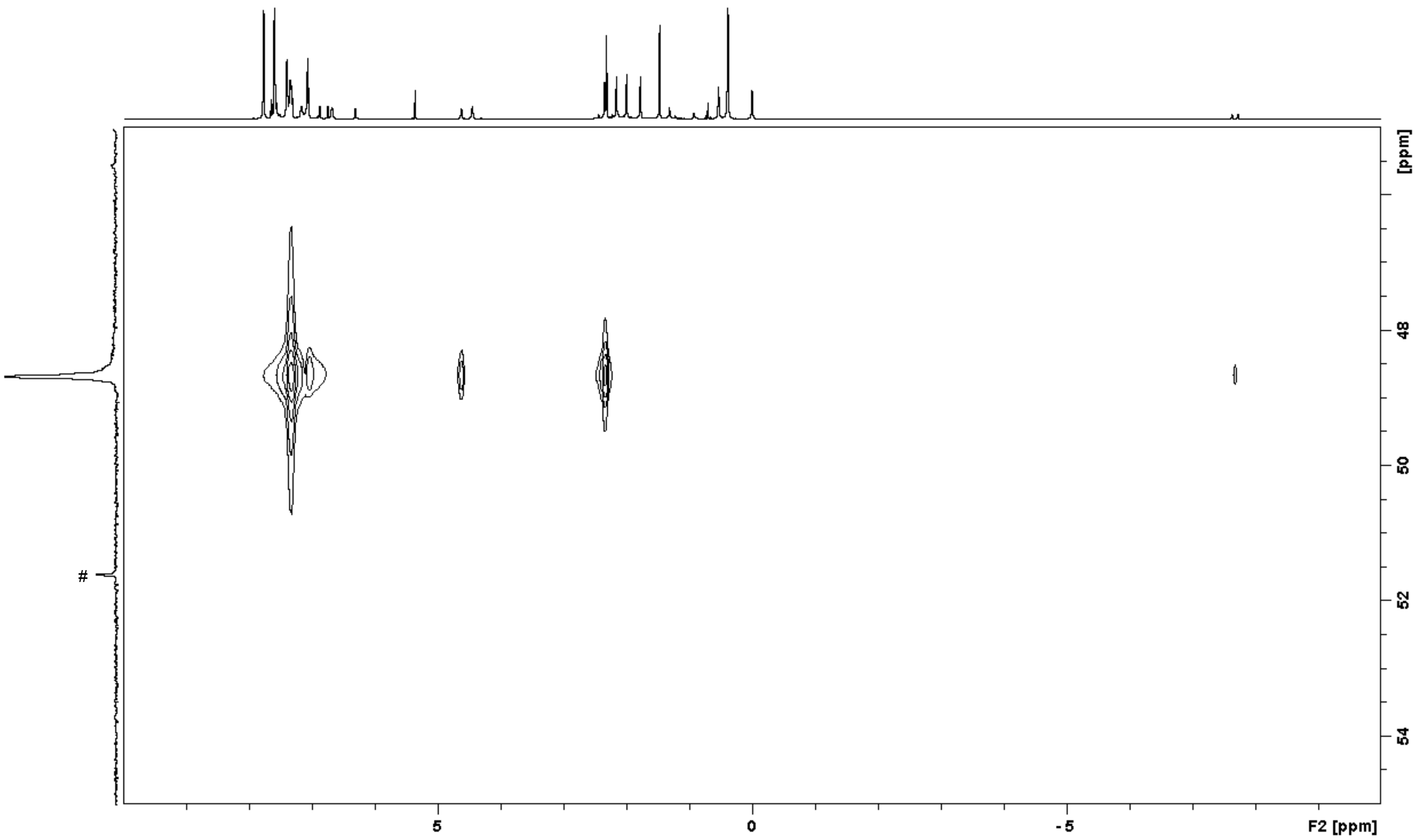

$[\{(p\text{-}FC_6H_4)_3P\}Ru(SDmp)\cdot Et_3SiH]^+[BAr^F_4]^-$ (**3bc**)

^1H NMR (500 MHz, CD_2Cl_2, 300 K): * = Et_3SiH

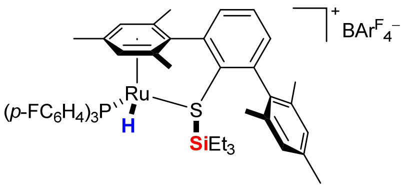

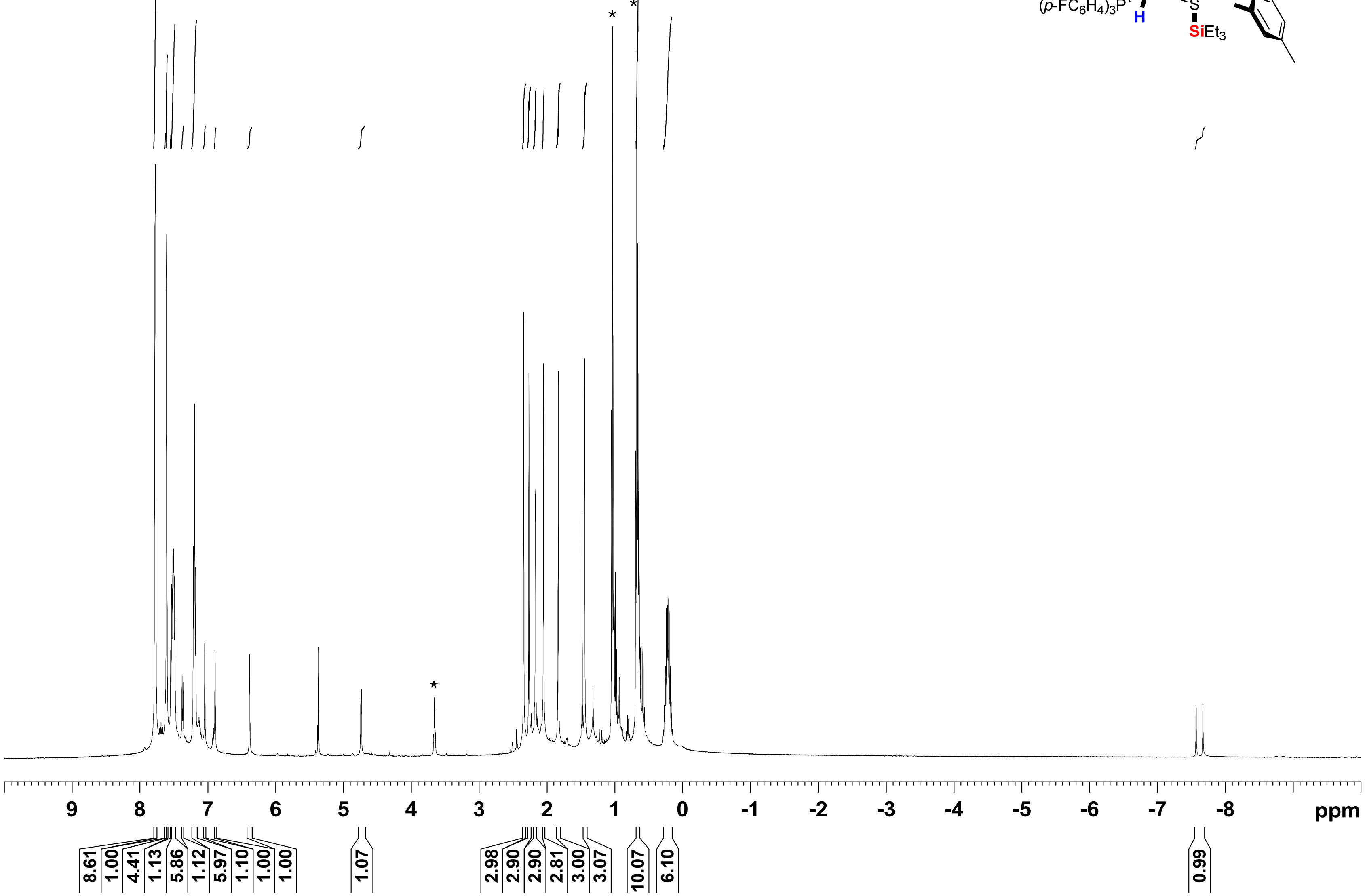

^{11}B NMR (161 MHz, CD_2Cl_2, 300 K):

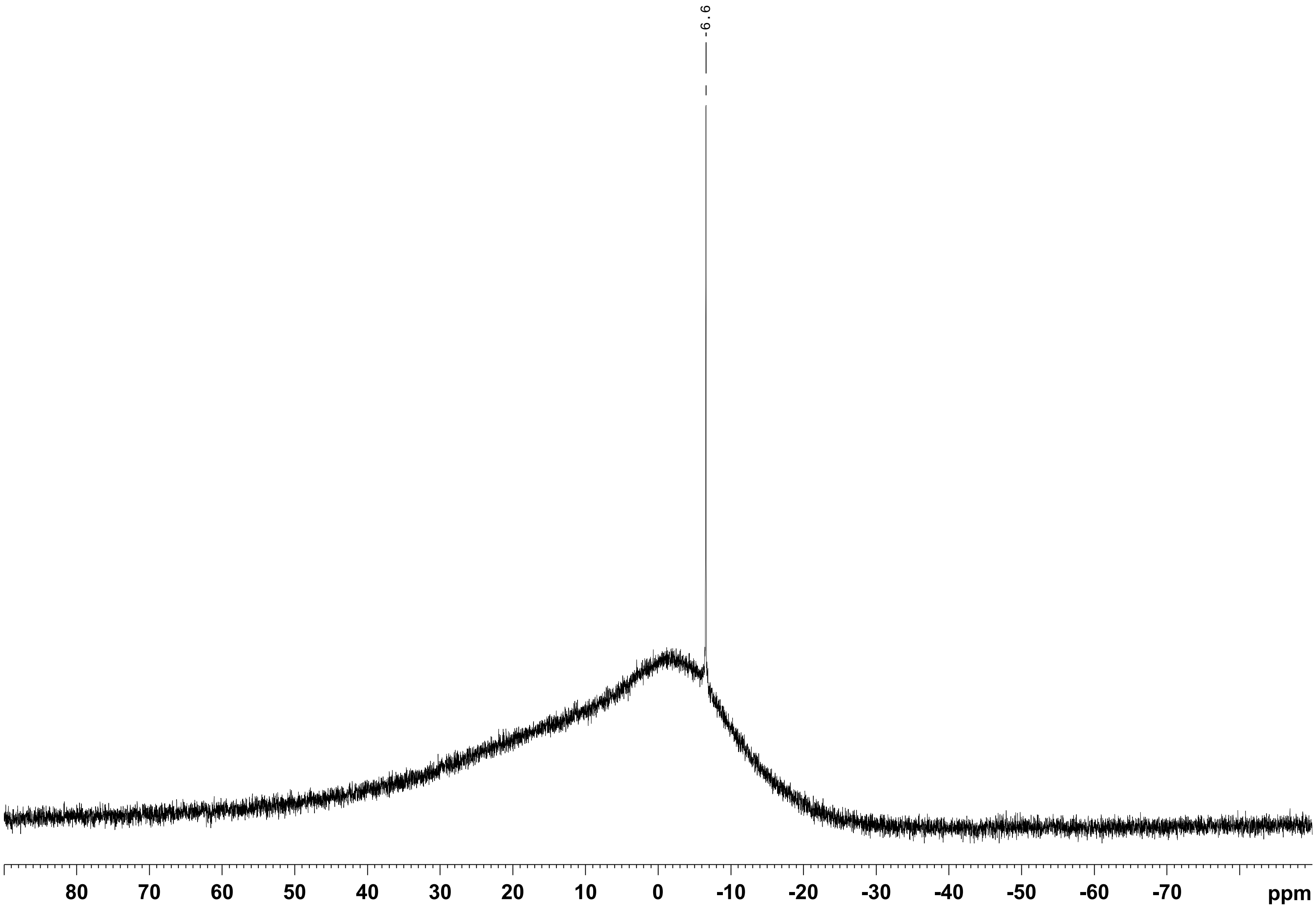

$^{19}F\{^{1}H\}$ NMR (471 MHz, CD_2Cl_2, 300 K):

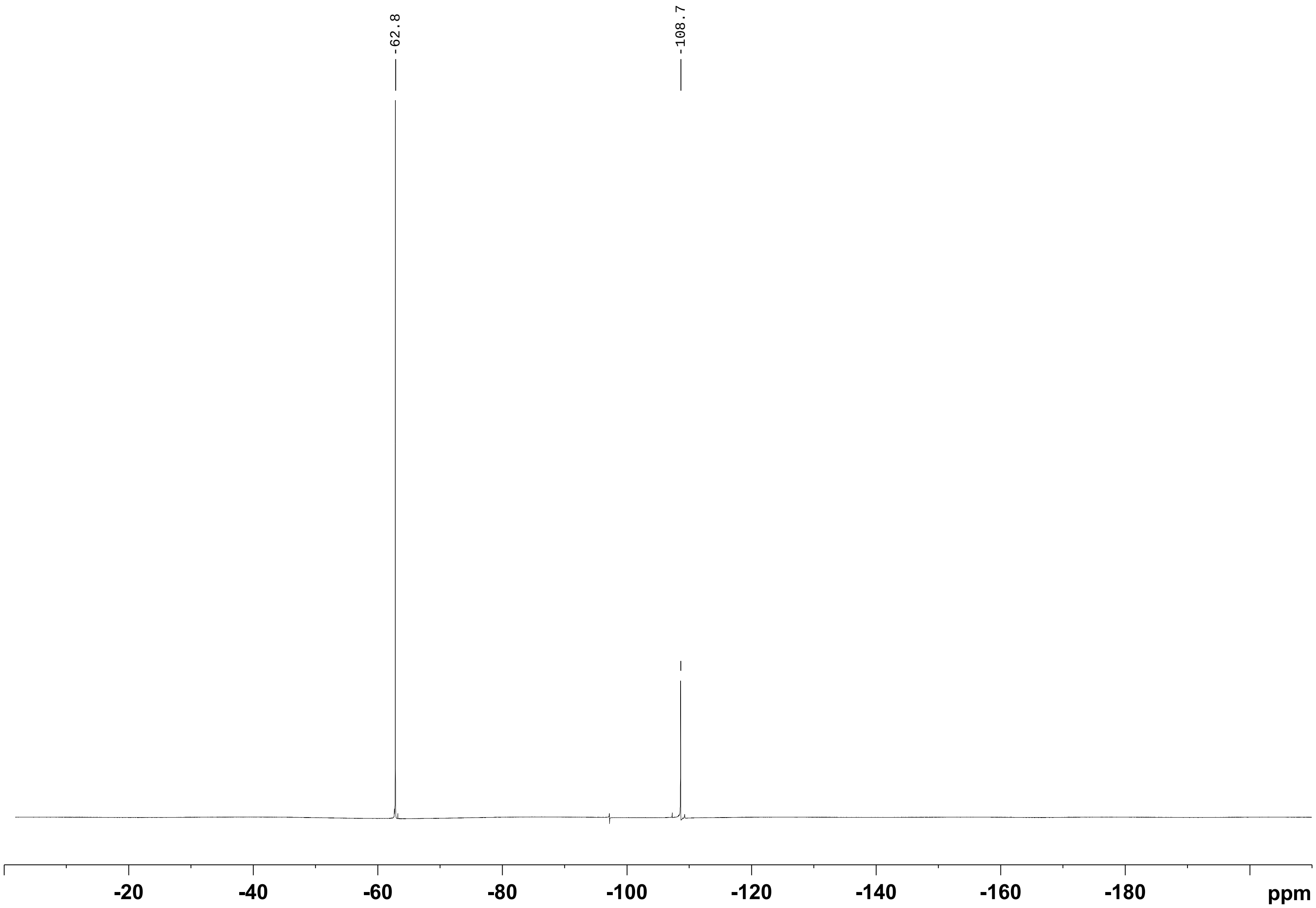

$^{31}P\{^{1}H\}$ NMR (203 MHz, CD_2Cl_2, 300 K): # = $[(p\text{-}FC_6H_4)_3POSiEt_3]^+[BAr^F{}_4]^-$

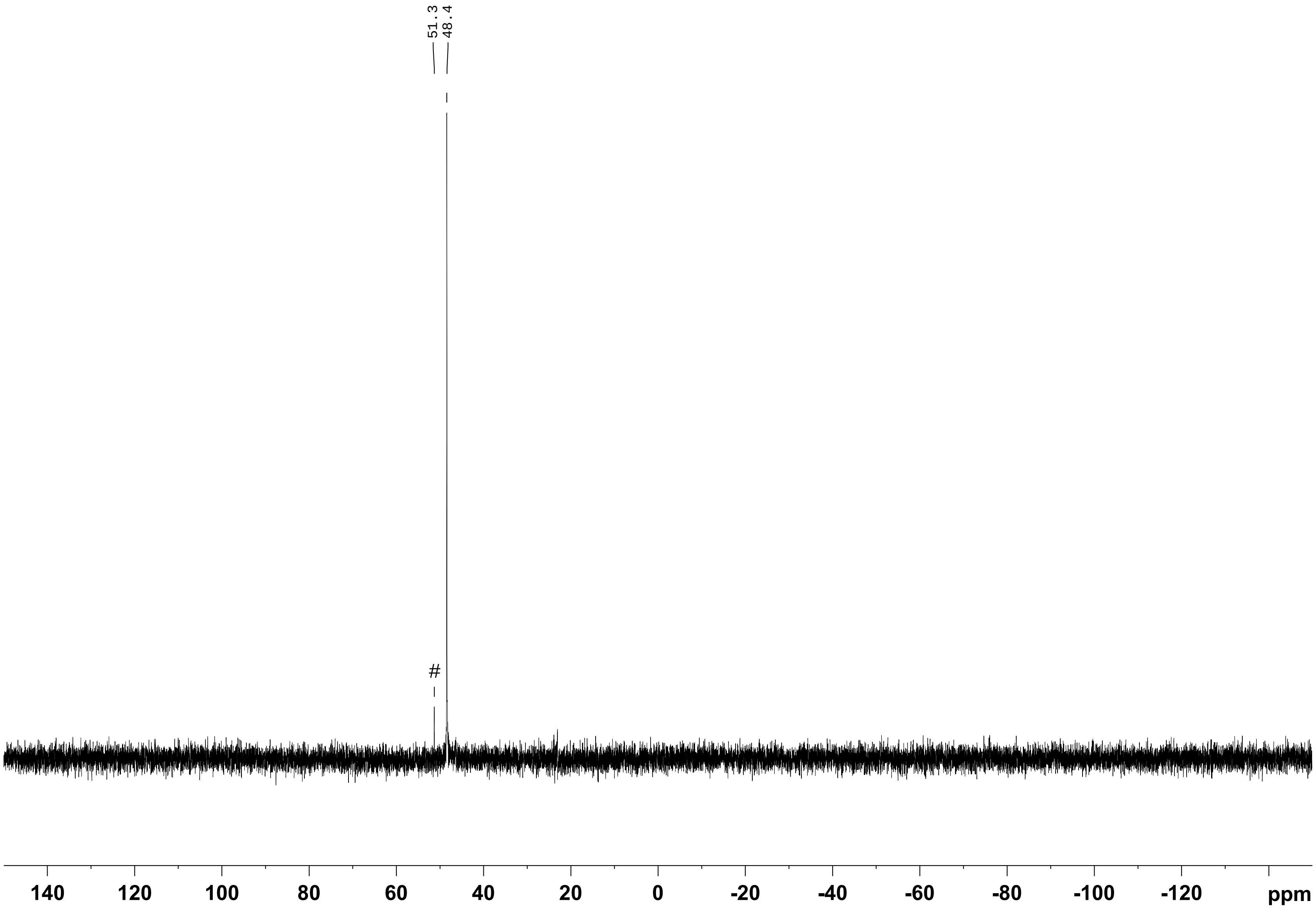

$^1H,^{29}Si$ HMQC NMR (500/99 MHz, CD_2Cl_2, 300 K, optimized for J = 8 Hz): * = Et_3SiH

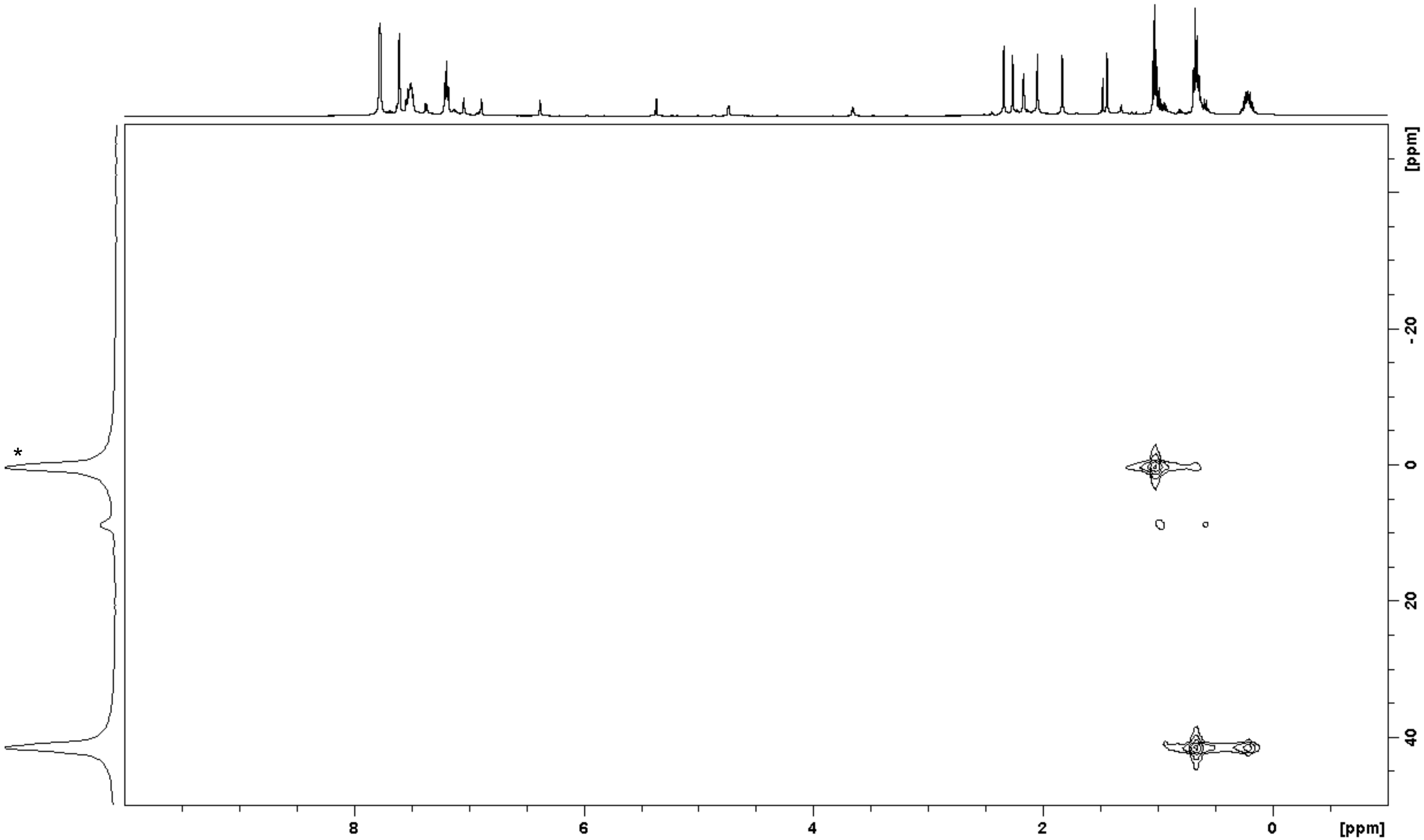

$^{1}H,^{31}P$ HMQC NMR (500/203 MHz, CD_2Cl_2, 300 K, optimized for J = 7 Hz): # = $[(p\text{-}FC_6H_4)_3POSiEt_3]^+[BAr^F{}_4]^-$

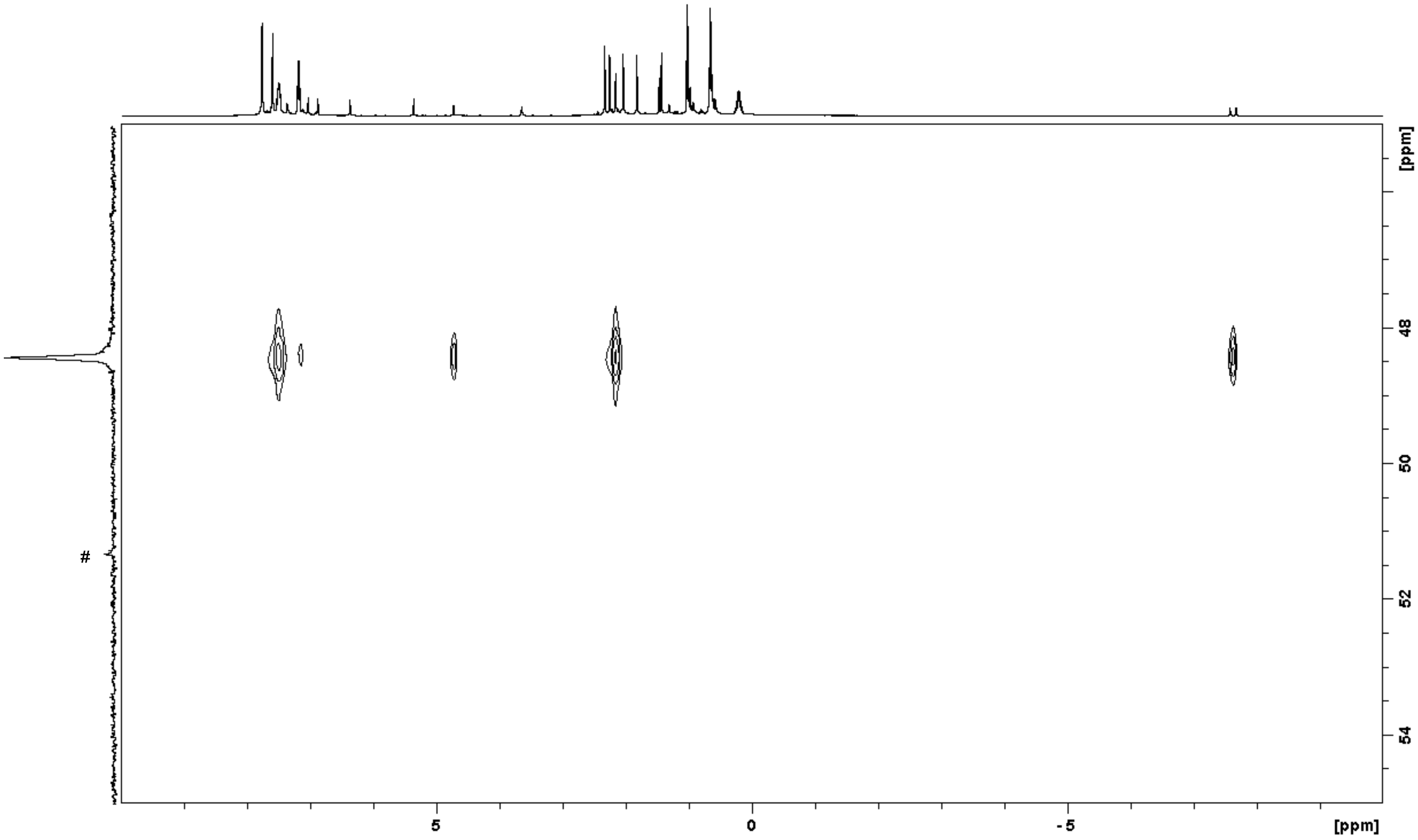

[{(p-$FC_6H_4)_3P$}Ru(SDmp)·iPrMePhSiH]$^+$[BAr$^F{}_4$]$^-$ (**3be**)

^{1}H NMR (500 MHz, CD_2Cl_2, 300 K): * = iPrMePhSiH

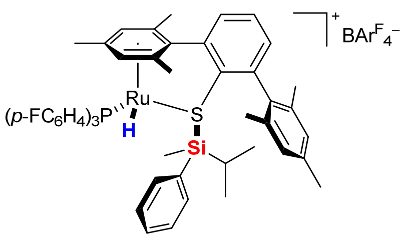

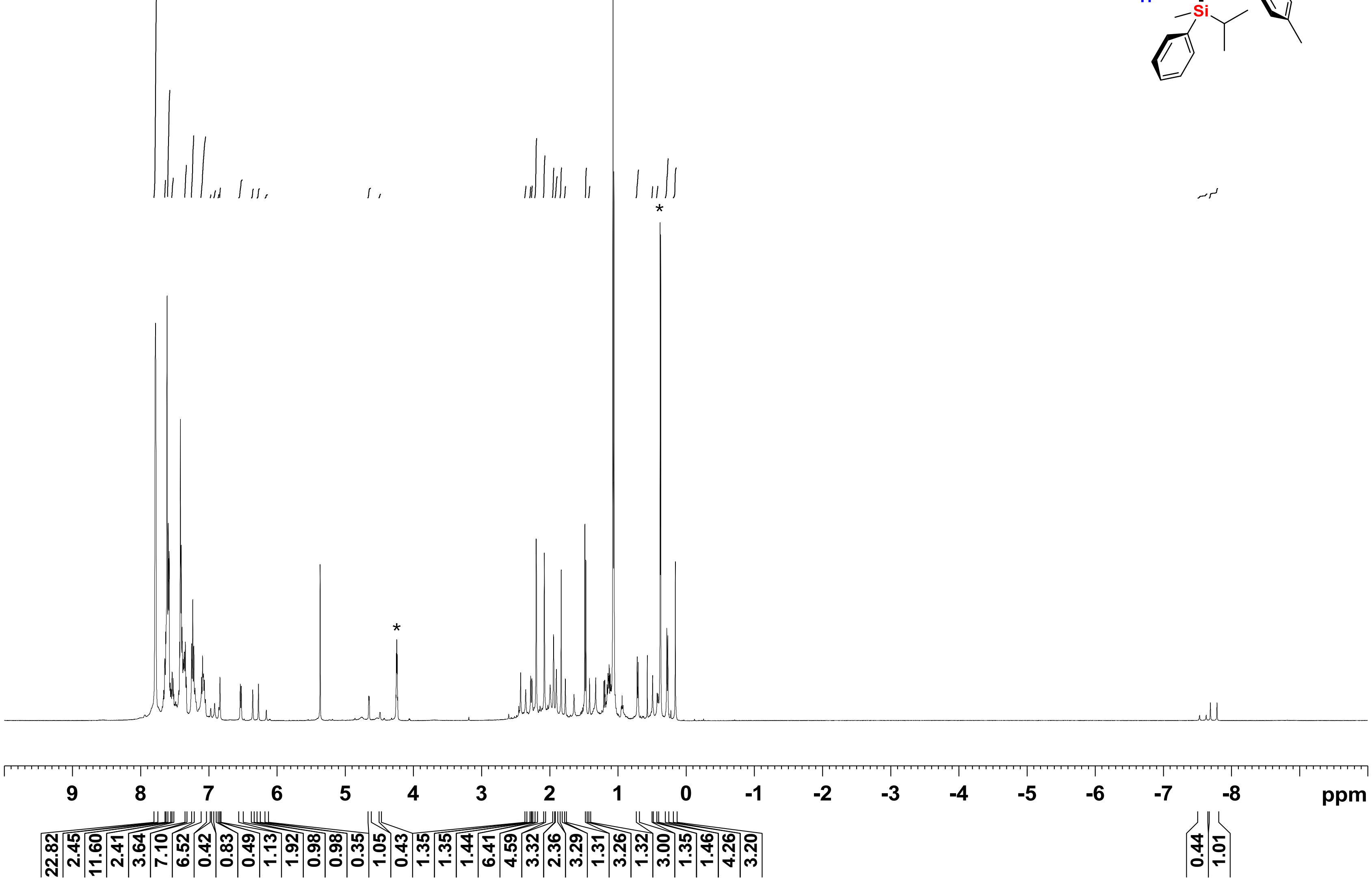

^{11}B NMR (161 MHz, CD_2Cl_2, 300 K):

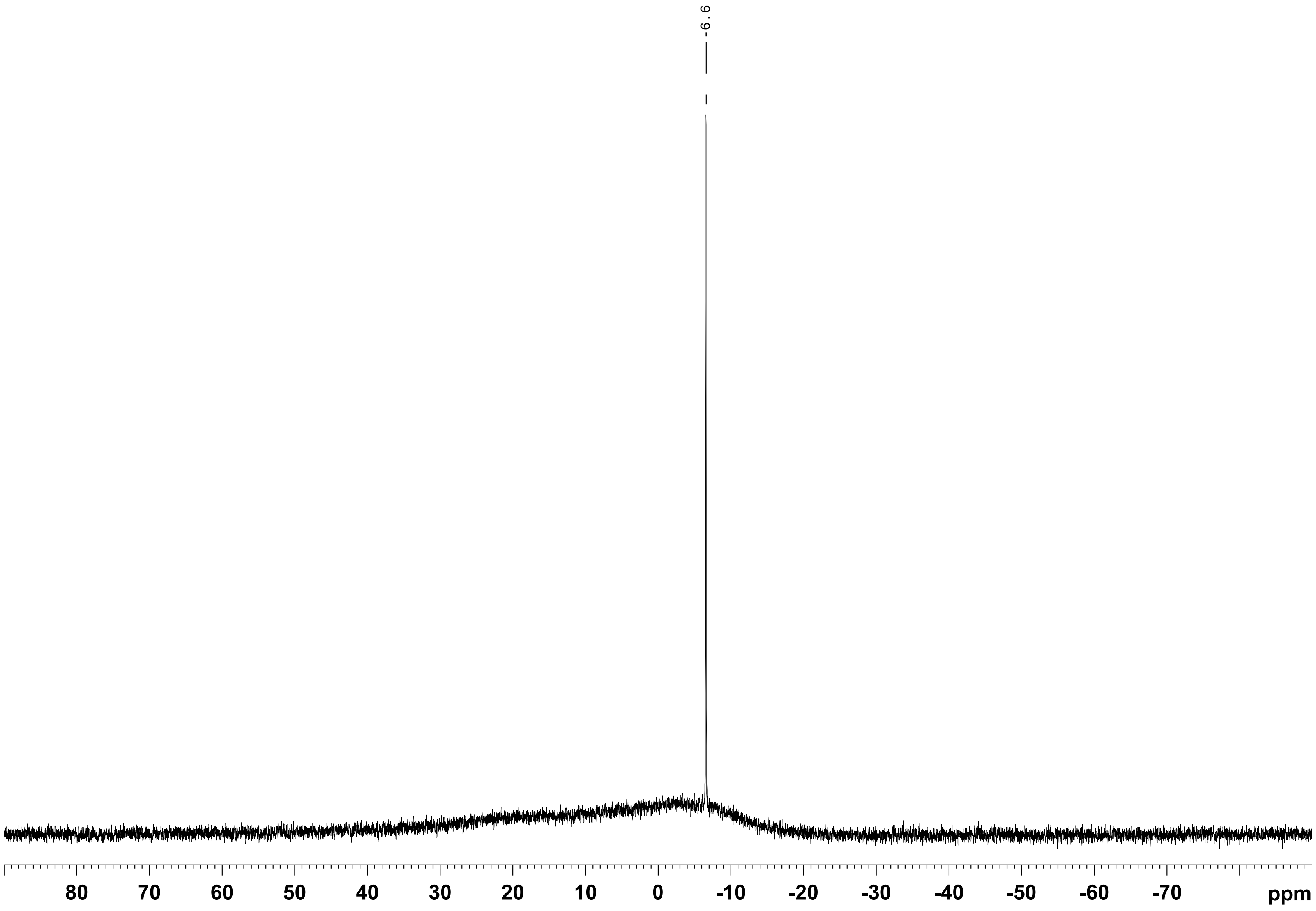

$^{19}F\{^1H\}$ NMR (471 MHz, CD_2Cl_2, 300 K): # = $[(p\text{-}FC_6H_4)_3POSi\mathit{i}PrMePh]^+[BAr^F{}_4]^-$

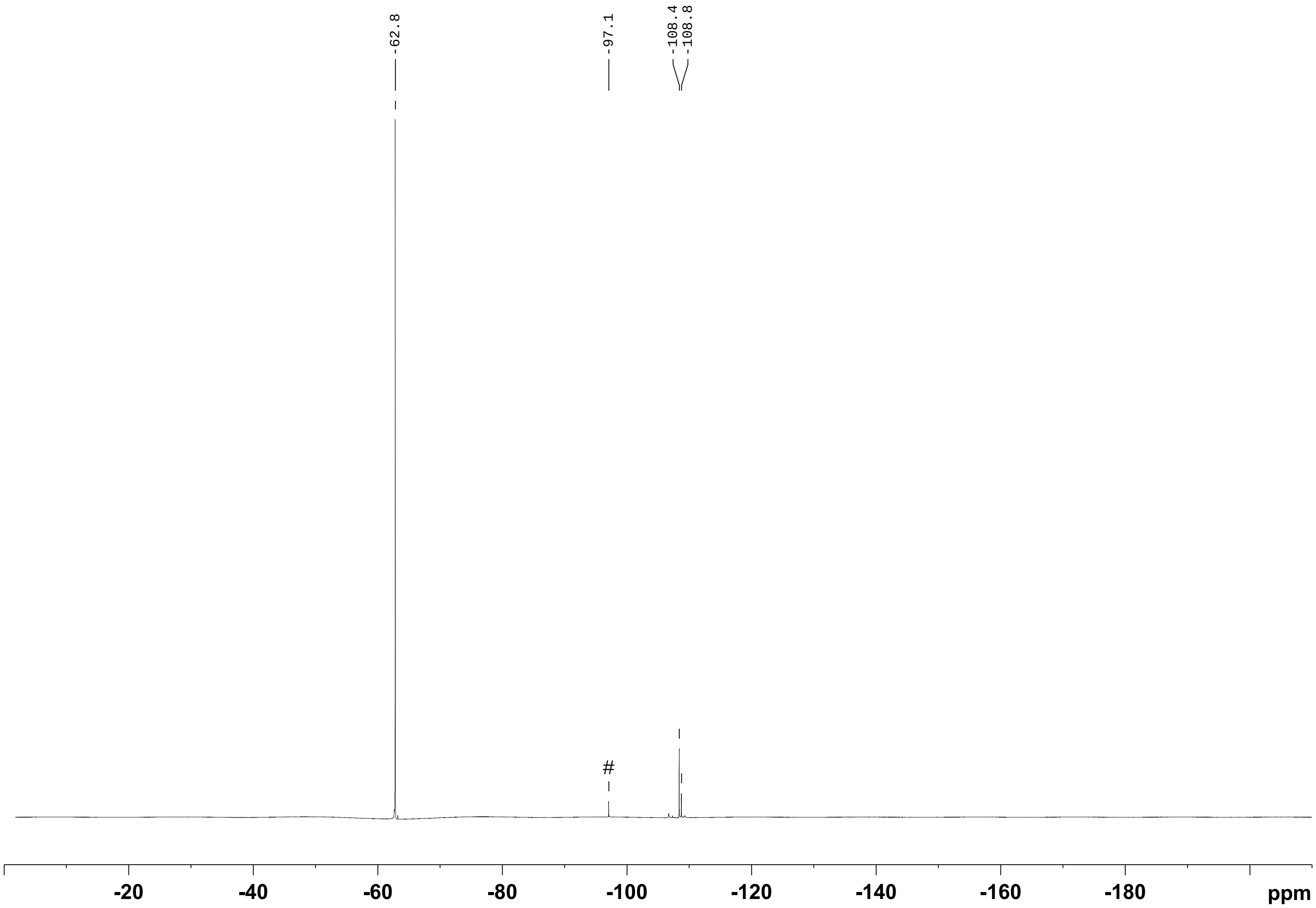

$^{31}P\{^{1}H\}$ NMR (203 MHz, CD_2Cl_2, 300 K): # = [(*p*-$FC_6H_4)_3$POS*i*PrMePh]$^{+}$[$BAr^{F}{}_4$]$^{-}$

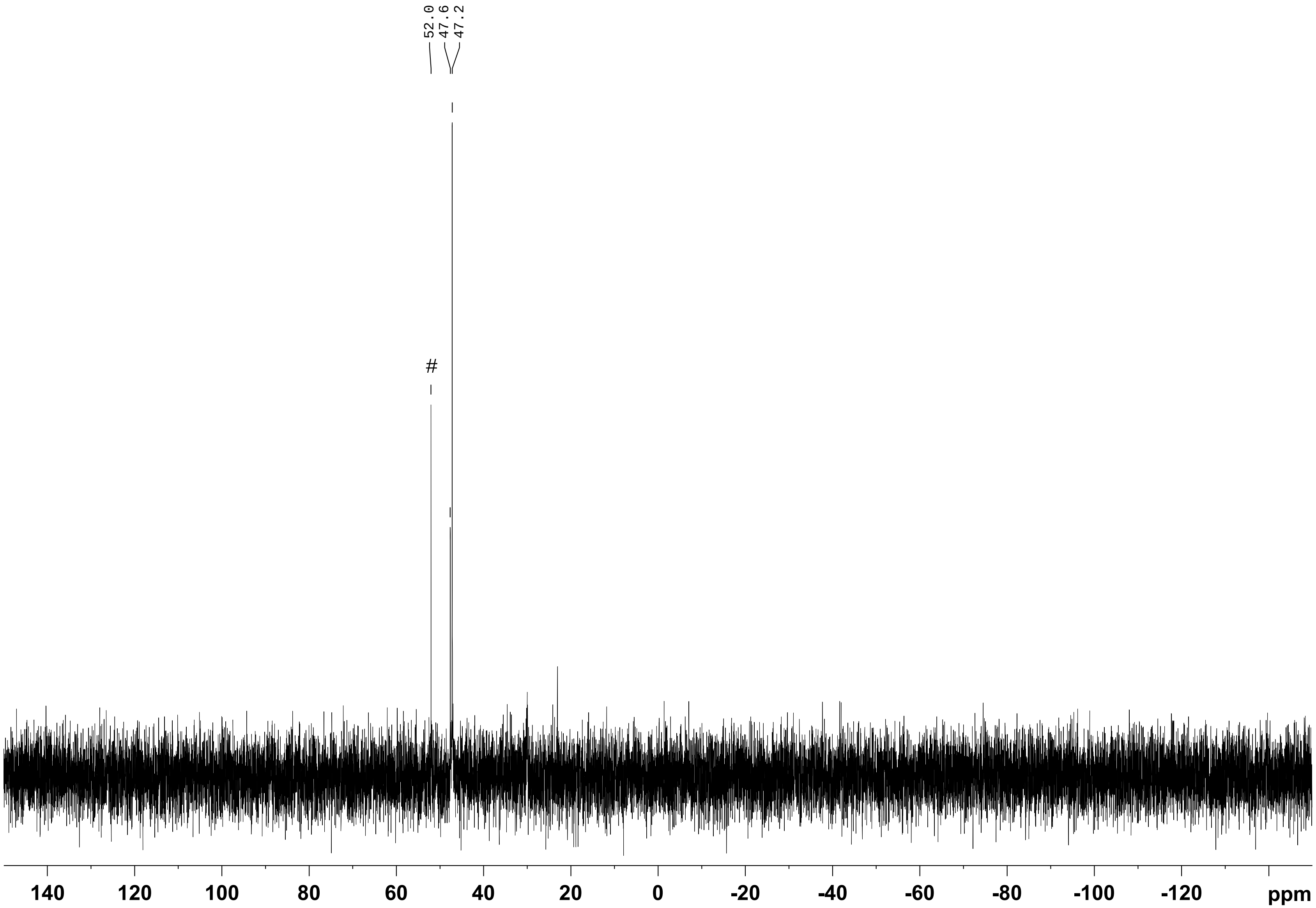

$^{1}H,^{29}Si$ HMQC NMR (500/99 MHz, CD_2Cl_2, 300 K, optimized for J = 8 Hz): * = *i*PrMePhSiH, # = [(*p*-$FC_6H_4)_3$POS*i*PrMePh]$^+$[BAr$^F{}_4$]$^-$

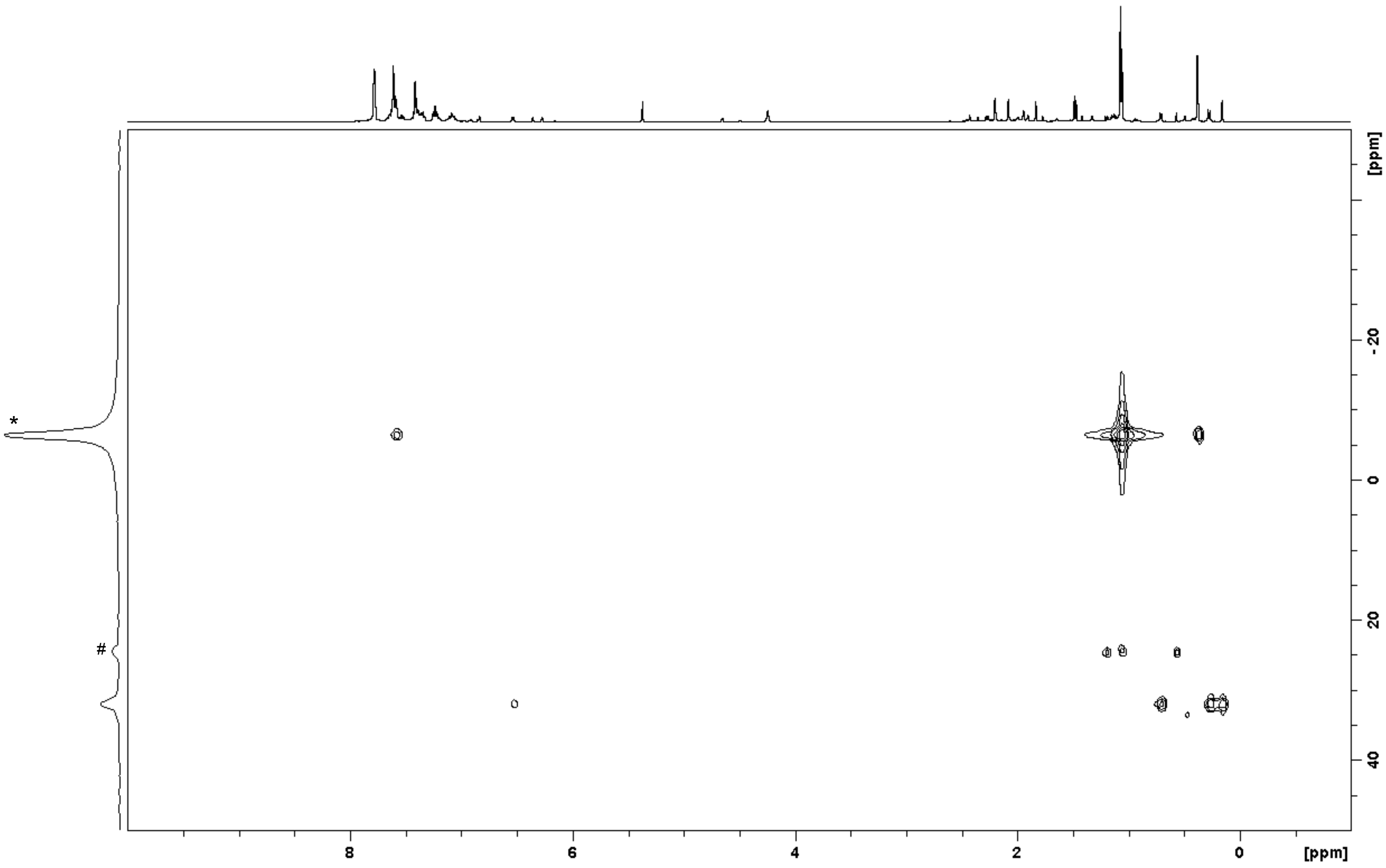

$^{1}H,^{31}P$ HMQC NMR (500/203 MHz, CD_2Cl_2, 300 K, optimized for J = 7 Hz): # = $[(p\text{-}FC_6H_4)_3POS\mathit{i}PrMePh]^+[BAr^F{}_4]^-$

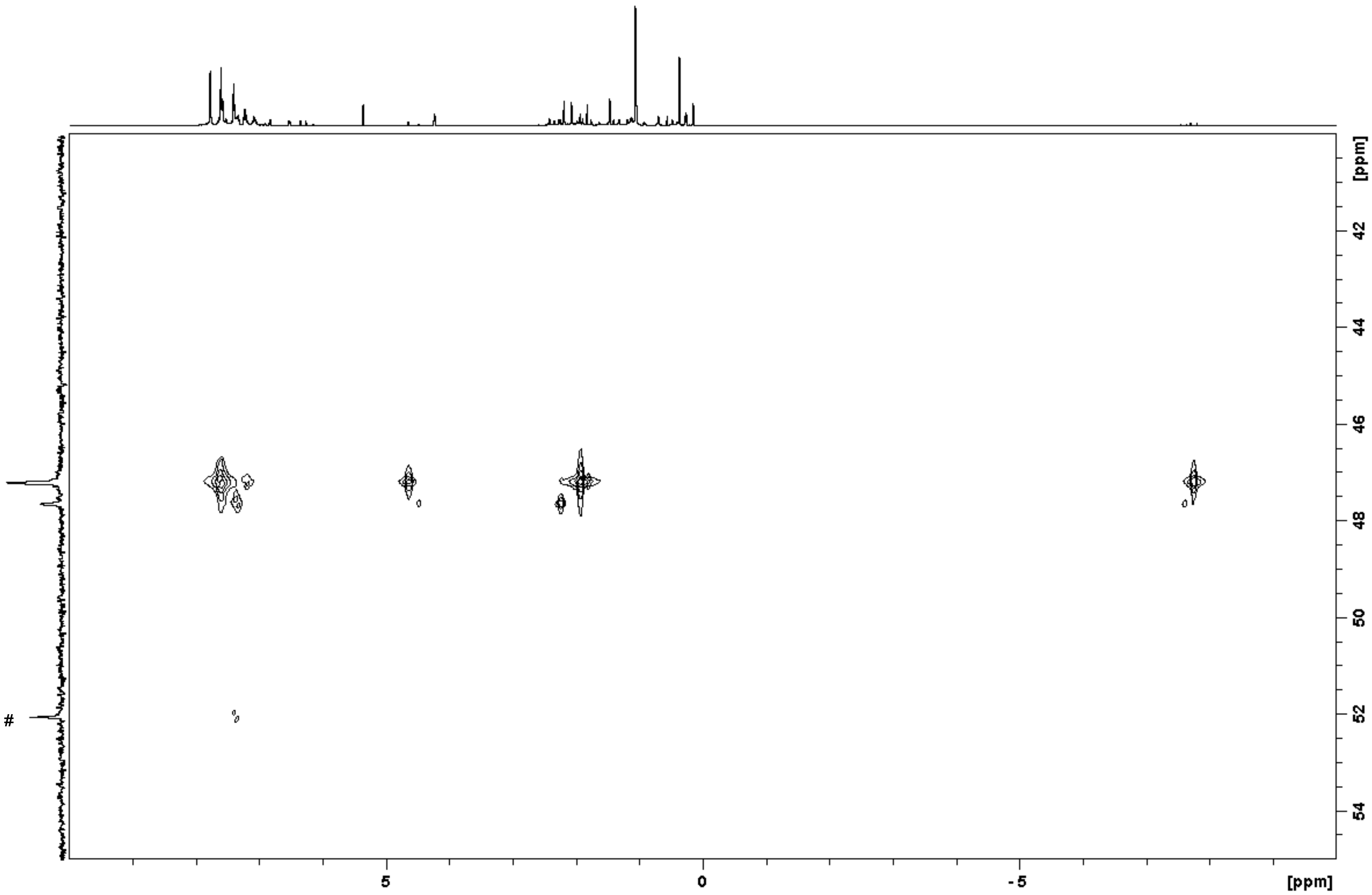

6.2 NMR Spectra of $[Et_3POSiMePh_2]^+[BAr^F{}_4]^-$ (10aa)

^{1}H NMR (500 MHz, C_6D_6, 300 K): * = $MePh_2SiH$, # = $[\{(p\text{-}FC_6H_4)_3P\}RuH(SDmp)]$

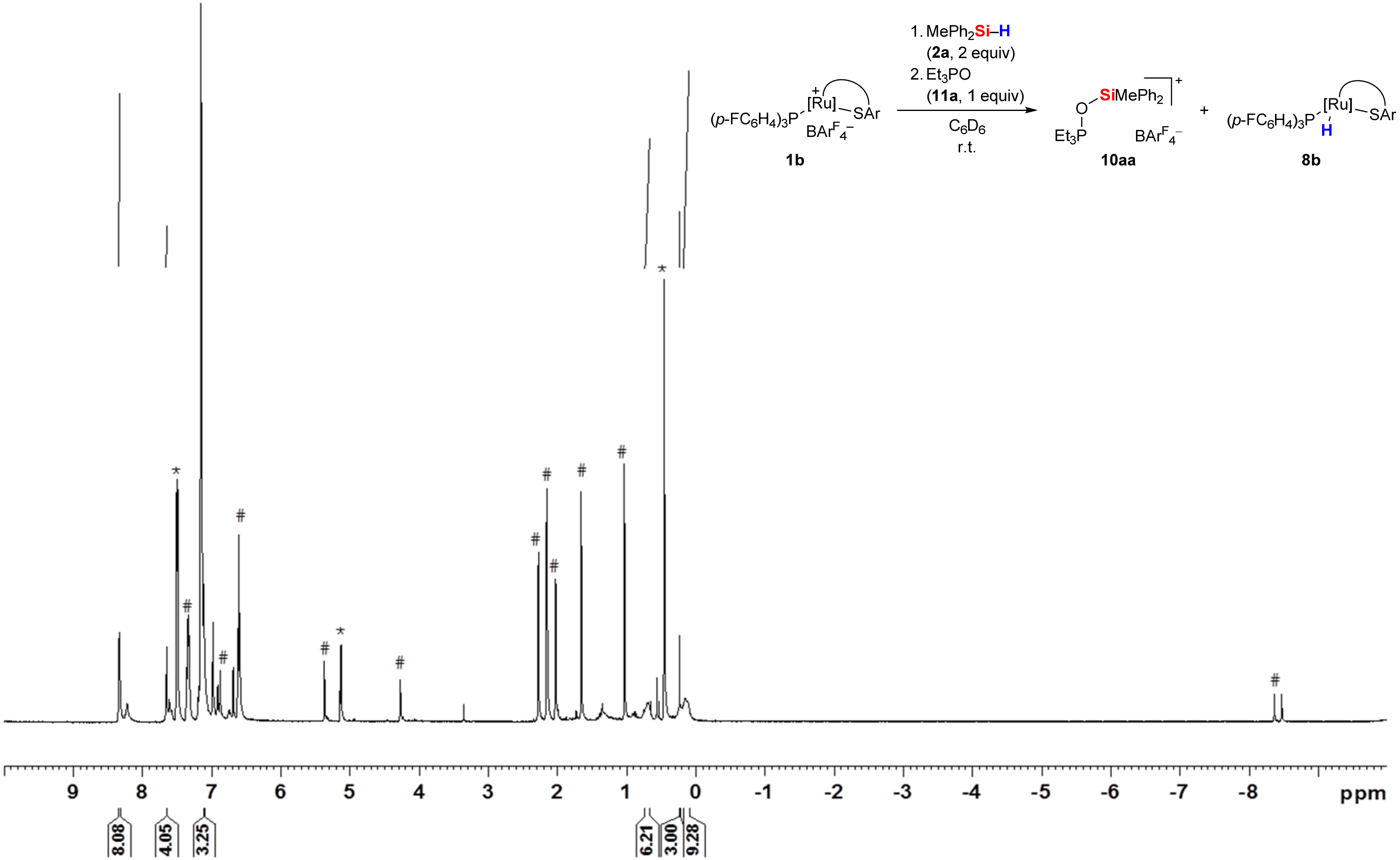

$^{31}P\{^{1}H\}$ NMR (203 MHz, C_6D_6, 300 K):

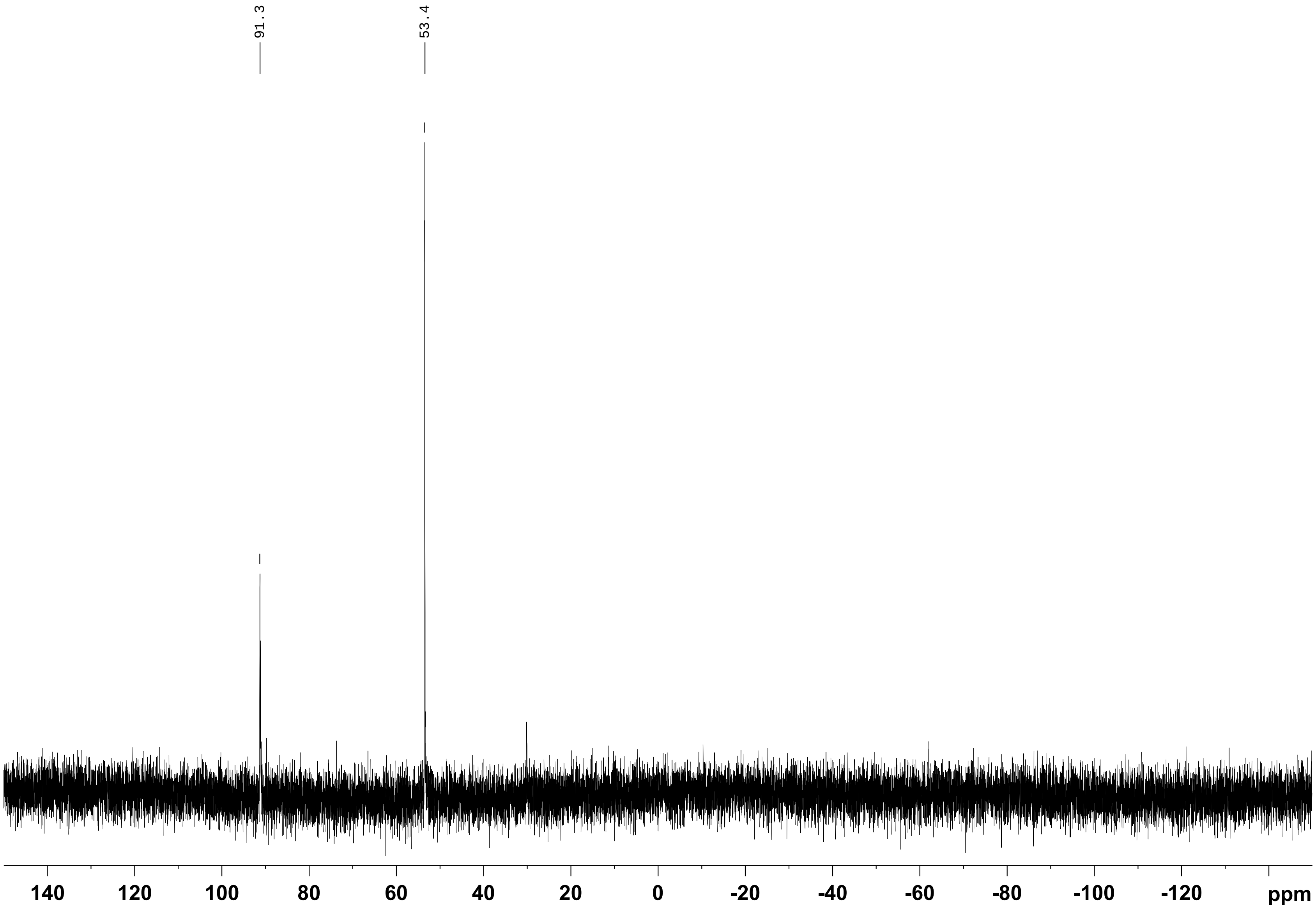

$^{1}H,^{29}Si$ HMQC NMR (500/99 MHz, C_6D_6, 300 K, optimized for J = 7 Hz):

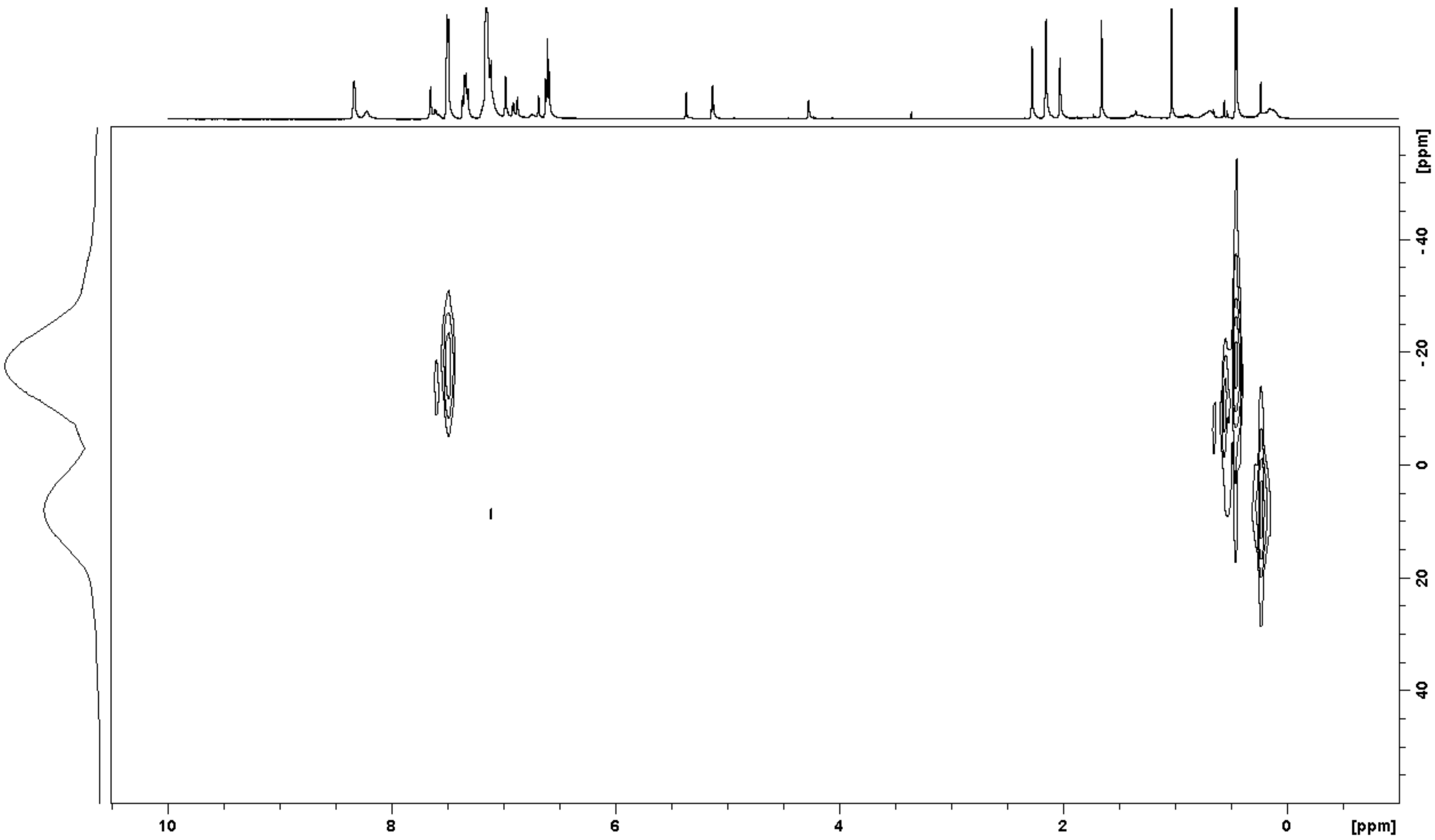

$^{1}H,^{31}P$ HMQC NMR (500/203 MHz, C_6D_6, 300 K, optimized for J = 7 Hz):

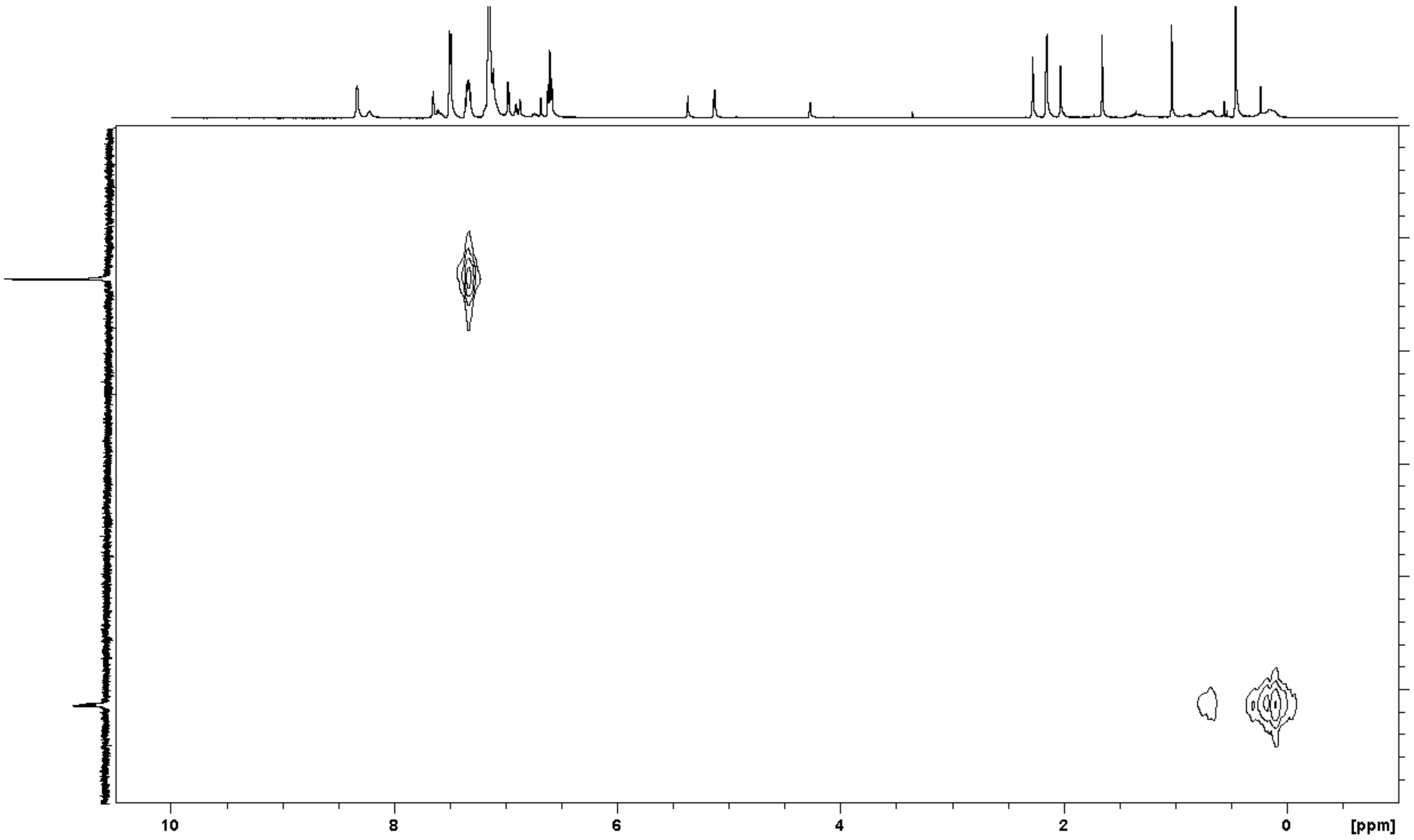

7 Crystallographic Data

Diffraction Data for the single-crystal structure analyses were collected on a Rigaku Saturn724 diffractometer at 173 K using multi-layer mirror monochromated Mo-K_{α} radiation (λ = 0.71075 Å). The crystallographic data were collected and processed using the CrystalClear software package.[S26] All calculations were performed using the CrystalStructure program package[S27] except for structure refinement, which was performed using SHELXL-97.[S28] Ortep-3 was used for the structure visualization.[S29]

7.1 Molecular Structure of $[(Et_3P)Ru(SDmp)\cdot Me_2PhSiH]^+[BAr^F{}_4]^-$ (3ab)

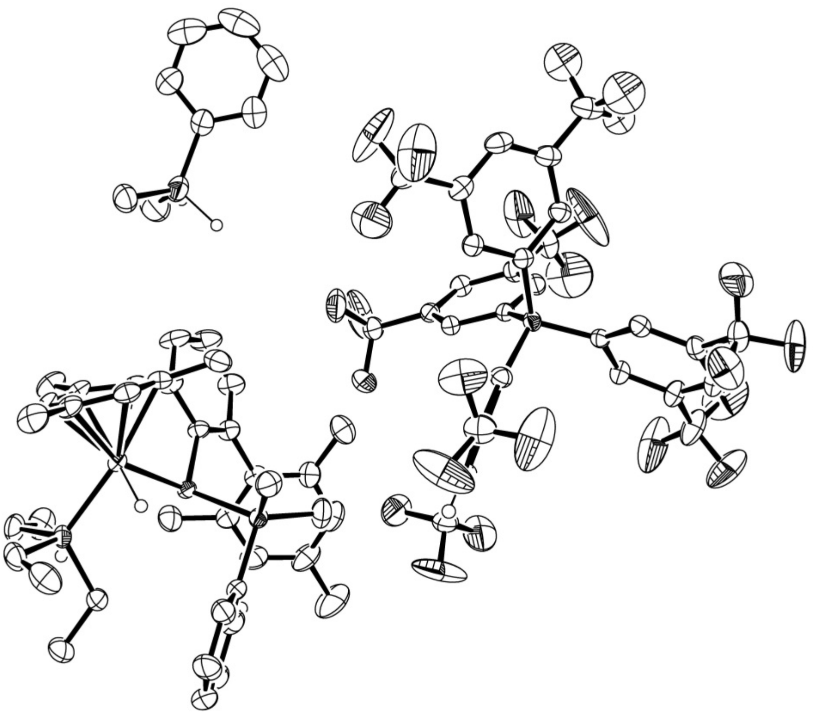

Table S5. Crystal data and structure refinement for **3ab**.

Empirical Formula	$C_{78}H_{76}BF_{24}PRuSSi_2$ (**3ab**·Me_2PhSiH)
Formula weight	1700.50 g mol^{-1}
Description and size of the crystal	yellow block, 0.240 × 0.170 × 0.070 mm
Crystal system	triclinic
Space group	P-1 (No.2)
Lattice parameters	a = 13.183(2) Å
	b = 16.891(2) Å
	c = 20.072(3) Å
	α = 67.256(7)°
	β = 76.552(8)°
	γ = 73.916(8)°
	V = 3920.8(9) $Å^3$
Z	2
Density (calculated)	1.440 g cm^{-3}
Absorption coefficient μ(Mo-K_α)	3.768 cm^{-1}
$2\theta_{max}$	55.0°
No. of collected reflections	48815
No. of independent reflections	17887 (R_{int} = 0.0393)
No. of observed reflections	17887
No. of refined parameters	972
$R1$ [$I > 2\sigma(I)$]	0.0607
$wR2$ (all reflections)	0.1722
Goodness of fit (GOF)	1.077

7.2 Molecular Structure of $[(Et_3P)Ru(SDmp)\cdot EtMe_2SiH]^+[BAr^F{}_4]^-$ (3ad)

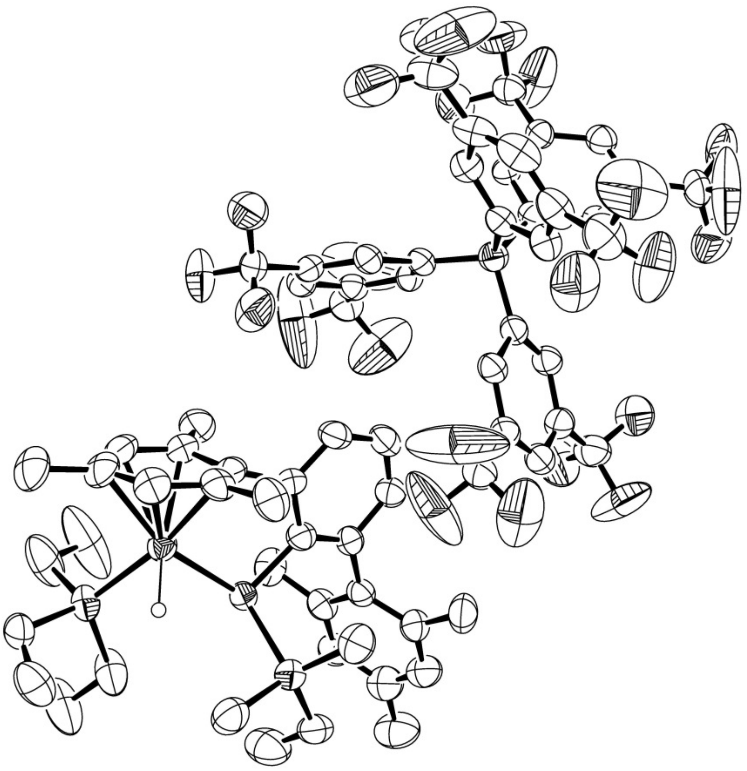

Table S6. Crystal data and structure refinement for **3ad**.

Empirical Formula	$C_{66}H_{64}BF_{24}PRuSSi$
Formula weight	1516.19 g mol^{-1}
Description and size of the crystal	yellow block, 0.070 × 0.070 × 0.020 mm
Crystal system	monoclinic
Space group	$P2_1/n$ (No.14)
Lattice parameters	a = 13.560(3) Å
	b = 27.705(4) Å
	c = 18.609(4) Å
	β = 102.006(3)°
	V = 6838(3) Å^3
Z	4
Density (calculated)	1.473 g cm^{-3}
Absorption coefficient μ(Mo-K_α)	4.055 cm^{-1}
$2\theta_{max}$	55.0°
No. of collected reflections	56447
No. of independent reflections	15660 (R_{int} = 0.0715)
No. of observed reflections	15660
No. of refined parameters	860
$R1$ [$I > 2\sigma(I)$]	0.0835
$wR2$ (all reflections)	0.2803
Goodness of fit (GOF)	1.045

7.3 Molecular Structure of $[Et_3POSiMe_2Ph]^+[BAr^F{}_4]^-$ (10ab)

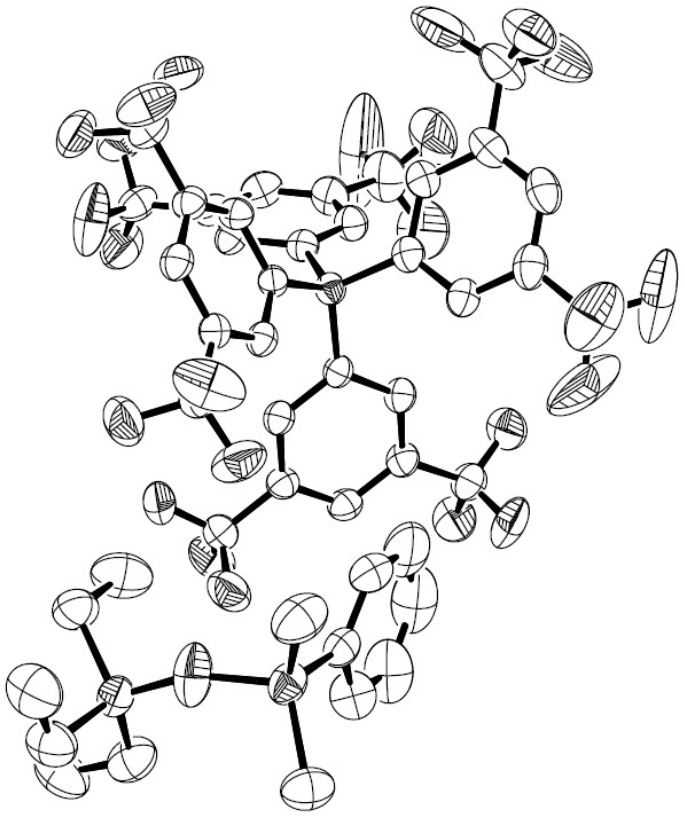

Table S7. Crystal data and structure refinement for **10ab**.

Empirical Formula	$C_{46}H_{38}BF_{24}OPSi$
Formula weight	1132.64 g mol^{-1}
Description and size of the crystal	colorless block, 0.100 × 0.100 × 0.070 mm
Crystal system	monoclinic
Space group	$P2_1/c$ (No.14)
Lattice parameters	a = 16.929(3) Å
	b = 18.161(3) Å
	c = 16.113(3) Å
	β = 97.045(4)°
	V = 4916(2) Å^3
Z	4
Density (calculated)	1.530 g cm^{-3}
Absorption coefficient μ(Mo-K_α)	2.070 cm^{-1}
$2\theta_{max}$	55.0°
No. of collected reflections	40288
No. of independent reflections	10995 (R_{int} = 0.0473)
No. of observed reflections	10995
No. of refined parameters	667
$R1$ [$I > 2\sigma(I)$]	0.0949
$wR2$ (all reflections)	0.3038
Goodness of fit (GOF)	1.096

8 Optimized Cartesian Coordinates of Selected Ruthenium(II)–Thiolate Complexes

[(Et$_3$P)RuSDmp]$^+$ (**1a**$^+$)

Ru	1.52015	-0.36860	0.10624
S	-0.62639	0.27110	0.15502
P	2.25196	1.90542	0.08158
C	-1.46036	-1.31123	-0.06965
C	-2.86545	-1.34558	-0.11298
C	-3.49646	-2.58716	-0.28354
C	-2.75875	-3.76335	-0.41206
C	-1.36071	-3.71980	-0.37922
C	-0.70821	-2.50040	-0.21423
C	0.78385	-2.38500	-0.18588
C	1.46519	-2.36699	1.06943
C	0.77078	-2.73524	2.34906
C	2.82508	-1.90600	1.12117
C	3.55246	-1.54954	-0.03945
C	4.99958	-1.15833	0.04128
C	2.84852	-1.53802	-1.26958
C	1.47936	-1.95420	-1.37513
C	0.80128	-1.92759	-2.71398
C	-3.66359	-0.08855	0.00910
C	-3.96188	0.66262	-1.14454
C	-3.47510	0.22109	-2.50351
C	-4.69252	1.84841	-1.00886
C	-5.13316	2.30416	0.23855
C	-5.94706	3.56910	0.35880
C	-4.81775	1.54311	1.37063
C	-4.09047	0.35181	1.27749
C	-3.74242	-0.42627	2.52343
C	2.14587	2.68939	1.75285
C	2.93863	1.93876	2.82528
C	1.23732	3.02804	-0.97518
C	1.22202	2.62434	-2.45091
C	3.99684	2.16523	-0.46918
C	4.45641	3.62063	-0.59697
H	-4.58144	-2.61953	-0.31644
H	-3.26843	-4.71252	-0.54396
H	-0.77917	-4.63054	-0.48769
H	1.23477	-2.23380	3.20169
H	0.85373	-3.81772	2.50740

H	-0.29156	-2.48920	2.32371
H	3.30203	-1.82185	2.09153
H	5.29002	-0.51071	-0.78812
H	5.62055	-2.06094	-0.00817
H	5.22662	-0.65440	0.98416
H	3.34369	-1.17135	-2.16196
H	-0.25176	-1.65503	-2.63496
H	0.84981	-2.92834	-3.16133
H	1.30126	-1.23045	-3.39007
H	-2.37901	0.22884	-2.55089
H	-3.84745	0.88667	-3.28679
H	-3.79902	-0.79791	-2.74129
H	-4.92215	2.42882	-1.89956
H	-5.71531	4.10917	1.28260
H	-7.02141	3.34606	0.37431
H	-5.76525	4.24287	-0.48412
H	-5.14432	1.88418	2.35049
H	-4.08208	-1.46589	2.46340
H	-4.19911	0.02840	3.40657
H	-2.65739	-0.45274	2.68294
H	2.48126	3.72957	1.67377
H	1.08137	2.71505	2.01016
H	2.57805	0.91027	2.93177
H	2.83201	2.43548	3.79472
H	4.00763	1.90221	2.58868
H	0.22238	3.00865	-0.56471
H	1.61294	4.04934	-0.84969
H	2.22473	2.64829	-2.89068
H	0.59288	3.31169	-3.02501
H	0.81771	1.61544	-2.57789
H	4.62595	1.62998	0.24782
H	4.10231	1.64324	-1.42587
H	3.89372	4.16230	-1.36246
H	5.51306	3.65044	-0.88297
H	4.35683	4.16499	0.34701

[(Ar$^{F}{}_{3}$P)RuSDmp]$^{+}$ (**1b**$^{+}$)

Ru	-0.57580	-1.39918	0.35732
S	1.36329	-0.27307	0.33962
P	-1.80094	0.61401	0.00322
C	2.54652	-1.62121	0.19834
C	3.91548	-1.32042	0.08370

C	4.82474	-2.38515	-0.00897
C	4.39148	-3.71042	0.00123
C	3.02512	-4.00082	0.08708
C	2.10110	-2.96243	0.17658
C	0.62339	-3.19688	0.21032
C	-0.13099	-3.02108	-1.01077
C	0.55198	-2.93091	-2.34390
C	-1.55998	-2.94333	-0.93856
C	-2.26576	-3.05036	0.28687
C	-3.76344	-3.00934	0.32615
C	-1.49815	-3.14052	1.47122
C	-0.06620	-3.25885	1.45773
C	0.67248	-3.35232	2.76153
C	4.38783	0.09542	0.03796
C	4.90073	0.70248	1.20135
C	4.96627	-0.05483	2.50618
C	5.33304	2.03148	1.13578
C	5.27249	2.76876	-0.05244
C	5.78573	4.18610	-0.11065
C	4.75067	2.14647	-1.19180
C	4.30675	0.81995	-1.16837
C	3.72660	0.19872	-2.41618
H	5.88301	-2.15869	-0.09773
H	5.11311	-4.51795	-0.07184
H	2.67950	-5.03017	0.07222
H	-0.10120	-2.46871	-3.08711
H	0.79475	-3.94310	-2.69062
H	1.48631	-2.37080	-2.28954
H	-2.11343	-2.76777	-1.85501
H	-4.12698	-2.61254	1.27571
H	-4.15402	-4.02849	0.21688
H	-4.17065	-2.40513	-0.48639
H	-2.00160	-3.11405	2.43159
H	1.62894	-2.82869	2.72476
H	0.88170	-4.40546	2.98655
H	0.07169	-2.94433	3.57806
H	3.99010	-0.47458	2.77540
H	5.28861	0.59963	3.32047
H	5.66915	-0.89415	2.45425
H	5.72583	2.50221	2.03411
H	5.64925	4.70159	0.84533
H	5.27679	4.76467	-0.88782
H	6.85893	4.20347	-0.34011
H	4.68982	2.70435	-2.12351

H	4.15309	-0.78976	-2.61586
H	3.91372	0.83210	-3.28735
H	2.64033	0.07542	-2.32557
C	-3.57902	0.40669	-0.39106
C	-4.03727	0.34058	-1.71437
C	-4.49501	0.23960	0.66093
C	-5.38582	0.10533	-1.99055
C	-5.84562	0.01444	0.40210
C	-6.26007	-0.05413	-0.92314
H	-3.35176	0.48712	-2.54137
H	-4.15980	0.29330	1.69174
H	-5.75717	0.06008	-3.00857
H	-6.56608	-0.10654	1.20372
C	-1.81867	1.80233	1.39502
C	-2.72063	2.88200	1.40214
C	-0.92265	1.65254	2.46307
C	-2.72560	3.79755	2.45180
C	-0.91393	2.56212	3.52170
C	-1.81678	3.61627	3.48957
H	-3.42834	3.00992	0.59011
H	-0.22260	0.82506	2.47555
H	-3.41500	4.63466	2.47279
H	-0.22521	2.46033	4.35313
C	-1.07594	1.52170	-1.41702
C	-0.72671	0.78677	-2.56262
C	-0.78311	2.89104	-1.37037
C	-0.12349	1.40352	-3.65524
C	-0.16351	3.52304	-2.45093
C	0.14878	2.76486	-3.57031
H	-0.90293	-0.28327	-2.59228
H	-1.01379	3.47459	-0.48732
H	0.15203	0.84817	-4.54522
H	0.08234	4.57890	-2.42365
F	-7.57214	-0.27968	-1.18339
F	0.75935	3.36959	-4.62027
F	-1.82041	4.50243	4.51521

[$(Et_3P)Ru(\eta^2$-$HSiMePh_2)SDmp]^+$ (**6'aa**$^+$)

Ru	-0.97944	-0.97165	0.21031
S	1.18841	-0.02840	-0.20287
P	-0.66591	-0.25241	2.41320
Si	-1.78008	1.16369	-1.12128

C	2.14703	-1.48738	-0.57002
C	3.53731	-1.35571	-0.79805
C	4.29264	-2.51126	-1.02715
C	3.70797	-3.78086	-1.04117
C	2.33281	-3.90602	-0.84248
C	1.55377	-2.76765	-0.61787
C	4.18720	-0.00912	-0.77827
C	4.94803	0.39403	0.33856
C	5.50087	1.68054	0.35836
C	5.33535	2.57199	-0.70591
C	4.60534	2.13984	-1.81888
C	4.03503	0.86405	-1.87740
C	5.20946	-0.53780	1.49857
C	5.96306	3.94407	-0.67506
C	3.27402	0.44515	-3.11108
C	0.06833	-2.83067	-0.51439
C	-0.69814	-2.37285	-1.65645
C	-2.07903	-2.15703	-1.49926
C	-2.76111	-2.42876	-0.27397
C	-1.99999	-2.95088	0.79365
C	-0.59833	-3.23125	0.67128
C	-0.01572	-2.15544	-2.97408
C	-4.24689	-2.24964	-0.16393
C	0.12106	-3.95944	1.76914
C	-0.94096	1.06741	-2.79941
C	-3.64495	1.07060	-1.39168
C	-1.26038	2.81400	-0.38124
C	-2.16009	3.66861	0.28187
C	-1.73279	4.87456	0.84317
C	-0.39052	5.25296	0.75209
C	0.51471	4.42980	0.07691
C	0.08173	3.22865	-0.48791
C	-0.27592	1.54110	2.56794
C	0.04297	2.05232	3.97540
C	0.57963	-1.09621	3.48447
C	2.03225	-1.05898	3.02056
C	-2.16862	-0.50346	3.46345
C	-3.43042	0.20591	2.97937
H	-1.90965	0.35090	0.41925
H	5.36072	-2.40594	-1.19464
H	4.31653	-4.66146	-1.21975
H	1.85902	-4.88285	-0.88004
H	6.07628	1.99036	1.22813
H	4.48127	2.81005	-2.66658

H	6.08214	-1.17308	1.30072
H	4.36866	-1.20525	1.69082
H	5.42005	0.02636	2.41206
H	6.99252	3.91408	-1.05433
H	6.00428	4.34310	0.34359
H	5.40757	4.65267	-1.29745
H	3.58713	-0.54252	-3.46484
H	3.42657	1.16120	-3.92335
H	2.19911	0.39277	-2.90994
H	-2.64903	-1.76615	-2.33505
H	-2.49574	-3.18227	1.73054
H	0.70857	-1.33984	-2.92515
H	-0.74050	-1.93719	-3.75997
H	0.53658	-3.05833	-3.25371
H	-4.59581	-1.41523	-0.77203
H	-4.55373	-2.08462	0.87154
H	-4.74796	-3.15824	-0.52022
H	-0.42636	-3.90479	2.71138
H	1.13778	-3.59421	1.91218
H	0.19017	-5.01924	1.49594
H	0.14351	1.05443	-2.68916
H	-1.21770	1.98035	-3.34123
H	-1.23670	0.21027	-3.40391
H	-3.20960	3.40505	0.35500
H	-2.44802	5.51997	1.34532
H	-0.05602	6.18855	1.19088
H	1.55760	4.72018	-0.01229
H	0.80679	2.60696	-1.00175
H	0.55590	1.73972	1.88918
H	-1.13326	2.07742	2.15522
H	-0.76000	1.84179	4.68925
H	0.17023	3.13897	3.93760
H	0.97159	1.62911	4.36868
H	0.47607	-0.61550	4.46371
H	0.23988	-2.12465	3.62185
H	2.16244	-1.58537	2.07489
H	2.67157	-1.53498	3.77176
H	2.38690	-0.03607	2.87385
H	-2.33581	-1.58465	3.52022
H	-1.90637	-0.17480	4.47520
H	-3.26918	1.28306	2.86914
H	-4.24464	0.06690	3.69806
H	-3.76208	-0.18915	2.01695
C	-4.15793	0.77895	-2.66929

C	-5.53362	0.67871	-2.89690
C	-6.43297	0.87742	-1.84679
C	-5.94730	1.17932	-0.57122
C	-4.57180	1.26838	-0.35124
H	-3.48234	0.62793	-3.50518
H	-5.90077	0.45060	-3.89344
H	-7.50211	0.80243	-2.02156
H	-6.63851	1.34048	0.25107
H	-4.22342	1.48468	0.65191

[$(Et_3P)Ru(\eta^1$-$HSiMe_2Ph)SDmp]^+$ (**6ab**$^+$)

Ru	-1.57173	-0.66608	-0.16141
S	0.77109	-0.17215	-0.14502
P	-1.67506	0.36552	1.92907
Si	-1.63320	1.61194	-1.87490
C	1.45915	-1.81431	-0.16433
C	2.86171	-1.97314	-0.08445
C	3.39018	-3.26655	-0.01845
C	2.56597	-4.39610	-0.04501
C	1.18475	-4.23784	-0.16816
C	0.63260	-2.95586	-0.23892
C	3.75011	-0.77093	-0.08204
C	4.28022	-0.27728	1.12705
C	5.06299	0.88295	1.10405
C	5.34262	1.56141	-0.08714
C	4.82052	1.04439	-1.27796
C	4.03002	-0.10985	-1.29661
C	4.03228	-0.98708	2.43612
C	6.22023	2.78898	-0.09212
C	3.48862	-0.62502	-2.60783
C	-0.82299	-2.72371	-0.47367
C	-1.23731	-2.30422	-1.79982
C	-2.53727	-1.79338	-1.95931
C	-3.48827	-1.75309	-0.88825
C	-3.08960	-2.28688	0.35504
C	-1.78058	-2.83382	0.57205
C	-0.27413	-2.39567	-2.94566
C	-4.88602	-1.24630	-1.10730
C	-1.45933	-3.54405	1.85465
C	-0.58159	0.93581	-3.26014
C	-3.36785	1.97863	-2.49565
C	-0.82962	3.12982	-1.14311

C	-1.60552	4.21264	-0.68822
C	-1.00716	5.32886	-0.09965
C	0.38268	5.37876	0.04649
C	1.17024	4.31986	-0.41433
C	0.56931	3.20943	-1.01007
C	-0.79502	1.97974	2.05313
C	-0.85389	2.67133	3.41753
C	-1.07829	-0.57184	3.40003
C	0.42020	-0.87655	3.39530
C	-3.41682	0.74843	2.41893
C	-4.14146	1.70596	1.47113
H	-2.10467	0.88444	-0.49849
H	4.46773	-3.38425	0.05275
H	2.99880	-5.38961	0.01169
H	0.53529	-5.10716	-0.22284
H	5.46336	1.26530	2.04060
H	5.03463	1.55034	-2.21675
H	4.68392	-1.86317	2.54258
H	3.00443	-1.34669	2.51468
H	4.23248	-0.32473	3.28339
H	7.28186	2.51219	-0.12030
H	6.06708	3.39425	0.80731
H	6.02319	3.41831	-0.96562
H	3.67208	-1.69746	-2.72926
H	3.94871	-0.10229	-3.45100
H	2.40525	-0.47291	-2.67080
H	-2.82942	-1.39980	-2.92824
H	-3.79403	-2.28830	1.18069
H	0.60139	-1.76297	-2.78353
H	-0.74978	-2.10768	-3.88441
H	0.08677	-3.42505	-3.04015
H	-4.90494	-0.37723	-1.76778
H	-5.36218	-0.97513	-0.16209
H	-5.49536	-2.02951	-1.57467
H	-2.19209	-3.31752	2.63027
H	-0.45764	-3.31682	2.21808
H	-1.50129	-4.62444	1.67300
H	0.37995	0.57217	-2.89704
H	-0.39941	1.76446	-3.95675
H	-1.08196	0.13701	-3.81003
H	-3.32311	2.79694	-3.22480
H	-4.06138	2.27109	-1.70205
H	-3.78642	1.10966	-3.01339
H	-2.68723	4.18911	-0.78945

H	-1.62161	6.15641	0.24317
H	0.84955	6.24329	0.50945
H	2.25069	4.35536	-0.30981
H	1.19732	2.39470	-1.35495
H	0.23990	1.79543	1.75459
H	-1.22103	2.62740	1.28678
H	-1.88090	2.87837	3.73502
H	-0.33258	3.63190	3.35393
H	-0.36695	2.08535	4.20231
H	-1.34643	0.02973	4.27579
H	-1.66569	-1.48944	3.46870
H	0.71071	-1.44731	2.51229
H	0.69067	-1.45319	4.28608
H	1.01532	0.04058	3.39589
H	-3.94577	-0.20897	2.47957
H	-3.39229	1.15949	3.43423
H	-3.61769	2.66398	1.39276
H	-5.15247	1.91094	1.83749
H	-4.22770	1.28414	0.46625

[(Ar$^{F}{}_{3}$P)Ru(η^{1}-HSiMePh$_2$)SDmp]$^{+}$ (**6ba**$^{+}$)

Ru	-0.63020	-0.50039	-1.24265
S	1.59092	0.13650	-0.65893
P	-1.04608	-1.02037	0.98544
C	2.51028	-1.16205	-1.45337
C	3.91181	-1.20387	-1.28454
C	4.61820	-2.28163	-1.82846
C	3.96964	-3.29374	-2.54517
C	2.59274	-3.21550	-2.76209
C	1.86106	-2.14938	-2.22983
C	0.41809	-1.92162	-2.54361
C	0.10958	-0.81916	-3.44667
C	1.20213	-0.14466	-4.22037
C	-1.21417	-0.37050	-3.52249
C	-2.29195	-1.00643	-2.82528
C	-3.69771	-0.49764	-2.96810
C	-1.98584	-2.15969	-2.08390
C	-0.64999	-2.67191	-1.96780
C	-0.46425	-3.99819	-1.28725
C	4.60737	-0.07802	-0.58365
C	4.76815	-0.08218	0.81510
C	4.26933	-1.23353	1.64797

C	5.36083	1.02317	1.43789
C	5.80125	2.13178	0.70943
C	6.45073	3.30888	1.39459
C	5.64541	2.11029	-0.68181
C	5.05848	1.02541	-1.34062
C	4.89435	1.05961	-2.84044
H	5.69486	-2.32129	-1.68965
H	4.53880	-4.12306	-2.95268
H	2.08649	-3.96926	-3.35897
H	0.79419	0.58019	-4.92623
H	1.78472	-0.88712	-4.77428
H	1.89613	0.37039	-3.55153
H	-1.43791	0.49856	-4.13157
H	-4.36536	-0.97899	-2.25204
H	-4.06935	-0.71590	-3.97641
H	-3.74779	0.58426	-2.82486
H	-2.77936	-2.67854	-1.55901
H	0.57938	-4.22317	-1.07978
H	-0.84805	-4.77518	-1.95983
H	-1.03662	-4.05829	-0.35871
H	3.17551	-1.25146	1.66168
H	4.61726	-1.15257	2.68154
H	4.60202	-2.19775	1.25177
H	5.47786	1.01545	2.51915
H	6.09081	4.25914	0.98501
H	7.53954	3.29326	1.26006
H	6.25203	3.30446	2.47059
H	5.98847	2.95968	-1.26863
H	5.31387	0.16881	-3.31894
H	5.38657	1.93839	-3.26606
H	3.83600	1.10097	-3.12147
H	-1.20542	0.96750	-0.60296
Si	-1.01301	2.42710	-1.21861
C	-2.71125	-1.78361	1.11173
C	-3.80111	-1.08695	0.56102
C	-2.92718	-3.05117	1.66851
C	-5.08511	-1.62407	0.58765
C	-4.20654	-3.61460	1.68708
C	-5.25840	-2.88472	1.15169
H	-3.64787	-0.12227	0.09180
H	-2.10394	-3.61495	2.08995
H	-5.93412	-1.09077	0.17371
H	-4.38577	-4.59707	2.11018
C	-1.08279	0.38211	2.16704

C	0.14566	0.98154	2.49701
C	-2.25123	0.81091	2.80853
C	0.21061	1.98328	3.45931
C	-2.20302	1.83063	3.76304
C	-0.97052	2.38958	4.06774
H	1.05743	0.65895	2.00649
H	-3.20441	0.34195	2.59657
H	1.14912	2.45758	3.71868
H	-3.09760	2.17515	4.27035
C	0.05604	-2.17196	1.89036
C	-0.15031	-2.37613	3.26790
C	1.09687	-2.84951	1.25187
C	0.64443	-3.26901	3.98194
C	1.89841	-3.75540	1.94838
C	1.64997	-3.94763	3.29882
H	-0.93220	-1.83511	3.79059
H	1.31131	-2.65532	0.21339
H	0.49874	-3.43714	5.04341
H	2.71322	-4.27947	1.46192
F	-6.50346	-3.42231	1.16891
F	-0.91690	3.37891	4.99670
F	2.42857	-4.81895	3.98850
C	-2.83869	2.77422	-1.42056
C	-3.74199	2.48045	-0.38135
C	-3.35129	3.30847	-2.61529
C	-5.11119	2.70171	-0.53261
C	-4.72258	3.53293	-2.77156
C	-5.60433	3.22486	-1.73281
H	-3.37133	2.08246	0.55958
H	-2.68119	3.55458	-3.43473
H	-5.79163	2.47210	0.28249
H	-5.10101	3.94749	-3.70117
H	-6.66947	3.39821	-1.85377
C	-0.23827	3.38704	0.18215
C	-0.03616	2.74399	-2.77612
H	0.04806	3.83147	-2.89167
H	-0.49940	2.33888	-3.67594
H	0.97302	2.33884	-2.68342
C	1.16490	3.46507	0.29395
C	1.76223	4.28265	1.25313
C	0.96858	5.04918	2.11157
C	-0.42302	4.99019	2.00971
C	-1.02029	4.16435	1.05537
H	1.79961	2.88380	-0.36590

H	2.84514	4.32278	1.32521
H	1.43184	5.68964	2.85651
H	-1.04320	5.58183	2.67629
H	-2.10289	4.14132	0.98635

[$(Et_3P)Ru(H)SDmp(SiMePh_2)]^{+‡}$ (**3aa**$^+$**_TS**)

Ru	1.50255	-0.71795	-0.37947
S	-0.83240	-0.30741	-0.37107
P	1.70755	0.39326	-2.40550
Si	0.08275	1.05247	1.41971
C	-1.46438	-1.88782	0.23122
C	-2.85838	-2.06774	0.36728
C	-3.32660	-3.34012	0.72645
C	-2.45390	-4.40224	0.96070
C	-1.07693	-4.20803	0.84193
C	-0.57884	-2.95705	0.47324
C	-3.82219	-0.93952	0.18355
C	-4.07156	-0.38999	-1.08953
C	-4.92486	0.71754	-1.20024
C	-5.54607	1.28714	-0.08823
C	-5.32297	0.69552	1.16228
C	-4.48890	-0.41369	1.31805
C	-3.47846	-0.96200	-2.35517
C	-6.45399	2.48448	-0.21889
C	-4.34562	-1.02925	2.69131
C	0.89125	-2.71838	0.33613
C	1.61371	-2.17174	1.45330
C	2.90053	-1.63107	1.20670
C	3.54912	-1.74893	-0.05132
C	2.84270	-2.42371	-1.08832
C	1.54186	-2.97037	-0.91627
C	1.05118	-2.20241	2.84539
C	4.95859	-1.26287	-0.24107
C	0.87291	-3.75489	-2.01032
C	-1.34747	0.64967	2.57976
C	1.59113	1.28352	2.57142
C	-0.31249	2.67905	0.55930
C	0.60065	3.74769	0.52480
C	0.28460	4.94837	-0.11714
C	-0.96026	5.10713	-0.73146
C	-1.89236	4.06590	-0.68345
C	-1.57100	2.86803	-0.04407

C	0.64988	1.88427	-2.62951
C	0.79533	2.61560	-3.96582
C	1.34989	-0.66032	-3.88252
C	-0.12667	-1.02545	-4.04138
C	3.42289	0.97284	-2.77287
C	3.97736	1.98390	-1.76987
H	1.72108	0.81873	0.02250
H	-4.39743	-3.48920	0.82246
H	-2.84439	-5.37662	1.23666
H	-0.38662	-5.02512	1.03003
H	-5.10996	1.13698	-2.18679
H	-5.81472	1.10844	2.04027
H	-4.26186	-1.10601	-3.10733
H	-2.99488	-1.92638	-2.19073
H	-2.73614	-0.28306	-2.78537
H	-7.47959	2.23711	0.07968
H	-6.48538	2.85230	-1.24853
H	-6.12046	3.30674	0.42476
H	-5.06490	-1.84567	2.83054
H	-4.54348	-0.28713	3.46980
H	-3.35204	-1.44639	2.86191
H	3.40615	-1.10686	2.01069
H	3.31599	-2.51902	-2.06095
H	-0.03048	-2.07240	2.86262
H	1.51458	-1.44367	3.47548
H	1.26337	-3.18343	3.28905
H	5.14282	-0.34665	0.32486
H	5.18533	-1.07634	-1.29322
H	5.66411	-2.02174	0.11889
H	1.35800	-3.58432	-2.97351
H	-0.18581	-3.50882	-2.10138
H	0.93943	-4.82738	-1.79106
H	-2.28424	0.74533	2.02968
H	-1.36808	1.37482	3.40086
H	-1.30774	-0.35298	3.00972
H	1.56895	3.65001	1.00318
H	1.00848	5.75836	-0.13347
H	-1.20670	6.03818	-1.23375
H	-2.86932	4.18506	-1.14342
H	-2.30704	2.07060	-0.02959
H	-0.38313	1.56151	-2.47672
H	0.87542	2.55549	-1.79874
H	1.80308	3.01924	-4.10316
H	0.09895	3.45989	-3.99430

H	0.56816	1.97235	-4.82167
H	1.71639	-0.13555	-4.77194
H	1.96216	-1.55995	-3.77253
H	-0.53519	-1.45074	-3.12338
H	-0.25463	-1.75782	-4.84504
H	-0.72639	-0.14769	-4.29810
H	4.05156	0.07702	-2.79964
H	3.42385	1.38715	-3.78703
H	3.34625	2.87567	-1.70326
H	4.97944	2.30709	-2.06994
H	4.04914	1.54558	-0.77201
C	2.85858	1.70436	2.12770
C	3.90980	1.93921	3.01409
C	3.72728	1.73916	4.38634
C	2.48254	1.31719	4.85458
C	1.43039	1.10145	3.95784
H	3.03583	1.84214	1.06713
H	4.87129	2.27487	2.63504
H	4.54499	1.91223	5.07969
H	2.32406	1.15743	5.91732
H	0.47669	0.77883	4.35849

[(Et$_3$P)Ru(H)SDmp(SiMe$_2$Ph)]$^{+‡}$ (**3ab$^+$_TS**)

Ru	-1.81144	-0.37313	-0.19044
S	0.52368	-0.21642	0.21003
P	-2.19154	1.35148	1.31086
Si	0.16478	0.58292	-2.04179
C	0.99721	-1.95602	0.22198
C	2.34996	-2.29278	0.44792
C	2.68607	-3.65153	0.52781
C	1.72357	-4.65121	0.38351
C	0.39030	-4.30585	0.15637
C	0.02127	-2.96141	0.07944
C	3.40246	-1.23754	0.56954
C	3.44770	-0.38864	1.69427
C	4.38461	0.65355	1.72723
C	5.28744	0.86821	0.68508
C	5.26280	-0.01970	-0.39831
C	4.35279	-1.07771	-0.46878
C	2.52840	-0.56007	2.88053
C	6.26853	2.01325	0.71624
C	4.44066	-2.03537	-1.63505

C	-1.39854	-2.55464	-0.16027
C	-1.84046	-2.32786	-1.51089
C	-3.05189	-1.61754	-1.69869
C	-3.90747	-1.27645	-0.61731
C	-3.49651	-1.66295	0.68878
C	-2.27825	-2.34266	0.95154
C	-1.07809	-2.87762	-2.68331
C	-5.23904	-0.62191	-0.86001
C	-1.92992	-2.79694	2.34104
C	1.60937	-0.39705	-2.74991
C	-1.06064	0.70511	-3.48969
C	0.70415	2.33153	-1.60964
C	-0.05384	3.44225	-2.02030
C	0.33407	4.74412	-1.69090
C	1.49606	4.95786	-0.94466
C	2.27387	3.86659	-0.54427
C	1.88049	2.56906	-0.87470
C	-0.89588	2.65806	1.39300
C	-1.13314	3.77950	2.40691
C	-2.39034	0.80977	3.06842
C	-1.08048	0.33815	3.70524
C	-3.75983	2.27573	0.99663
C	-3.78928	3.02497	-0.33690
H	-1.76403	0.96966	-1.05369
H	3.72240	-3.92045	0.70655
H	2.01062	-5.69588	0.44972
H	-0.36579	-5.07680	0.04039
H	4.40753	1.30793	2.59615
H	5.97690	0.11268	-1.20821
H	2.04893	-1.54087	2.89519
H	1.73968	0.19854	2.88083
H	3.08911	-0.44764	3.81431
H	7.30084	1.65547	0.62851
H	6.18838	2.58245	1.64674
H	6.09554	2.70302	-0.11849
H	5.05803	-2.90465	-1.37665
H	4.90822	-1.55180	-2.49730
H	3.46630	-2.41561	-1.94449
H	-3.34577	-1.34286	-2.70658
H	-4.14089	-1.41315	1.52602
H	-0.00241	-2.87713	-2.51710
H	-1.29790	-2.33519	-3.60400
H	-1.37982	-3.92145	-2.83736
H	-5.17339	0.14740	-1.63371

H	-5.63231	-0.16836	0.05241
H	-5.96707	-1.36995	-1.19655
H	-2.50080	-2.24451	3.09009
H	-0.86716	-2.68263	2.55611
H	-2.17140	-3.86075	2.45550
H	2.53098	-0.12180	-2.23293
H	1.72189	-0.15741	-3.81433
H	1.48753	-1.47841	-2.66481
H	-0.52042	1.15338	-4.33465
H	-1.94085	1.31566	-3.27642
H	-1.40402	-0.28055	-3.81167
H	-0.96373	3.29678	-2.59465
H	-0.26929	5.58784	-2.01405
H	1.79741	5.96819	-0.68330
H	3.18379	4.02540	0.02747
H	2.48966	1.73486	-0.54061
H	0.05132	2.15183	1.59517
H	-0.80519	3.06858	0.38624
H	-2.04878	4.34028	2.19561
H	-0.30033	4.48891	2.36125
H	-1.19376	3.40756	3.43408
H	-2.81481	1.64750	3.63375
H	-3.13922	0.01230	3.07927
H	-0.58258	-0.41132	3.08781
H	-1.26847	-0.10040	4.69061
H	-0.38613	1.17175	3.84262
H	-4.57278	1.54415	1.04386
H	-3.91061	2.96927	1.83113
H	-3.00348	3.78501	-0.38723
H	-4.75101	3.53215	-0.46486
H	-3.64925	2.34284	-1.17984

$[(Ar^F{}_3P)Ru(H)SDmp(SiMePh_2)]^{+\ddagger}$ (**3ba$^+$_TS**)

Ru	0.77029	-0.69774	-1.08597
S	-1.43118	-0.04150	-0.39786
P	1.90657	1.08537	0.04486
C	-2.39611	-0.35792	-1.90486
C	-3.76525	0.00485	-1.96679
C	-4.45339	-0.22667	-3.17007
C	-3.83322	-0.79780	-4.28039
C	-2.48108	-1.13380	-4.21638
C	-1.75916	-0.90553	-3.04064
C	-0.29046	-1.20287	-2.97953

C	0.16413	-2.47881	-2.50394
C	-0.73920	-3.68127	-2.44304
C	1.54368	-2.61859	-2.18919
C	2.49231	-1.60994	-2.46400
C	3.96498	-1.86542	-2.29855
C	2.00604	-0.39738	-3.04110
C	0.65050	-0.19510	-3.39506
C	0.24037	0.99745	-4.21859
C	-4.51869	0.63875	-0.83182
C	-4.31119	2.00664	-0.51687
C	-3.35919	2.86962	-1.31403
C	-5.04416	2.58745	0.52173
C	-5.99495	1.86491	1.25697
C	-6.76700	2.52024	2.37763
C	-6.21654	0.53341	0.90192
C	-5.51178	-0.09066	-0.14072
C	-5.89753	-1.50891	-0.50802
H	-5.50052	0.05620	-3.22649
H	-4.39629	-0.96791	-5.19312
H	-1.98101	-1.56557	-5.07907
H	-0.45851	-4.36190	-1.63755
H	-0.63586	-4.23178	-3.38777
H	-1.78979	-3.41257	-2.34200
H	1.88165	-3.55234	-1.74957
H	4.52327	-0.94502	-2.12241
H	4.35547	-2.31765	-3.21959
H	4.16287	-2.56350	-1.48185
H	2.71938	0.39154	-3.26307
H	-0.71205	1.42622	-3.90203
H	0.11935	0.68715	-5.26445
H	1.00660	1.77551	-4.19548
H	-2.32137	2.53601	-1.21379
H	-3.40631	3.90842	-0.97519
H	-3.60481	2.85529	-2.38268
H	-4.88014	3.63804	0.75357
H	-7.31192	3.40279	2.02206
H	-6.09951	2.85869	3.17959
H	-7.49506	1.83198	2.81689
H	-6.96939	-0.04082	1.43816
H	-6.75927	-1.51041	-1.18825
H	-6.19502	-2.07351	0.38147
H	-5.09647	-2.05632	-1.00660
H	0.86953	-1.28208	0.42090
Si	-0.93101	-2.16803	0.94384

C	3.73368	0.79384	0.00564
C	4.26427	-0.26802	0.76200
C	4.60819	1.54737	-0.79355
C	5.62953	-0.55106	0.75517
C	5.97864	1.26924	-0.82473
C	6.46006	0.22892	-0.04230
H	3.61025	-0.88228	1.37300
H	4.23601	2.36338	-1.40228
H	6.04609	-1.35894	1.34764
H	6.65949	1.84942	-1.43883
C	1.56507	1.49275	1.82345
C	0.25731	1.88522	2.16057
C	2.56974	1.59526	2.79825
C	-0.04441	2.36790	3.43223
C	2.28144	2.06574	4.08253
C	0.97868	2.44594	4.36880
H	-0.53948	1.83534	1.42638
H	3.59499	1.32907	2.57306
H	-1.05041	2.67380	3.69636
H	3.05328	2.14627	4.84104
C	1.69048	2.78962	-0.64832
C	2.45663	3.86299	-0.15269
C	0.68174	3.05583	-1.58010
C	2.24843	5.16229	-0.60948
C	0.45064	4.35463	-2.04478
C	1.24783	5.37774	-1.55353
H	3.21746	3.69103	0.60230
H	0.05614	2.24637	-1.93173
H	2.83535	5.99634	-0.23914
H	-0.33261	4.57149	-2.76347
F	7.78935	-0.04422	-0.06093
F	0.69159	2.91519	5.61146
F	1.03717	6.64240	-1.99929
C	-1.01712	-1.38192	2.65951
C	-2.19574	-0.72252	3.06468
C	-0.02958	-1.61423	3.63616
C	-2.38642	-0.32753	4.39033
C	-0.21212	-1.21095	4.96180
C	-1.39358	-0.57068	5.34412
H	-2.98352	-0.52062	2.34605
H	0.88419	-2.13849	3.37576
H	-3.31004	0.16798	4.67701
H	0.56448	-1.40656	5.69614
H	-1.54004	-0.26569	6.37660

C	-2.65223	-2.86226	0.57298
H	-2.91066	-3.56923	1.37088
H	-3.38912	-2.05970	0.59759
H	-2.74410	-3.38461	-0.38130
C	0.22036	-3.70388	1.05337
C	-0.36194	-4.98879	1.04022
C	1.60785	-3.63920	1.28838
C	0.39813	-6.14368	1.25899
C	2.37252	-4.78559	1.51806
C	1.76891	-6.04699	1.50278
H	-1.42476	-5.11076	0.86293
H	2.10870	-2.67580	1.29146
H	-0.08674	-7.11596	1.24131
H	3.43871	-4.69502	1.70958
H	2.36002	-6.94115	1.67955

$[(Et_3P)Ru(H)SDmp(SiMePh_2)]^+$ (**3aa**$^+$)

Ru	1.86996	-0.39111	-0.42286
S	-0.49685	-0.10701	-0.24077
P	2.20674	1.57722	-1.56356
Si	-0.98052	0.35238	1.91963
C	-1.11210	-1.72644	-0.82193
C	-2.47225	-1.92237	-1.14716
C	-2.84030	-3.16519	-1.69303
C	-1.91336	-4.17864	-1.91576
C	-0.56831	-3.95831	-1.62108
C	-0.16045	-2.73300	-1.09301
C	-3.54183	-0.89298	-0.97416
C	-3.52603	0.30914	-1.71281
C	-4.54869	1.24765	-1.51301
C	-5.60447	1.01864	-0.62991
C	-5.63291	-0.20554	0.04981
C	-4.63150	-1.16544	-0.10984
C	-2.48084	0.62198	-2.75630
C	-6.69359	2.04077	-0.42168
C	-4.74564	-2.45508	0.67176
C	1.28978	-2.46113	-0.85909
C	1.79818	-2.52948	0.49576
C	3.06419	-1.97497	0.75596
C	3.88088	-1.43317	-0.28103
C	3.39424	-1.52245	-1.61724
C	2.13449	-2.10056	-1.95440

C	0.98653	-3.16558	1.58679
C	5.25321	-0.89848	0.01939
C	1.72213	-2.25801	-3.39162
C	-2.75754	-0.07707	2.27591
C	0.12472	-0.43058	3.21462
C	-0.80250	2.21282	1.78643
C	0.14648	2.96142	2.50215
C	0.21405	4.35151	2.36671
C	-0.67296	5.01670	1.51705
C	-1.63491	4.28905	0.80877
C	-1.69631	2.90227	0.94254
C	0.97389	2.91126	-1.24839
C	1.19226	4.22659	-1.99934
C	2.25043	1.42560	-3.40829
C	0.88243	1.11994	-4.02173
C	3.83716	2.38401	-1.22238
C	4.03537	2.82534	0.22857
H	2.01451	0.69987	0.72830
H	-3.88036	-3.31892	-1.96029
H	-2.23276	-5.12582	-2.33874
H	0.17403	-4.72609	-1.81614
H	-4.52385	2.17435	-2.08248
H	-6.45220	-0.41396	0.73418
H	-2.96122	0.97009	-3.67698
H	-1.86920	-0.24469	-3.00866
H	-1.80860	1.41623	-2.41834
H	-7.67500	1.63527	-0.69366
H	-6.52123	2.93655	-1.02501
H	-6.75295	2.34879	0.62879
H	-5.25014	-3.23354	0.08694
H	-5.33728	-2.29936	1.57837
H	-3.77308	-2.85974	0.96182
H	3.41337	-1.93269	1.78245
H	4.01223	-1.13184	-2.41980
H	-0.04758	-2.81925	1.59059
H	1.42025	-2.96510	2.56656
H	0.96081	-4.25148	1.43442
H	5.27975	-0.39136	0.98706
H	5.58753	-0.19628	-0.74767
H	5.97918	-1.72053	0.05345
H	2.24749	-1.54529	-4.03117
H	0.64869	-2.12161	-3.52912
H	1.97210	-3.26627	-3.74462
H	-3.42988	0.43399	1.58741

H	-2.98673	0.27140	3.29036
H	-2.96274	-1.14760	2.22797
H	0.83579	2.46635	3.17775
H	0.95620	4.91282	2.92706
H	-0.61992	6.09636	1.41164
H	-2.33270	4.80132	0.15248
H	-2.44322	2.35166	0.37591
H	-0.00655	2.49074	-1.48529
H	0.97335	3.07724	-0.16898
H	2.13552	4.70621	-1.72076
H	0.38697	4.92708	-1.75554
H	1.19158	4.08941	-3.08522
H	2.66091	2.35310	-3.82358
H	2.96770	0.63183	-3.64155
H	0.41255	0.25786	-3.54342
H	0.97707	0.90510	-5.09123
H	0.20230	1.96954	-3.91533
H	4.61074	1.66547	-1.51370
H	3.93449	3.24015	-1.89938
H	3.27570	3.55129	0.53523
H	5.01549	3.29831	0.35105
H	3.97799	1.97664	0.91522
C	1.49038	-0.13197	3.37393
C	2.24360	-0.71017	4.39667
C	1.64662	-1.60595	5.28849
C	0.29005	-1.91148	5.15459
C	-0.45947	-1.32671	4.13165
H	1.97836	0.54154	2.68063
H	3.29577	-0.45908	4.49935
H	2.23192	-2.05686	6.08449
H	-0.18477	-2.60174	5.84561
H	-1.51144	-1.58199	4.05199

$[(Et_3P)Ru(H)SDmp(SiMe_2Ph)]^+$ (**3ab**$^+$)

Ru	-1.95540	-0.47889	-0.26235
S	0.41783	-0.18832	-0.15195
P	-2.38787	1.21689	1.24128
Si	1.04600	0.74962	-2.10383
C	0.99768	-1.90260	0.08205
C	2.33196	-2.17855	0.44988
C	2.67181	-3.51696	0.71448
C	1.73866	-4.54612	0.63205

C	0.41498	-4.25249	0.30550
C	0.03636	-2.93466	0.04533
C	3.39221	-1.13781	0.61548
C	3.30448	-0.17081	1.64007
C	4.32378	0.78330	1.76739
C	5.44229	0.79040	0.93232
C	5.53563	-0.20885	-0.04434
C	4.54128	-1.17545	-0.21178
C	2.17602	-0.13536	2.64236
C	6.53445	1.81847	1.08968
C	4.73493	-2.22761	-1.28007
C	-1.39392	-2.59923	-0.22721
C	-1.80297	-2.37555	-1.59766
C	-3.05507	-1.77389	-1.82598
C	-3.94745	-1.45703	-0.75837
C	-3.55482	-1.83068	0.55911
C	-2.31692	-2.47111	0.85691
C	-0.90463	-2.77675	-2.73285
C	-5.29899	-0.85657	-1.02979
C	-2.00264	-2.92369	2.25534
C	2.81043	0.33780	-2.54148
C	-0.06401	0.37215	-3.56189
C	0.88312	2.53377	-1.56485
C	-0.11070	3.37768	-2.08888
C	-0.21266	4.70933	-1.67585
C	0.67941	5.21588	-0.72699
C	1.67485	4.38937	-0.19516
C	1.77323	3.06125	-0.61084
C	-1.12437	2.55415	1.33644
C	-1.39312	3.67022	2.34781
C	-2.60240	0.67750	3.00061
C	-1.29350	0.23136	3.65710
C	-3.97296	2.11896	0.92858
C	-3.99979	2.91114	-0.37973
H	-2.00839	0.81999	-1.19488
H	3.69203	-3.73854	1.00948
H	2.03464	-5.56813	0.84666
H	-0.33227	-5.03931	0.26812
H	4.24595	1.52678	2.55798
H	6.40465	-0.23285	-0.69847
H	2.57069	0.03036	3.65009
H	1.60444	-1.06457	2.65809
H	1.48014	0.67992	2.42437
H	7.48194	1.34627	1.37495

H	6.28229	2.55366	1.85907
H	6.71304	2.35729	0.15204
H	5.23014	-3.11795	-0.87382
H	5.36942	-1.84417	-2.08406
H	3.79190	-2.56152	-1.71792
H	-3.34095	-1.52202	-2.84257
H	-4.22946	-1.60934	1.38025
H	0.12198	-2.43517	-2.58723
H	-1.27503	-2.38801	-3.68261
H	-0.86869	-3.87033	-2.80381
H	-5.25144	-0.10866	-1.82562
H	-5.70844	-0.38351	-0.13414
H	-6.00349	-1.63619	-1.34479
H	-2.60071	-2.37426	2.98551
H	-0.94791	-2.79668	2.50233
H	-2.23865	-3.98898	2.36801
H	3.52485	0.63366	-1.77296
H	3.04469	0.89599	-3.45772
H	2.94604	-0.72415	-2.76012
H	0.07026	1.17739	-4.29563
H	-1.12070	0.31976	-3.29640
H	0.23422	-0.55886	-4.04929
H	-0.82048	2.99590	-2.81686
H	-0.98728	5.34790	-2.09066
H	0.60143	6.24996	-0.40418
H	2.37108	4.77989	0.54154
H	2.54313	2.42734	-0.17859
H	-0.16800	2.06750	1.54282
H	-1.04042	2.96260	0.32787
H	-2.31687	4.21315	2.12484
H	-0.57436	4.39647	2.31888
H	-1.46362	3.29355	3.37282
H	-3.05385	1.50293	3.56302
H	-3.33356	-0.13750	2.99742
H	-0.77161	-0.50656	3.04470
H	-1.48506	-0.21575	4.63809
H	-0.61719	1.07759	3.80733
H	-4.76795	1.36573	0.93513
H	-4.15647	2.78094	1.78231
H	-3.24099	3.69974	-0.38617
H	-4.97643	3.38777	-0.51452
H	-3.81236	2.26317	-1.24034

[(Ar$^F{}_3$P)Ru(H)SDmp(SiMePh$_2$)]$^+$ (**3ba**$^+$)

Ru	-1.20042	-1.29282	-0.61088
S	1.07977	-0.59772	-0.30073
P	-2.06113	0.32512	0.81212
C	1.97089	-2.18586	-0.44304
C	3.30868	-2.32613	-0.01834
C	3.92905	-3.57182	-0.22519
C	3.25888	-4.64277	-0.80934
C	1.90798	-4.51493	-1.13406
C	1.25486	-3.29931	-0.92682
C	-0.23394	-3.22257	-1.01989
C	-0.89840	-2.86191	-2.23338
C	-0.18110	-2.82510	-3.55156
C	-2.28557	-2.54897	-2.14910
C	-3.02106	-2.68911	-0.94712
C	-4.50686	-2.47721	-0.90893
C	-2.33117	-3.16889	0.21009
C	-0.96808	-3.51397	0.19175
C	-0.30535	-4.14780	1.38502
C	4.07084	-1.27564	0.72149
C	3.63395	-0.88408	2.01441
C	2.42696	-1.50237	2.67895
C	4.37120	0.06696	2.72381
C	5.54651	0.62958	2.21132
C	6.33246	1.64185	3.00604
C	5.98521	0.19506	0.96150
C	5.28423	-0.76137	0.21440
C	5.90338	-1.20636	-1.09145
H	4.95592	-3.69329	0.10396
H	3.77157	-5.58697	-0.96336
H	1.34682	-5.36397	-1.51247
H	-0.72523	-2.21284	-4.27165
H	-0.10630	-3.84177	-3.95720
H	0.83371	-2.43913	-3.45757
H	-2.79465	-2.18757	-3.03643
H	-4.83023	-2.05463	0.04409
H	-5.01342	-3.44245	-1.03505
H	-4.83849	-1.81761	-1.71190
H	-2.89239	-3.28863	1.13217
H	0.59985	-3.62000	1.69098
H	-0.01239	-5.17686	1.14793
H	-0.99470	-4.18748	2.23187
H	1.48895	-1.09795	2.28743

H	2.44260	-1.30604	3.75418
H	2.40577	-2.58693	2.53614
H	4.02980	0.36324	3.71299
H	6.93204	2.28382	2.35385
H	7.02311	1.14413	3.69852
H	5.67606	2.27766	3.60879
H	6.90254	0.60744	0.54772
H	6.80975	-1.79331	-0.89869
H	6.20942	-0.34119	-1.68910
H	5.24199	-1.82570	-1.69599
H	-1.37207	-0.04178	-1.55322
Si	1.79667	0.65219	-2.06351
C	-3.87643	0.49883	0.60921
C	-4.37381	0.79009	-0.67313
C	-4.77726	0.32363	1.66757
C	-5.74145	0.93591	-0.89265
C	-6.15439	0.44173	1.45900
C	-6.60386	0.75305	0.18342
H	-3.68743	0.89514	-1.50740
H	-4.41636	0.08933	2.66259
H	-6.13939	1.16995	-1.87424
H	-6.86470	0.30254	2.26667
C	-1.46279	2.06187	0.64882
C	-0.12620	2.34314	0.97781
C	-2.29357	3.11459	0.24005
C	0.37925	3.63778	0.88120
C	-1.80311	4.41856	0.13713
C	-0.47240	4.64887	0.45606
H	0.53513	1.55402	1.31749
H	-3.33370	2.93570	0.00144
H	1.41418	3.85801	1.11108
H	-2.43862	5.23879	-0.17904
C	-1.81849	0.07598	2.61683
C	-2.07430	1.11386	3.53051
C	-1.34471	-1.14901	3.09676
C	-1.87210	0.92605	4.89621
C	-1.13345	-1.35778	4.46111
C	-1.40365	-0.31035	5.33059
H	-2.42643	2.07737	3.17764
H	-1.12262	-1.93990	2.39502
H	-2.06282	1.71685	5.61347
H	-0.75642	-2.30025	4.84317
F	-7.94002	0.87742	-0.02630
F	0.01584	5.91278	0.35427

F	-1.19263	-0.49405	6.65892
C	2.59039	2.17170	-1.31030
C	3.29598	2.13635	-0.09642
C	2.53621	3.39232	-2.01175
C	3.92562	3.27793	0.40299
C	3.15874	4.53776	-1.51138
C	3.85569	4.48268	-0.30140
H	3.34770	1.21841	0.47405
H	1.99485	3.45878	-2.95071
H	4.46541	3.22468	1.34275
H	3.09409	5.47163	-2.06186
H	4.33615	5.37396	0.09158
C	3.00397	-0.38906	-3.03914
H	3.96555	-0.41550	-2.52812
H	2.67323	-1.41806	-3.19996
H	3.16476	0.08291	-4.01560
C	0.33101	1.16706	-3.10235
C	0.04607	0.52283	-4.31752
C	-0.52275	2.20394	-2.68459
C	-1.07302	0.87611	-5.07529
C	-1.64131	2.56194	-3.43642
C	-1.92479	1.89079	-4.63023
H	0.69938	-0.26303	-4.68177
H	-0.31319	2.73539	-1.76508
H	-1.27554	0.36673	-6.01333
H	-2.28743	3.36454	-3.09321
H	-2.79469	2.16813	-5.21885

[(Et$_3$P)Ru(SiMe$_2$Ph)S(H)Dmp]$^{+‡}$ (**4ab$^+$_TS**)

Ru	-1.30895	-0.90102	-0.11224
S	1.01010	0.03787	-0.05716
P	-1.82003	0.40458	1.78445
Si	-2.07056	0.79105	-1.74776
C	1.94044	-1.50368	-0.23894
C	3.34261	-1.40921	-0.23587
C	4.08378	-2.59568	-0.32549
C	3.45228	-3.83558	-0.42148
C	2.05822	-3.91141	-0.42655
C	1.29188	-2.74742	-0.32823
C	4.02331	-0.08365	-0.12903
C	4.48709	0.36342	1.12518
C	5.10453	1.61633	1.20958

C	5.27852	2.42959	0.08370
C	4.81323	1.96096	-1.15009
C	4.18751	0.71613	-1.27794
C	4.32577	-0.48597	2.36347
C	5.97869	3.76126	0.19125
C	3.68105	0.26080	-2.62484
C	-0.20148	-2.79987	-0.33719
C	-0.88943	-2.62332	-1.58980
C	-2.28462	-2.39061	-1.54259
C	-3.01434	-2.40034	-0.31797
C	-2.30036	-2.74114	0.87026
C	-0.91779	-3.03464	0.88317
C	-0.13827	-2.67574	-2.89235
C	-4.50710	-2.22858	-0.29118
C	-0.23034	-3.54594	2.11732
C	-1.05521	0.61342	-3.35701
C	-3.86143	0.64059	-2.37095
C	-1.81624	2.61386	-1.30213
C	-2.89566	3.47252	-1.02635
C	-2.69296	4.80992	-0.67265
C	-1.39527	5.32306	-0.59377
C	-0.30745	4.49632	-0.89145
C	-0.52084	3.16241	-1.24414
C	-1.28644	2.17006	1.88096
C	-1.37921	2.83345	3.25777
C	-1.04479	-0.33247	3.30375
C	0.48317	-0.29427	3.34422
C	-3.59040	0.41915	2.32082
C	-4.52467	1.19986	1.40016
H	5.16760	-2.53268	-0.32440
H	4.04364	-4.74280	-0.49443
H	1.56207	-4.87403	-0.50469
H	5.46085	1.96249	2.17710
H	4.94276	2.57727	-2.03659
H	4.65872	0.05672	3.25210
H	4.91066	-1.41038	2.29716
H	3.28322	-0.78305	2.52167
H	7.05974	3.64650	0.04156
H	5.83291	4.21343	1.17737
H	5.61781	4.46556	-0.56472
H	3.97889	-0.76900	-2.84625
H	4.06097	0.90404	-3.42292
H	2.58548	0.29723	-2.66804
H	-2.81423	-2.19570	-2.46810

H	-2.85261	-2.78742	1.80423
H	0.67399	-1.94725	-2.93619
H	-0.80852	-2.49891	-3.73437
H	0.31202	-3.66649	-3.01764
H	-4.86258	-1.68586	-1.16763
H	-4.83205	-1.69142	0.60235
H	-4.99393	-3.21163	-0.28317
H	-0.87810	-3.46215	2.99211
H	0.70775	-3.02769	2.32068
H	0.01188	-4.60719	1.98669
H	0.02939	0.69058	-3.21860
H	-1.34489	1.42601	-4.03498
H	-1.25726	-0.33174	-3.86850
H	-4.03310	1.44693	-3.09530
H	-4.63730	0.71804	-1.60601
H	-4.01505	-0.30128	-2.90733
H	-3.91295	3.09608	-1.08022
H	-3.54534	5.44957	-0.46025
H	-1.23360	6.36067	-0.31625
H	0.70419	4.89043	-0.84849
H	0.34419	2.54446	-1.47096
H	-0.26214	2.21418	1.50065
H	-1.89327	2.71992	1.16307
H	-2.39526	2.80082	3.66427
H	-1.10107	3.88842	3.16425
H	-0.70489	2.37983	3.98930
H	-1.45751	0.21197	4.16018
H	-1.40898	-1.35977	3.38771
H	0.92113	-0.86830	2.52718
H	0.84671	-0.71775	4.28642
H	0.86440	0.72818	3.26923
H	-3.89970	-0.62992	2.39074
H	-3.62444	0.82264	3.33878
H	-4.27034	2.26313	1.38194
H	-5.56165	1.11016	1.73999
H	-4.46900	0.82568	0.37736
H	0.14265	-0.05446	-1.16481

$[(Et_3P)Ru(SiMe_2Ph)S(H)Dmp]^{+‡}$ (**4ab**$^+$)

Ru	-1.31804	-0.86530	-0.15062
S	0.88730	-0.07764	-0.01530
P	-1.90500	0.43038	1.71528

Si	-1.76808	0.90276	-1.78940
C	1.85339	-1.61996	-0.11448
C	3.25367	-1.54660	-0.06993
C	3.96997	-2.75110	-0.09508
C	3.30857	-3.97905	-0.15741
C	1.91409	-4.02810	-0.21565
C	1.17323	-2.84311	-0.20064
C	3.93816	-0.22121	-0.00750
C	4.26195	0.33892	1.24434
C	4.84754	1.60855	1.28469
C	5.12168	2.33113	0.11728
C	4.79987	1.74869	-1.11381
C	4.21165	0.48137	-1.19822
C	3.97516	-0.40980	2.52262
C	5.78169	3.68556	0.18422
C	3.88630	-0.11294	-2.54821
C	-0.31531	-2.82446	-0.33248
C	-0.87471	-2.56920	-1.63842
C	-2.25588	-2.28270	-1.71964
C	-3.09591	-2.26842	-0.56949
C	-2.51094	-2.65620	0.67171
C	-1.15267	-3.04016	0.80612
C	-0.00432	-2.61752	-2.86429
C	-4.57346	-2.01023	-0.67320
C	-0.62386	-3.63283	2.08152
C	-0.64443	0.70558	-3.32243
C	-3.51157	0.88743	-2.55495
C	-1.45281	2.70760	-1.28809
C	-2.51173	3.60417	-1.05433
C	-2.28095	4.92226	-0.64937
C	-0.97261	5.38056	-0.47206
C	0.09822	4.51873	-0.72748
C	-0.14495	3.20579	-1.13570
C	-1.26230	2.15077	1.91138
C	-1.41880	2.78218	3.29702
C	-1.31873	-0.39438	3.27152
C	0.19944	-0.50853	3.39869
C	-3.71156	0.55850	2.11014
C	-4.51872	1.47392	1.19234
H	5.05447	-2.71472	-0.06137
H	3.88114	-4.90113	-0.17038
H	1.39974	-4.98152	-0.28654
H	5.09277	2.04358	2.25085
H	5.01244	2.29264	-2.03103

H	4.22416	0.19942	3.39527
H	4.55497	-1.33792	2.58350
H	2.91991	-0.69124	2.59701
H	6.87408	3.58583	0.21623
H	5.47870	4.23448	1.08145
H	5.53505	4.29528	-0.69040
H	4.41962	-1.05588	-2.71155
H	4.16005	0.57372	-3.35330
H	2.81779	-0.33732	-2.65723
H	-2.68422	-2.05130	-2.68842
H	-3.14741	-2.68608	1.55126
H	0.83424	-1.92039	-2.79813
H	-0.58004	-2.38094	-3.76011
H	0.41732	-3.62141	-2.98442
H	-4.80699	-1.35849	-1.51607
H	-4.95951	-1.54863	0.23925
H	-5.10984	-2.95605	-0.81920
H	-1.28086	-3.41871	2.92634
H	0.38163	-3.28375	2.31912
H	-0.57110	-4.72365	1.97835
H	0.43200	0.69492	-3.11779
H	-0.82756	1.56323	-3.98189
H	-0.87867	-0.19966	-3.88878
H	-3.59425	1.72562	-3.25826
H	-4.32935	0.97919	-1.83657
H	-3.68043	-0.02771	-3.13177
H	-3.53782	3.27115	-1.17934
H	-3.12006	5.58962	-0.47209
H	-0.78881	6.40238	-0.15293
H	1.12003	4.86967	-0.61143
H	0.71203	2.56838	-1.33687
H	-0.21138	2.13305	1.61027
H	-1.77020	2.76065	1.16577
H	-2.46419	2.80950	3.62134
H	-1.06203	3.81685	3.26192
H	-0.83980	2.26037	4.06431
H	-1.72721	0.16906	4.11684
H	-1.78522	-1.38223	3.30185
H	0.62631	-1.09447	2.58267
H	0.46985	-1.00488	4.33665
H	0.68066	0.47361	3.39293
H	-4.10441	-0.46393	2.07960
H	-3.79375	0.89401	3.15020
H	-4.16148	2.50610	1.24124

H	-5.57319	1.47179	1.48785
H	-4.46175	1.14782	0.15283
H	1.24766	0.37976	-1.23887

9 References

[S1] Y. Ohki, Y. Takikawa, H. Sadohara, C. Kesenheimer, B. Engendahl, E. Kapatina and K. Tatsumi, *Chem. Asian J.*, 2008, **3**, 1625–1635.

[S2] T. Stahl, H. F. T. Klare and M. Oestreich, *J. Am. Chem. Soc.*, 2013, **135**, 1248–1251.

[S3] H. F. T. Klare, M. Oestreich, J.-i. Ito, H. Nishiyama, Y. Ohki and K. Tatsumi, *J. Am. Chem. Soc.*, 2011, **133**, 3312–3315.

[S4] R. K. Harris, E. D. Becker, S. M. Cabral de Menezes, R. Goodfellow and P. Granger, *Pure Appl. Chem.*, 2001, **73**, 1795–1818.

[S5] (*a*) C. Lee, W. Yang and R. G. Parr, *Phys. Rev. B*, 1988, **37**, 785–789; (*b*) A. D. Becke, *J. Chem. Phys.*, 1993, **98**, 5648–5652; (*c*) P. J. Stephens, F. J. Devlin, C. F. Chabalowski and M. J. Frisch, *J. Phys. Chem.*, 1994, **98**, 11623–11627 and references cited therein.

[S6] S. Grimme, J. Antony, S. Ehrlich and H. Krieg, *J. Chem. Phys.*, 2010, **132**, 154104.

[S7] S. Grimme, S. Ehrlich and L. Goerigk, *J. Comp. Chem.*, 2011, **32**, 1456–1465.

[S8] M. J. Frisch, G. W. Trucks, H. B. Schlegel, G. E. Scuseria, M. A. Robb, J. R. Cheeseman, G. Scalmani, V. Barone, B. Mennucci, G. A. Petersson, H. Nakatsuji, M. Caricato, X. Li, H. P. Hratchian, A. F. Izmaylov, J. Bloino, G. Zheng, J. L. Sonnenberg, M. Hada, M. Ehara, K. Toyota, R. Fukuda, J. Hasegawa, M. Ishida, T. Nakajima, Y. Honda, O. Kitao, H. Nakai, T. Vreven, J. A. Montgomery, Jr., J. E. Peralta, F. Ogliaro, M. Bearpark, J. J. Heyd, E. Brothers, K. N. Kudin, V. N. Staroverov, R. Kobayashi, J. Normand, K. Raghavachari, A. Rendell, J. C. Burant, S. S. Iyengar, J. Tomasi, M. Cossi, N. Rega, N. J. Millam, M. Klene, J. E. Knox, J. B. Cross, V. Bakken, C. Adamo, J. Jaramillo, R. Gomperts, R. E. Stratmann, O. Yazyev, A. J. Austin, R. Cammi, C. Pomelli, J. W. Ochterski, R. L. Martin, K. Morokuma, V. G. Zakrzewski, G. A. Voth, P. Salvador, J. J. Dannenberg, S. Dapprich, A. D. Daniels, Ö. Farkas, J. B. Foresman, J. V. Ortiz, J. Cioslowski and D. J. Fox, GAUSSIAN 09 (Revision D.01), Gaussian Inc., Wallingford CT, 2009.

[S9] D. Andrae, U. Häussermann, M. Dolg, H. Stoll and H. Preuss, *Theor. Chim. Acta*, 1990, **77**, 123–141.

[S10] A. V. Marenich, C. J. Cramer and D. G. Truhlar, *J. Phys. Chem. B*, 2009, **113**, 6378–6396.

[S11] A. E. Reed, L. A. Curtiss and F. Weinhold, *Chem. Rev.*, 1988, **88**, 899–926.

[S12] K. A. Wiberg, *Tetrahedron*, 1968, **24**, 1083–1096.

[S13] E. D. Glendening, A. E. Reed, J. E. Carpenter and F. Weinhold, NBO version 3.1.

[S14] (*a*) M. Kohout, *Int. J. Quantum Chem.*, 2004, **97**, 651–658; (*b*) M. Kohout, K. Pernal, F. R. Wagner and Y. Grin, *Theor. Chem. Acc.*, 2004, **112**, 453–459.

[S15] M. Kohout, DGrid, version 4.6, Radebeul, 2011.

[S16] Paraview, version 4.2, Kitware Inc., Clifton Park, New York, USA, 2014 (available from http://www.paraview.org).

[S17] ReSpect, version 3.3.0, 2014; Relativistic Spectroscopy DFT program written by M. Repiský, S. Komorovský, V. G. Malkin, O. L. Malkina, M. Kaupp and K. Ruud with contributions from R. Bast, U. Ekstrom, S. Knecht, I. Malkin Ondik and E. Malkin (see http://rel-qchem.sav.sk).

[S18] S. Komorovský, M. Repiský, O. L. Malkina and V. G. Malkin, *J. Chem. Phys.*, 2010, **132**, 154101.

[S19] P. Hrobárik, V. Hrobáriková, F. Meier, M. Repiský, S. Komorovský and M. Kaupp, *J. Phys. Chem. A*, 2011, **115**, 5654–5659.

[S20] (*a*) J. P. Perdew, K. Burke and M. Ernzerhof, *Phys. Rev. Lett.*, 1996, **77**, 3865–3868; (*b*) J. P. Perdew, K. Burke and M. Ernzerhof, *Phys. Rev. Lett.*, 1997, **78**, 1396.

[S21] K. G. Dyall, *Theor. Chem. Acc.*, 2007, **117**, 483–489.

[S22] W. Kutzelnigg, U. Fleischer and M. Schindler in *NMR Basic Principles and Progress*, ed. P. Diehl, E. Fluck, H. Günther, R. Kosfeld and J. Seelig, Springer-Verlag, Berlin Heidelberg, 1991, vol. 23, pp. 165–262.

[S23] E. J. Moore, J. M. Sullivan and J. R. Norton, *J. Am. Chem. Soc.*, 1986, **108**, 2257–2263.

[S24] This work.

[S25] D. J. Harrison, D. R. Edwards, R. McDonald and L. Rosenberg, *Dalton Trans.*, 2008, 3401–3411.

[S26] CrystalClear, Rigaku Corporation, Tokyo, Japan, 1999.

[S27] CrystalStructure 4.0, Crystal Structure Analysis Package, Rigaku Corporation, Tokyo, Japan, 2000–2010.

[S28] G. M. Sheldrick, *Acta Crystallogr., Sect. A*, 2008, **64**, 112–122.

[S29] L. J. Farrugia, *J. Appl. Crystallogr.*, 1997, **30**, 565.
